# Dual Activation of H_2_ and CO_2_ by a Pincer‐Type Ni–Zn Heterobimetallic Complex

**DOI:** 10.1002/anie.9254970

**Published:** 2026-06-20

**Authors:** Krishnendu Dey, Matthias Borowski, Gregor Schnakenburg, Connie C. Lu

**Affiliations:** ^1^ Institut für Anorganische Chemie Universität Bonn Bonn Germany

**Keywords:** CO_2_ hydrogenation, dihydride, heterobimetallic, Lewis acid, nickel, zinc

## Abstract

Various Ni‐based metalloenzymes employ synergistic heterometallic cooperativity where a Ni center and a second metal ion cofacilitate small‐molecule conversion. Inspired by these biological systems, synthetic model complexes have been developed that exploit bimetallic cooperativity to achieve analogous reactivity. In this work, we report a bimetallic Ni−Zn complex comprising a pincer‐type diamido‐diaminodiphosphine ligand, in which a weakly Lewis acidic Zn(II) center stabilizes a T‐shaped Ni(0) site. Subsequent reactivity with H_2_ led to the isolation of a rare Ni dihydride complex, which dynamically interconverts between Zn(*μ*‐H)Ni(H) and *trans*‐Ni(H)_2_ motifs via rotational rocking about the P−Ni−P axis. The Ni dihydride complex is a competent alkene hydrogenation catalyst (5 mol% loading, 70°C, 2 bar H_2_) for terminal olefins. Also, the Ni dihydride inserts CO_2_ to give a rare Ni hydrido formate. This product further reacts with a second equivalent of CO_2_ through a carboxylate shift mechanism that unveils a nucleophilic Zn(II) amide group that inserts CO_2_ to give carbamate. Collectively, our experimental and computational results highlight the role of Zn(II) as a weakly Lewis acidic partner, which promotes the dual activation of H_2_ and CO_2_ by a single Ni center in the absence of any external base.

## Graphical abstract

A bimetallic Ni−Zn complex performs sequential activations of H_2_ (1 equiv) and CO_2_ (2 equiv). This bimetallic cooperativity is attributed to the weak Lewis acidic nature of Zn(II), which promotes fluxional ligand binding. Namely, an X‐type ligand at Ni, where X is hydride or formate, toggles between two different binding modes: bridging Zn(*μ*‐X)Ni and terminal Ni−X. The rocking motion ultimately sets off two sequential CO_2_ insertions.

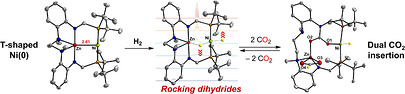

## 1 Introduction

**FIGURE 1:**
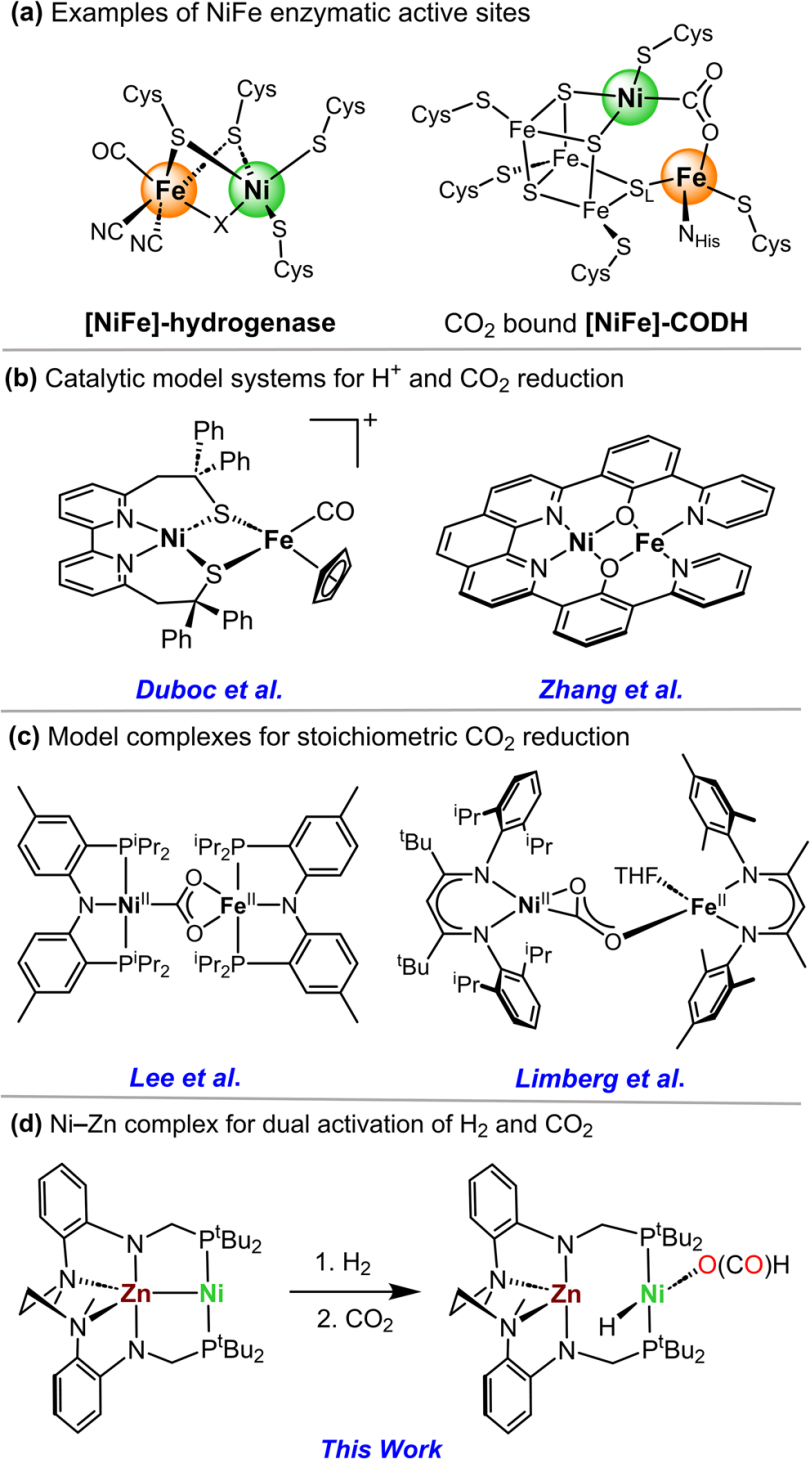
(a) Enzymatic active site of [NiFe]‐hydrogenase [1, 2] and [NiFe]‐carbon monoxide dehydrogenase (CODH) [3]. (b) Catalytic model systems for H^+^ and CO_2_ reduction [10, 11]. (c) Model complexes for stoichiometric CO_2_ reduction [12, 14]. (d) Ni−Zn heterobimetallic complex for dual activation of H_2_ and CO_2_ (this work).

Several nickel‐containing metalloenzymes, such as [NiFe]‐hydrogenase, [NiFe]‐carbon monoxide dehydrogenase (CODH), and acetyl coenzyme A synthase, feature active sites with a redox‐active Ni center in close proximity to a second metal center (Figure 1a) [1, 2, 3, 4, 5]. Motivated to understand and to mimic the activity of these metalloenzymes, many Ni‐based heterobimetallic systems have been developed to exploit bimetallic cooperativity in the activation of H_2_ or CO_2_ [6, 7, 8, 9, 10, 11, 12, 13, 14]. H_2_ evolution from weak acids at low overpotentials and the reverse reaction, H_2_ oxidation, have been successfully catalyzed with Ni–Fe synthetic model complexes (Figure 1b, *left*) [8, 11]. This cooperativity concept has also been extended to Ni‐main group and Ni‐lanthanide catalysts that exploit direct metal‐metal cooperativity to activate H_2_ for olefin and alkyne (semi)hydrogenation [15, 16, 17, 18, 19, 20, 21, 22, 23].

Moving to CO_2_, only one heterobimetallic Ni‐based catalyst system for CO_2_ reduction has been reported to date [10, 24, 25, 26]. Namely, the NiFe catalyst (Figure 1b, *right*) in combination with Ru(bpy)_3_ as a photosensitizer generates CO in high selectivity under irradiation [10]. Another Ni–Ga catalyst was shown to hydrogenate CO_2_ to formate, but a high pressure of a H_2_/CO_2_ gas mixture and a strong stoichiometric base were needed for high turnovers [27]. Of biological relevance, two NiFe heterobimetallic systems have demonstrated stoichiometric CO_2_ reduction to CO via bridging carboxylate intermediates that are akin to one observed for the C‐cluster in [NiFe]‐CODH [28] (Figure 1c) [12, 14].

Considering the widespread role of Zn(II) as a Lewis acid in enzymes, we began to investigate the Ni‐Zn pairing (Figure 1d). While Ni–Zn(II) heterobimetallic complexes are already known, the reported reactivity has been largely limited to H_2_ activation [29, 30, 31, 32, 33]. Using a new ligand framework, [((*o*‐NCH_2_P^t^Bu_2_‐C_6_H_4_)NMeCH_2_)_2_]^2−^ (abbreviated as **L** ^2−^), a Ni–Zn complex was isolated featuring a T‐shaped Ni(0) center bound to Zn(II). The reactivity of the Ni–Zn system with small molecules (H_2_, CO, and CO_2_) was investigated, and the products were characterized by X‐ray crystallography, variable‐temperature NMR spectroscopy, and IR spectroscopy. Density functional theory (DFT) calculations were also performed to provide further insight into the reaction mechanisms.

A highlight of this work is the formation of a Ni hydrido formate complex from the dual activation of H_2_ and CO_2_ without any external base. While H_2_ activation by heterobimetallic Ni systems has been established, the subsequent reactivity of the resulting Ni hydrides is largely limited to olefin hydrogenation, with no reports of base‐free CO_2_/carbonyl reactivity thus far. Indeed, among the late first‐row transition metals, well‐defined metal dihydrides that can directly insert CO_2_ are only reported for Fe [34, 35, 36, 37, 38, 39, 40, 41, 42]. After formate formation, another CO_2_ equivalent is inserted into a Zn−N_amide_ bond. The DFT‐calculated mechanism reveals that this insertion is triggered by a change in the binding mode of the formate ligand. The latter is reminiscent of the carboxylate‐shift mechanism in metalloenzymatic active sites. The Zn(II) co‐active site plays an important functional role and enables the unique reactivity of this Ni–Zn system.

## 2 Results and Discussion

### 2.1 Synthesis and Characterization of Ni–Zn Bimetallic Compound

Entry into the Ni–Zn heterobimetallic system began with the salt metathesis of the lithiated ligand Li_2_ **L** (**1**) and ZnCl_2_ to obtain the neutral Zn metalloligand **2** as white powder in good yield (78%, see Supporting Information Experimental Section). Consistent with *C*_2_ symmetry in solution, the NMR spectra of **2** displayed a single ^31^P{^1^H} NMR signal (28.8 ppm) and two sets of diastereotopic methylene protons, one for C*H*_2_P^t^Bu_2_ and one for the ethylene backbone of the ligand (Figures S28, S29).

**FIGURE 2:**
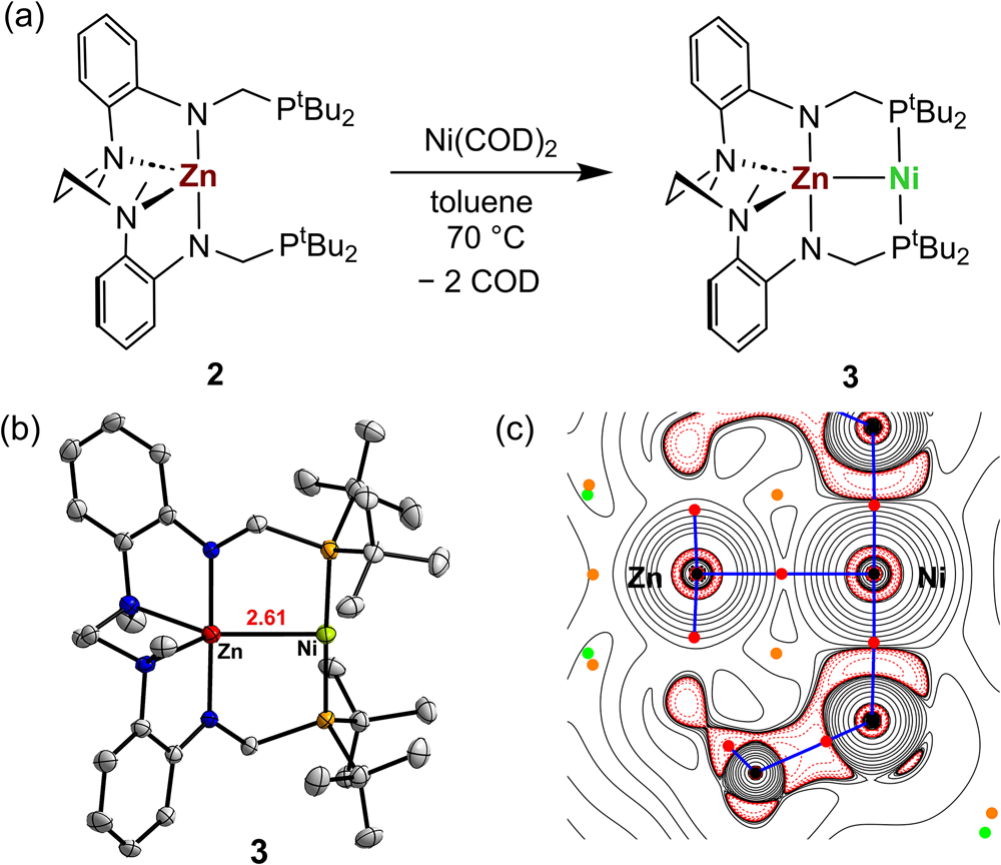
(a) Synthesis of the Ni–Zn bimetallic complex **3**. (b) Molecular structure of **3** shown at 50% thermal ellipsoid probability. One of two independent molecules is shown. H atoms have been omitted for clarity. The average Ni−Zn bond length (Å) is shown in red. (c) QTAIM molecular graphs of **3** showing key bond critical points (bcps, in red) and ring critical points (rcps, in orange).

Next, **2** was reacted with Ni(COD)_2_, where COD is 1,5‐cyclooctadiene, to yield the bimetallic Ni–Zn complex (**3**), which was isolated as a dark‐red solid (65% yield, Figure 2a). Like **2**, complex **3** displays a *C*_2_ symmetric solution‐state structure, as evidenced by the single ^31^P{^1^H} NMR signal (61 ppm) and the two sets of diastereotopic methylene protons (Figures S35, S36). The UV‐Vis spectrum of **3** shows three overlapping bands in the visible region from 440 to 560 nm (Figure S93).

Single crystals of **3** were grown from vapor diffusion of hexane into a concentrated benzene solution [43]. The solid‐state structure of **3** (Figure 2b, see Supporting Information X‐ray Crystallographic Section, Table S1) revealed a T‐shaped Ni(0) geometry (∠P–Ni–P = 176.92(4)°). Only a handful of T‐shaped Ni(0) examples are known, but so far, they all bear one tetrylene‐based σ‐acceptor in combination with phosphine and/or *N*‐heterocyclic carbene donors [20, 44, 45, 46]. The geometry around Zn in **3** is distorted trigonal bipyramidal with a τ_5_ value of 0.6 [47, 48]. Of interest, the Ni–Zn bond distance of 2.6145(6) Å may signify a weak bonding interaction as it is slightly longer than the sum of the two metals’ covalent radii at 2.46 Å (Figure 2b) [49].

A quantum theory of atoms in molecules (QTAIM) analysis was conducted on **3** to evaluate whether the Ni and Zn are in a direct bonding interaction [50, 51]. One bond critical point (bcp) was located between Ni and Zn, with significant electron density, ρ(r) = 0.038 e·Bohr^−3^ and a positive Laplacian (∇^2^ρ) of 0.046 e·Bohr^−5^ that indicates a polar Ni–Zn bond (Figure 2c) [52]. A frontier molecular orbital (MO) analysis of **3** essentially shows five doubly‐occupied Ni 3d orbitals, which is consistent with Ni(0) (Figure S109).

### 2.2 Dihydrogen Activation

**SCHEME 1:**
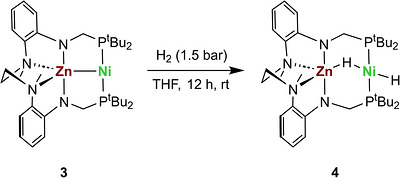
Reaction of Ni–Zn **3** with H_2_.

Addition of 1.5 bar of H_2_ to **3** at room temperature (rt) in THF resulted in a color change from dark red to light orange in 12 h and a shift of the ^31^P signal to 95.3 ppm. Of note, the product **4**, which was isolated in 86% yield, is stable to vacuum at rt and does not revert back to **3** (Scheme 1). The ^1^H NMR spectrum of **4** at rt showed a characteristic triplet at −10.19 ppm (*J*_P−H_ = 40.8 Hz), that integrated to two protons (Figure S43). Such a peak is consistent with the formation of a terminal dihydride species, LZnNi(H)_2_. To verify the presence of dihydride ligands, we carried out *T*_1_ measurements between 193 and 303 K (Figure S96). The *T*_1(min)_ relaxation time of 523 ms at 233 K is fully consistent with dihydride ligands and excludes the possibility of an intact H_2_ ligand (Table S4) [53].

Exposure of **3** to D_2_ in THF solution generated **4**−**D** in 12 h, which was subsequently isolated as an orange solid powder in 90% yield. Characteristic ^2^H resonances for the Ni dideuteride complex were observed at −10 ppm as a triplet (*J*_P−D_ = 6.6 Hz, Figure S50). The ATR‐IR spectrum of **4** showed a prominent absorption at 1642 cm^−1^ that corresponds to the Ni–H stretch, which for **4**–**D** shifts to 1182 cm^−1^ (predicted ν_Ni−D_ = 1171 cm^−1^ using Hooke's law, Figures S85−S88).

**FIGURE 3:**
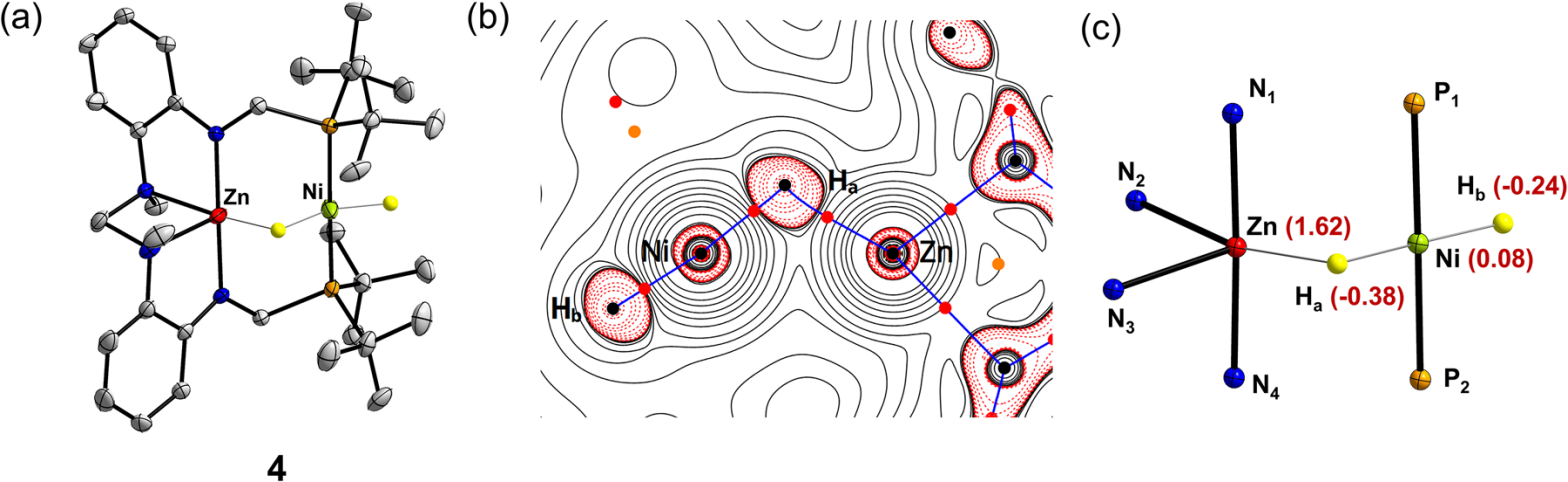
(a) Molecular structure of **4** shown at 50% thermal ellipsoid probability except for H atoms. H atoms of the pincer‐type ligand have been omitted for clarity. (b) QTAIM molecular graphs of **4** showing key bcps (in red) and rcps (in orange). (c) Charges obtained by natural population analysis (NPA) for the first coordination shell of **4**.

A single crystal of **4**, grown from vapor diffusion of hexane into a concentrated THF solution, was investigated by X‐ray diffraction (Table S2). The Ni−Zn distance of 2.6690(6) Å in **4** is elongated by 0.055 Å compared to that in **3** and indicates the absence of any metal‐metal interaction (Figure 3a). In support, QTAIM predicted no bcps between Ni and Zn (Figure 3b). Both hydride atoms were successfully located in the electron difference map, and the refined structure is consistent with LZn(*μ*‐H)Ni(H), where one hydride is terminal at Ni and the second hydride bridges between Ni and Zn. The HNi(*μ*‐H)Zn connectivity was also predicted by DFT (see below), and the natural population analysis (NPA) charges on the H atoms (−0.38, −0.24) further support their hydridic nature (Figure 3c). Moreover, bcps were located along the Ni−H_terminal_ (ρ(r) = 0.13 e·Bohr^−3^), Ni−H_bridging_ (0.11 e·Bohr^−3^), and Zn−H_bridging_ (0.06 e·Bohr^−3^) bonds.

The seeming discrepancy between the solution‐ and solid‐state structures was resolved by variable‐temperature NMR studies (see below), which demonstrate that **4** is fluxional and can interconvert between *trans*‐Ni(H)_2_ and Zn(*μ*‐H)Ni(H). The latter motif had been previously proposed in other Ni−E heterobimetallic systems, [^Mes^DPB^Ph^]Ni and (^Mes^PAlP)Ni, which also react with H_2_ to form dihydride species featuring the HNi(*μ*‐H)E core [15, 16, 54]. However, these literature samples, which are generated in situ under an H_2_ atmosphere, release H_2_ readily under vacuum, and hence, their solid‐state structures have not been reported. Complex **4** is distinct in that it is stable to H_2_ loss, and to the best of our knowledge, this is the first structural characterization of the HNi(*μ*‐H)E core that arises from H_2_ activation. Of note, **4** is a competent hydrogenation catalyst for terminal olefins such as styrene and its *para*‐substituted derivatives, as well as 1‐hexene and 1‐octene (5 mol% loading, 70°C, 2 bar H_2_; Table S7). In comparison to other base‐metal hydrogenation catalysts, both the activity and the substrate scope of **4** are limited [55, 56].

The *trans*‐Ni(H)_2_ structural motif with two terminal hydrides at Ni, which is accessible in solution, has not been previously invoked to the best of our knowledge. A search of the Cambridge Structural Database (CSD) [57] of Ni(H)_2_ yielded no hits for the two terminal hydrides, with the majority of examples bearing two bridging hydrides, Ni(*μ*‐H)_2_Ni, in a Ni_2_H_2_ diamond core and one containing Al(*μ*‐H)_2_Ni [58, 59, 60, 61, 62, 63, 64, 65]. However, two structures are noteworthy for further comparison: a Ni *cis‐*dihydride and a hexagonal Ni trihydride, which are supported by disilylene and Mg(nacnac) ligands, respectively [66, 67]. In the first case, the Ni *cis‐*dihydride bears a “significant bonding interaction” to the silylene ligand (Ni−H—Si), as evidenced by the ^1^ *J*_Si−H_ coupling of 44.2 Hz, which is intermediate between that of a classical Ni silyl hydride (11 Hz) [68, 69] and that of a nonclassical Ni hydrosilane σ‐complex (111 Hz) [66, 70, 71, 72, 73, 74, 75]. In the second case, a more delocalized Ni(*μ*‐H)Mg bonding was invoked, with Mg–H bond lengths of 2.03(3) to 2.04(3) Å, based on a neutron diffraction study. We postulate that the lability of the bridging hydride in **4** is due to the weak Lewis acidity of the Zn(II) metalloligand. In support, the fluoride ion affinity (FIA) for **4** in the gas phase was calculated to be 233 kJ/mol, which is on par with other weak Lewis acids, including Si(OH)_4_ (FIA 223) and PPh_5_ (226) [76].

### 2.3 VT‐NMR and DFT Study of the Stereodynamics of **4**

**FIGURE 4:**
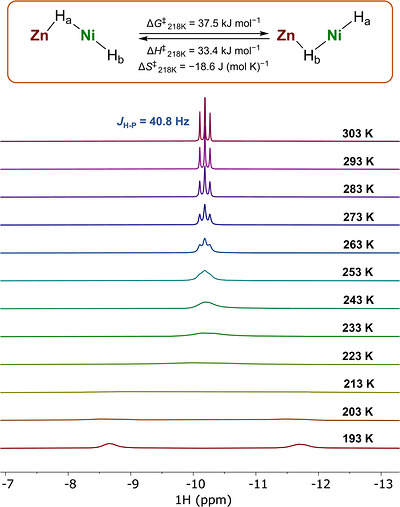
Stacked ^1^H VT‐NMR plot of **4** in THF‐*d*_8_ from 193 to 303 K in the region from −7 to −13 ppm to show the fluxionality of the two hydride resonances. (For additional NMR spectra, see Figures S94, S95).

The stereodynamics of **4** was investigated using variable temperature (VT) ^1^H NMR spectroscopy in the temperature range of 193 to 303 K (Figure 4). At 193 K, two broad peaks were observed at −8.65 and −11.71 ppm, corresponding to the two distinct hydrides. Typically, in the HNi(*μ*‐H)E systems, the more upfield signal corresponds to the terminal hydride [16]. Upon raising the temperature, the two hydride peaks coalesce at 218 K and further sharpen into a triplet at −10.19 ppm above 263 K. Standard activation parameters of the exchange process were determined through Eyring analysis of the temperature‐dependent exchange rate constants (k/Hz), which were obtained by gNMR full lineshape analysis of the hydride signals (Figure S98 and Table S5 and S6) [77].

The standard activation enthalpy (Δ*H* ^‡^_218 K_) and the standard free activation energy (Δ*G* ^‡^_218 K_) for the hydride exchange in **4** are 33.4(1.0) and 37.5(0.6) kJ mol^−1^, respectively. The standard activation entropy for the hydride exchange in **4** is negative and relatively small, Δ*S* ^‡^_218 K_ = −18.6(4.1) J (mol K)^−1^. Hence, the enthalpic contribution to the energy barrier is more significant, which is consistent with a mechanism that involves Zn−H bond cleavage. In support of the small entropic barrier, the ^1^H NMR shifts of the ligand backbone are nearly unchanged from 193 to 303 K (Figure S94).

**FIGURE 5:**
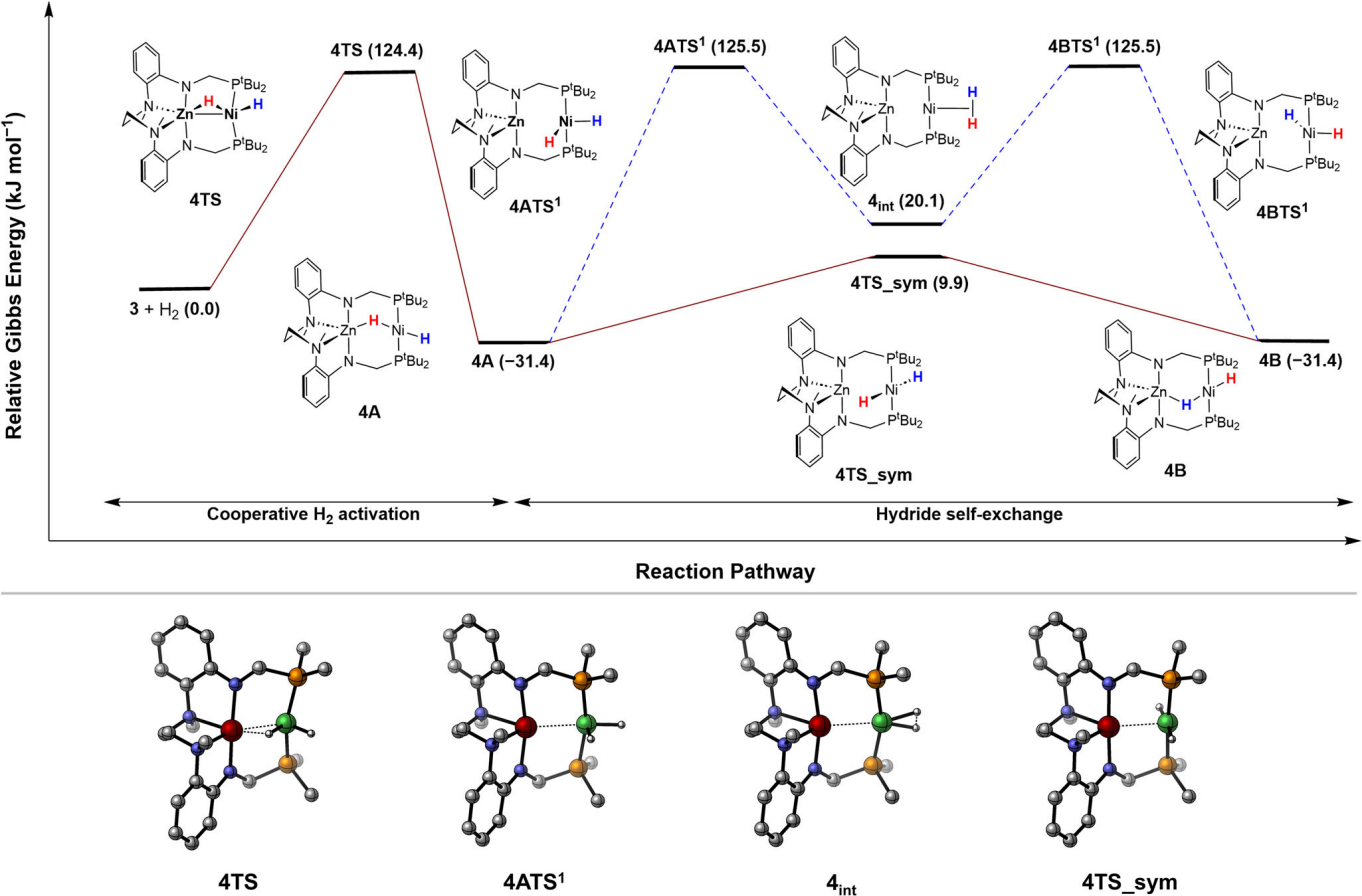
(*top*) DFT‐calculated reaction pathways for cooperative H_2_ activation by **3** and the self‐exchange of hydrides in **4**. Values in the parentheses refer to the computed free energy differences in kJ mol^−1^. (*bottom*) DFT structures of key intermediates and transition states. Ligand H atoms and the methyl groups of the ^t^Bu substituents have been omitted for clarity.

The reaction profile of the H_2_ activation for **3** was investigated by DFT calculations at the r^2^Scan D4/def2‐TZVP level of theory [78, 79]. A transition state (**4TS**) was located at an activation energy barrier of 124.4 kJ mol^−1^ and is consistent with a concerted activation of H_2_ across the Ni−Zn bond (Figure 5). **4TS** is a late transition state, wherein the Ni−H, Ni−(*μ*‐H) and Zn−(*μ*‐H) bond lengths are within 0.1 Å of those found in the product **4**. The H−H distance is significantly lengthened to 1.948 Å versus 0.74 Å in free H_2_, indicating that the H−H bond is completely cleaved [53]. The notable differences in **4TS** compared to **4** are the presence of a Ni−Zn bond (2.48 Å) in **4TS**, and the acute H−Ni−H and P−Ni−P bond angles of 79.5 and 156.0 deg, respectively.

Next, two possible mechanisms were investigated for the self‐exchange of the dihydrides in **4** (from **4A** to **4B**). The first pathway involves H_2_ reductive elimination to form the nonclassical Ni−H_2_ adduct intermediate, **4_int_**, which is endergonic by 51.5 kJ mol^−1^. The transition state (**4ATS^1^**) was located at 156.9 kJ mol^−1^ above **4**, and resembles a *cis*‐dihydride Ni species. As an aside, **4ATS^1^** is energetically similar to **4TS**, making Ni‐mediated H_2_ activation (**4_int_**) also viable. The second pathway is a direct exchange reaction via a *C*_2_ symmetric transition state, **4TS_sym**, with a square‐planar *trans*‐dihydride Ni motif. The low activation energy barrier of 41.3 kJ mol^−1^ makes this the energetically preferred pathway, and involves the cleavage of the Zn−(*μ*‐H) bond and partial rotation about the P−Ni−P axis. Of note, the calculated activation energy barrier closely matches that determined experimentally.

### 2.4 Reaction of Ni−Zn Bimetallic With CO

**FIGURE 6:**
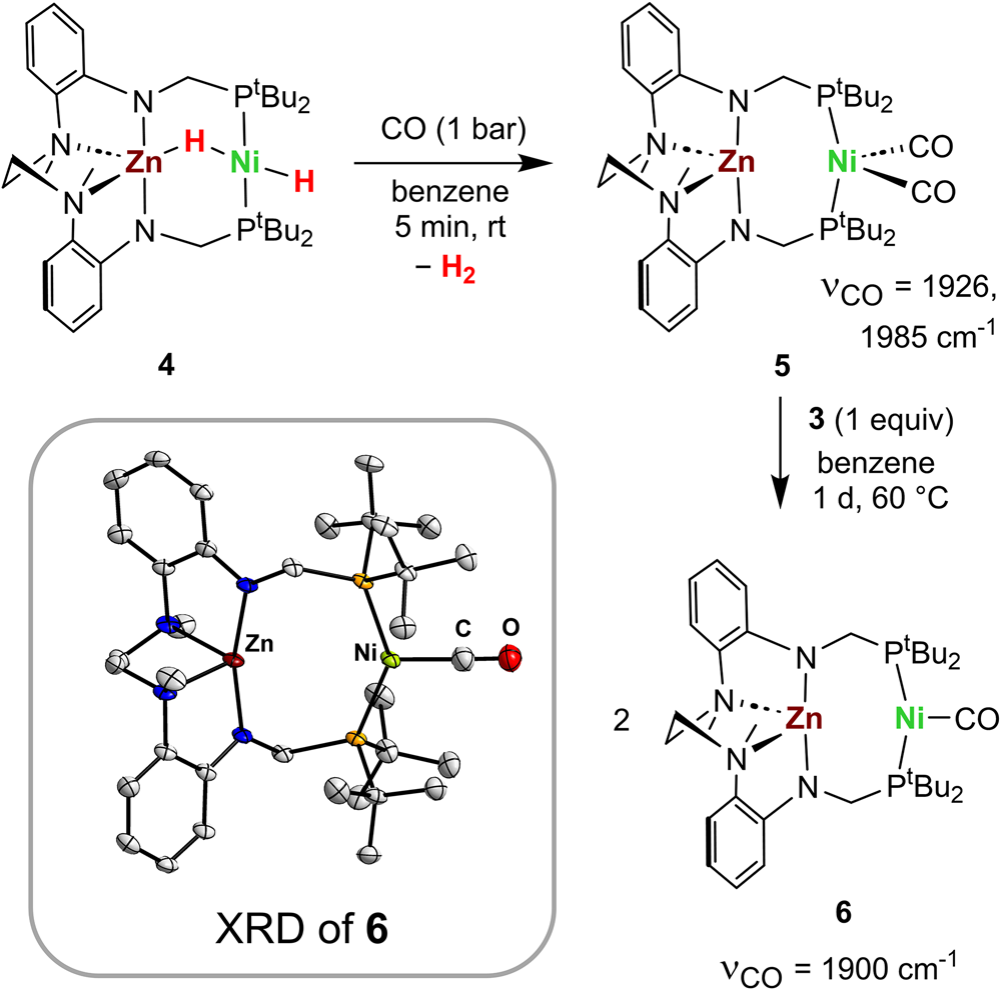
Formation of Ni–Zn bis‐ and monocarbonyl complexes (**5** and **6**, respectively) via H_2_ reductive elimination from **4**. (*inset*) Molecular structure of **6** shown at 50% thermal ellipsoid probability. H atoms have been omitted for clarity.

Since **4** does not release H_2_ spontaneously to give **3**, we investigated the reaction of **4** with CO to force the reductive elimination of H_2_. Gratifyingly, the addition of 1 bar of CO to **4** in benzene resulted in the disappearance of the ^1^H hydride resonances with a concomitant color change from light orange to light yellow within 5 min, while the ^31^P peak shifted to 68.5 ppm. The ATR‐IR spectra of the newly formed complex **5** show two CO bands at 1926 cm^−1^ and 1985 cm^−1^, which we assign to a Ni bis(carbonyl) species (Figures 6 and S89).

The mono carbonyl complex **6** (ν_CO_ = 1900 cm^−1^) was accessed in quantitative yield by reacting **5** with one equiv of **3**, resulting in a rare release of a CO ligand from a Ni(0) center (Figures 6 and S90) [80]. Alternatively, **6** can be independently prepared by careful addition of one equiv of CO to either **3** or **4**. Single‐crystal X‐ray diffraction analysis confirmed the structure of **6** with a distorted trigonal planar Ni and a long Ni∙∙∙Zn distance of 3.80 Å, indicating no interaction (Tables S2, S3).

### 2.5 Reactivity of the Dihydride Complex **4** With CO_2_

**FIGURE 7:**
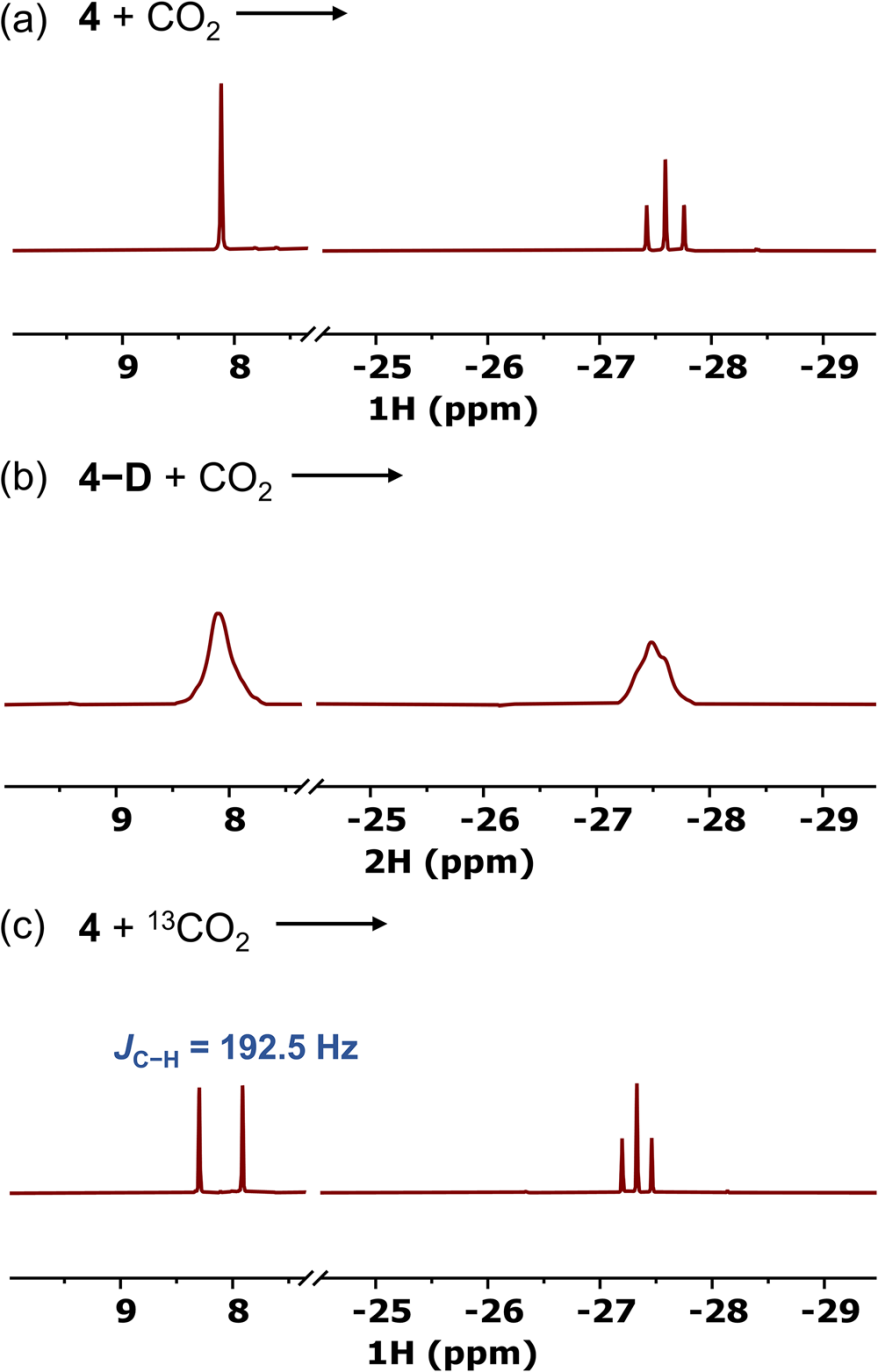
Partial ^1^H/^2^H NMR spectra of reactions between **4** or **4−D** and ^12^CO_2_ or ^13^CO_2_ (1 bar) at t ∼5 min. (a) ^1^H spectrum of **4** and CO_2_, (b) ^2^H spectrum of **4−D** and CO_2_, and (c) ^1^H spectrum of **4** and ^13^CO_2_. For full NMR spectra, see Figures S68, S76, and S78.

Addition of 1 bar of CO_2_ to a THF‐*d*_8_ solution of **4** at rt resulted in an immediate color change from orange to light yellow with a new ^31^P{^1^H} peak at 54.7 ppm. In addition to the ligand signals, two new ^1^H peaks were observed at 8.1 (singlet) and −27.6 ppm (triplet, ^2^ *J*_P‐H_ = 67 Hz), which would be consistent with a formate H and a terminal Ni−H, respectively (Figure 7a). Further support for this new product **7** was evidenced through isotope‐labeling studies. Exposure of **4−D** to CO_2_ led to the expected ^2^H signals for both the formate D and the Ni–D resonances (Figure 7b). Reacting **4** with ^13^CO_2_ resulted in a splitting of the formate H resonance into a doublet (^1^ *J*_C−H_ = 192.5 Hz, Figure 7c).

The new product **7** exhibits two carboxylate bands in the IR spectrum in C_6_D_6_. The antisymmetric (*ν*_a_(COO)) mode at 1625 cm^−1^ shifts to 1585 cm^−1^ for the ^13^C‐labelled analogue, **7^13C^** (Figures S91, S92). The less intense symmetric (*ν*_s_(COO)) mode was identified in the difference IR spectrum and appears at 1313 cm^−1^ for **7**, and 1290 cm^−1^ for **7^13C^**. For metal carboxylate complexes, the energy difference of these two stretches (Δ = *ν*_a −_ *ν*_s_) informs on the nature of the binding of the carboxylate [81]. The Δ of 312 cm^−1^ is most consistent with a κ^1^‐monodentate formate (228−470 cm^−1^) and falls outside the expected values for bridging (138−190 cm^−1^) and κ^2^‐bidentate (42−190 cm^−1^) binding modes. Two stable isomers of **7** were predicted by DFT, **7a** and **7b**, of which the latter is more stable by a mere 2.7 kJ mol^−1^ (Figure 9). While **7a** is a Ni(*μ*‐H)Zn with a monodentate *κ* ^1^ *O*‐formate bound to Ni, **7b** is a bridging Ni(*μ*‐OCHO‐*κ* ^1^ *O,κ* ^1^ *O'*)Zn with a terminal Ni hydride. The DFT‐calculated Δ values are 365 and 260 cm^−1^, respectively. The observed Δ is essentially the same as their average value, suggesting that **7a** and **7b** may coexist in rapid equilibrium. Of note, the Δ for **7b** is higher than expected for bridging carboxylate ligands. However, this discrepancy can be attributed to the asymmetric bridging mode in the predicted structure, with a longer Zn–O distance of 2.20 Å and a shorter Ni−O distance of 1.93 Å.

**FIGURE 8:**
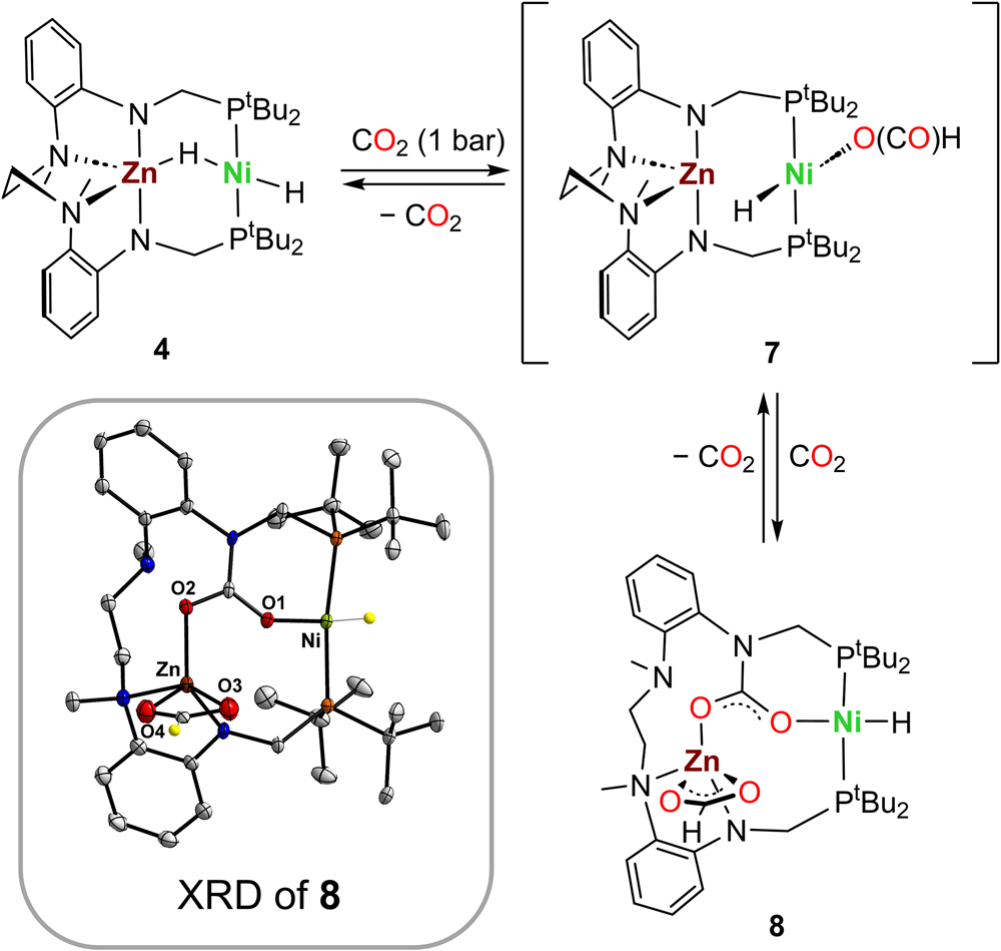
Reversible reactivity between **4** and CO_2_ (1 bar) to yield **7** (*t* ∼ 5 min) and **8** (*t* ∼ 1 day). (*inset*) Molecular structure of **8** shown at 50% thermal ellipsoid probability. H atoms (except Ni−H and formyl H) and lattice solvent molecules have been omitted for clarity.

Complex **7** is unstable to vacuum and reverts slowly to **4** under an N_2_ atmosphere (Figure 8). Due to the reversibility of CO_2_ insertion, clean cycling between **4** and **7** was effected through repeated exposures to vacuum and CO_2_ (Figures S100, S101). Complex **7** reacts further with CO_2_ (t_½_ ∼ 12 h under 1 bar CO_2_) to form a new product **8**, which was crystallized from a concentrated benzene solution under CO_2_ (Figure 8). The molecular structure revealed a second CO_2_ insertion into the Zn–N_amide_ bond, forming a new bridging carbamate ligand between Zn and Ni. Simultaneously, the formate ligand has migrated fully to Zn as a bidentate ligand. This is reminiscent of the carboxylic shift mechanism observed in metalloenzymes, where a carboxylate ligand can switch from binding one metal ion to two in bridging fashion, and vice versa [82].

**FIGURE 9:**
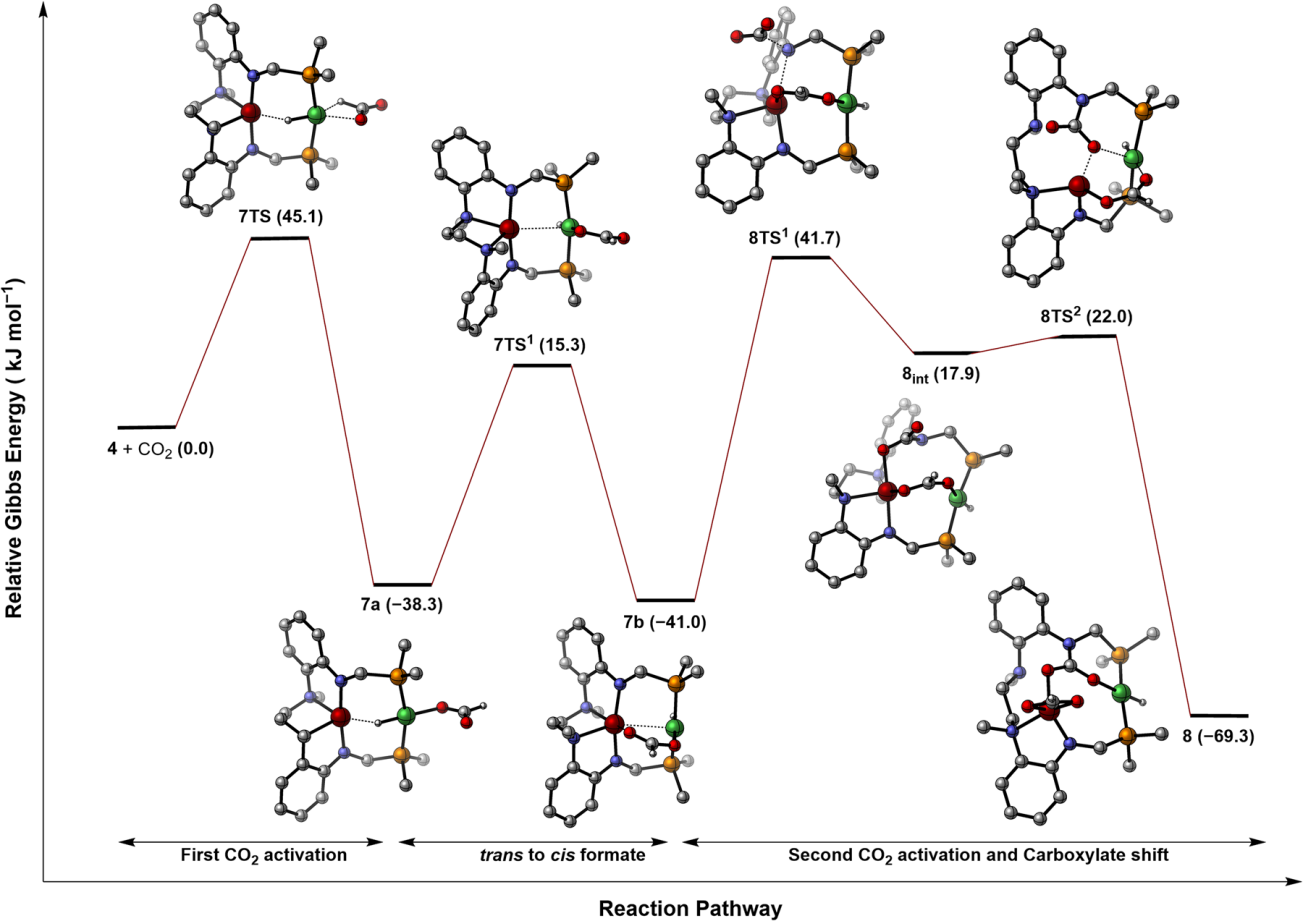
DFT‐calculated reaction pathways for the insertion of CO_2_ into **4**. Ligand H atoms and the methyl groups of the ^t^Bu substituents have been omitted for clarity. Values in the parentheses refer to the computed free energy differences in kJ mol^−1^.

The DFT‐calculated mechanism of double CO_2_ insertion into **4** to generate **8** is also shown in Figure 9. The first CO_2_ insertion into the Ni−H bond in **4** proceeds via a low activation energy barrier of 45.1 kJ mol^−1^, via a 4‐centered late‐stage transition‐state, with the following bond distances in the 4‐membered ring: Ni–H, 1.77; C–O, 1.26; Ni–O, 2.20; and C–H, 1.20 Å, to give **7a**. The exchange process between the two isomers **7a** and **7b** is essentially a toggling of the bridging ligand from H to formate. This proceeds through **7TS^1^**, where neither H nor formate is bridging, with an energy barrier of 53.6 kJ mol^−1^.

The second molecule of CO_2_, which formally inserts into the Zn−N_amide_ bond, undergoes nucleophilic attack by the amide upon its approach. A high‐energy transition state **8TS^1^** (Δ*G^‡^* = 82.7 kJ mol^−1^) was located, where the Zn−N_amide_ bond has elongated to 2.52 Å with partial formation of the N_amide_—CO_2_ bond at 1.65 Å. We postulate that the elongation of the Zn−N_amide_ bond is necessary to create a sufficiently nucleophilic N to attack CO_2_. In support, the other Zn−N_amide_ bond remains short at 2.05 Å. Moreover, the bridging formate ligand forms a stronger interaction with Zn at 2.01 Å, which likely compensates for the weakening of one Zn−N_amide_ interaction. This is an interesting case of the carboxylate shift mechanism. A classical carboxylate shift mechanism in methane monooxygenases involves a change from bridging mode to a single‐metal binding, so as to open a coordination site for oxygen binding at one of the iron centers [82, 83, 84, 85]. In the present case, the carboxylate shift from single‐metal to bridging weakens the coordination of an existing metal‐ligand bond, that is, Zn−N_amide_, so as to enable further reactivity with a substrate, that is, nucleophilic attack of the amide on an external CO_2_.

The immediate product **8_int_** thus features a new carbamate motif that is *κ* ^1^ *O*‐bound to Zn. Next, **8_int_** goes through a very low energy transition state **8TS^2^** (Δ*G^‡^* = 4.1 kJ mol^−1^) to give the final product **8**. **8TS^2^** is interesting as one of the oxygen atoms of the carbamate bridges between the two metal centers and completes a 6‐membered cyclic transition state along with the bridging formate ligand. From **8_int_** to **8**, the carbamate ligand changes coordination modes from *κ* ^1^ *O* to Zn, to *κ* ^1^ *O*‐bridging between Zn and Ni, and finally to a bridging *κ* ^1^ *O,κ* ^1^ *O'*‐carbamate. This is accompanied by a reverse formate shift from bridging to bidentate only to Zn, as well as a cleavage of a Zn−N_amine_ bond.

Next, we briefly investigated the potential for the Ni−Zn system to catalyze CO_2_ reduction. As the release of formic acid from **7** was significantly uphill by +104.9 kJ/mol (Figure S115), different bases were added to drive formate release. Complex **4** was heated in the presence of 4 equiv base under 1 bar of H_2_:CO_2_. While partial formate release was detected (Figure S103), no catalytic turnover was observed. Replacing H_2_ with HBPin did generate CO_2_‐reduced products, HCO_2_BPin and CO; but the HCO_2_BPin turnovers were quite limited at ∼23, and CO deactivated the catalyst as the monocarbonyl **6** [86]. While catalytic CO_2_ hydrogenation was not achieved, the Ni−Zn system has accomplished the first step of CO_2_ hydrogenation to formate without needing additives or stoichiometric bases.

## 3 Conclusion

In summary, a T‐shaped Ni(0) complex supported by a Lewis acidic Zn(II) center (**3**) reacted with H_2_ to form a stable Ni dihydride species (**4**), which is rare. In the solid state, the Zn(*μ*‐H)Ni(H) motif was observed, but in solution at rt, the two hydrides are equivalent in the ^1^H NMR spectrum, consistent with a *trans‐*Ni(H)_2_ motif. The ability to toggle easily between these two motifs is attributed to the weak Lewis acidity of the Zn(II) ion in **4**, which is supported by QTAIM calculations, where the Zn−(*μ*‐H) bond critical point has a lower electron density (0.06 e·Bohr^−3^) compared to the Ni−(*μ*‐H) one (0.11 e·Bohr^−3^). Moreover, the barrier between the Zn(*μ*‐H)Ni(H) and *trans‐*Ni(H)_2_ motifs is predicted by DFT to be only 41.3 kJ mol^−1^.

The Ni hydrido formate complex (**7**) is, to the best of our knowledge, the first Ni example that forms from the simple addition of H_2_ and CO_2_ without an external base. This insertion of CO_2_ into an H_2_‐derived Ni dihydride is a desirable transformation because it could represent a key step in Ni‐mediated CO_2_ hydrogenation that only uses H_2_ and CO_2_. However, additional strategic designs are needed to enable formic acid release in this system to allow for efficient catalysis. Finally, **7** reacted with another CO_2_ molecule to give the dual CO_2_ activation product, **8**, which features a bridging *κ* ^1^ *O,κ* ^1^ *O'*‐carbamate ligand between Ni and Zn. The DFT‐predicted mechanism reveals the importance of a carboxylate shift mechanism to create a sufficiently nucleophilic Zn−N_amide_ that directly inserts CO_2_ to generate a carbamate group.

In all these transformations, the Zn metalloligand plays a key role. Compared to other Ni‐Lewis acid pairings in the literature, the weaker Lewis acidity of Zn(II) enables lability of the bridging ligands in Ni(*μ*‐L)Zn, where L is hydride in **4** or formate in **7**. This fluxionality allows for a rocking rotation about the P−Ni−P vector that toggles between bridging and terminal binding modes. The fluxional binding of the formate ligand to Zn(II) is also key to the reaction of **7** and CO_2_ to give **8**. A combined study with X‐ray structures and IR analysis supported by DFT calculations showed three different binding modes of formate: *κ* ^1^ *O* on Ni, bridging *κ* ^1^ *O,κ* ^1^ *O'* between Ni and Zn, and terminal κ^2^ *O,O'* on Zn. As a whole, this work shows a new variation of metal‐metal cooperativity to promote Ni‐mediated H_2_/CO_2_ activation, which can inform the design of future Ni‐based catalysts for atom‐efficient CO_2_ hydrogenation.

## Author Contributions

**Krishnendu Dey**: writing – original draft, writing – review and editing, conceptualization, investigation, visualization. **Matthias Borowski**: investigation. **Gregor Schnakenburg**: writing – review and editing, supervision, formal analysis, visualization, data curation. **Connie C. Lu**: writing – original draft, writing – review and editing, supervision, conceptualization, project administration, resources, data curation.

## Conflicts of Interest

The authors declare no conflicts of interest.

## Supporting information



The authors have cited additional references within the Supporting Information.
**Supporting File 1**: anie73052‐sup‐0001‐SuppMat.pdf.

**Supporting File 2**: anie73052‐sup‐0002‐Data.zip.

## Data Availability

The data that supports the findings of this study are available in the supplementary material of this article.
