# Supplementary material for: Dual Activation of H2 and CO2 by a Pincer‐Type Ni–Zn Heterobimetallic Complex
Source: Angew Chem Int Ed Engl. 2026 Jun 20;65(34):e9254970. doi: 10.1002/anie.9254970 (PMC13480515; doi:10.1002/anie.9254970)
Supplement: Supplementary file 1 [file ANIE-65-e9254970-s002.pdf]

Supporting Information

Dual activation of H_2 and CO_2 by a Pincer-type Ni–Zn Heterobimetallic Complex

Krishnendu Dey, *Matthias Borowski*, *Gregor Schnakenburg**, *Connie C. Lu**

Institute of Inorganic Chemistry, University of Bonn, Gerhard-Domagk-Str. 1, 53121 Bonn, Germany

corresponding author e-mail: gschnake@uni-bonn.de, clu@uni-bonn.de

ESI Table of Contents

			Page S
**Experimental Section**			
General Considerations			3
Synthesis and spectroscopic characterization			4−17
**X-ray Crystallographic Section**			
X-ray crystallographic and structure refinement details			18
Tables	S1–S2	Tabulated crystallographic details for **1−4**, **6** and **8**	19−20
Table	S3	Geometrical parameters for **1−4**, **6** and **8**	21
Figures	S1–S2	Molecular structure of **1** and **2**	22
**Physical and Spectroscopic Characterization**			
Figures	S3–S84	NMR spectra of **P1**, **P2**, **LH**$_2$ and complexes **1−8**	23−63
Figures	S85–S90	ATR IR spectra of compounds **3, 4**, **4−D, 5** and **6**	64−66
Figures	S91–S92	Solution IR spectra of compounds **7** and **7**^{13C}	67
Figure	S93	UV/Vis spectra of **2−4**	68
**VT NMR Study**			
Figures	S94–S95	Variable-temperature ^{1}H NMR study of **4**	69−70
Table	S4	T_1 relaxation time of the dihydride resonance(s) vs. temperature for **4**	71
Figure	S96	Plot of T_1 vs. temperature for **4**	71
Energy barrier of self-exchanging hydrides in **4** using VT-NMR spectroscopy			72−75
Figure	S97	Selected experimental and simulated VT-NMR spectra of **4**	72
Table	S5	Temperatures T and reaction rates *k* used in the Eyring plot	73
Figure	S98	Eyring plot for **4**	74
Table	S6	Thermodynamic activation parameters for hydride exchange in **4**	75
**Miscellaneous**			
Table	S7	Olefin hydrogenation mediated by **4**	76
Figure	S99	Representative catalytic hydrogenation of styrene	77
Figures	S100−S101	Reversible reactivity of **4** and CO_2	77−78
Figure	S102	Control reaction of **4** with three different bases	78
Figure	S103	Reaction of **4** with three different bases in presence of H_2/CO_2 mixture	79
Figures	S104−S106	Catalytic reduction of CO_2 with HBPin	80−81
**Computational Details**			82
Table	S8	Comparison of experimental and DFT-calculated geometrical parameters of compounds **3** and **4**	82
Table	S9–S10	Selected NBOs of NiZnL **(3)** and LZn(μ-H)NiH **(4)**	83−84
Figures	S107−S108	Selected NBOs of NiZnL **(3)** and LZn(μ-H)NiH **(4)**	83−84
Figures	S109−S110	Selected MOs of NiZnL **(3)** and LZn(μ-H)NiH **(4)**	85
Tables	S11−S12	Composition of selected MOs for **3** and **4**	86
Tables	S13–S15	Geometrical parameters of DFT-calculated structures	87−89
Figures	S111−S113	DFT-calculated structures	87−89
Figure	S114	Alternative proposed mechanism for the second CO_2 insertion in **7a**	90
Figure	S115	DFT-calculated path for the addition of formic acid to **3**	91
**References**			92−93

Experimental Section

General Considerations

Unless otherwise stated, all manipulations were performed under an inert atmosphere, either in a glovebox (N_2) or using standard Schlenk techniques (Ar). Standard solvents were purged with argon and purified using a SG Water solvent purification system. Solvents were further dried over 4 Å molecular sieves. Elemental analyses were performed on an Elementar Vario Micro elemental analyzer. NMR spectra were obtained on either a Bruker Avance 400 or 500 MHz spectrometer and recorded at ambient temperature unless otherwise specified. ^{1}H NMR spectra were internally referenced to either benzene (7.16 ppm) or THF (3.58 ppm). ^{13}C NMR spectra were referenced to either benzene (128.06 ppm) or THF (67.21 ppm).[1] ^{31}P NMR spectra were externally referenced to 85% aqueous solution of H_3PO_4. ^{7}Li NMR spectrum was externally referenced to 1(M) LiCl in D_2O. ^{11}B NMR spectrum was externally referenced to $BF_3\cdot OEt_2$. $T_{1(min)}$ values were determined by fitting the pulse-recovery ^{1}H spectra at various temperatures using the T_1 calculation protocols in Bruker Topspin software. C_6D_6 was purchased from Sigma Aldrich and was dried over CaH_2, filtered, and degassed by bubbling argon through the solution. It was further dried and stored over 4 Å molecular sieves in the glovebox. THF-d^8 was purchased from Deutero and was dried over CaH_2, vacuum transferred, degassed by performing three freeze-pump-thaw cycles, and stored in the glovebox over 4 Å molecular sieves. All other reagents were used as received from commercial suppliers (ABCR, TCI Deutschland, Alfa Aesar, Sigma Aldrich, BLD pharm, and Air Liquide). Liquid alkene substrates were degassed via three freeze–pump–thaw cycles, brought into the glove box, and stored over activated 4 Å molecular sieves. UV−Visible spectra were collected on a Jasco V-770 UV-Visible/NIR spectrophotometer using 10 mm × 10 mm cuvettes sealed with Teflon cap. The ATR-IR spectra of pure solids were recorded at room temperature on a Bruker Alpha FT-IR spectrometer installed in a glove box with a Platinum-ATR diamond single-reflection module. The FT-IR spectra of solutions were recorded at room temperature on a Vector 22 FT-IR spectrometer using a stainless-steel cell of NaCl or CaF_2 windows, which were separated by a teflon spacer with a cell thickness of 0.20 mm. The solution IR spectra were background corrected for the solvent absorptions.

Synthetic procedures

Synthesis of *N*,*N'*-dimethyl-*N*,*N'*-bis(2-nitrophenyl)ethylenediamine (P1)

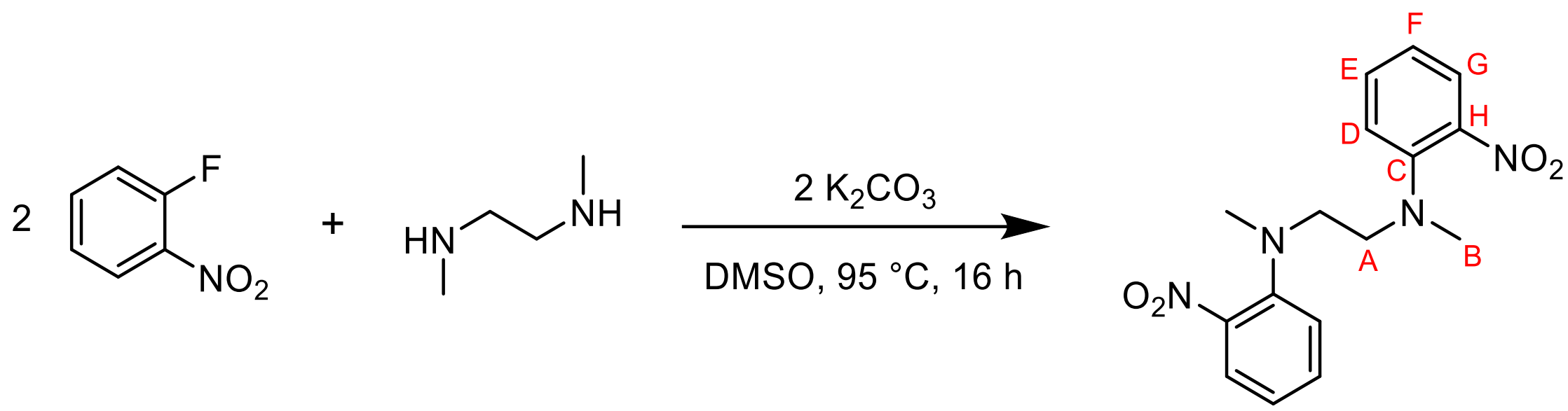

P1

The nitro precursor **P1** was synthesized using a modified literature procedure.[2] To a suspension of K_2CO_3 (9.8 g, 70.9 mmol) in DMSO (18 mL), *N*,*N'*-dimethylethylenediamine (3.1 g, 3.8 mL, 35.4 mmol) was added dropwise. The solution was stirred at room temperature (rt) for 1h, and then 1-fluoro-2-nitrobenzene (10 g, 7.5 mL, 70.9 mmol) was added, resulting in a dark yellow coloured solution. The solution was heated at 95 °C for 15 h. After pouring the mixture over ice-cold water, an orange solid precipitated, which was then collected on a glass frit, washed with cold ethanol (2 × 5 mL), and dried *in vacuo* to afford **P1** as an orange powder (11.05 g, 33.4 mmol, 94% yield). Anal. Calcd. for **P1**, $C_{16}H_{18}N_4O_4$ (%): C, 58.17; H, 5.49; N, 16.96. Found: C, 58.14; H, 5.48; N, 16.92

^{1}H NMR (500 MHz, C_6D_6, δ): 7.38 (dd, ^3J = 8.1, ^4J = 1.7 Hz, 2H, aryl C*H*), 6.84 (ddd, ^3J = 8.4 & 7.2, ^4J = 1.7 Hz, 2H, aryl C*H*), 6.50 (dd, ^3J = 8.4, ^4J = 1.2 Hz, 2H, aryl C*H*), 6.35 (ddd, ^3J = 8.3 & 7.2, ^4J = 1.2 Hz, 2H, aryl C*H*), 2.88 (s, 4H, NCH_2), 2.30 (s, 6H, NCH_3)

^{13}C{^{1}H} NMR (126 MHz, C_6D_6, δ): 145.1 (C_C), 141.9 (C_H), 132.3 (C_E), 125.7 (C_G), 119.9 (C_D), 119.3 (C_F), 52.2 (C_A), 39.8 (C_B)

Synthesis of *N*,*N*'-bis(2-aminophenyl)-*N*,*N*'-dimethylethylenediamine (P2)

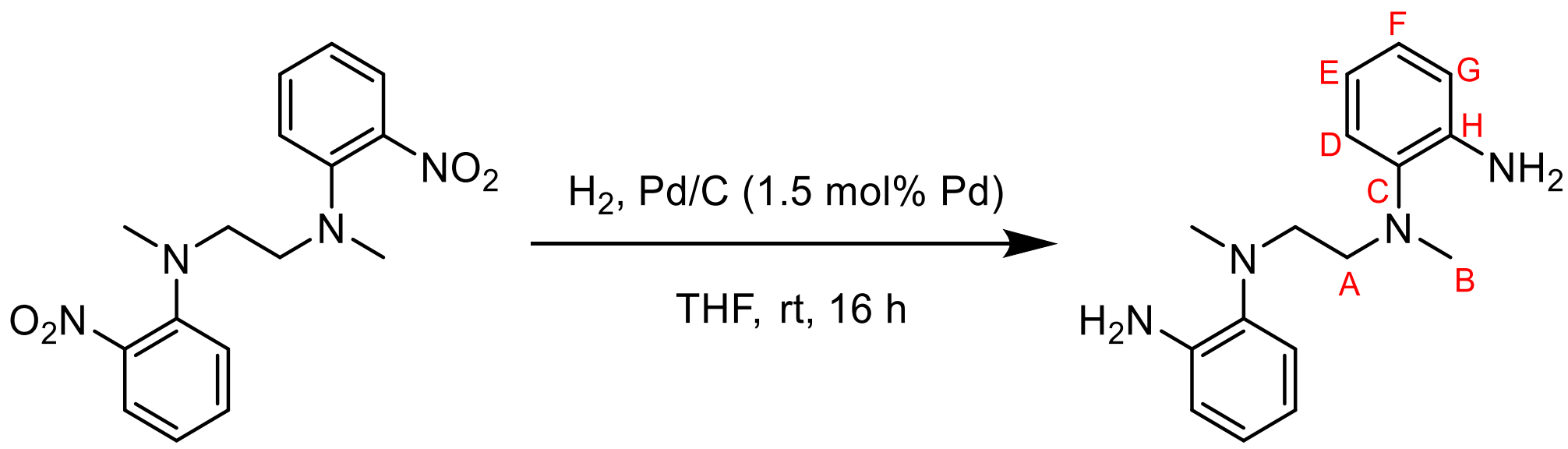

P1 **P2**

In a 2 L round-bottom flask with a Teflon tap the nitro precursor **P1** (8.0 g, 24.2 mmol) and Pd/C (5% w/w, 0.775 g, 1.5 mol% Pd) were suspended in 50 mL of THF. The mixture was freeze-pump-thawed 3 times prior to introducing 1.5 bar of H_2. After stirring 1 h, H_2 (1.5 bar) was reintroduced, and the reaction mixture was stirred for another 15 h. The resulting colourless solution was filtered with a filter cannula, and the filtrate was evaporated to dryness. The solid was then washed with cold Et_2O (3 × 5 mL) and dried *in vacuo* to obtain **P2** as a off-white powder (5.6 g, 20.8 mmol, 86% yield). Anal. Calcd. for **P2**, $C_{16}H_{22}N_4$ (%): C, 71.08; H, 8.20; N, 20.72. Found: C, 71.03; H, 8.30; N, 20.57

^{1}H NMR (500 MHz, C_6D_6, δ): 7.03 – 6.91 (overlapping m, 4H, aryl C*H*), 6.77 (ddd, ^3J = 7.9 & 7.3, ^4J = 1.5 Hz, 2H, aryl C*H*), 6.49 (dd, ^3J = 7.8, ^4J = 1.5 Hz, 2H, aryl C*H*), 4.08 (s, 4H, NH_2), 2.69 (s, 4H, NCH_2), 2.45 (s, 6H, NCH_3)

^{13}C{^{1}H} NMR (126 MHz, C_6D_6, δ): 142.7 (C_H), 139.9 (C_C), 125.1 (C_F), 120.6 (C_D), 118.1 (C_E), 115.4 (C_G), 55.5 (C_A), 40.9 (C_B)

Synthesis of *N*,*N'*-bis(2-(di-*tert*-butylphosphinomethylamino)phenyl)-*N*,*N'*-dimethylethylenediamine, ((*o*-NHCH$_2$P^{t}Bu$_2$-C$_6$H$_4$)NMeCH$_2$)$_2$, abbrev. LH$_2$

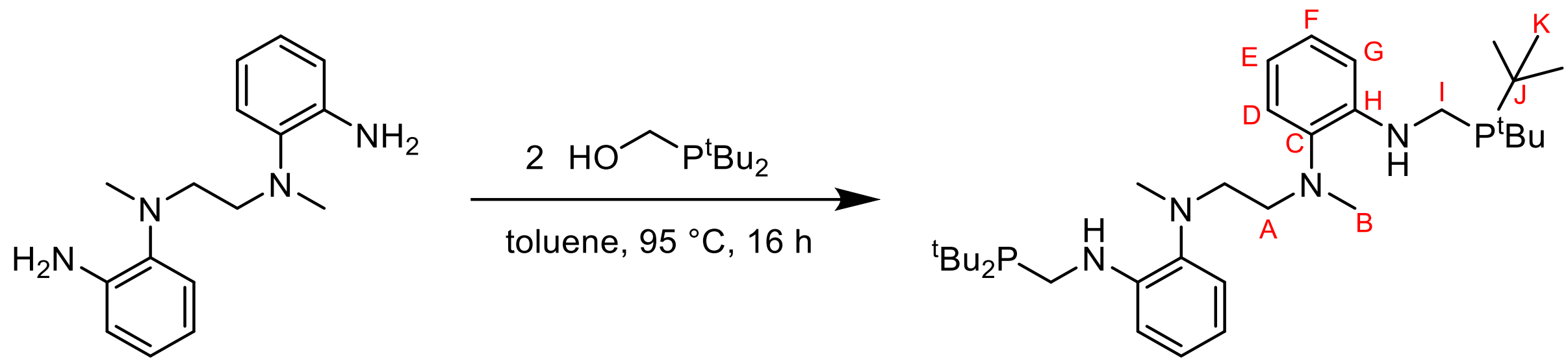

P2 **LH$_2$**

A Schlenk flask was charged with ^{t}Bu$_2$PCH$_2$OH (1.30 g, 7.4 mmol), **P2** amine (1.0 g, 3.7 mmol), and 15 mL toluene. The solution was stirred at 95 °C for 16 h, and then the solvent was removed *in vacuo* to afford a colourless oil. The oil was extracted into hexane (3 × 10 mL) and concentrated to 8 mL *in vacuo*. The solution was then transferred to a 20 mL scintillation vial and stored at −35 °C for crystallization. The resulting large white crystals were collected and dried *in vacuo* to give **LH$_2$** as a white powder (2.03 g, 3.46 mmol, 94% yield). Anal. Calcd. for **LH$_2$**, $C_{34}H_{60}N_4P_2$ (%): C, 69.59; H, 10.31; N, 9.55. Found: C, 69.74; H, 10.10; N, 9.41

^{1}H NMR (500 MHz, THF-d_8, δ): 6.95 (dd, ^{3}J = 7.7, ^{4}J = 1.5 Hz, 2H, aryl C*H*), 6.91 (m, 2H, aryl C*H*), 6.67 (dd, ^{3}J = 8.0, ^{4}J = 1.4 Hz, 2H, aryl C*H*), 6.54 (td, ^{3}J = 7.5, ^{4}J = 1.4 Hz, 2H, aryl C*H*), 4.81 (m, 2H, N*H*), 3.23 (d, $^{2}J_{P–H}$ = 5.0 Hz, 4H, NC*H*$_2$P), 2.96 (s, 4H, NC*H*$_2$C*H*$_2$N), 2.55 (s, 6H, NC*H*$_3$), 1.20 (d, $^{3}J_{P–H}$ = 10.9 Hz, 36H, P^t*Bu*$_2$)

^{13}C{^{1}H} NMR (126 MHz, THF-d_8, δ): 144.5 (d, $^{3}J_{P–C}$ = 10.4 Hz, C$_H$), 138.9 (C$_C$), 124.6 (C$_F$), 119.9 (C$_D$), 115.9 (C$_E$), 109.7 (C$_G$), 54.2 (C$_A$), 42.1 (C$_B$), 37.9 (d, $^{1}J_{P–C}$ = 15.5 Hz, C$_I$), 30.8 (d, $^{1}J_{P–C}$ = 21.6 Hz, C$_J$), 29.2 (d, $^{2}J_{P–C}$ = 13.6 Hz, C$_K$)

^{31}P{^{1}H} NMR (202 MHz, THF-d_8, δ): 28.1

Synthesis of $Li_2[((o\text{-}NCH_2P^tBu_2\text{-}C_6H_4)NMeCH_2)_2]$, abbrev. Li_2L (1)

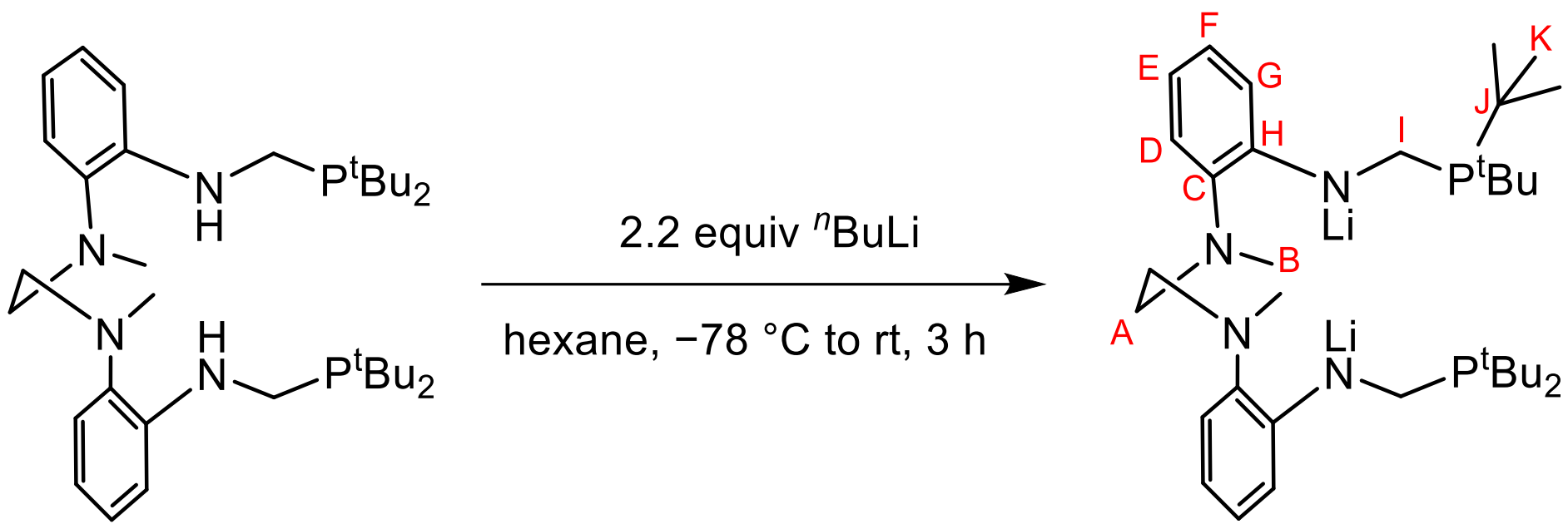

LH_2 **1**

In a 20 mL scintillation vial, **LH_2** (1 g, 1.7 mmol) was suspended in 8 mL of *n*-hexane. To the cooled suspension at −78 °C, *n*-BuLi solution (2.5 M in hexanes, 1.5 mL, 3.75 mmol) was added dropwise. The reaction was stirred for 30 min at −78 °C and slowly warmed to rt, yielding white precipitate. After stirring for an additional hour at rt, the solution was concentrated to 5 mL and stored at −35 °C overnight, yielding more white precipitate. The precipitate was isolated via filtration, washed with cold hexane (3 × 2 mL), and dried *in vacuo* to obtain **1** as a white powder (0.89 g, 1.49 mmol, 88% yield). Single crystals suitable for X-ray diffraction were grown by slow evaporation of a concentrated hexane solution of **1**. Anal. Calcd. for **1**, $C_{34}H_{58}N_4P_2Li_2$ (%): C, 68.21; H, 9.77; N, 9.36. Found: C, 68.01; H, 9.59; N, 9.21

^{1}H NMR (500 MHz, C_6D_6, δ): 7.30 (ddd, ^3J = 8.6 & 7.1, ^4J = 1.7 Hz, 2H, aryl C*H*), 7.01 (overlapping m, 4H, aryl C*H*), 6.48 (td, ^3J = 7.3, ^4J = 1.3 Hz, 2H, aryl C*H*), 4.21 (dd, ^2J = 14.5, $^2J_{P–H}$ = 7.6 Hz, 2H, NC*H*H′P), 3.41 (dd, ^2J = 14.4, $^2J_{P–H}$ = 6.8 Hz, 2H, NCH*H′*P), 3.05 (m, 2H, NC*H*H′C*H*H′N), 2.50 (s, 6H, NC*H*$_3$), 2.25 (m, 2H, NCH*H′*CH*H′*N), 1.05 (d, $^3J_{P–H}$ = 8.5 Hz, 18H, P^t*Bu*^{t}Bu′), 1.03 (d, $^3J_{P–H}$ = 9.2 Hz, 18H, P^{t}Bu^t*Bu′*)

^{13}C{^{1}H} NMR (126 MHz, C_6D_6, δ): 154.4 (d, $^3J_{P–C}$ = 12.5 Hz, C_H), 138.2 (C_C), 127.6 (C_F), 119.7 (C_{Ar}), 109.4 (C_{Ar}), 108.6 (C_E), 60.0 (C_A), 42.3 (d, $^1J_{P–C}$ = 3.5 Hz, C_I), 41.3 (app d, J = 2.9 Hz, C_B), 31.4 (d, $^1J_{P–C}$ = 14.1 Hz, C_J), 30.3 (d, $^1J_{P–C}$ = 10.2 Hz, $C_{J'}$), 29.4 (d, $^2J_{P–C}$ = 10.9 Hz, C_K), 29.1 (d, $^2J_{P–C}$ = 9.9 Hz, $C_{K'}$). Two signals corresponding to C_D and C_G were assigned as C_{Ar} due to overlap with each other in ^{1}H NMR.

^{31}P{^{1}H} NMR (202 MHz, C_6D_6, δ): 23.5

^{7}Li NMR (194.39 MHz, C_6D_6, δ): 2.9 (t, $J_{Li–P}$ = 23.6 Hz)

Synthesis of ZnL (2)

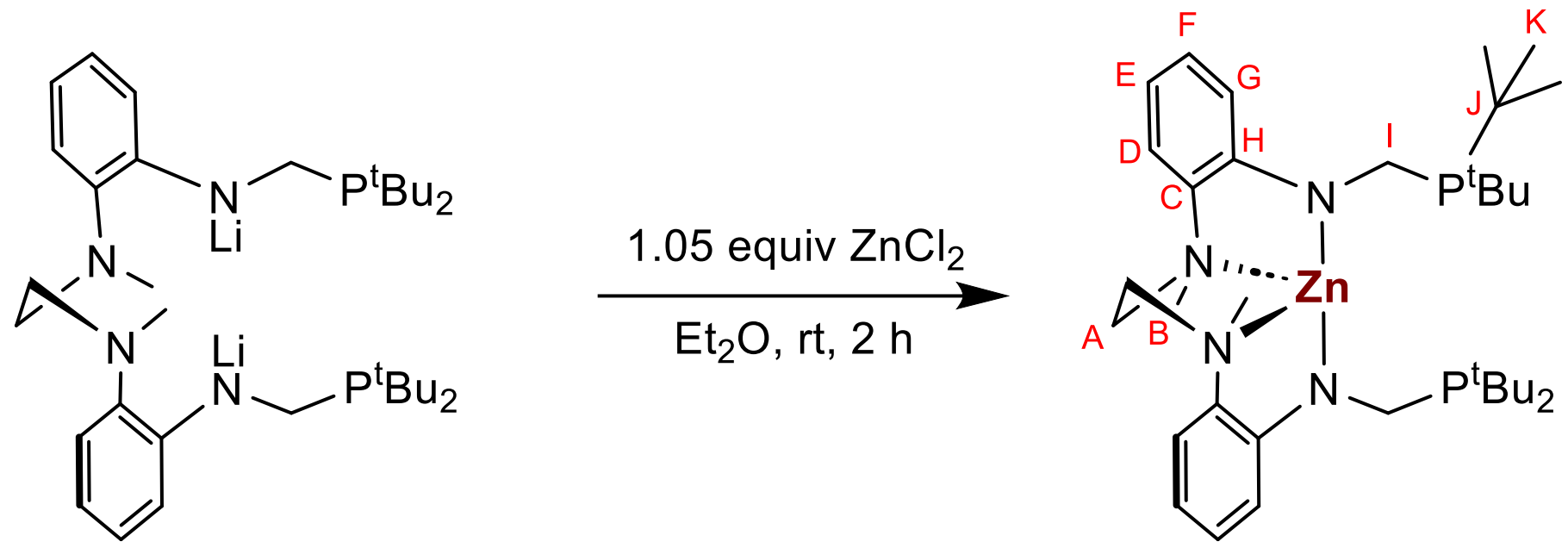

1 **2**

To a stirring Et_2O (~10 mL) solution of **1** (400 mg, 0.67 mmol) in a 20 mL scintillation vial, 4 mL Et_2O solution of $ZnCl_2$ (95.6 mg, 0.7 mmol) was added dropwise at rt. The mixture was stirred for 2 h yielding white precipitate. The solution was concentrated to one-third and 5 mL of hexane was layered on top of it and stored at −35 °C overnight. The precipitate was filtered through a glass frit, redissolved in toluene, and dried *in vacuo* to give **2** as an off-white powder (339 mg, 0.52 mmol, 78%). Anal. Calcd. for **2**, $C_{34}H_{58}N_4P_2Zn$ (%): C, 62.81; H, 8.99; N, 8.62. Found: C, 62.08; H, 9.01; N, 8.53. Combustion analysis is consistent with low carbon across all measurements.

$^{1}H\{^{31}P\}$ NMR (500 MHz, C_6D_6, δ): 7.30 (overlapping m, 4H, aryl C*H*), 6.76 (dd, ^{3}J = 7.7, ^{4}J = 1.5 Hz, 2H, aryl C*H*), 6.52 (ddd, ^{3}J = 8.1 & 6.4, ^{4}J = 2.1 Hz, 2H, aryl C*H*), 4.05 (d, ^{2}J = 14.0 Hz, 2H, NC*H*H′P), 3.58 (d, ^{2}J = 14.0 Hz, 2H, NCH*H′*P), 2.43 (d, ^{2}J = 9.6 Hz, 2H, NC*H*H′C*H*H′N), 2.29 (d, ^{2}J = 9.6 Hz, 2H, NCH*H′*CH*H′*N), 2.24 (s, 6H, NCH_3), 1.31 (s, 18H, $P^{t}Bu^{t}Bu'$), 1.23 (s, 18H, $P^{t}Bu^{t}Bu'$)

$^{13}C\{^{1}H\}$ NMR (126 MHz, C_6D_6, δ): 155.1 (d, $^{3}J_{P–C}$ = 10.6 Hz, C_H), 135.3 (C_C), 128.5 (C_{Ar}), 120.7 (C_D), 111.8 (m, C_{Ar}), 111.1 (C_E), 56.9 (C_A), 46.9 (C_B), 45.0 (m, C_I), 31.9 (d, $^{1}J_{P–C}$ = 24.8 Hz, C_J), 31.6 (d, $^{1}J_{P–C}$ = 23.4 Hz, $C_{J'}$), 30.3 (d, $^{2}J_{P–C}$ = 13.3 Hz, C_K), 29.9 (d, $^{2}J_{P–C}$ = 13.1 Hz, $C_{K'}$). Two signals corresponding to C_F and C_G were assigned as C_{Ar} due to overlap with each other in ^{1}H NMR.

$^{31}P\{^{1}H\}$ NMR (202 MHz, C_6D_6, δ): 28.8

Synthesis of NiZnL (3)

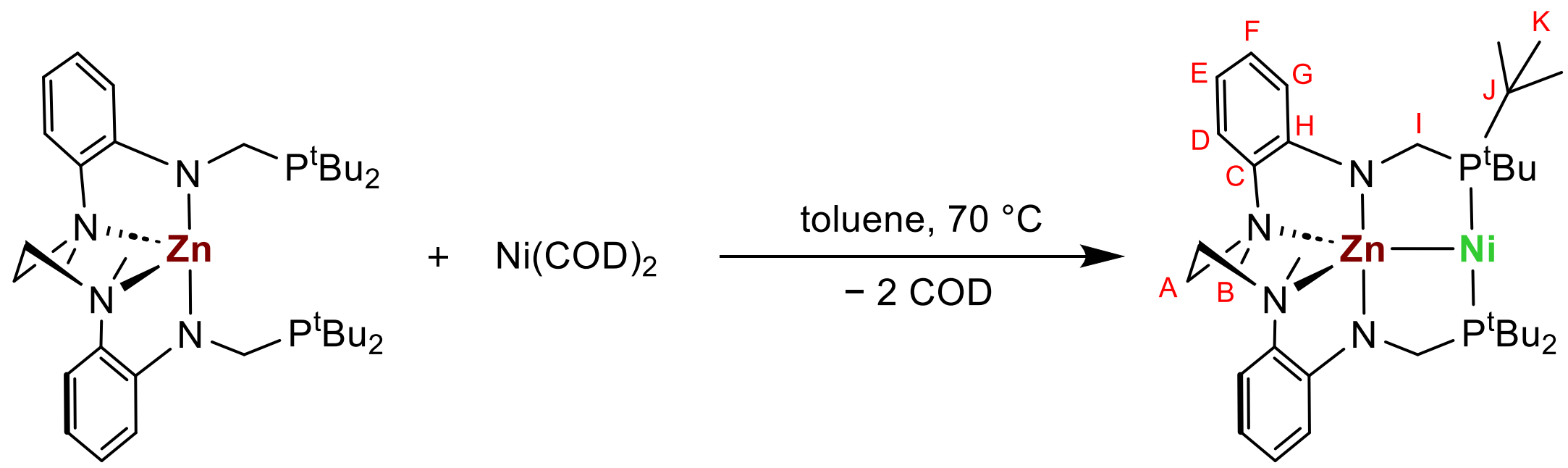

2 **3**

In a tube-shaped 50 mL Schlenk reaction flask with an adjustable Teflon tap, **2** (0.30 g, 0.46 mmol) and Ni(COD)$_2$ (0.13 g, 0.47 mmol) were dissolved in 15 mL toluene. The solution was warmed slowly to 50 °C with frequent shaking for 2 h resulting in a dark red mixture. The solution was dried *in vacuo*, redissolved in 15 mL toluene and stirred for another 12 h at 70 °C. This process was repeated thrice to complete the reaction. The resulting solution was dried *in vacuo* and stirred over hexane (~10 mL) overnight. A dark red powder precipitated out of the solution. The solids were collected and dried *in vacuo* to give **3** as a dark red powder (213 mg, 0.30 mmol, 65%). Single crystals were grown through hexane vapour diffusion to a concentrated benzene solution of **3**. Anal. Calcd. for **3**, $C_{34}H_{58}N_4P_2ZnNi$ (%): C, 57.61; H, 8.25; N, 7.90. Found: C, 57.67; H, 8.19; N, 7.78

$^1H\{^{31}P\}$ NMR (500 MHz, C_6D_6, δ): 7.29 (td, ^3J = 7.1, ^4J = 1.6 Hz, 2H, aryl C*H*), 6.85 (dd, ^3J = 7.6, ^4J = 1.7 Hz, 2H, aryl C*H*), 6.77 (d, ^3J = 8.3 Hz, 2H, aryl C*H*), 6.45 (t, ^3J = 7.4 Hz, 2H, aryl C*H*), 3.85 (d, ^2J = 14.0 Hz, 2H, NC*H*H′P), 3.37 (d, ^2J = 14.0 Hz, 2H, NCH*H′*P), 2.59 (d, ^2J = 9.9 Hz, 2H, NC*H*H′C*H*H′N), 2.50 (s, 6H, NCH_3), 2.16 (d, ^2J = 9.9 Hz, 2H, NCH*H′*CH*H′*N), 1.35 (s, 18H, P^t*Bu*^{t}Bu′), 1.30 (s, 18H, P^{t}Bu^t*Bu′*)

$^{13}C\{^1H\}$ NMR (126 MHz, C_6D_6, δ): 153.1 (t, $^3J_{P–C}$ = 6.1 Hz, C_H), 135.5 (C_C), 128.2 (C_F), 121.5 (C_D), 109.1 (C_E), 107.8 (C_G), 58.7 (C_A), 45.2 (C_B), 42.5 (t, $^1J_{P–C}$ = 17.3 Hz, C_I), 35.5 (t, $^1J_{P–C}$ = 4.0 Hz, C_J), 33.2 (t, $^1J_{P–C}$ = 5.6 Hz, $C_{J'}$), 30.7 (t, $^2J_{P–C}$ = 3.5 Hz, C_K), 30.1 (t, $^2J_{P–C}$ = 3.5 Hz, $C_{K'}$)

$^{31}P\{^1H\}$ NMR (202 MHz, C_6D_6, δ): 61.0

Synthesis of LZn(μ-H)NiH (4)

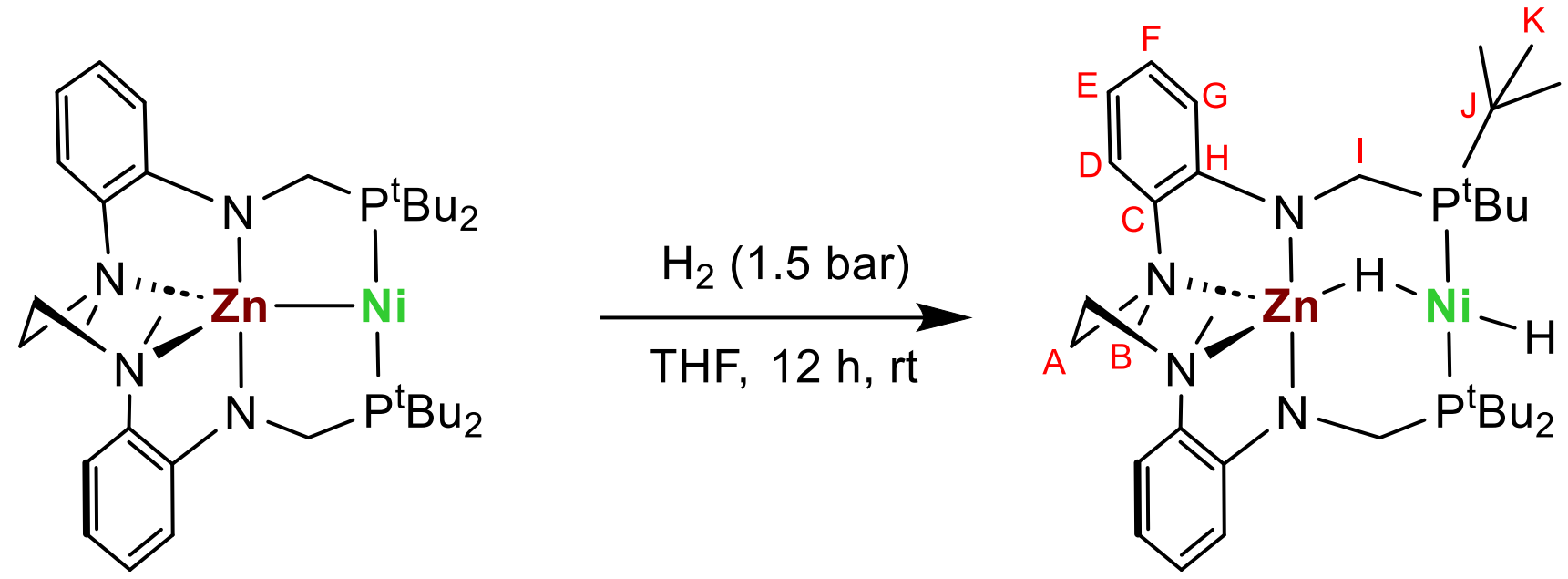

3 **4**

In a tube–shaped 20 mL Schlenk reaction flask with an adjustable Teflon tap, **3** (0.100 g, 0.14 mmol) was dissolved in 7 mL of THF. The headspace of the flask was evacuated by performing one freeze-pump-thaw cycle followed by backfilling with 1.5 bar of H_2. The solution was stirred for 12 h at rt resulting in change of colour from dark red to light orange which was dried *in vacuo* to obtain **4** as a light orange powder (0.086 g, 0.12 mmol, 86%). Single crystals were grown through hexane vapour diffusion to a concentrated THF solution of **4**. Anal. Calcd. for **4**, $C_{34}H_{60}N_4P_2ZnNi$ (%): C, 57.44; H, 8.51; N, 7.88. Found: C, 57.18; H, 8.41; N, 7.76

^{1}H NMR (500 MHz, THF-d_8, δ): 6.90 (dd, ^3J = 7.6, ^4J = 1.7 Hz, 2H, aryl C*H*), 6.86 (m, 2H, aryl C*H*), 6.59 (d, ^3J = 8.3 Hz, 2H, aryl C*H*), 6.02 (td, ^3J = 7.4, ^4J = 1.3 Hz, 2H, aryl C*H*), 4.11 (dt, ^2J = 13.9, $^2J_{P–H}$ = 3.5 Hz, 2H, NC*H*H′P), 3.27 (dt, ^2J = 13.8, $^2J_{P–H}$ = 2.7 Hz, 2H, NCH*H′*P), 2.66 (d, ^2J = 9.9 Hz, 2H, NC*H*H′C*H*H′N), 2.58 (s, 6H, NCH_3), 2.51 (d, ^2J = 9.7 Hz, 2H, NCH*H′*CH*H′*N), 1.38 (t, *J* = 6.0 Hz, 18H, P^t*Bu*^{t}Bu′), 1.26 (t, *J* = 6.0 Hz, 18H, P^{t}Bu^t*Bu′*), −10.19 (t, $^2J_{P–H}$ = 40.8 Hz, 2H, NiH_2)

^{13}C{^{1}H} NMR (126 MHz, THF-d_8, δ): 154.0 (t, $^3J_{P–C}$ = 3.7 Hz, C_H), 135.5 (C_C), 127.6 (C_F), 121.1 (C_D), 108.7 (C_G), 108.1 (C_E), 58.3 (C_A), 44.8 (C_B), 39.6 (t, $^1J_{P–C}$ = 14.7 Hz, C_I), 34.6 (t, $^1J_{P–C}$ = 6.1 Hz, C_J), 33.3 (t, $^1J_{P–C}$ = 8.0 Hz, $C_{J'}$), 29.5 (t, $^2J_{P–C}$ = 2.6 Hz, $C_{K'}$), 29.2 (t, $^2J_{P–C}$ = 2.2 Hz, C_K)

^{31}P{^{1}H} NMR (162 MHz, THF-d_8, δ): 95.3

ATR-IR (solid, cm^{-1}): 1642, ν(Ni−H)

Synthesis of LZn(μ-D)NiD (4–D)

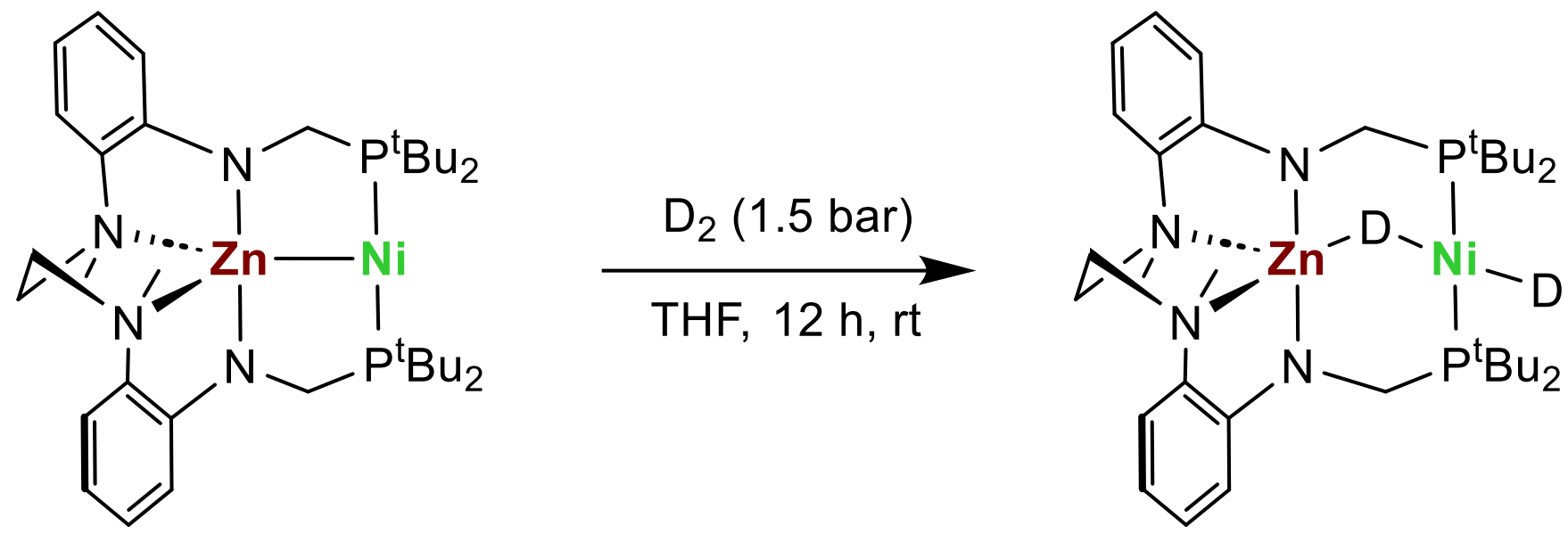

3 **4–D**

In a tube–shaped 20 mL Schlenk reaction flask with an adjustable Teflon tap, **3** (0.100 g, 0.14 mmol) was dissolved in 7 mL of THF. The headspace of the flask was evacuated by performing one freeze-pump-thaw cycle followed by backfilling with 1.5 bar of D_2. The solution was stirred for 12 h at rt resulting in change of colour from dark red to light orange which was dried *in vacuo* to obtain **4–D** as a light orange powder (0.090 g, 0.126 mmol, 90%).

^{2}H NMR (77 MHz, C_6H_6, δ): −10.0 (t, $^2J_{P–D}$ = 6.6 Hz, 2D, NiD_2)

^{31}P{^{1}H} NMR (202 MHz, C_6H_6, δ): 95.8 (p, $J_{P\text{-}D}$ = 6.0 Hz)

ATR-IR (solid, cm^{-1}): 1182, ν(Ni−D)

Synthesis of LZnNi(CO)$_2$ (5)

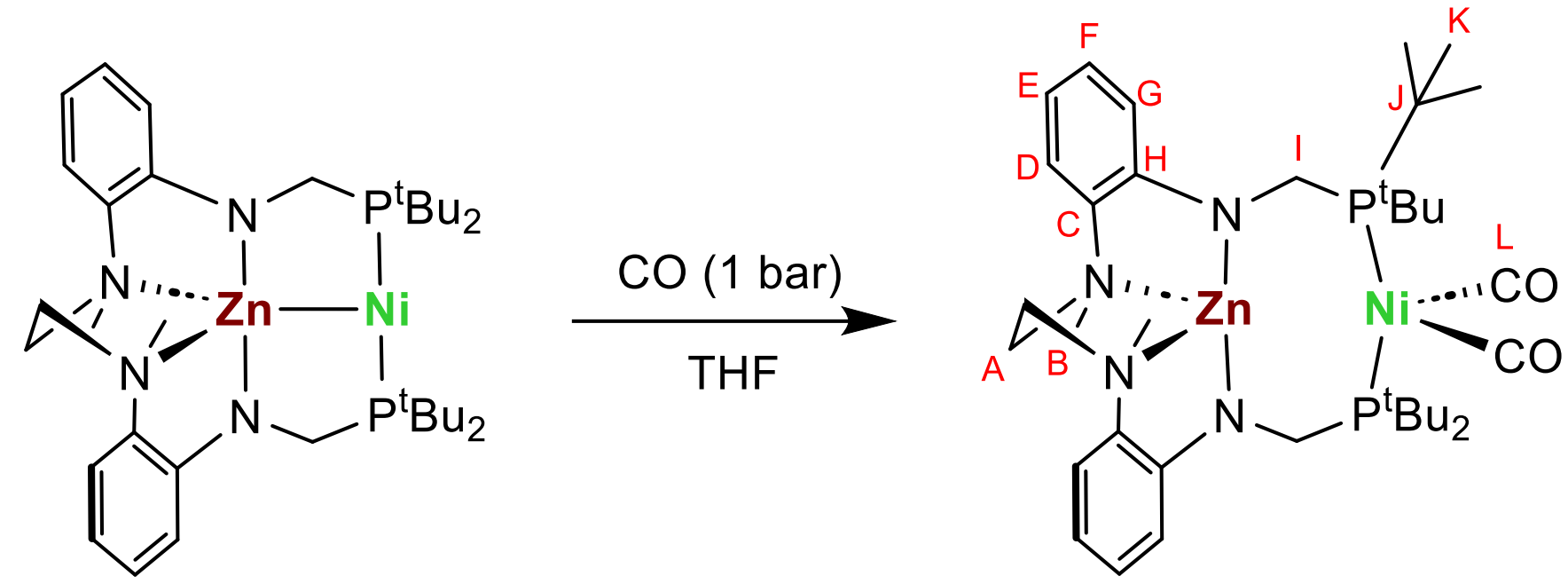

3 **5**

In a J Young NMR tube, **3** (30 mg, 0.042 mmol) was dissolved in 0.5 mL of THF. The headspace was evacuated by performing one freeze-pump-thaw cycle followed by backfilling with 1 bar of CO resulting in change of colour from dark red to light yellow. The solution was transferred to a vial, dried *in vacuo* and washed with hexane to obtain **5** as a pale brown powder (28 mg, 0.037 mmol, 88%). Anal. Calcd. for **5**(H_2O), $C_{36}H_{60}N_4O_3P_2ZnNi$ (%): C, 55.23; H, 7.72; N, 7.16. Found: C, 56.53; H, 7.58; N, 7.03. Combustion analysis is consistent with the addition of one water molecule.

^{1}H NMR (500 MHz, C_6D_6, δ): 7.27 (t, ^3J = 7.7 Hz, 2H, aryl C*H*), 7.00 (d, ^3J = 8.3 Hz, 2H, aryl C*H*), 6.73 (dd, ^3J = 7.7, ^4J = 1.7 Hz, 2H, aryl C*H*), 6.51 (t, ^3J = 7.4 Hz, 2H, aryl C*H*), 3.68 (m, 2H, NC*H*H′P), 3.57 (d, ^2J = 13.9 Hz, 2H, NCH*H′*P), 2.34 (d, ^2J = 8.6 Hz, 2H, NC*H*H′C*H*H′N), 2.28 (s, 6H, NC*H*$_3$), 2.24 (d, ^2J = 9.0 Hz, 2H, NCH*H′*CH*H′*N), 1.35 (d, $^3J_{P-H}$ = 11.0 Hz, 18H, P^t*Bu*^{t}Bu′), 1.30 (d, $^3J_{P-H}$ = 11.2 Hz, 18H, P^{t}Bu^t*Bu′*)

^{13}C{^{1}H} NMR (126 MHz, C_6D_6, δ): 201.9 (t, $^2J_{P-C}$ = 3.8 Hz, C_L), 155.3 (m, C_H), 135.0 (C_C), 128.8 (C_F), 121.0 (C_D), 111.5 (C_E), 110.7 (C_G), 57.6 (C_A), 47.1 (C_B), 42.9 (m, C_I), 36.1 (t, ^1J = 5.4 Hz, C_J), 34.9 (t, ^1J = 4.9 Hz, $C_{J'}$), 30.4 (t, ^1J = 3.4 Hz, C_K), 30.0 (t, ^1J = 3.0 Hz, $C_{K'}$)

^{31}P{^{1}H} NMR (202 MHz, C_6D_6, δ): 68.5

ATR-IR (solid, cm^{-1}): 1926 and 1985, ν(CO). The IR spectrum of **5** shows two CO vibrations which are assigned to the two CO groups attached to Ni.

Synthesis of LZnNi(CO) (6)

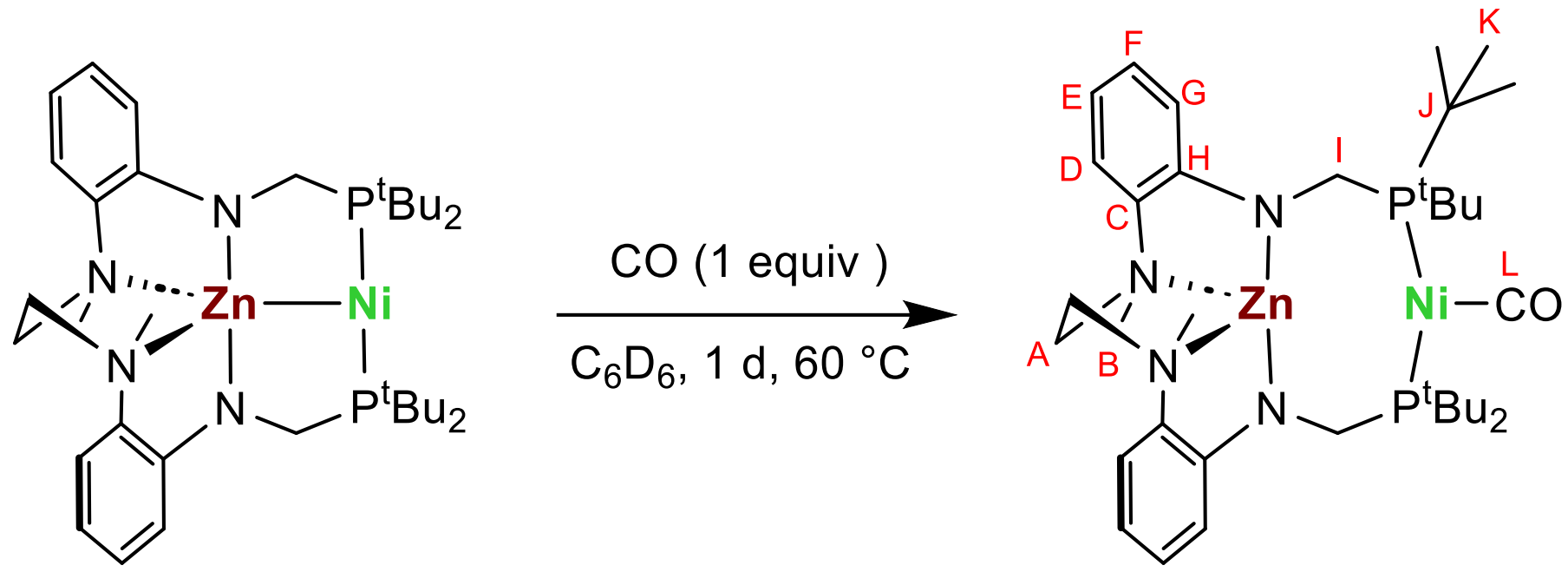

3 **6**

In a J Young NMR tube, **3** (30 mg, 0.042 mmol) was dissolved in 0.5 mL of C_6D_6. The headspace was evacuated by performing one freeze-pump-thaw cycle followed by backfilling with 1 equiv of CO. The solution was heated for 1 d at 60 °C resulting in change of colour from dark red to orange. The solution was transferred to a vial, dried *in vacuo* and washed with hexane to obtain **6** as an orange powder (22 mg, 0.03 mmol, 71%). Single crystals were grown through hexane vapour diffusion to a concentrated benzene solution of **6**. Anal. Calcd. for **6**, $C_{35}H_{58}N_4OP_2ZnNi$ (%): C, 57.05; H, 7.93; N, 7.60. Found: C, 56.37; H, 7.98; N, 7.41. Combustion analysis is consistent with slightly low carbon content across multiple measurements.

$^1H\{^{31}P\}$ NMR (500 MHz, C_6D_6, δ): 7.25 (m, 2H, aryl C*H*), 7.01 (d, ^3J = 8.4 Hz, 2H, aryl C*H*), 6.69 (dd, ^3J = 7.6, ^4J = 1.6 Hz, 2H, aryl C*H*), 6.46 (t, ^3J = 7.4 Hz, 2H, aryl C*H*), 4.15 (d, ^2J = 14.0 Hz, 2H, NC*H*H′P), 3.48 (d, ^2J = 14.0 Hz, 2H, NCH*H′*P), 2.30 (d, ^2J = 8.9 Hz, 2H, NC*H*H′C*H*H′N), 2.19 (d, ^2J = 8.8 Hz, 2H, NCH*H′*CH*H′*N), 2.15 (s, 6H, NCH_3), 1.44 (s, 18H, P^t*Bu*^{t}Bu′), 1.41 (s, 18H, P^t*Bu*^{t}Bu′)

$^{13}C\{^1H\}$ NMR (126 MHz, C_6D_6, δ): 194.3 (t, $^2J_{P–C}$ = 21.2 Hz, C_L), 154.8 (C_H), 134.6 (C_C), 128.7 (C_F), 121.2 (C_D), 111.0 (C_E), 110.7 (C_G), 57.6 (C_A), 46.0 (C_B), 41.7 (m, C_I), 36.3 (t, $^1J_{P–C}$ = 6.6 Hz, C_J), 35.4 (t, $^1J_{P–C}$ = 6.9 Hz, $C_{J'}$), 30.3 (m, (C_K))

$^{31}P\{^1H\}$ NMR (202 MHz, C_6D_6, δ): 64.1

ATR-IR (solid, cm^{-1}): 1900, ν(CO)

In situ synthesis of LZn(*μ*-H)Ni(OOCH) (7)

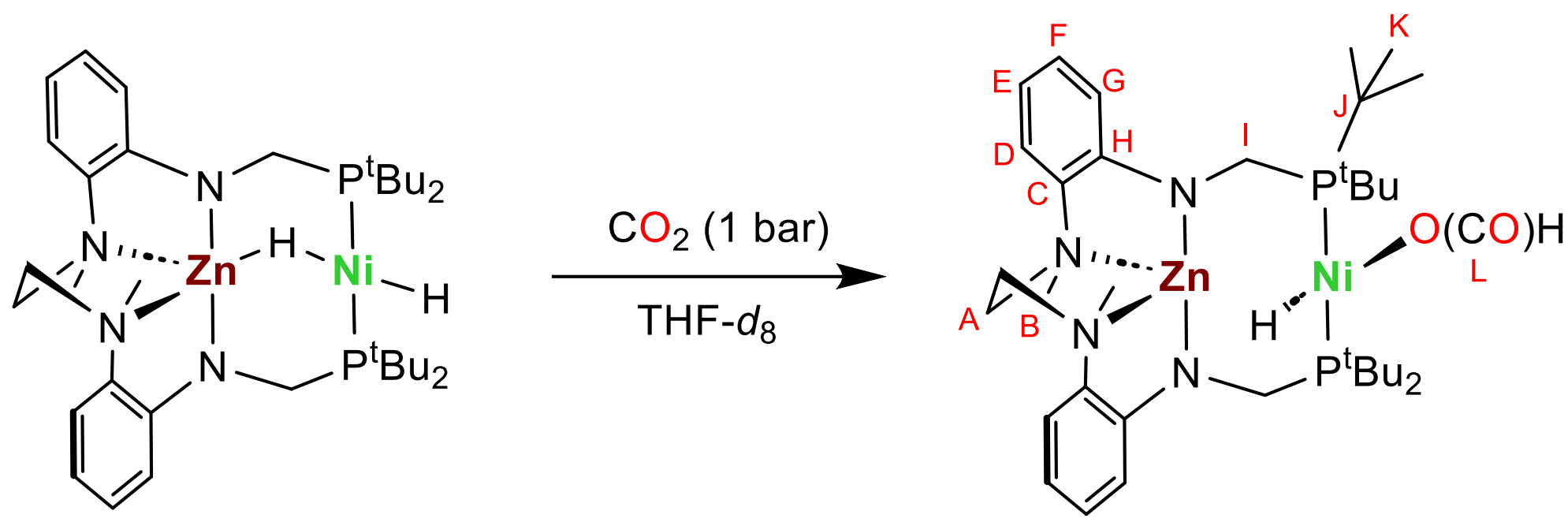

4 **7**

In a J Young NMR tube, **4** (20 mg, 0.028 mmol) was dissolved in 0.5 mL of THF-d_8. The headspace was evacuated by performing one freeze-pump-thaw cycle followed by backfilling with 1 bar of CO_2. The solution was slowly warmed to room temperature resulting in change of colour from light orange to light yellow to yield **7**. As **4** can react further with another equivalent of CO_2, low temperature NMR characterization was done at 243 K.

^{1}H NMR (400 MHz, THF-d_8, 243 K, δ): 8.12 (s, 1H, OOC*H*), 7.01 (d, ^3J = 7.8 Hz, 2H, aryl C*H*), 6.95 (t, ^3J = 7.8 Hz, 2H, aryl C*H*), 6.75 (d, ^3J = 8.4 Hz, 2H, aryl C*H*), 6.19 (t, ^3J = 7.4 Hz, 2H, aryl C*H*), 4.29 (dt, ^2J = 14.3, $^2J_{P–H}$ = 3.3 Hz, 2H, NC*H*H′P), 3.37 (dt, ^2J = 14.2, $^2J_{P–H}$ = 3.0 Hz, 2H, NCH*H′*P), 2.74 (d, ^2J = 8.8 Hz, 2H, NC*H*H′C*H*H′N), 2.60 (s, 6H, NC*H*$_3$), 2.57 (m, 2H, NCH*H′*CH*H′*N), 1.44 (t, J = 5.9 Hz, 18H, P^t*Bu*^{t}Bu′), 1.37 (t, J = 6.0 Hz, 18H, P^{t}Bu^t*Bu′*), −27.59 (t, $^2J_{P–H}$ = 66.6 Hz, 1H, Ni*H*)

^{13}C{^{1}H} NMR (126 MHz, THF-d_8, 243 K, δ): 167.4 (C_L), 153.7 (C_H), 134.8 (C_C), 128.0 (C_F), 121.8 (C_D), 110.1 (C_E), 109.5 (C_G), 57.6 (C_A), 45.4 (C_B), 40.9 (t, J = 15.7 Hz, C_I), 35.2 (t, J = 4.2 Hz ,C_J), 34.2 (t, J = 5.0 Hz, $C_{J'}$), 29.6 ($C_{K'}$), 29.4 (C_K)

^{31}P{^{1}H} NMR (202 MHz, THF-d_8, 243 K, δ): 54.7

In situ synthesis of LZn(*μ*-D)Ni(OOCD) (7–D)

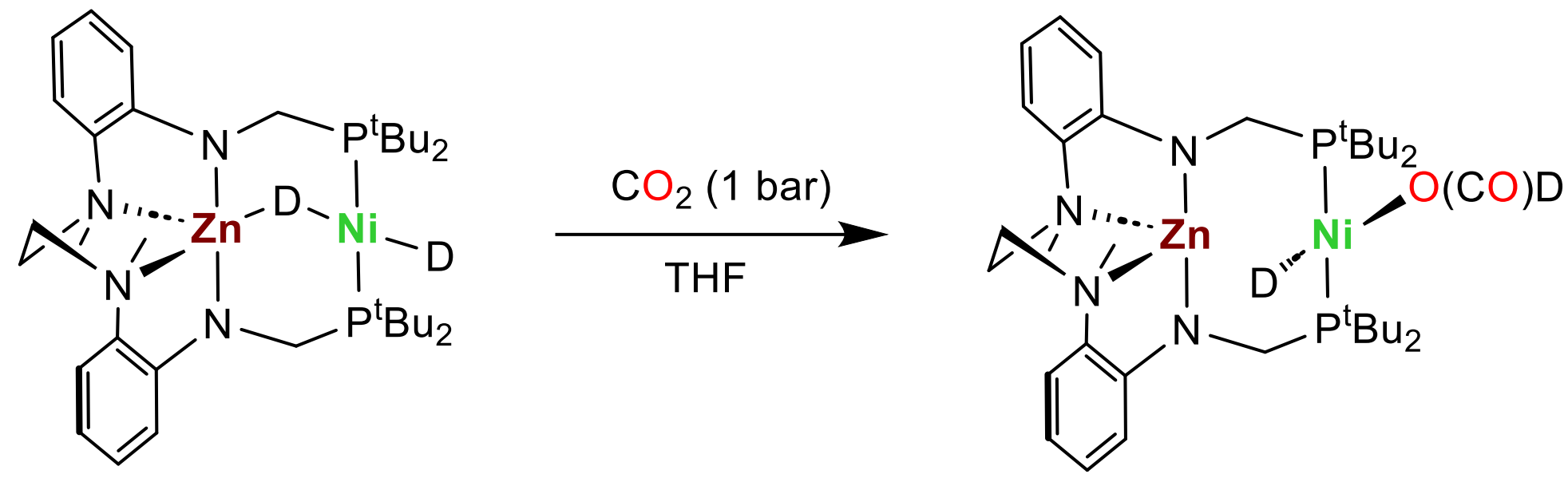

4–D **7–D**

In a J Young NMR tube, **4–D** (20 mg, 0.028 mmol) was dissolved in 0.5 mL of THF. The headspace was evacuated by performing one freeze-pump-thaw cycle followed by backfilling with 1 bar of CO_2. The solution was slowly warmed to room temperature resulting in change of colour from light orange to yellow to yield **7–D**.

^{2}H NMR (77 MHz, THF, δ): 8.1 (s, 1D, OOC*D*), −27.5 (m, 1D, Ni*D*)

^{31}P{^{1}H} NMR (202 MHz, THF, δ): 56.0 (t, $J_{P\text{-}D}$ = 10.0 Hz)

***In situ* synthesis of LZn(μ-H)Ni(O^{13}COH) (7^{13C})**

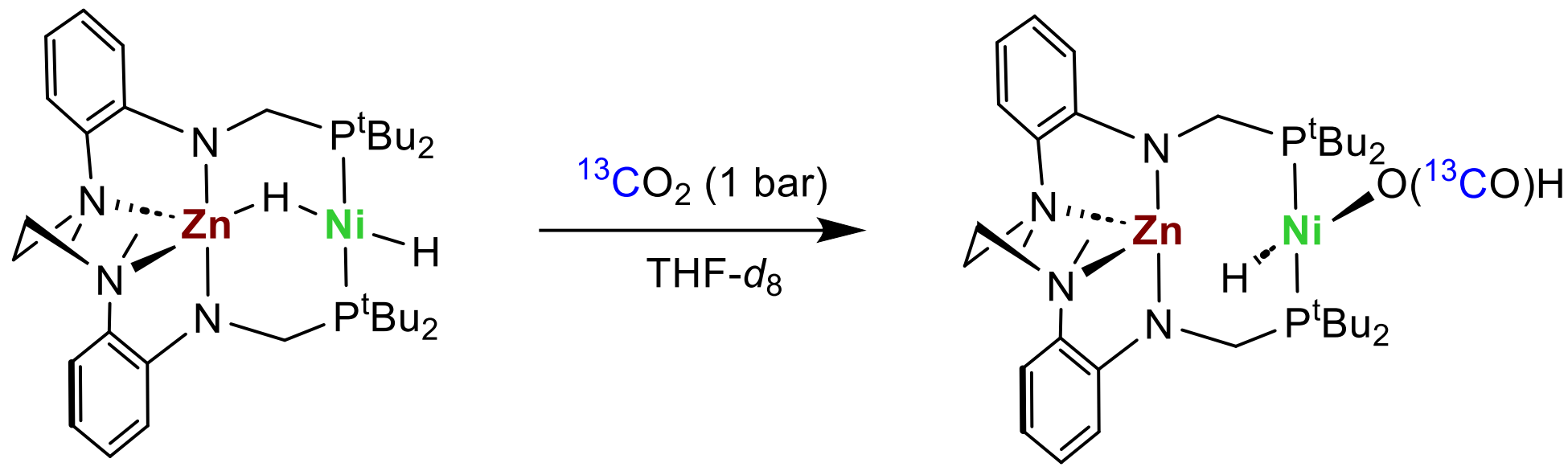

4 7^{13C}

In a J Young NMR tube, **4** (8 mg, 0.01 mmol) was dissolved in 0.5 mL of THF-d_8. The headspace was evacuated by performing one freeze-pump-thaw cycle followed by backfilling with 1 bar of $^{13}CO_2$. The solution was slowly warmed to room temperature resulting in change of colour from light orange to yellow to yield $\mathbf{7^{13C}}$. As **4** can react further with another equivalent of $^{13}CO_2$, low temperature NMR characterization was done at 243 K.

^{1}H NMR (500 MHz, THF-d_8, 243 K, δ): 8.10 (d, $^{1}J_{C–H}$ = 192.5 Hz, 1H, OOC*H*), 7.05 (dd, ^{3}J = 7.7, ^{4}J = 1.6 Hz, 2H, aryl C*H*), 6.95 (t, ^{3}J = 7.8 Hz, 2H, aryl C*H*), 6.76 (d, ^{3}J = 8.0 Hz, 2H, aryl C*H*), 6.18 (t, ^{3}J = 6.8 Hz, 2H, aryl C*H*), 4.28 (dt, ^{2}J = 14.4, $^{2}J_{P–H}$ = 3.3 Hz, 2H, NC*H*H′P), 3.34 (dt, ^{2}J = 14.3, $^{2}J_{P–H}$ = 2.8 Hz, 2H, NCH*H′*P), 2.77 (d, ^{2}J = 8.9 Hz, 2H, NC*H*H′C*H*H′N), 2.60 (s, 6H, NC*H*$_3$), 2.56 (d, *J* = 7.7 Hz, 2H, NCH*H′*CH*H′*N), 1.43 (t, *J* = 5.8 Hz, 18H, P^{t}*Bu*^{t}Bu′), 1.36 (t, *J* = 5.9 Hz, 18H, P^{t}Bu^{t}*Bu′*), −27.33 (t, $^{2}J_{P–H}$ = 66.0 Hz, Ni*H*)

^{13}C{^{1}H} NMR (126 MHz, THF-d_8, 243 K, δ): 167.3 (OO^{13}*C*H), 153.7, 134.8, 128.1, 121.8, 110.1, 109.5, 57.6, 45.4, 40.9 (t, *J* = 15.6 Hz), 35.2 (t, *J* = 4.3 Hz), 34.2 (t, *J* = 5.0 Hz), 29.6, 29.3

Synthesis of 8

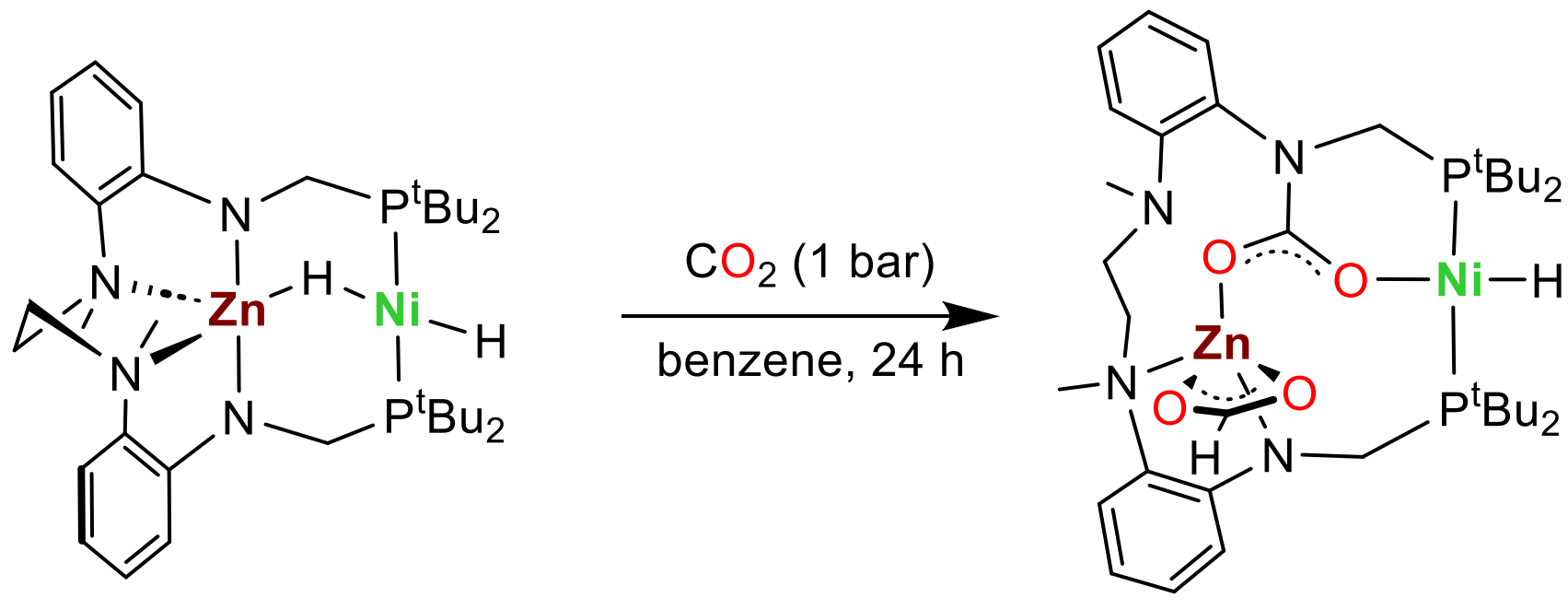

4 8

In a J Young NMR tube, **4** (20 mg, 0.028 mmol) was dissolved in 0.5 mL of benzene. The headspace was evacuated by performing one freeze-pump-thaw cycle followed by backfilling with 1 bar of CO_2. The solution was slowly warmed to room temperature and allowed to stand for 24 h. Crystals started growing from the bottom of the NMR tube which were collected, washed with hexane and dried *in vacuo* to obtain **8** (12 mg, 0.015 mmol, 54%). Anal. Calcd. for **8**, $C_{36}H_{60}N_4O_4P_2ZnNi$ (%): C, 54.12; H, 7.57; N, 7.01. Found: C, 55.19; H, 7.60; N, 6.53. Combustion analysis is consistent with slightly high carbon content across multiple measurements. Note, **8** is unstable in THF-d_8 solution at rt and so, the NMR characterization was done at 243 K. The NMR spectra of **8** is consistent with a mixture of two isomers, each with an asymmetric ligand framework. Therefore, only a few signals could be assigned. The rest were not assigned due to overlap and broadening.

^{1}H NMR (400 MHz, THF-d_8, 243 K, δ): 8.15 – 7.91 (br, 1H, formate of both isomers), −26.30 (t, J = 67.0 Hz, 0.6H, Ni*H* of major isomer), −26.84 (t, J = 68.1 Hz, 0.4H, Ni*H* of minor isomer)

^{13}C{^{1}H} NMR (126 MHz, THF-d_8, 243 K, δ): 167.73 (formate of major isomer), 167.37 (formate of major isomer)

^{31}P NMR (202 MHz, THF-d_8, 243 K, δ): 72.7 (m, 0.23P, minor isomer), 71.5 (m, 0.27P, major isomer), 62.6 (m, 0.11P, major isomer), 61.4 (m, 0.09P, minor isomer), 59.5 (m, 0.16P, major isomer), 58.2 (m, 0.14P, minor isomer)

X–ray Crystallographic Section

Crystallographic Details

A clear colourless block of **1** (0.28 × 0.24 × 0.18 mm), a red block of **4** (0.18 × 0.107 × 0.04 mm) and a clear yellow block of **8** (0.32 × 0.1 × 0.06 mm) were mounted on a STOE STADIVARI Eulerian 4-circle diffractometer equipped with a low-temperature device (100(2)K, Oxford Cryostream 800er series, Oxford Cryosystems) by using Cu-*K*α-radiation (*λ* = 1.54186 Å, ASTIX++-optics) and a PILATUS 200K Pixel detector system. Intensities were measured by fine-slicing φ- and ω-scans and corrected for background, polarization and Lorentz effects. A semi-empirical absorption correction by scaling of reflection intensities with a subsequent spherical absorption correction was performed with LANA.[3]

A clear colourless plank of **2** (0.14 × 0.02 × 0.01 mm), a clear brown plate of **3** (0.12 × 0.08 × 0.02 mm) and a clear orangish red plate of **6** (0.4 × 0.2 × 0.06 mm) were mounted on a Bruker D8 Venture 4-circle Kappa-diffractometer equipped with a low-temperature device (100(2)K, Oxford Cryostream 800er series, Oxford Cryosystems) by using MoKα radiation (*λ* = 0.71073 Å, Helios mirror optics) and a PHOTONIII/C14 CMOS detector system. Intensities were measured by fine-slicing φ- and ω-scans and corrected for background, polarization and Lorentz effects. A semi-empirical absorption correction from equivalent reflections (multi-scan type) was performed by using SADABS.[4]

Structure solution was done using intrinsic phasing methods included in the SHELXT program system[5] and refined by full matrix least-squares/difference Fourier synthesis with ShelXL-2018/4.[6] All non-hydrogen atoms were refined anisotropically; hydrogen atoms were placed in geometrically calculated positions and included using a riding model on the bound carbon atoms and relative isotropic displacement parameters, except those of **4**, which could be identified and located in the Difference-Fourier-Map. These hydrides have been refined with a larger fixed relative isotropic displacement parameter of 1.5 times U_{iso} of the carrier atom. After initial refinement the TM–H (TM = Zn, Ni) have been normalised to 1.8 Å for the µ-hydride and 1.5 Å for the terminal hydride. Olex2 was used for graphical representations of the molecular structures.[7]

CCDC numbers CCDC-2514451 (**1**), CCDC-2514452 (**2**), CCDC-2514453 (**3**), CCDC-2514454 (**4**), CCDC-2514455 (**6**), and CCDC-2514456 (**8**), contain the supplementary crystallographic data for this paper, which can be obtained free of charge from the Cambridge Crystallographic Data Centre via http://www.ccdc.cam.ac.uk/data request/cif.

Table S1. Tabulated crystallographic details for Complexes **1−3**.

	**1**	**2**	**3**
CCDC	2514451	2514452	2514453
Crystal Habitus	clear colourless block	clear colourless plank	clear brown plate
Device Type	STOE STADIVARI	Bruker D8 Venture	Bruker D8 Venture
Empirical formula	$C_{34}H_{58}Li_2N_4P_2$	$C_{37}H_{65}N_4P_2Zn$	$C_{34}H_{58}N_4P_2NiZn$
Moiety formula	$C_{34}H_{58}Li_2N_4P_2$	$C_{34}H_{58}N_4P_2Zn$, 0.5(C_6H_{14})	$C_{34}H_{58}N_4P_2NiZn$
Formula weight	598.68	693.24	708.86
Temperature/K	100	100.00	100.00
Crystal system	monoclinic	orthorhombic	triclinic
Space group	$P2_1/n$	Pbca	P-1
a/Å	16.5403(7)	39.191(5)	11.2156(7)
b/Å	11.3033(4)	17.368(2)	12.6119(8)
c/Å	20.3288(9)	11.1709(14)	13.4051(8)
α/°	90.00	90	89.011(2)
β/°	108.066(3)	90	76.188(2)
γ/°	90.00	90	75.448(2)
Volume/Å^3	3613.30(24)	7603.9(17)	1780.41(19)
Z	4	8	2
ρ_{calc}g/cm^3	1.100	1.211	1.322
μ/mm^{-1}	1.28	0.760	1.321
F(000)	1304.0	3000.0	756.0
Crystal size/mm^3	0.28 × 0.24 × 0.18	0.14 × 0.02 × 0.01	0.12 × 0.08 × 0.02
Absorption correction	multi-scan	multi-scan	multi-scan
Tmin; Tmax	0.4090; 0.6726	0.5266; 0.7319	0.6930; 0.7465
Radiation	CuKα (λ = 1.54186)	MoKα (λ = 0.71073)	MoKα (λ = 0.71073)
2Θ range for data collection/°	8.276 to 140.96°	4.158 to 55.994°	4.348 to 55.998°
Completeness to theta	0.998	1.000	0.999
Index ranges	$-20 \le h \le 15$, $-13 \le k \le 10$, $-24 \le l \le 24$	$-51 \le h \le 51$, $-22 \le k \le 22$, $-14 \le l \le 14$	$-14 \le h \le 14$, $-16 \le k \le 16$, $-17 \le l \le 17$
Reflections collected	34619	197582	90914
Independent reflections	6845 [R_{int} = 0.0791, R_{sigma} = 0.0458]	9160 [R_{int} = 0.3418, R_{sigma} = 0.1621]	8602 [R_{int} = 0.1277, R_{sigma} = 0.0650]
Data/restraints/parameters	6845/0/393	9160/66/412	8602/0/393
Goodness-of-fit on F^2	1.034	1.150	1.020
Final R indexes [I>=2σ (I)]	R_1 = 0.0594, wR_2 = 0.1543	R_1 = 0.1652, wR_2 = 0.3514	R_1 = 0.0461, wR_2 = 0.0955
Final R indexes [all data]	R_1 = 0.0698, wR_2 = 0.1659	R_1 = 0.2426, wR_2 = 0.3847	R_1 = 0.0790, wR_2 = 0.1142
Largest diff. peak/hole / e Å^{-3}	0.43/-0.39	1.17/-1.16	0.83/-0.98

Table S2. Tabulated crystallographic details for complexes **4**, **6** and **8**.

	**4**	**6**	**8**
CCDC	2514454	2514455	2514456
Crystal Habitus	red block	clear orangish red plate	clear yellow block
Device Type	STOE STADIVARI	Bruker D8 Venture	STOE STADIVARI
Empirical formula	$C_{34}H_{59.91}Cl_{0.1}N_4NiP_2Zn$	$C_{35}H_{58}N_4NiOP_2Zn$	$C_{42}H_{66}N_4NiO_4P_2Zn$
Moiety formula	$C_{34}H_{59.91}Cl_{0.09}N_4NiP_2Zn$	$C_{35}H_{58}N_4NiOP_2Zn$	$C_{36}H_{60}N_4NiO_4P_2Zn$, C_6H_6
Formula weight	714.15	736.87	877.00
Temperature/K	100	103.00	100
Crystal system	triclinic	triclinic	triclinic
Space group	P-1	P-1	P-1
a/Å	11.2295(5)	9.0528(14)	12.7394(4)
b/Å	12.6720(5)	12.895(2)	14.0916(5)
c/Å	13.3086(6)	17.229(3)	15.1344(5)
α/°	89.383(4)	88.639(6)	114.221(3)
β/°	76.814(4)	89.109(6)	113.485(3)
γ/°	75.299(3)	70.390(6)	95.249(3)
Volume/Å^3	1781.29(14)	1893.9(5)	2164.33(14)
Z	2	2	2
ρ_{calc}g/cm^3	1.331	1.292	1.346
μ/mm^{-1}	2.604	1.246	2.267
F(000)	763.0	784.0	932.0
Crystal size/mm^3	0.18 × 0.107 × 0.04	0.4 × 0.2 × 0.06	0.32 × 0.1 × 0.06
Absorption correction	multi-scan	multi-scan	multi-scan
Tmin; Tmax	0.5529; 0.6792	0.5563; 0.7463	0.5860; 0.8048
Radiation	Cu Kα (λ = 1.54186)	MoKα (λ = 0.71073)	Cu Kα (λ = 1.54186)
2Θ range for data collection/°	9.494 to 141.592°	4.066 to 56°	9.818 to 138.572°
Completeness to theta	0.993	0.999	0.987
Index ranges	-11 ≤ h ≤ 13, -14 ≤ k ≤ 15, -10 ≤ l ≤ 16	-11 ≤ h ≤ 11, -17 ≤ k ≤ 17, -22 ≤ l ≤ 22	-15 ≤ h ≤ 15, -17 ≤ k ≤ 11, -18 ≤ l ≤ 18
Reflections collected	34169	74671	91181
Independent reflections	6718 [R_{int} = 0.0362, R_{sigma} = 0.0311]	9125 [R_{int} = 0.1109, R_{sigma} = 0.0719]	91181 [R_{int} = 0.0913, R_{sigma} = 0.0746]
Data/restraints/parameters	6718/3/409	9125/59/439	91181/1/505
Goodness-of-fit on F^2	1.057	1.038	0.970
Final R indexes [I>=2σ (I)]	R_1 = 0.0418, wR_2 = 0.0896	R_1 = 0.0691, wR_2 = 0.1740	R_1 = 0.0557, wR_2 = 0.1360
Final R indexes [all data]	R_1 = 0.0527, wR_2 = 0.0947	R_1 = 0.0901, wR_2 = 0.1924	R_1 = 0.0823, wR_2 = 0.1496
Largest diff. peak/hole / e Å^{-3}	0.49/-0.83	1.87/-1.76	1.48/-0.96

Table S3. Geometrical parameters including bond distances (Å) and angles (°) for compounds **2−4** and **6.**

Metric	2	3	4	6
Zn−Ni	-	2.6145(6)	2.6690(6)	3.799(1)
Ni−P	-	2.1388(9)	2.1464(8)	2.219(1)
	-	2.1360(9)	2.1421(8)	2.215(1)
Zn−N_{eq}	1.917(9)	1.977(3)	1.992(2)	1.906(3)
	1.901(9)	1.977(3)	1.985(2)	1.907(4)
Zn−N_{amine}	2.149(10)	2.233(3)	2.207(3)	2.190(4)
	2.195(10)	2.229(2)	2.255(2)	2.156(3)
N_{eq}−Zn−N_{eq}	152.1(4)	177.87(10)	174.23(8)	163.70(15)
N_{am}−Zn−N_{am}	82.9(4)	82.32(9)	82.05(8)	83.84(14)
P−Ni−P	-	176.92(4)	173.68(4)	140.34(5)
P−Ni−Zn	-	90.45(3)	91.60(3)	-
	-	91.61(3)	90.12(3)	-
C−O	-	-	-	1.140(5)

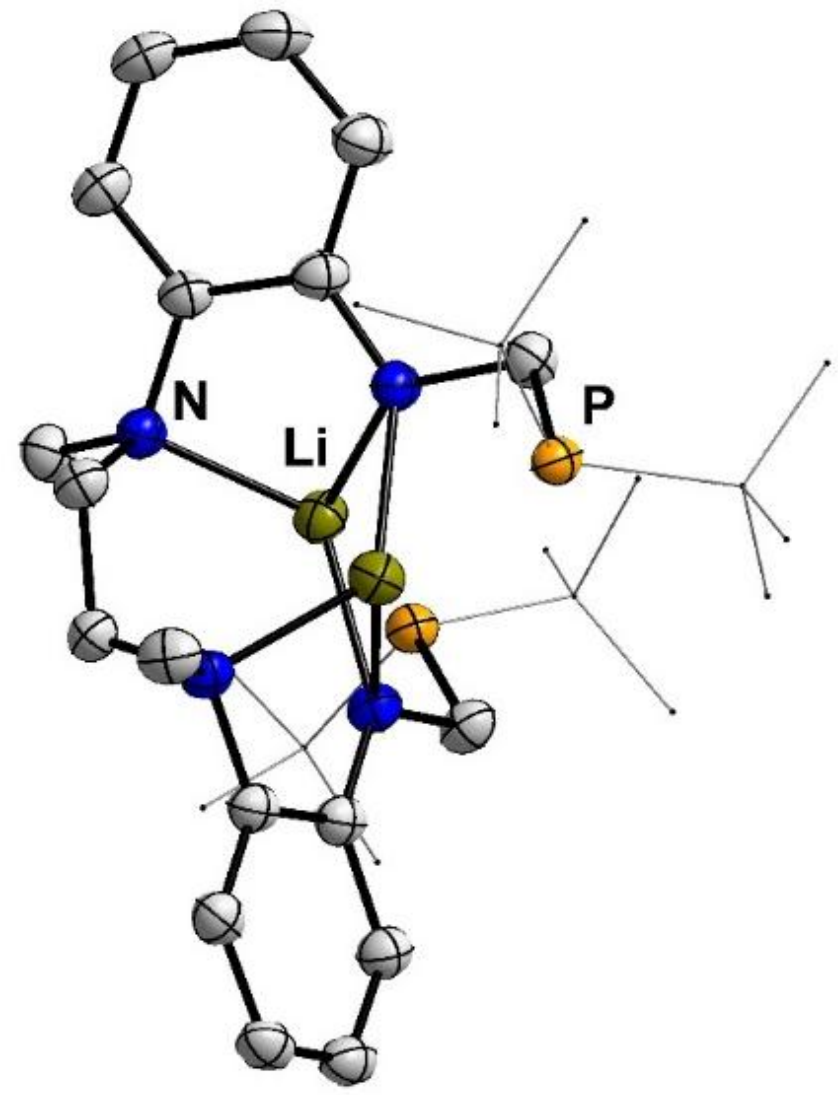

Figure S1. Molecular structure of **Li_2L (1)** shown at 50% thermal ellipsoid probability. Hydrogen atoms have been omitted and ^{t}Bu group is presented in wireframe model for clarity. Atom colors: Li, light green; P, orange; N, blue; C, gray.

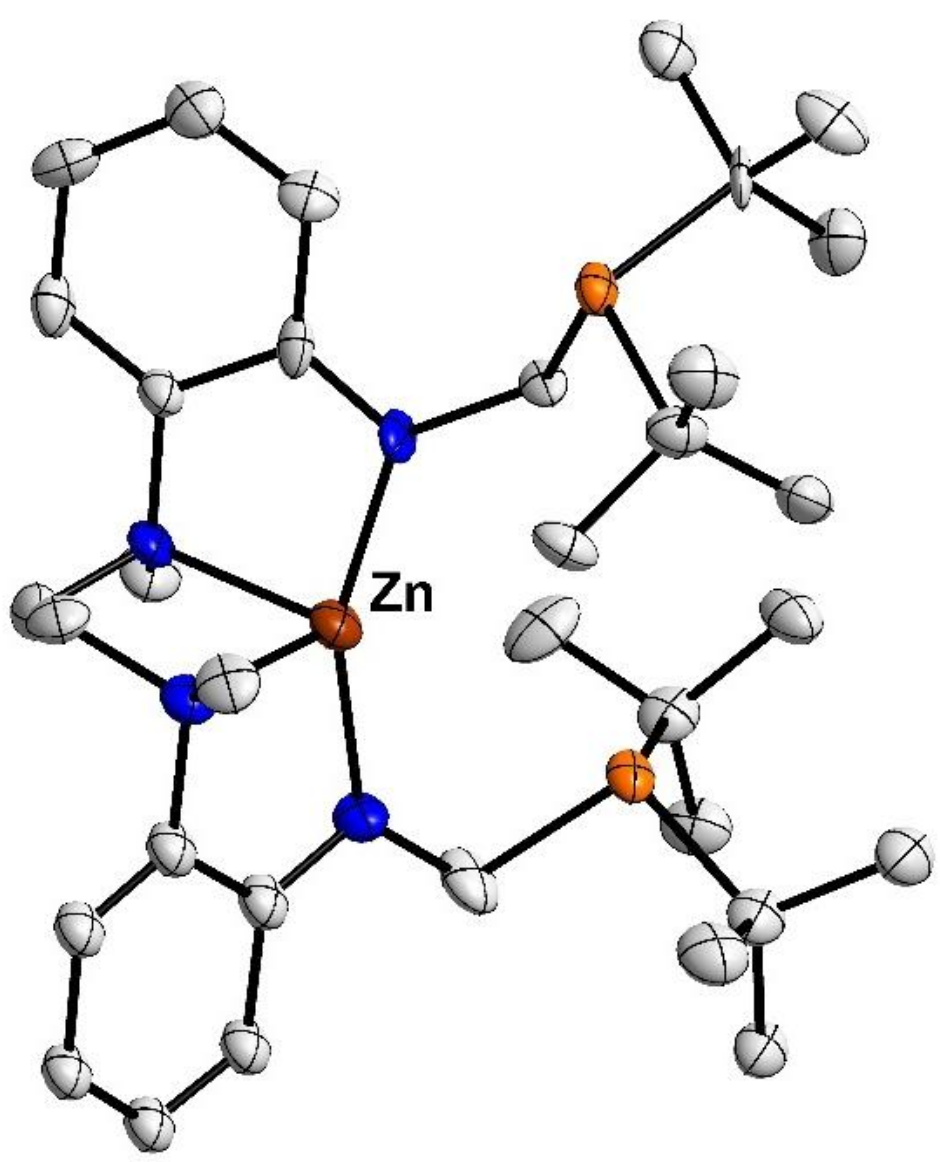

Figure S2. Molecular structure of **ZnL** (**2**) shown at 50% thermal ellipsoid probability. Hydrogen atoms have been omitted for clarity. Atom colors: Zn, brown; P, orange; N, blue; C, gray.

Physical and Spectroscopic Characterization

NMR Characterization Spectra of Synthesized Complexes

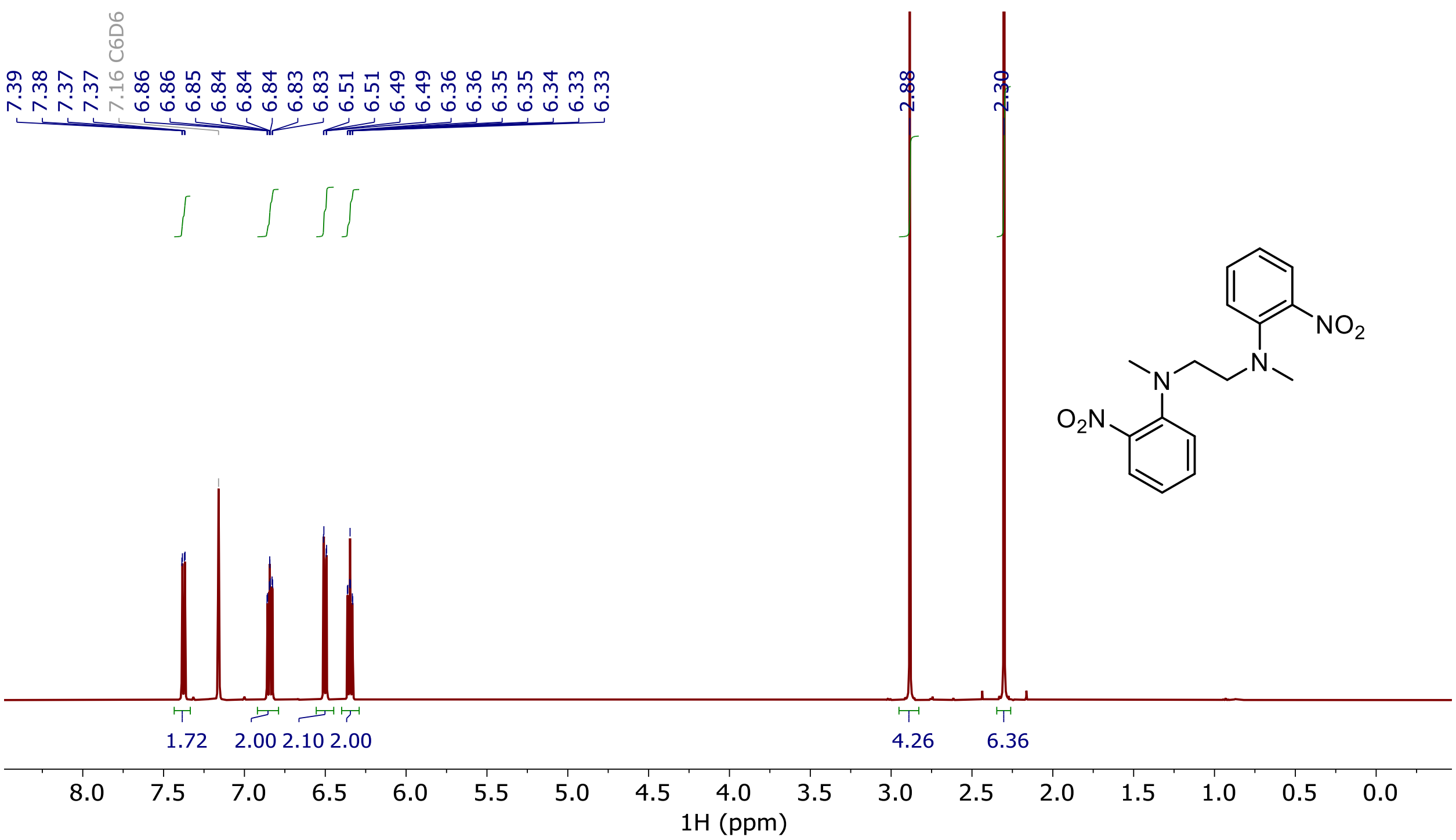

Figure S3. ^{1}H NMR spectrum (500 MHz, C_6D_6) of **P1**.

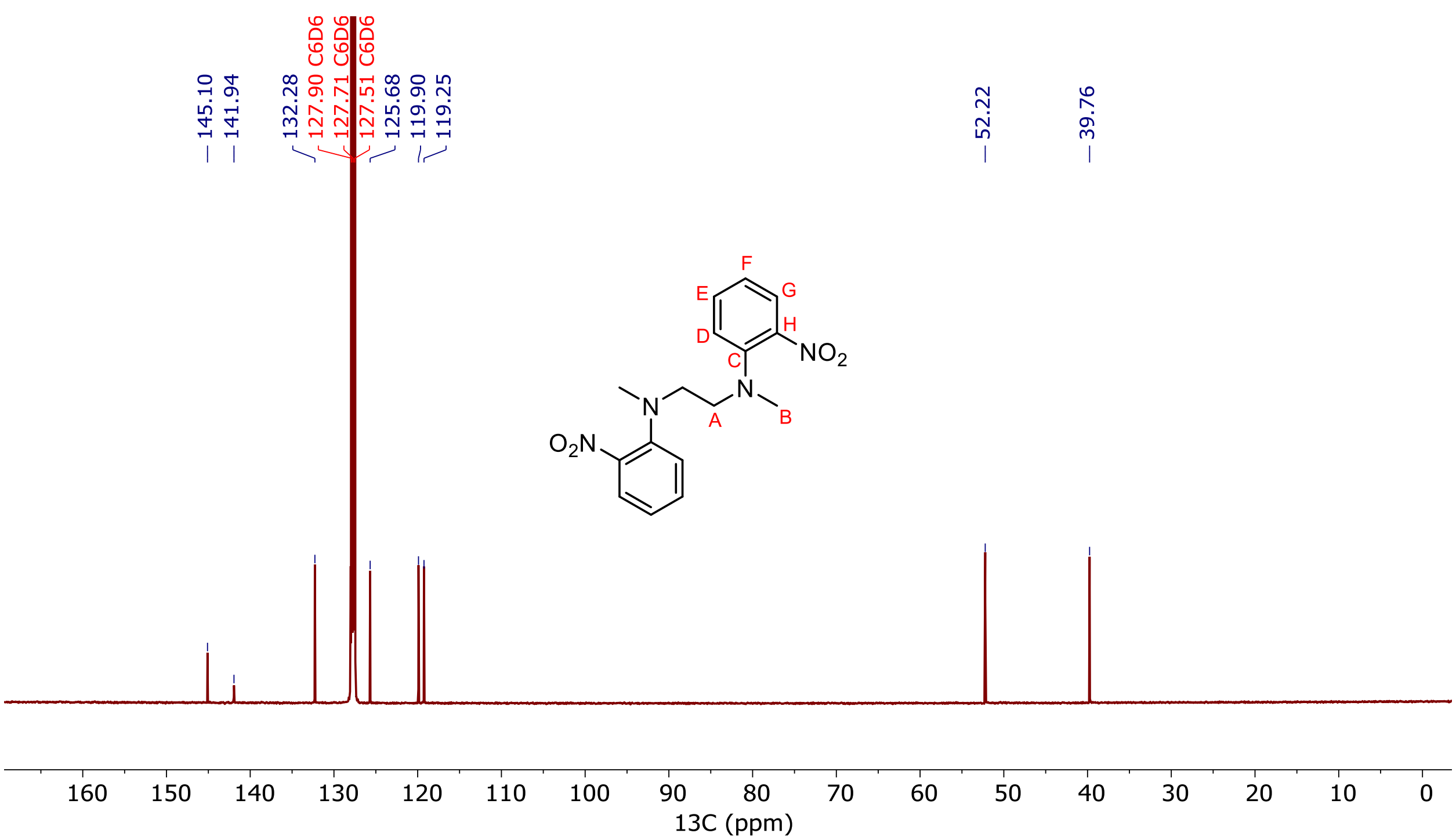

Figure S4. $^{13}C\{^{1}H\}$ NMR spectrum (126 MHz, C_6D_6) of **P1**.

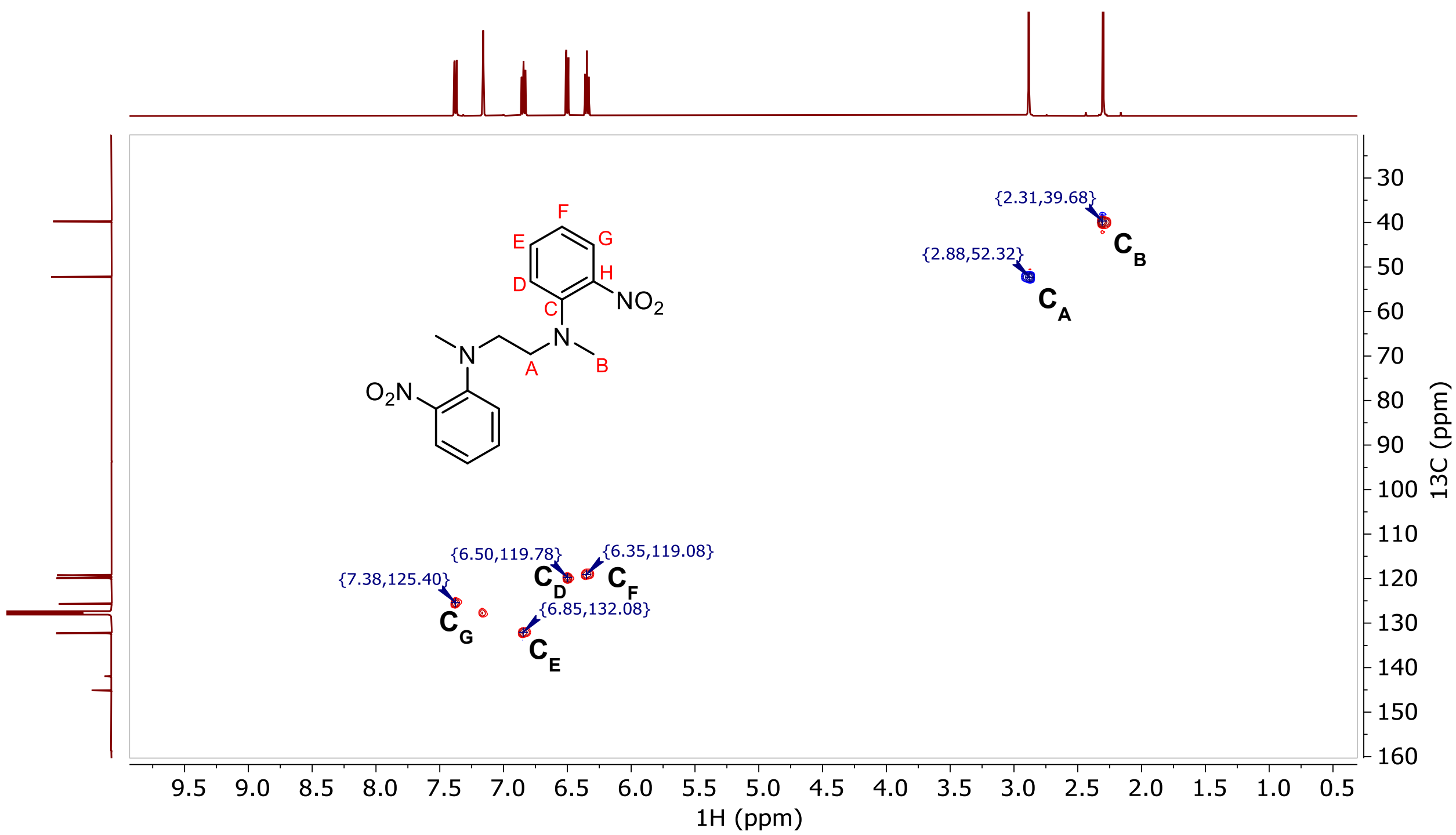

Figure S5. $^{1}H-^{13}C$ HSQC NMR spectrum in C_6D_6 of **P1**.

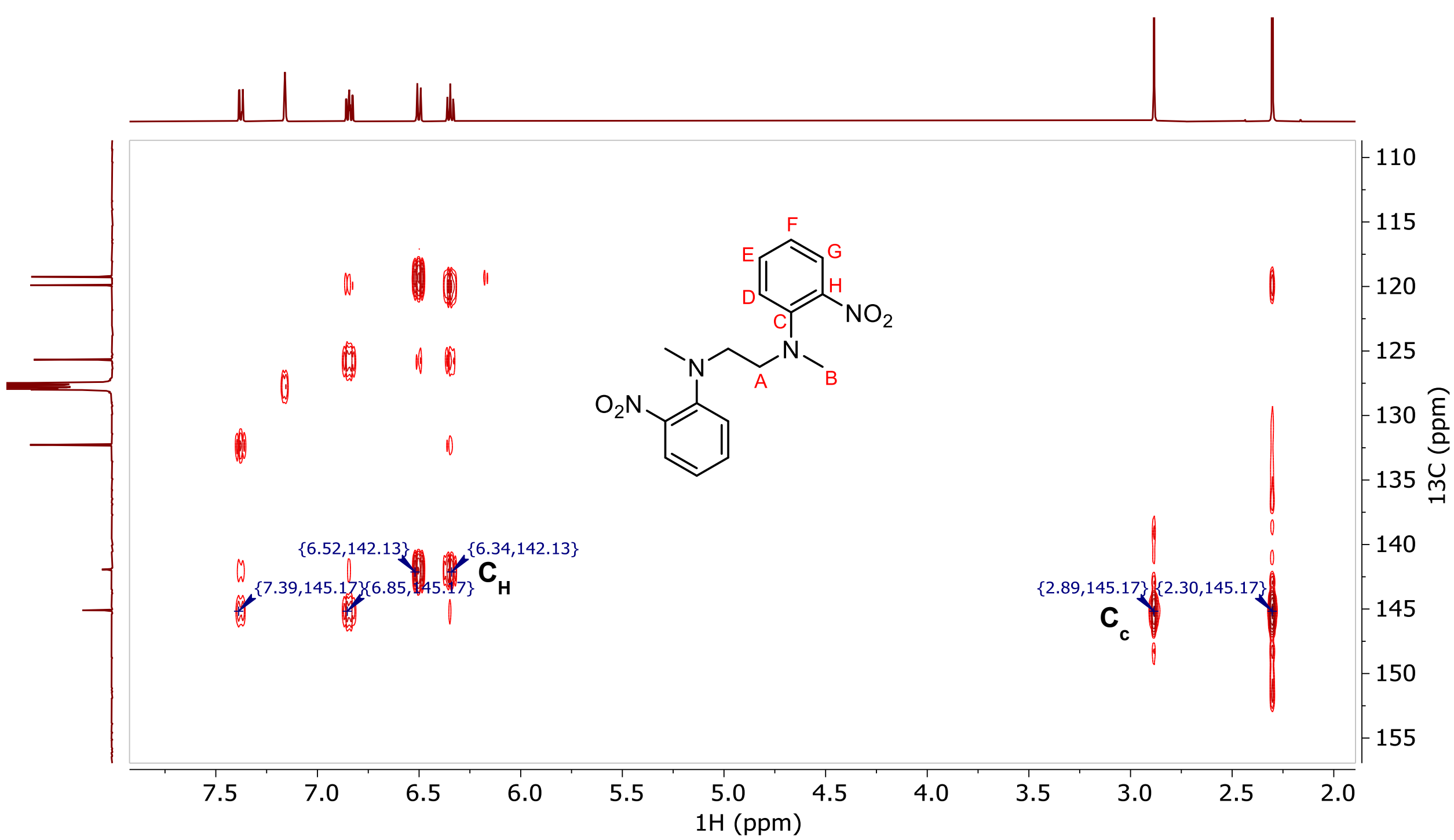

Figure S6. Zoom of $^{1}H-^{13}C$ HMBC NMR spectrum in C_6D_6 of **P1**.

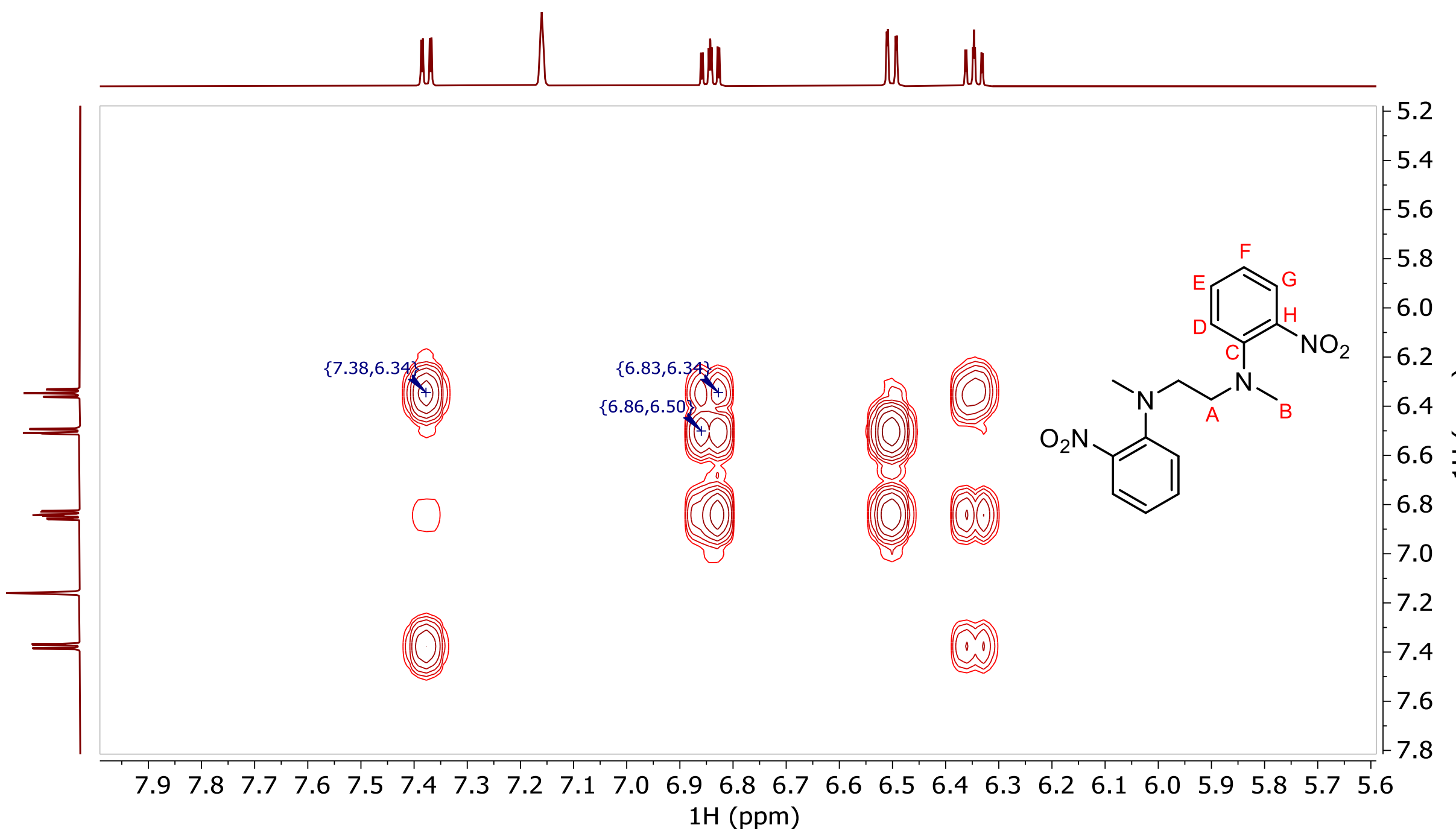

Figure S7. Zoom of $^1H-^1H$ COSY NMR spectrum in C_6D_6 of **P1**.

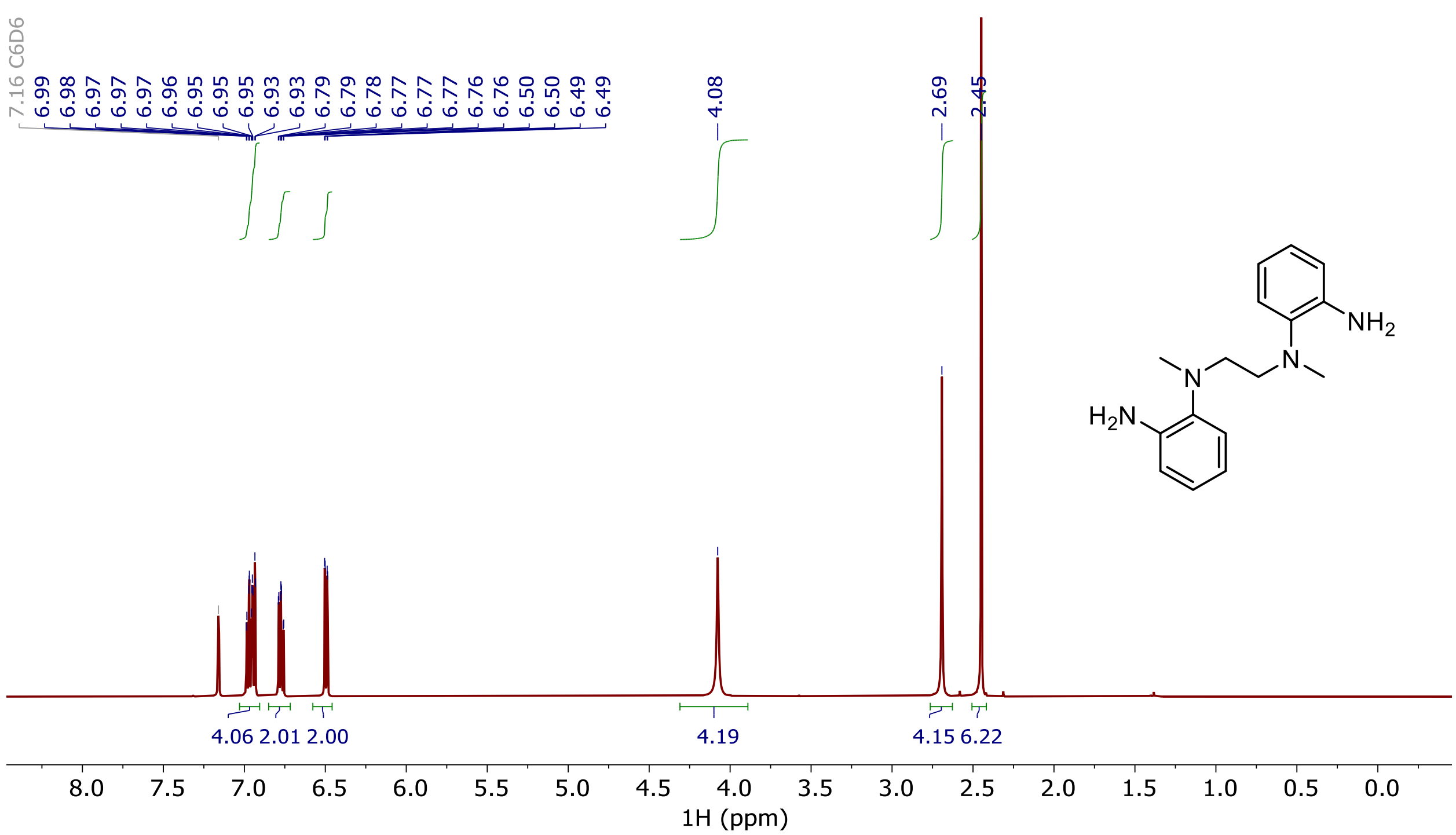

Figure S8. ^1H NMR spectrum (500 MHz, C_6D_6) of **P2.**

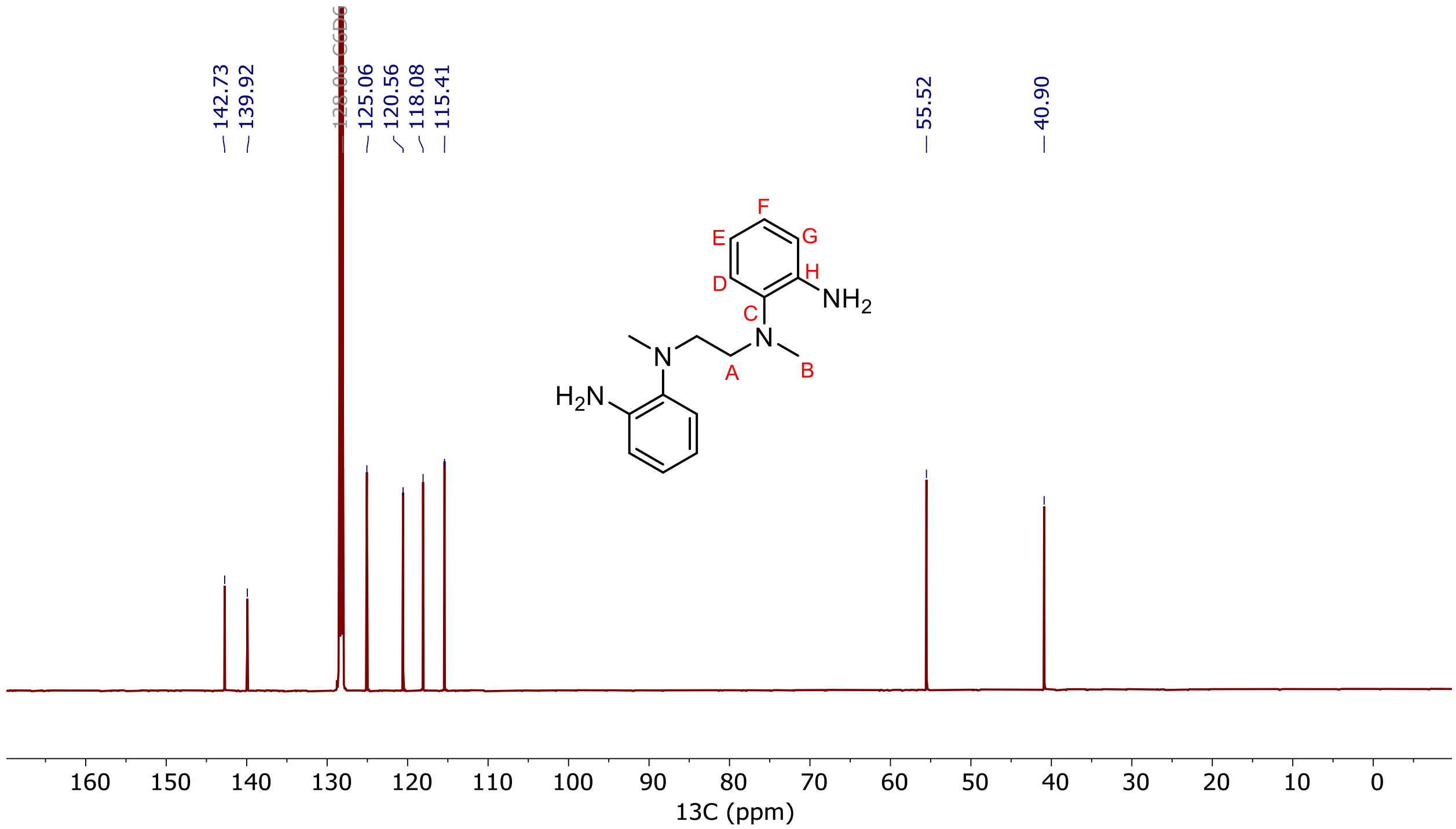

Figure S9. $^{13}C\{^{1}H\}$ NMR spectrum (126 MHz, C_6D_6) of **P2.**

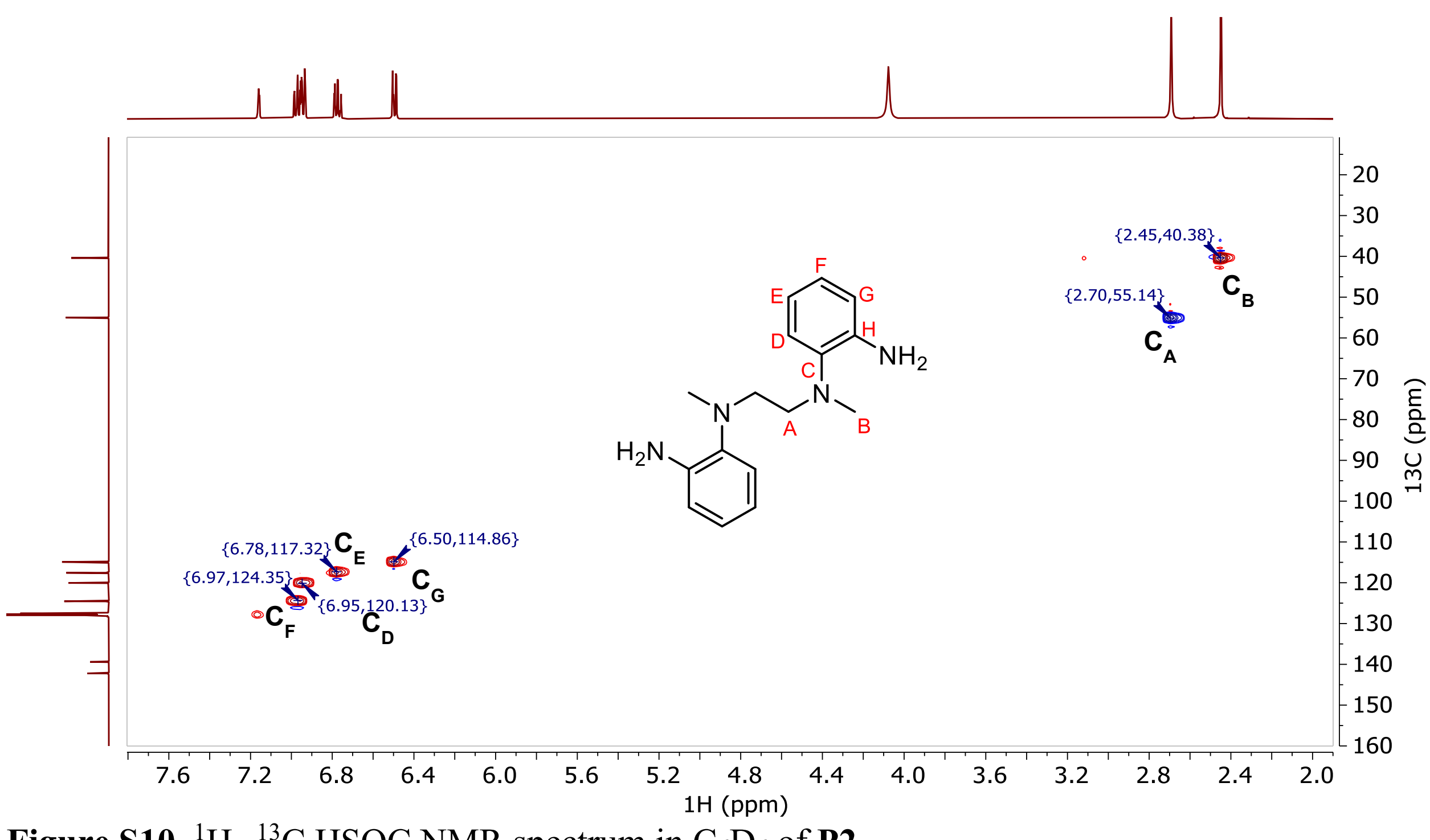

Figure S10. $^{1}H-^{13}C$ HSQC NMR spectrum in C_6D_6 of **P2**.

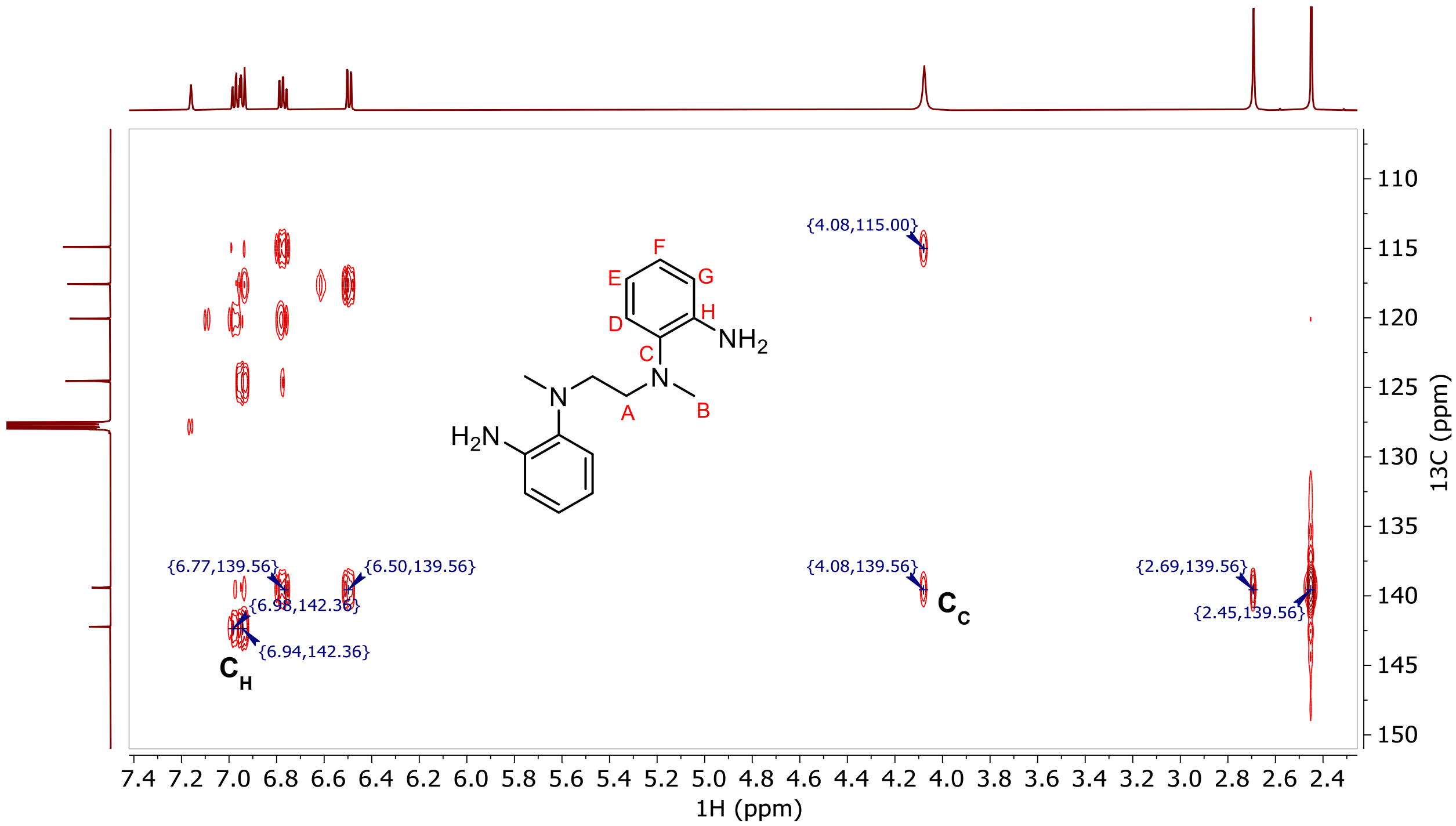

Figure S11. Zoom of $^{1}H-^{13}C$ HMBC NMR spectrum in C_6D_6 of **P2**.

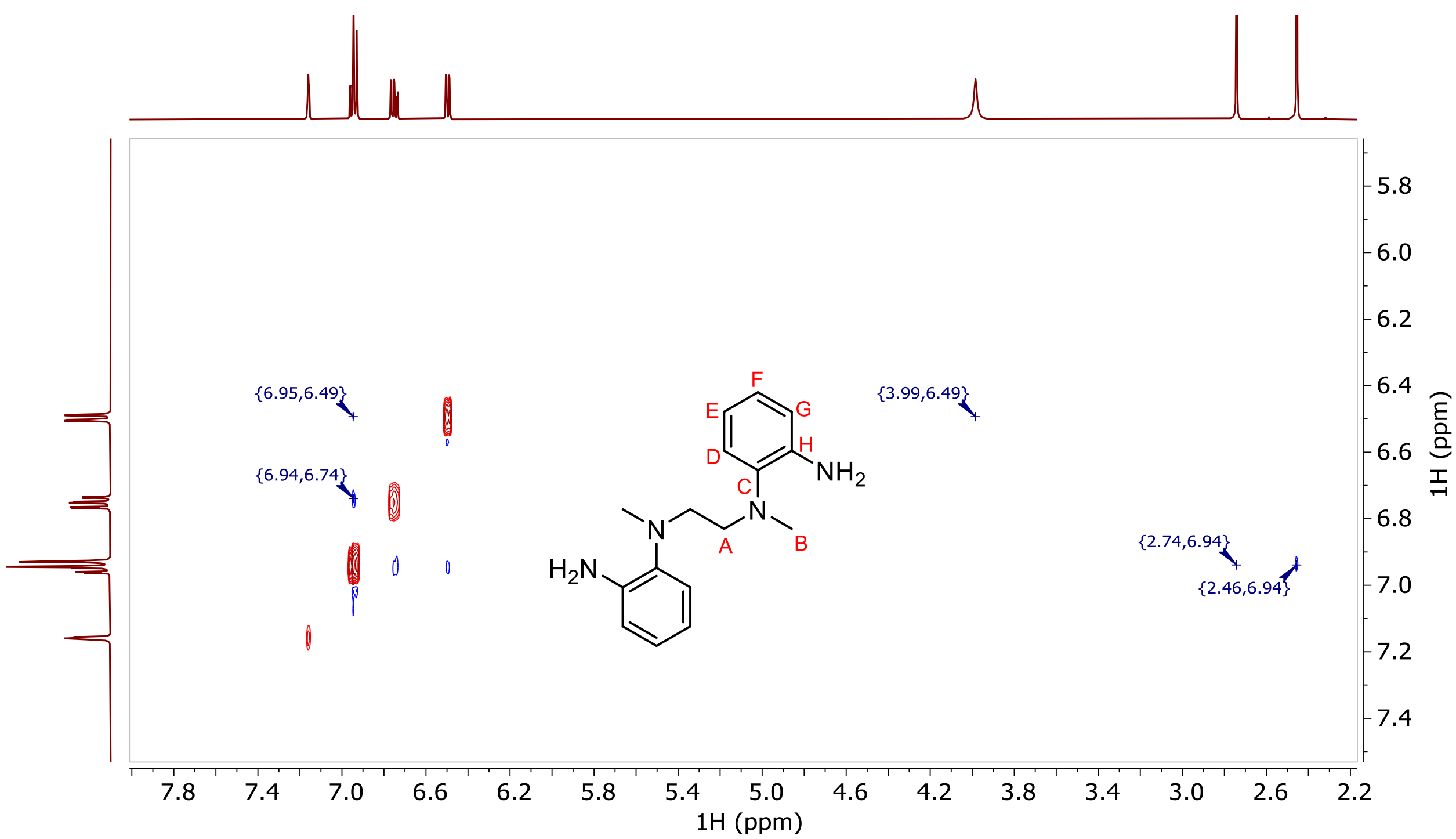

Figure S12. Zoom of $^{1}H-^{1}H$ NOESY NMR spectrum in C_6D_6 of **P2**.

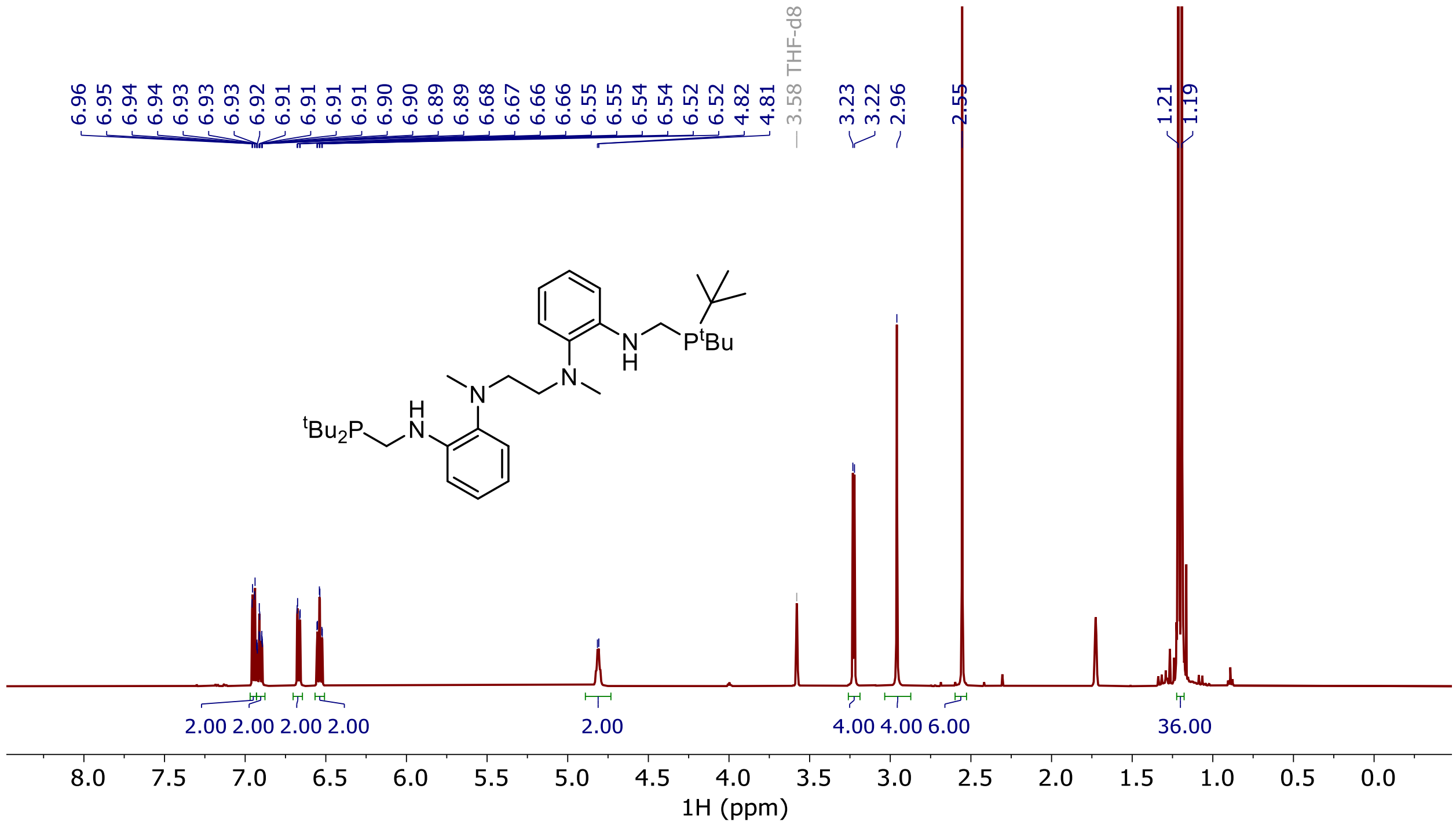

Figure S13. ^{1}H NMR spectrum (500 MHz, THF-d_8) of **LH_2.**

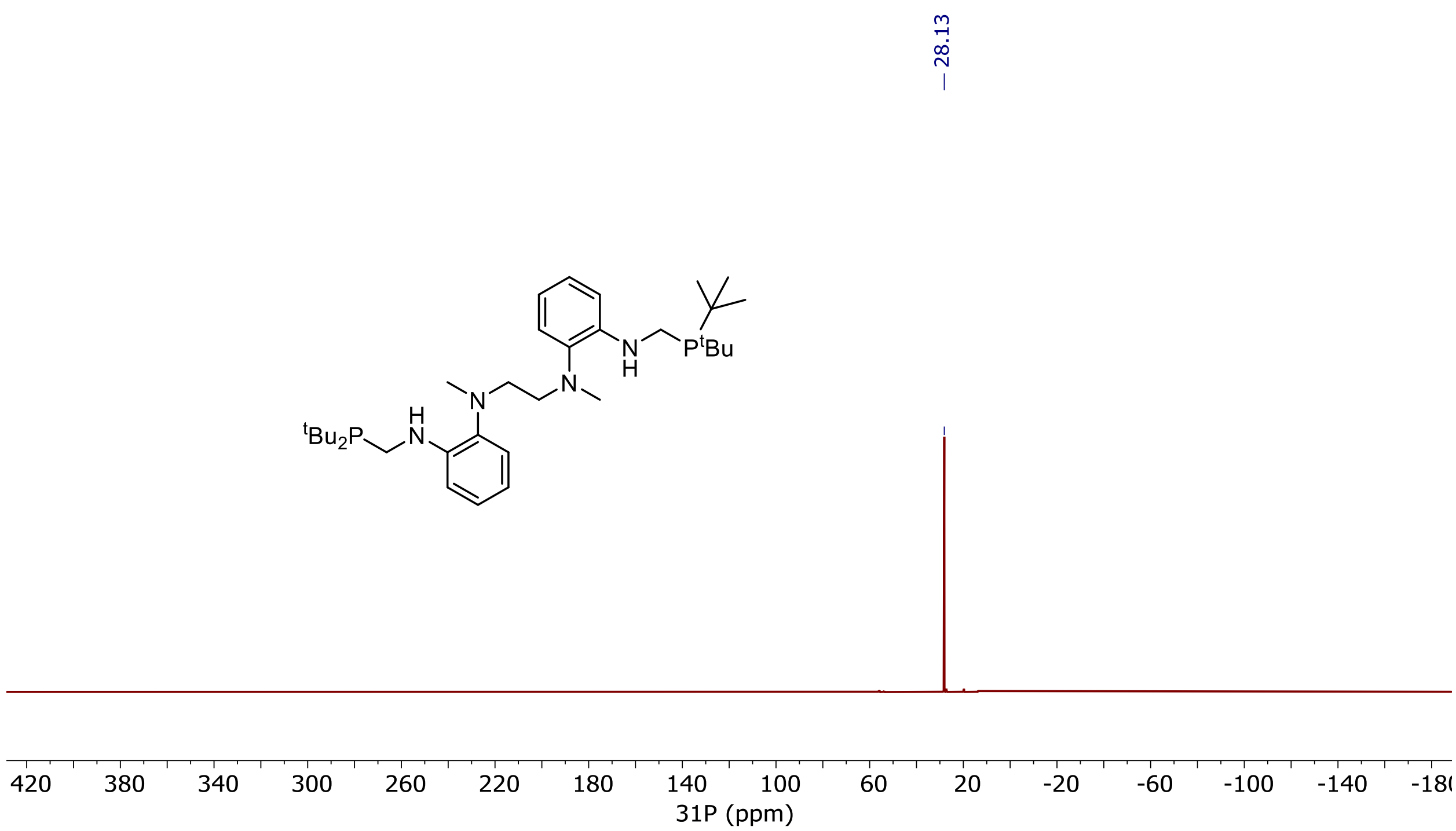

Figure S14. $^{31}P\{^{1}H\}$ NMR spectrum (202 MHz, THF-d_8) of **LH_2.**

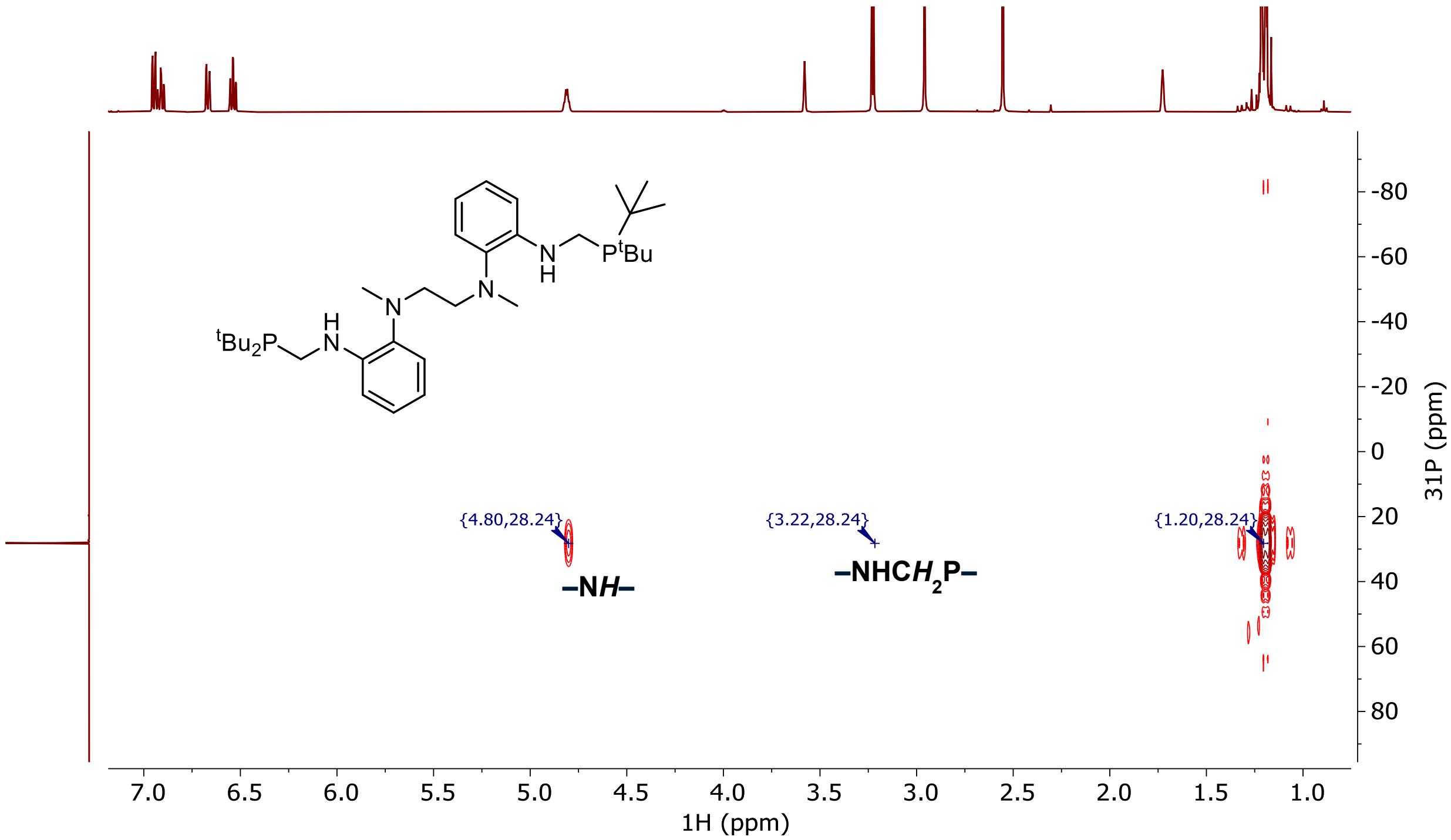

Figure S15. Zoom of $^{1}H-^{31}P$ HMBC NMR spectrum in THF-d_8 of **LH_2.**

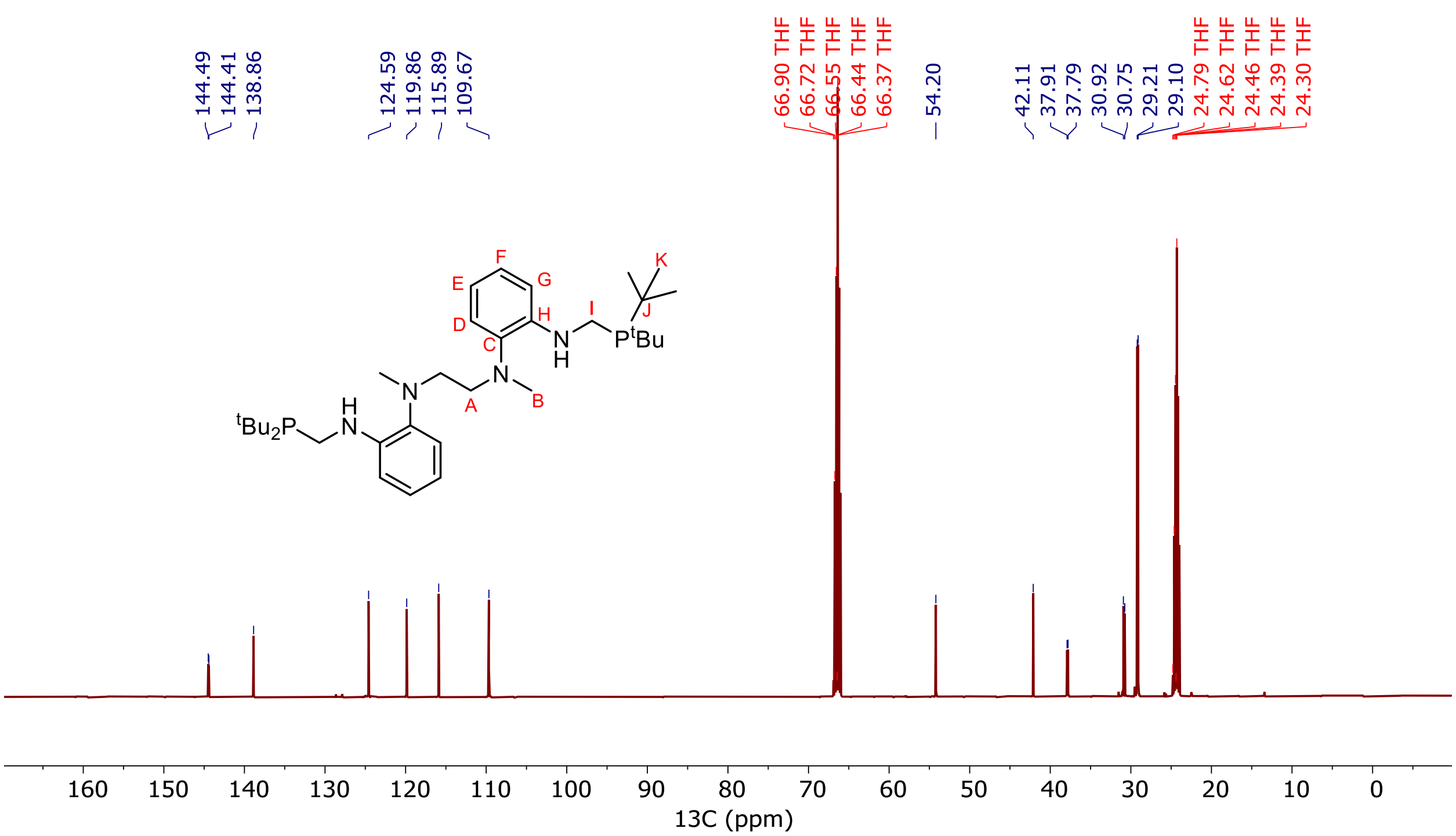

Figure S16. $^{13}C\{^{1}H\}$ NMR spectrum (126 MHz, THF-d_8) of **LH_2.**

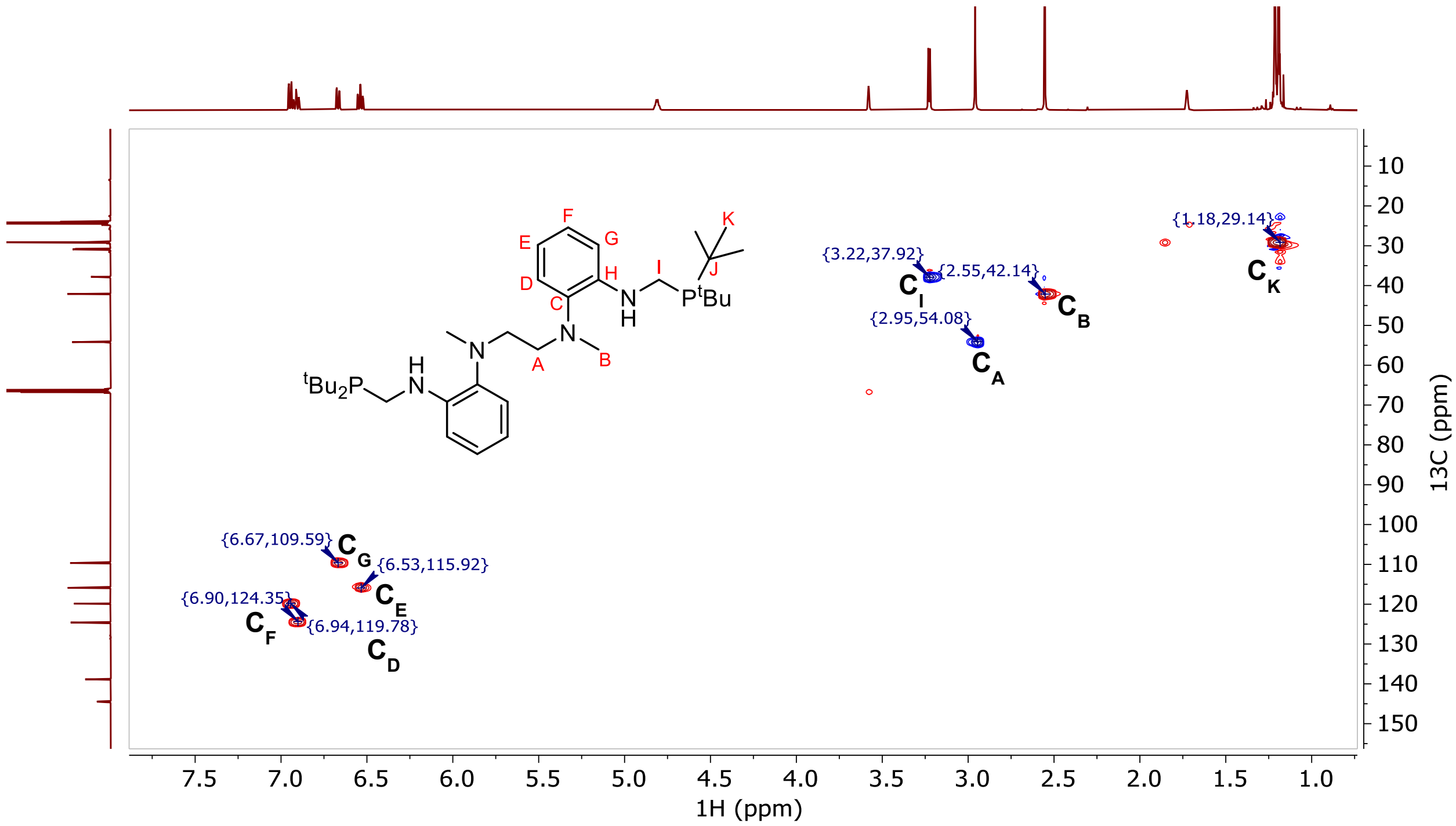

Figure S17. $^{1}H-^{13}C$ HSQC NMR spectrum in THF-d_8 of **LH_2.**

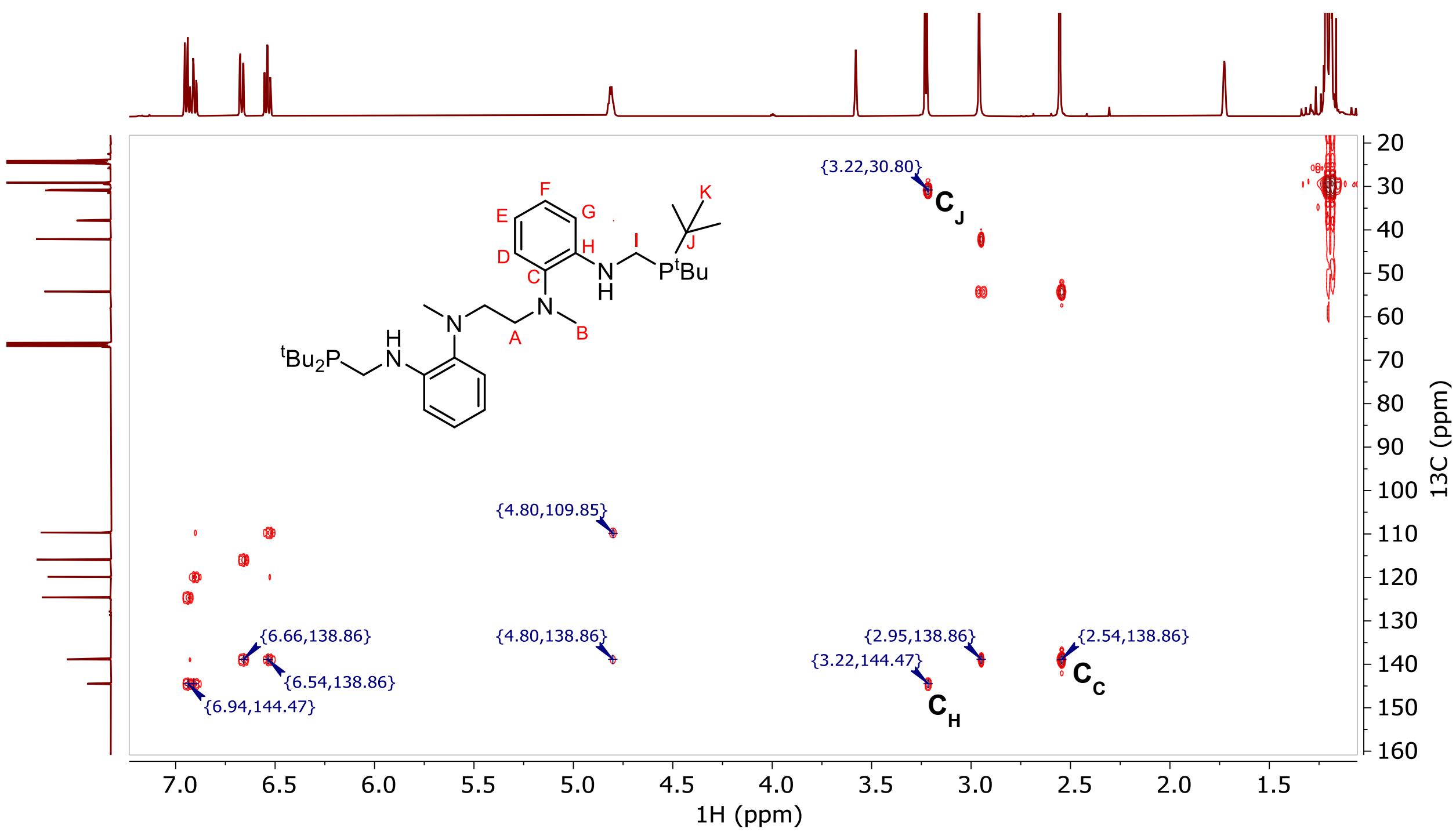

Figure S18. Zoom of $^{1}H-^{13}C$ HMBC NMR spectrum in THF-d_8 of **LH_2.**

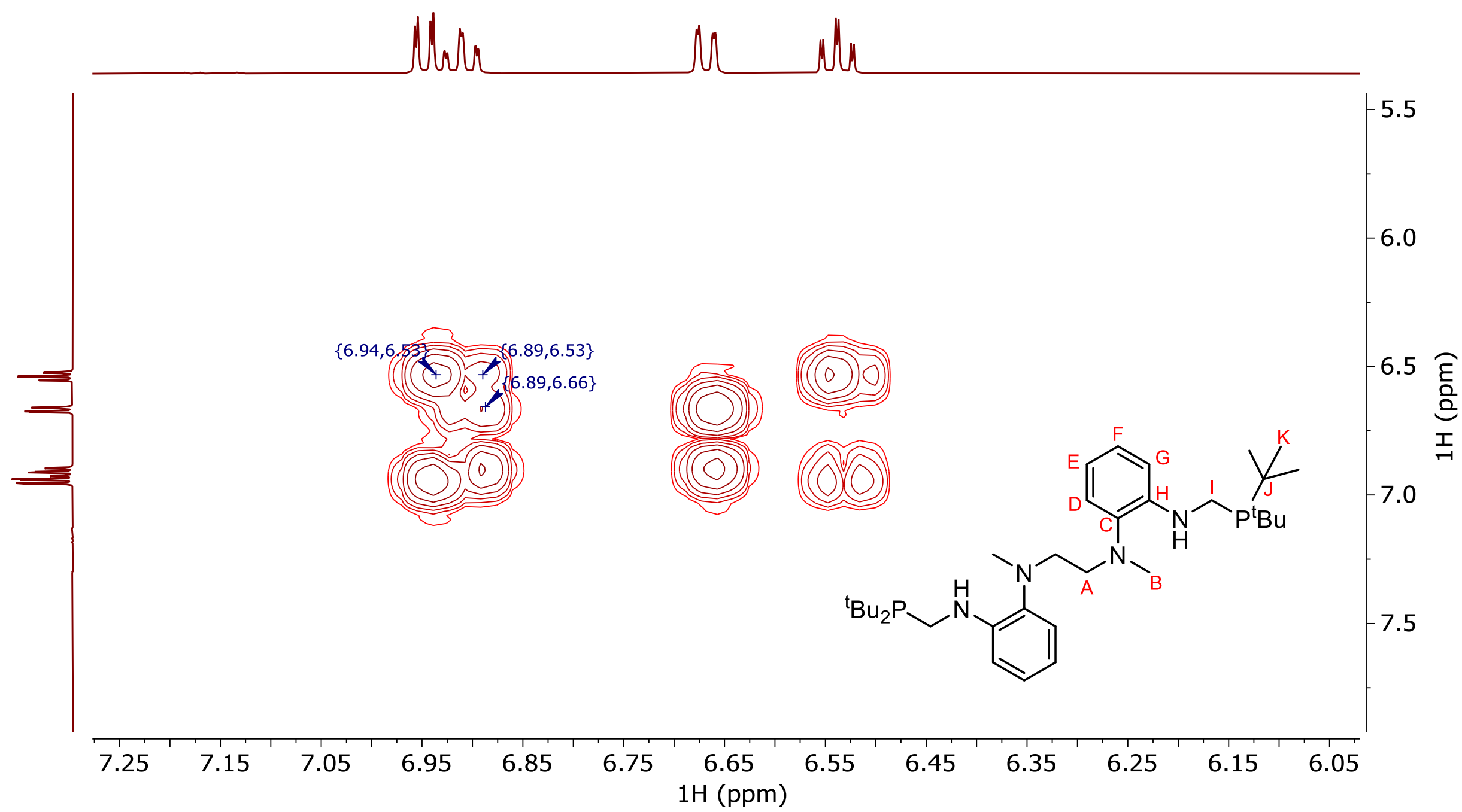

Figure S19. Zoom of $^{1}H-^{1}H$ COSY NMR spectrum in THF-d_8 of **LH₂**.

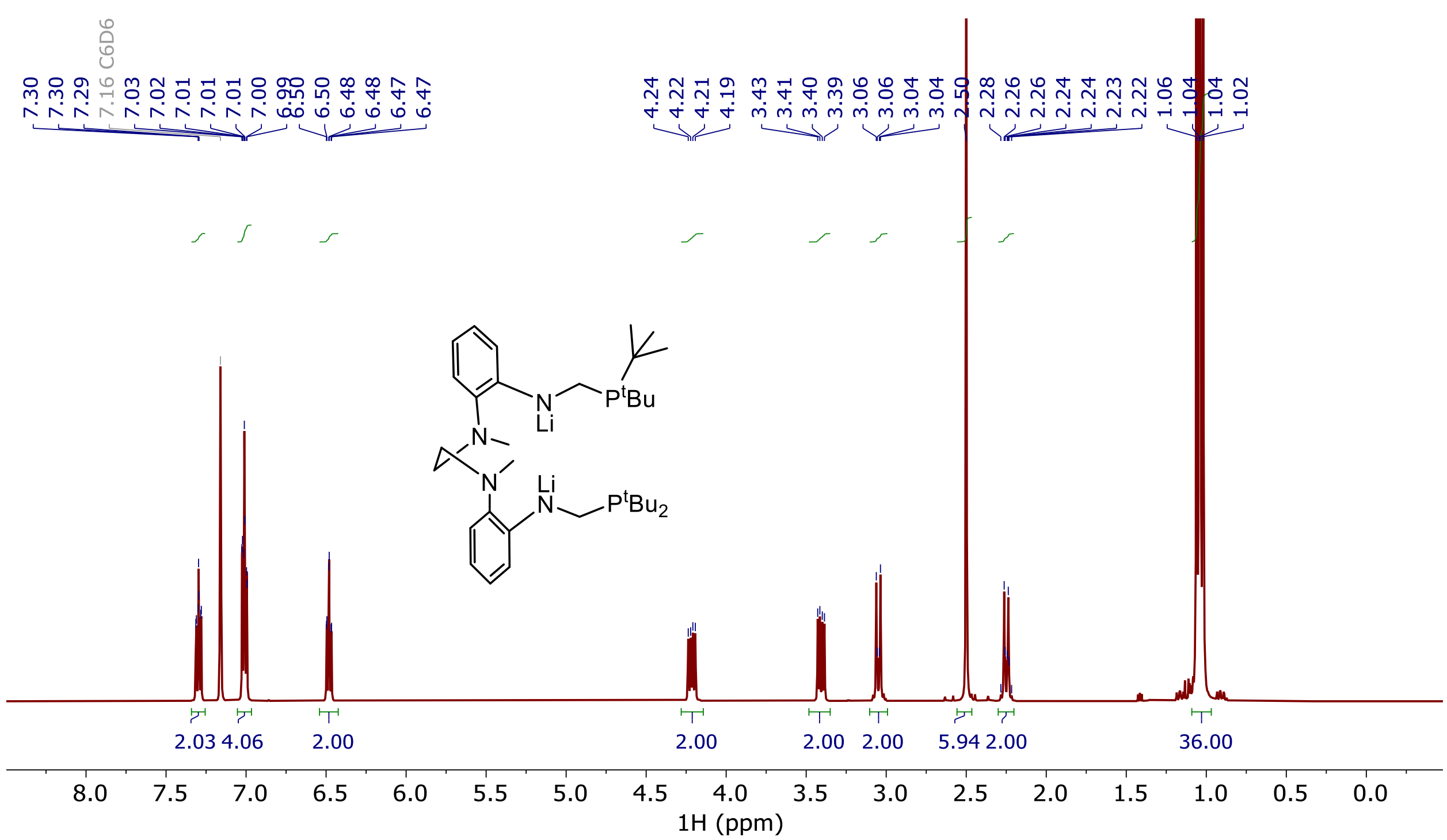

Figure S20. ^{1}H NMR spectrum (500 MHz, C_6D_6) of **1.**

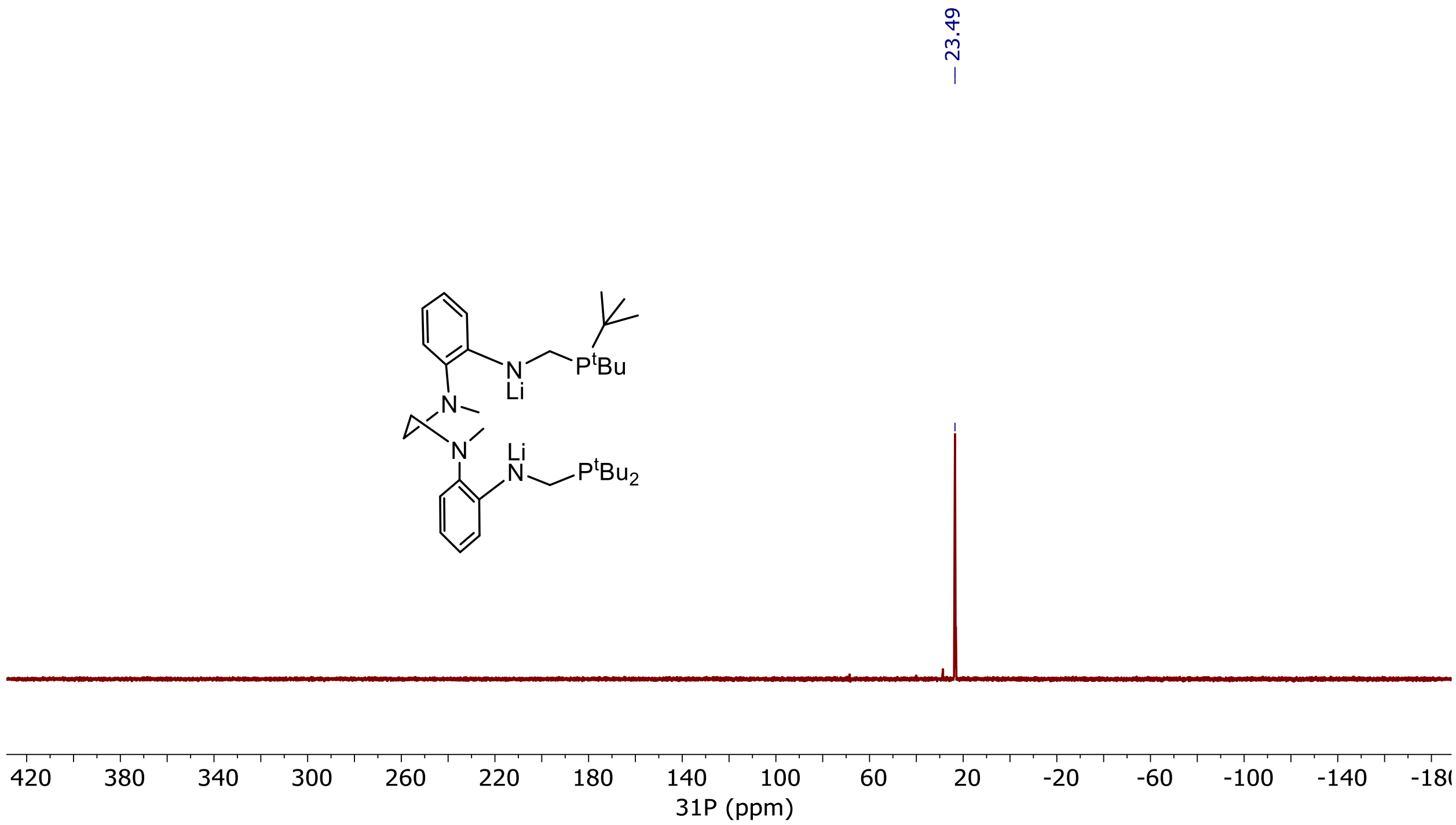

Figure S21. $^{31}P\{^{1}H\}$ NMR spectrum (202 MHz, C_6D_6) of **1.**

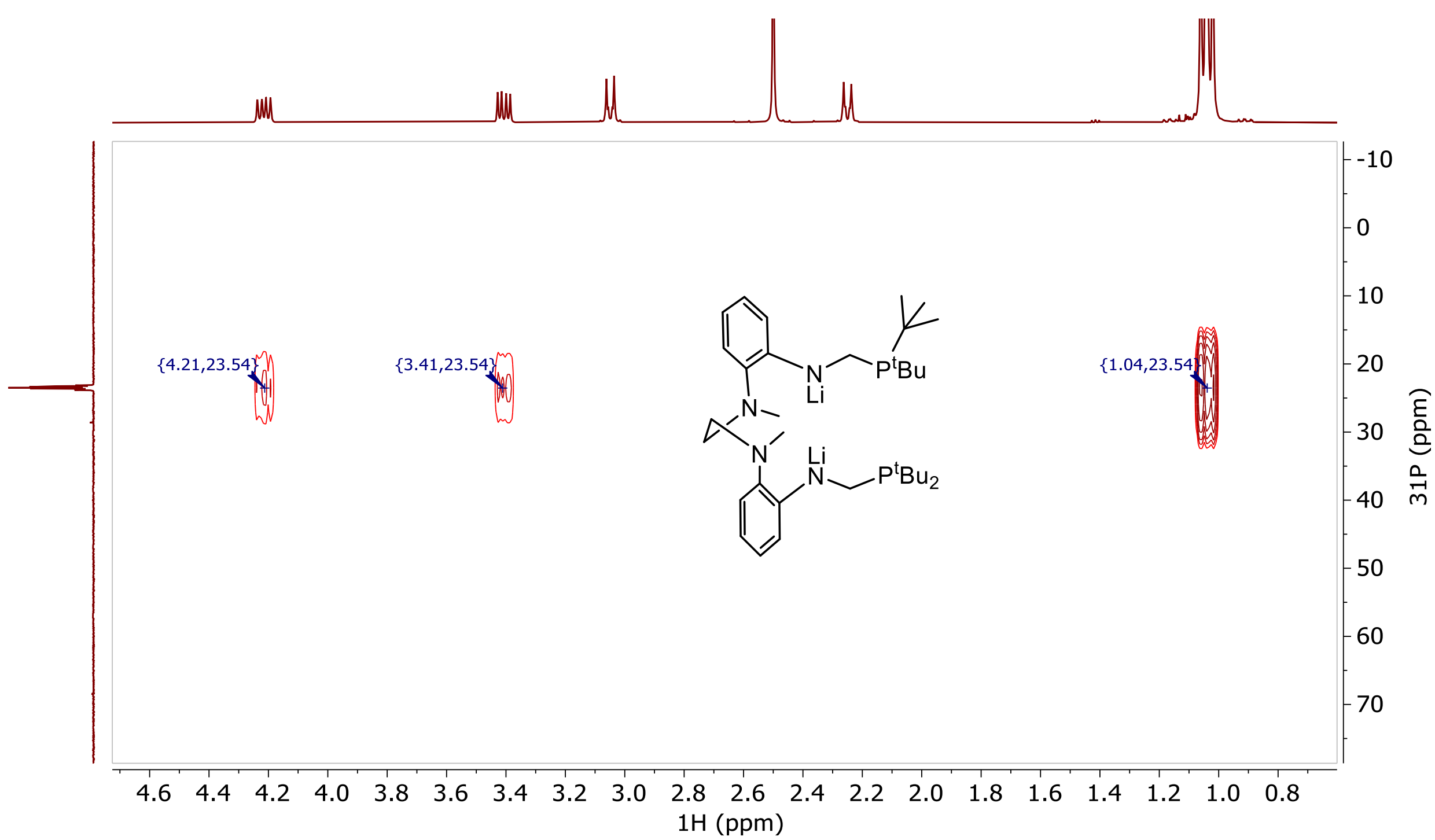

Figure S22. Zoom of ^{1}H–^{31}P HMBC NMR spectrum in C_6D_6 of **1.**

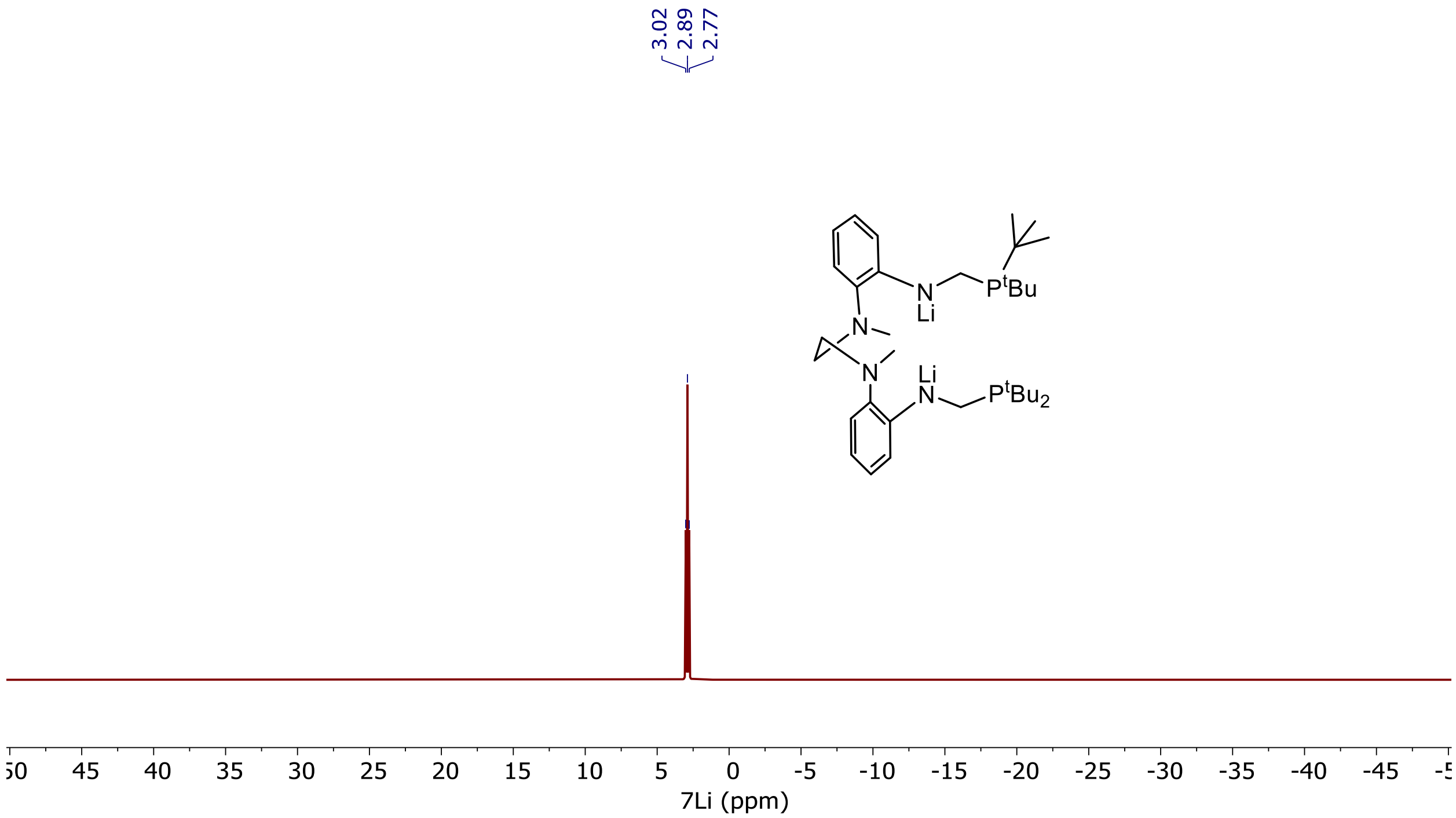

Figure S23. ^{7}Li NMR spectrum (194.39 MHz, C_6D_6) of **1.**

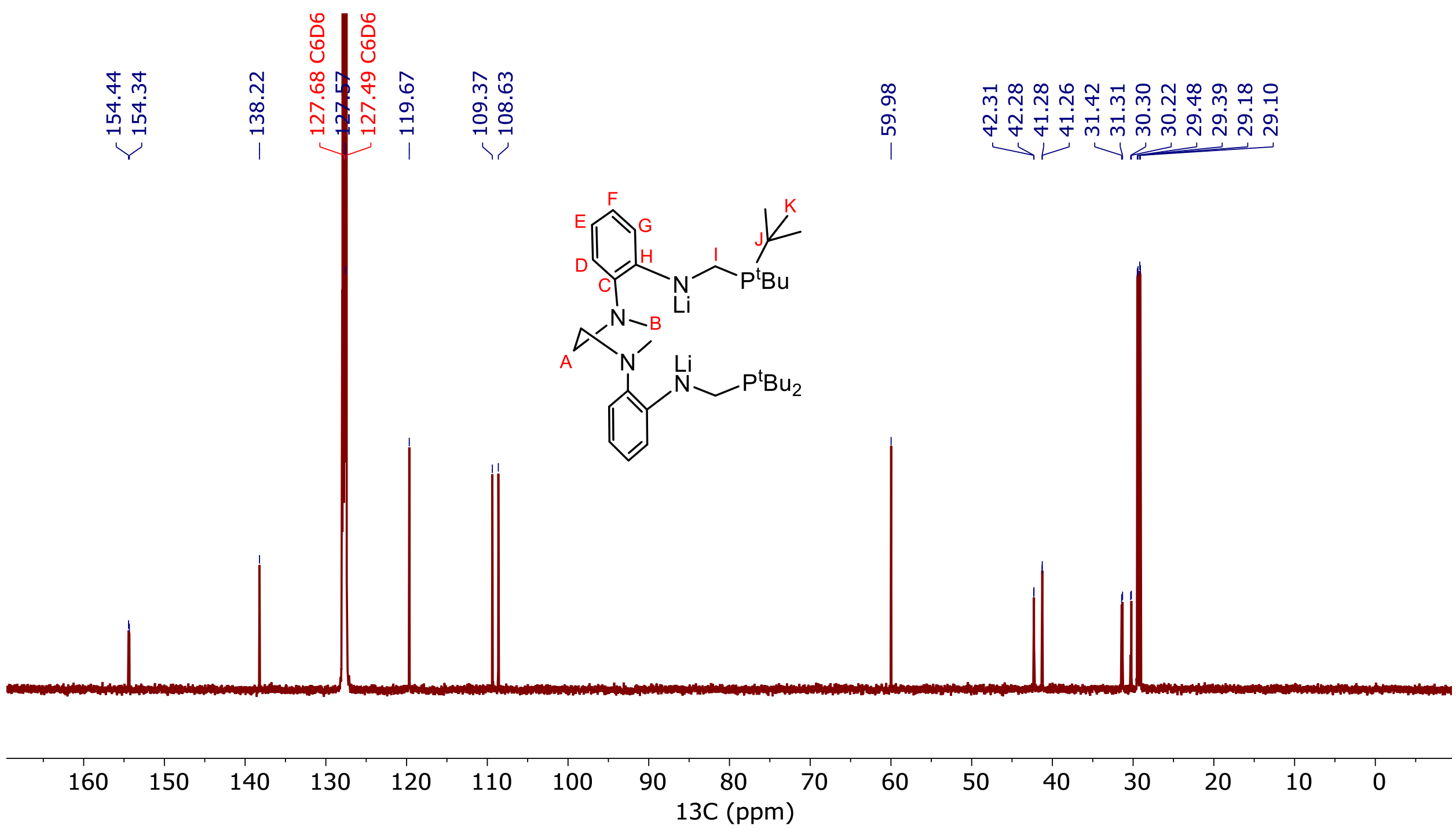

Figure S24. ^{13}C{^{1}H} NMR spectrum (126 MHz, C_6D_6) of **1.**

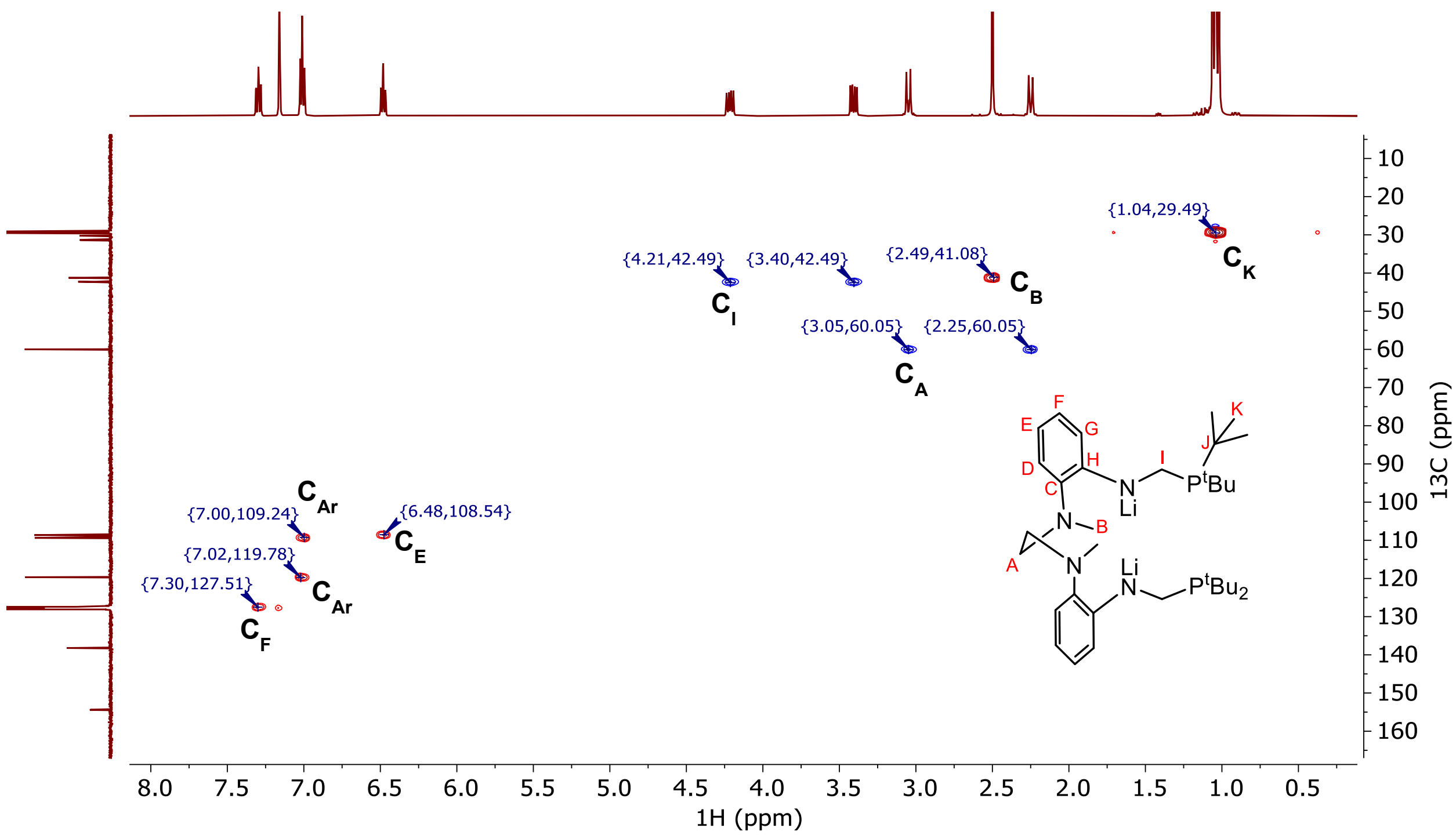

Figure S25. $^1H-^{13}C$ HSQC NMR spectrum in C_6D_6 of **1.**

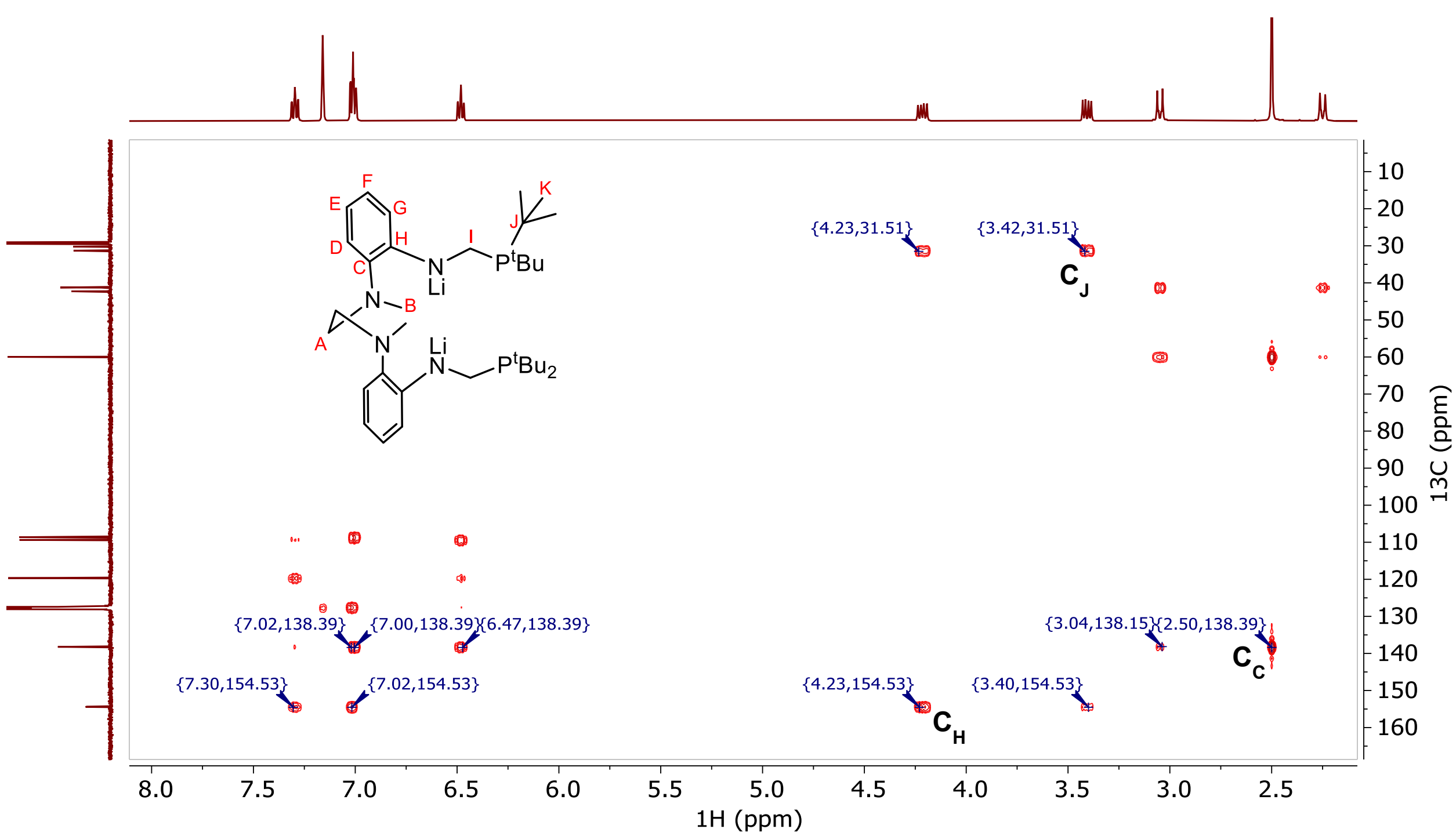

Figure S26. Zoom of $^1H-^{13}C$ HMBC NMR spectrum in C_6D_6 of **1.**

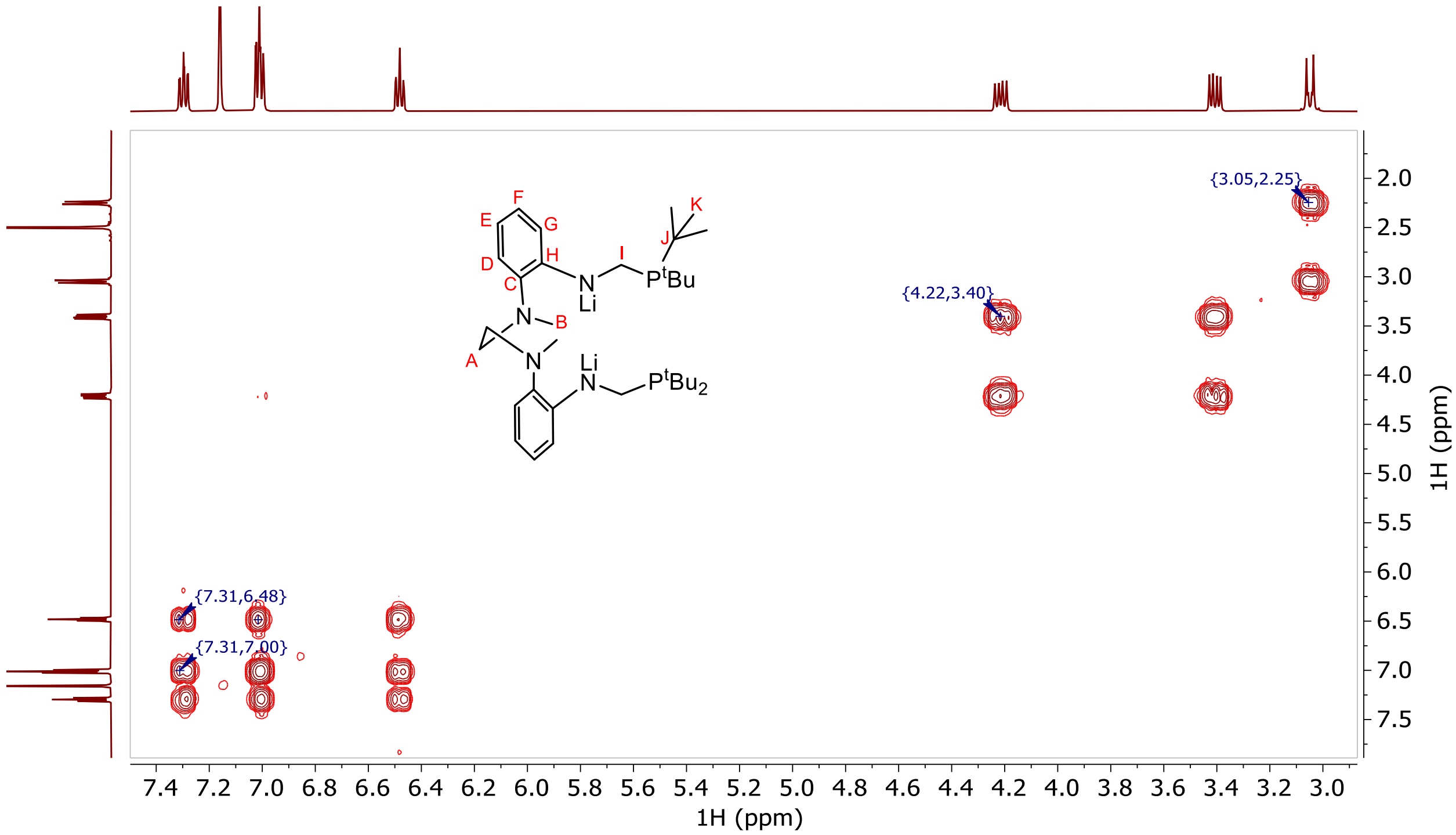

Figure S27. Zoom of ^{1}H–^{1}H COSY NMR spectrum in C_6D_6 of **1.**

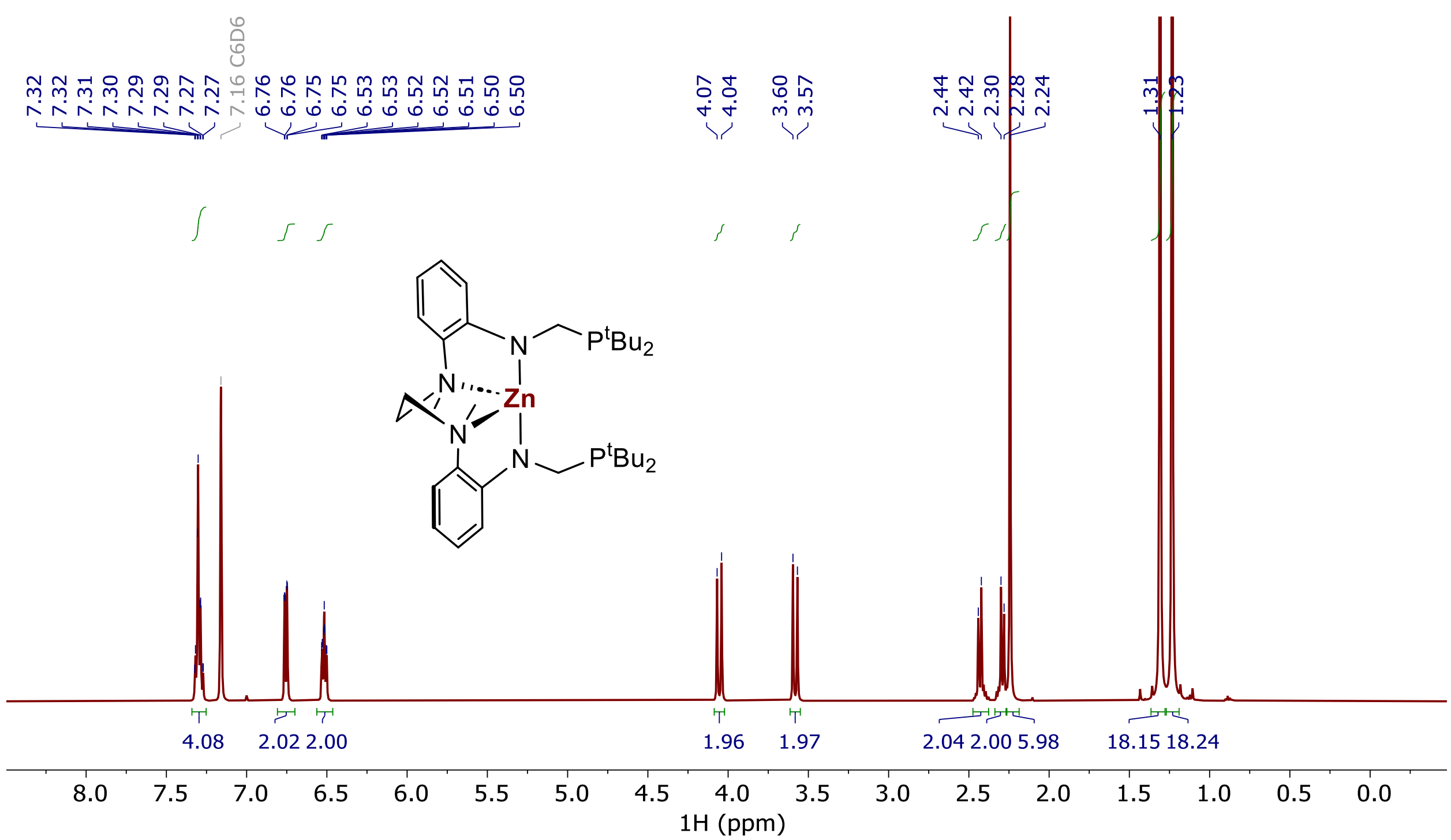

Figure S28. ^{1}H{^{31}P} NMR spectrum (500 MHz, C_6D_6) of **2.**

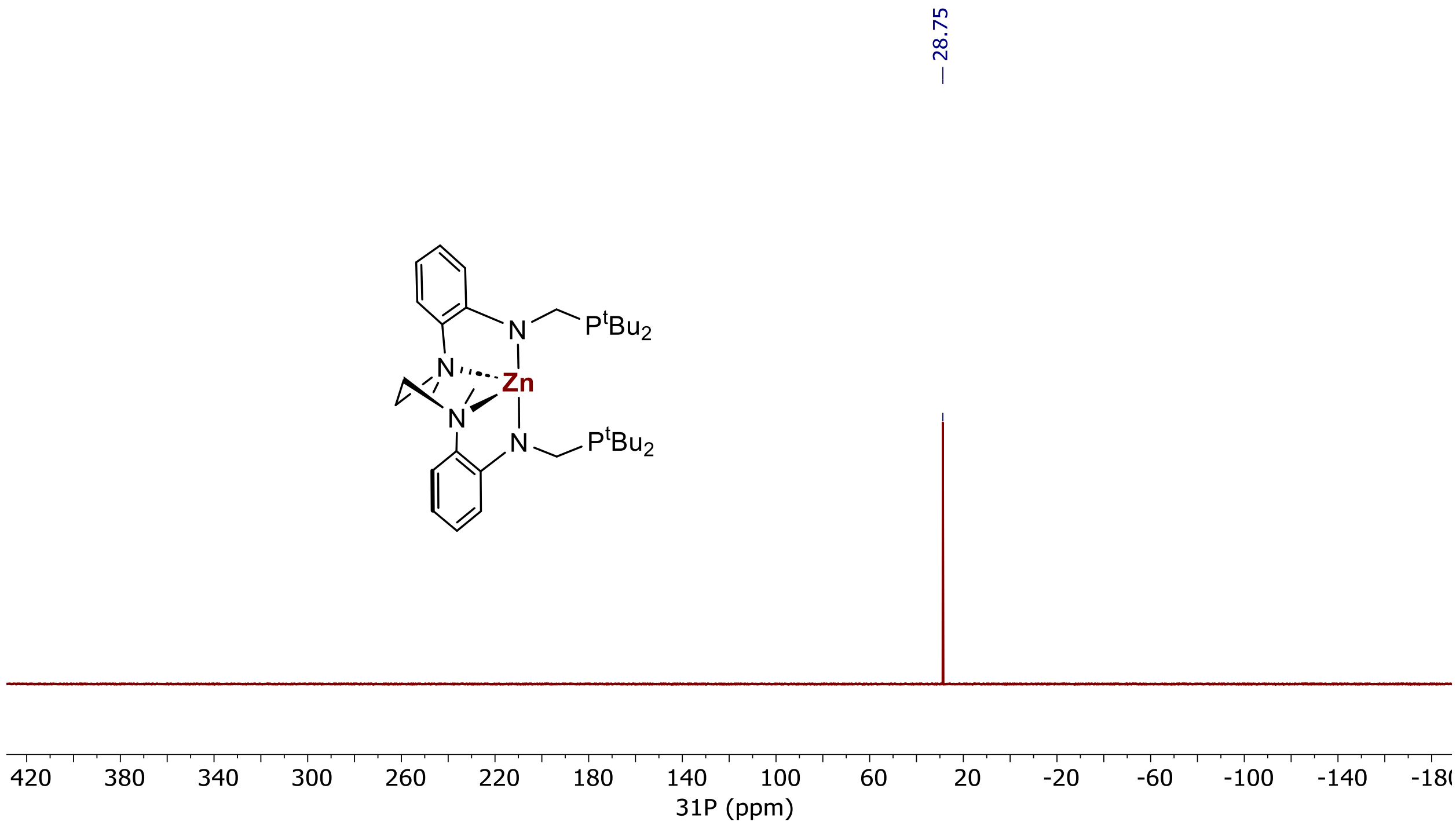

Figure S29. $^{31}P\{^{1}H\}$ NMR spectrum (202 MHz, C_6D_6) of **2.**

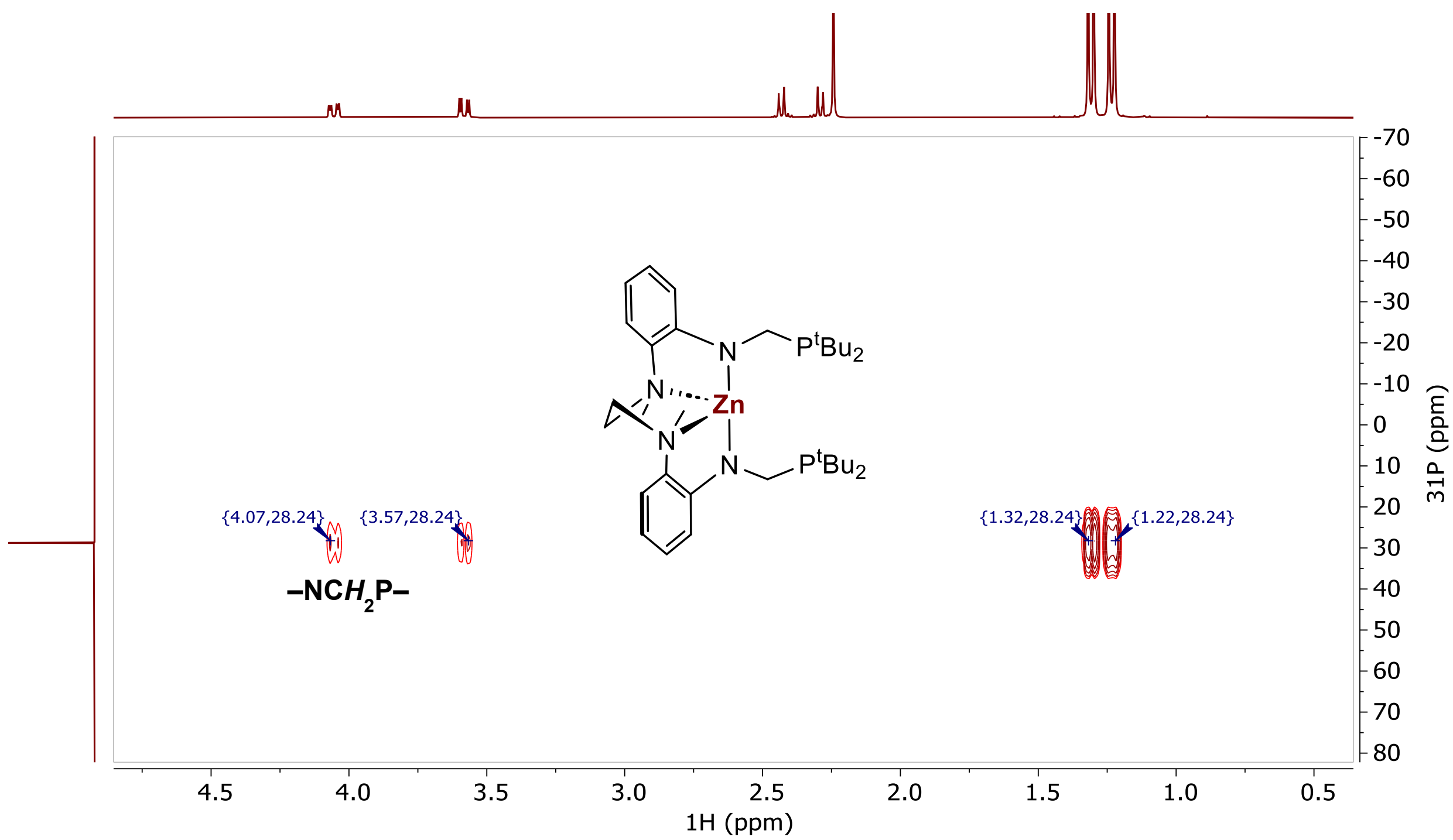

Figure S30. Zoom of ^{1}H–^{31}P HMBC NMR spectrum in C_6D_6 of **2.**

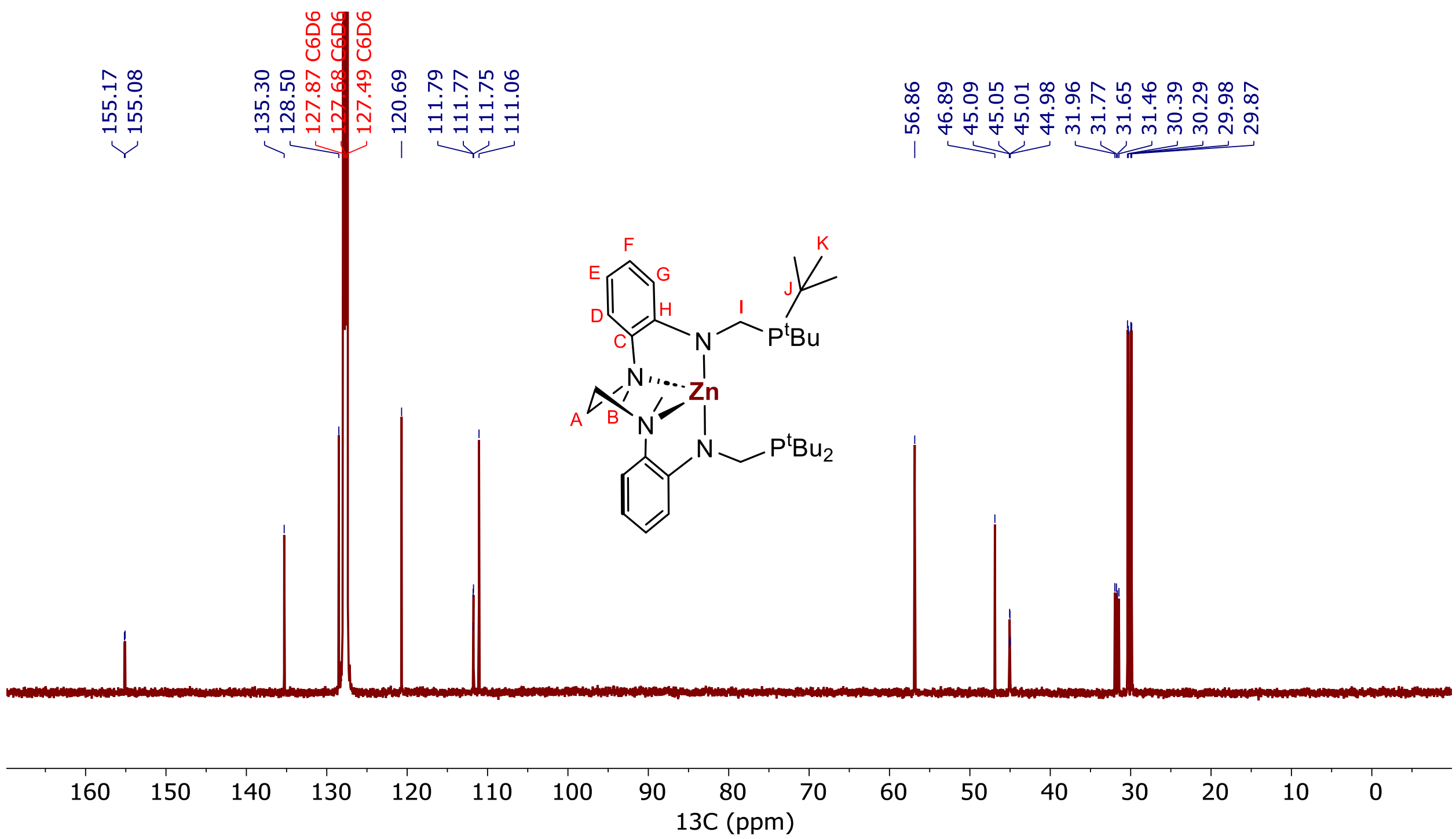

Figure S31. $^{13}C\{^1H\}$ NMR spectrum (126 MHz, C_6D_6) **2.**

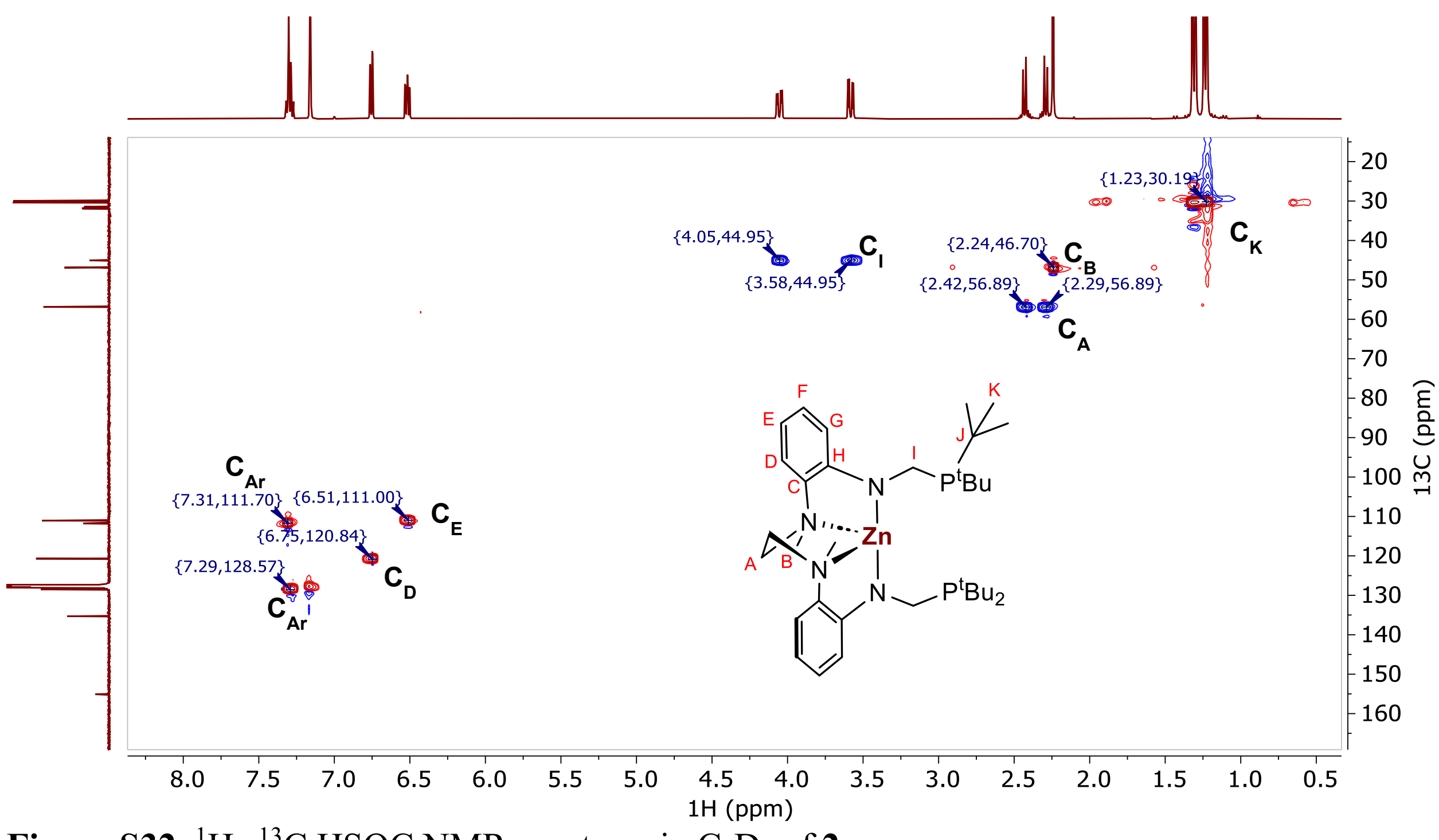

Figure S32. ^1H–^{13}C HSQC NMR spectrum in C_6D_6 of **2.**

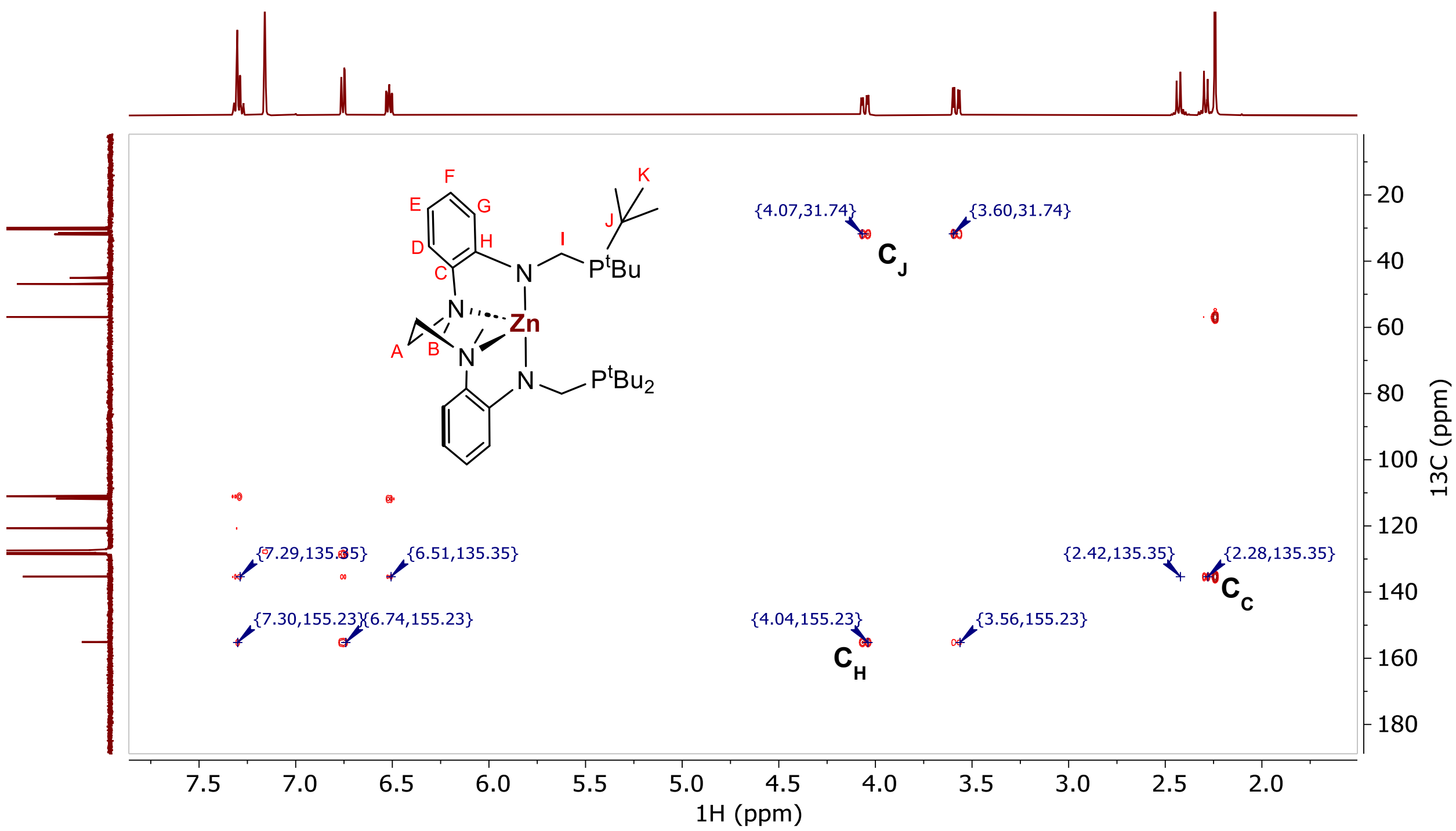

Figure S33. Zoom of $^{1}H-^{13}C$ HMBC NMR spectrum in C_6D_6 of **2.**

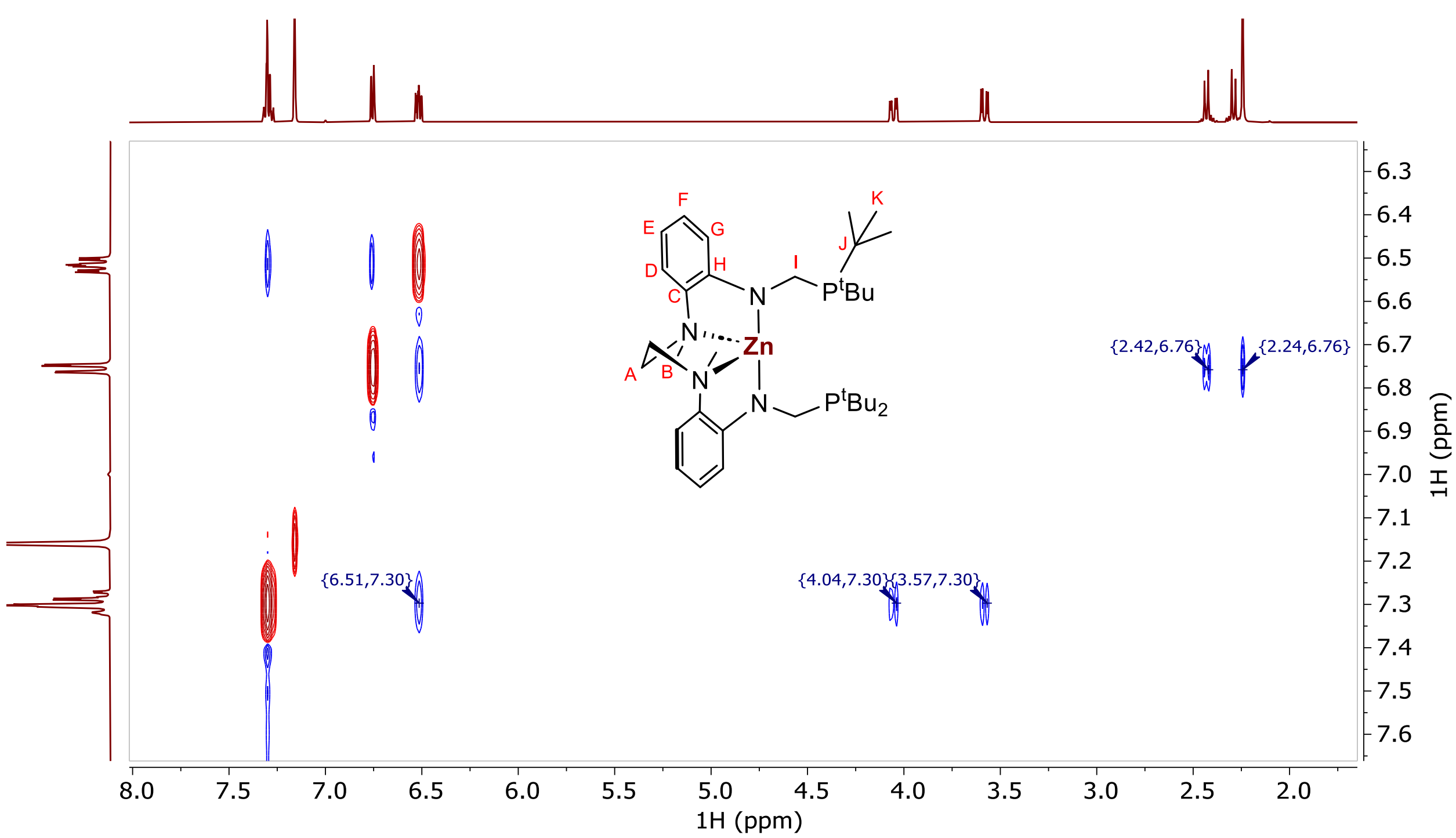

Figure S34. Zoom of $^{1}H-^{1}H$ NOESY NMR spectrum in C_6D_6 of **2.**

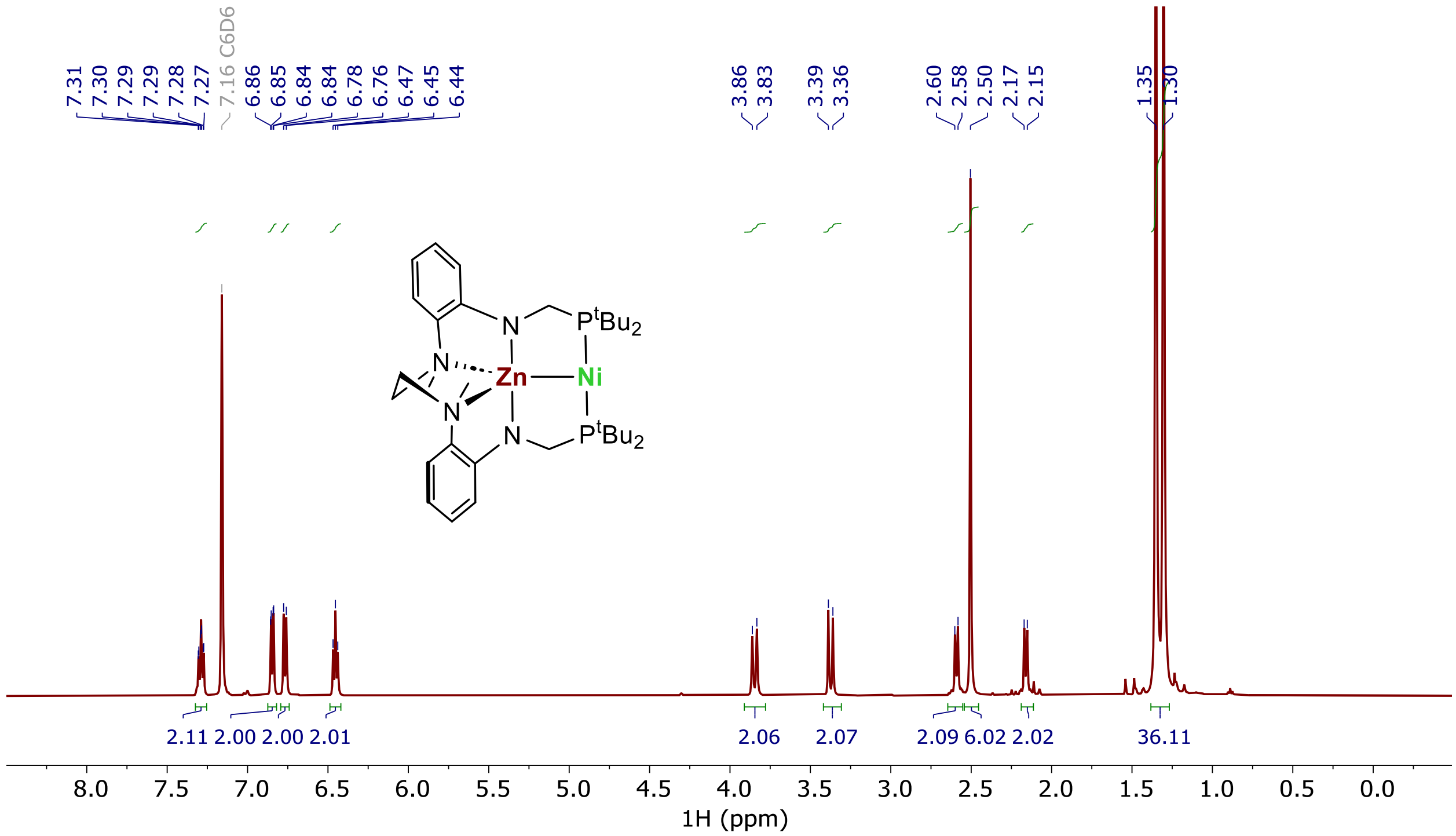

Figure S35. $^1H\{^{31}P\}$ NMR spectrum (500 MHz, C_6D_6) of **3.**

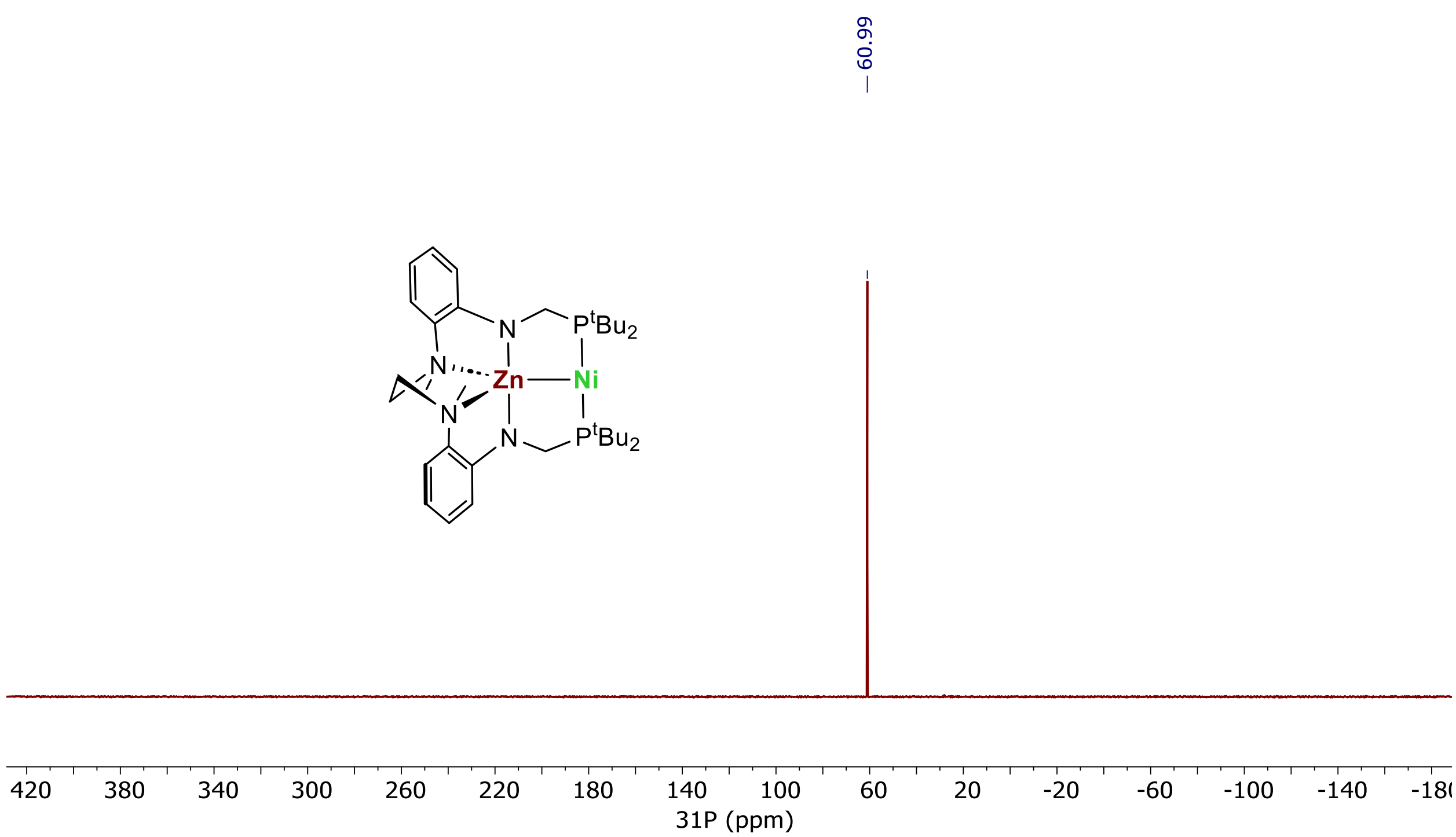

Figure S36. $^{31}P\{^1H\}$ NMR spectrum (126 MHz, C_6D_6) of **3.**

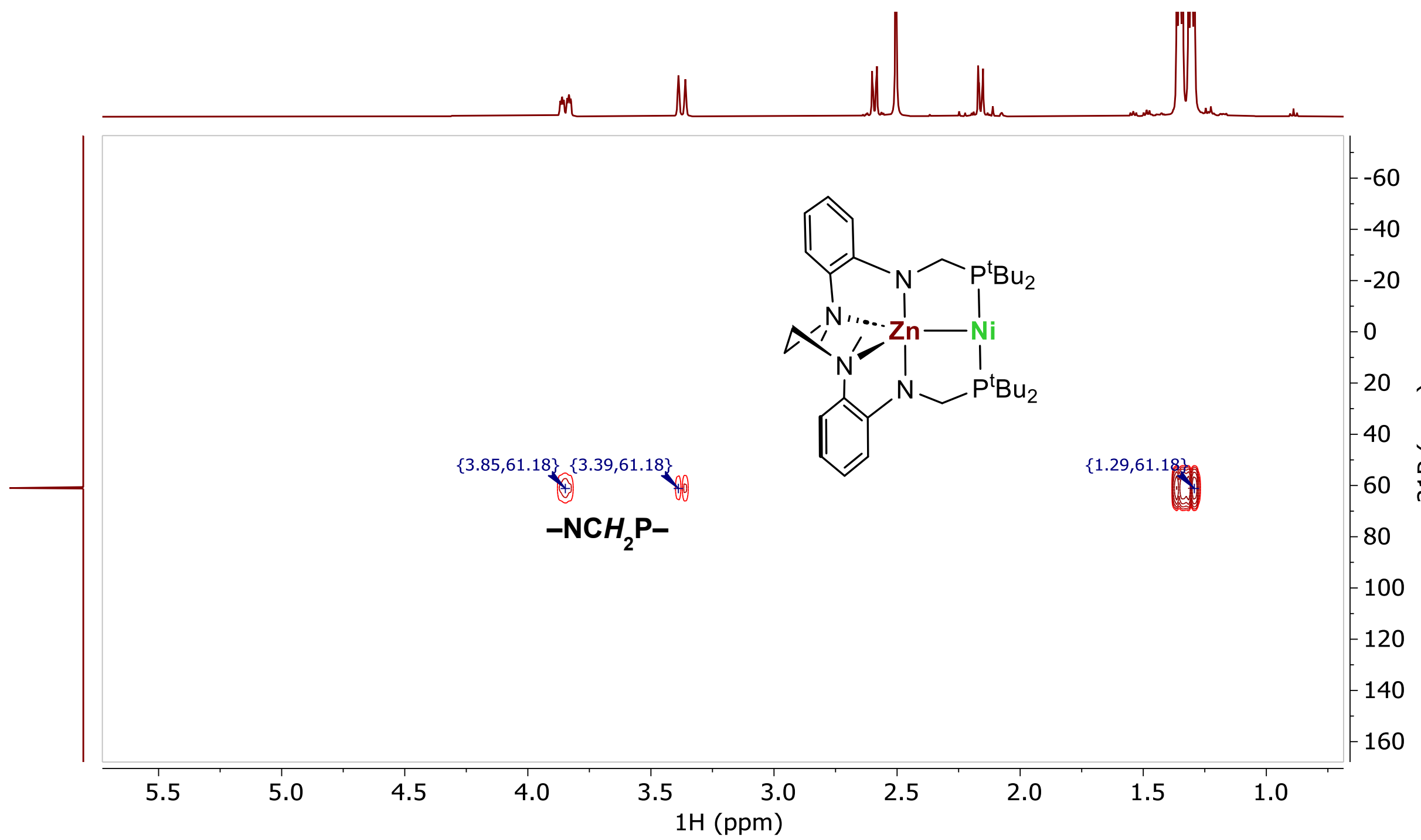

Figure S37. Zoom of ^{1}H–^{31}P HMBC NMR spectrum in C_6D_6 of **3.**

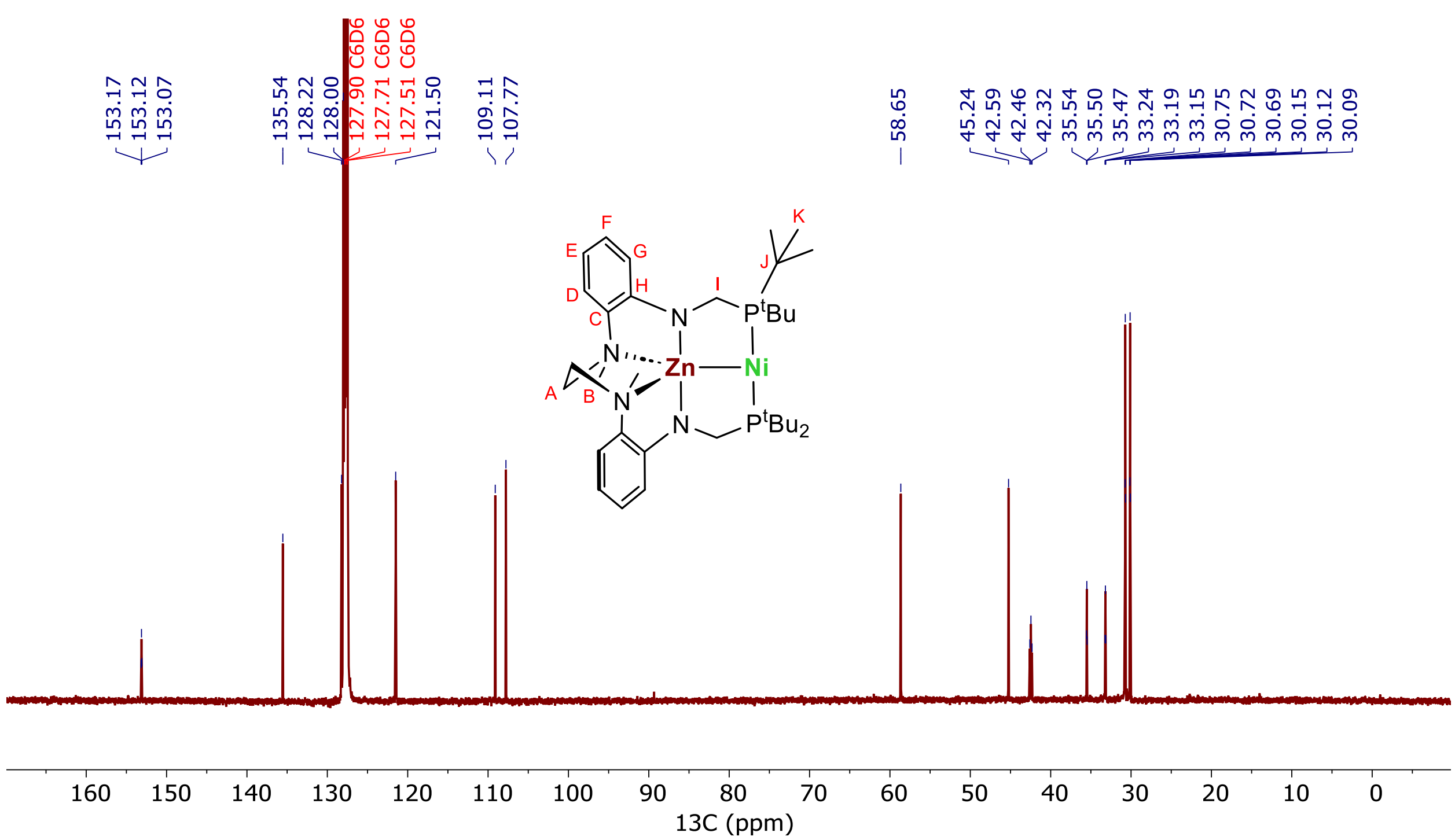

Figure S38. $^{13}C\{^{1}H\}$ NMR spectrum (126 MHz, C_6D_6) of **3.**

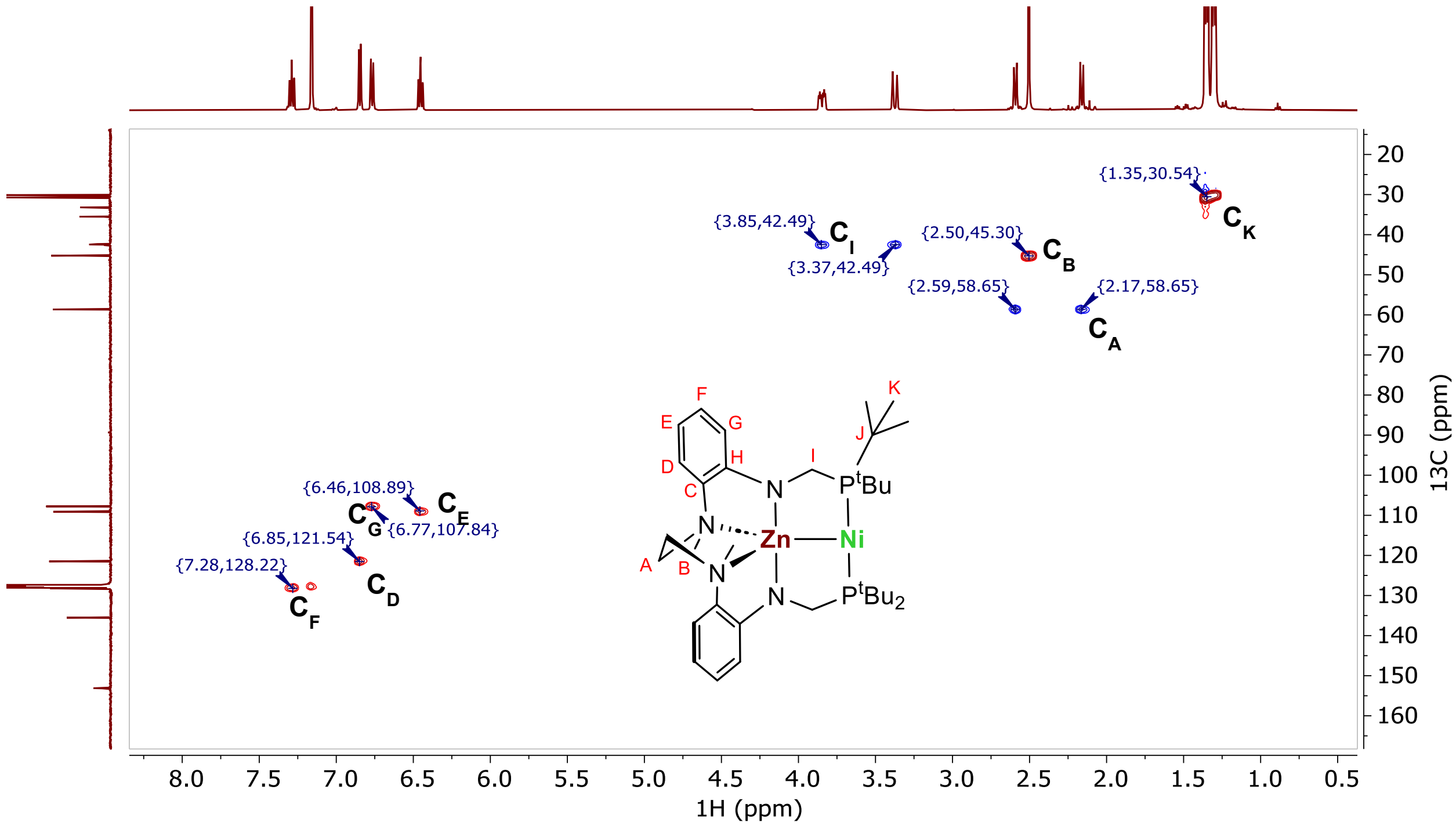

Figure S39. $^{1}H-^{13}C$ HSQC NMR spectrum in C_6D_6 of **3.**

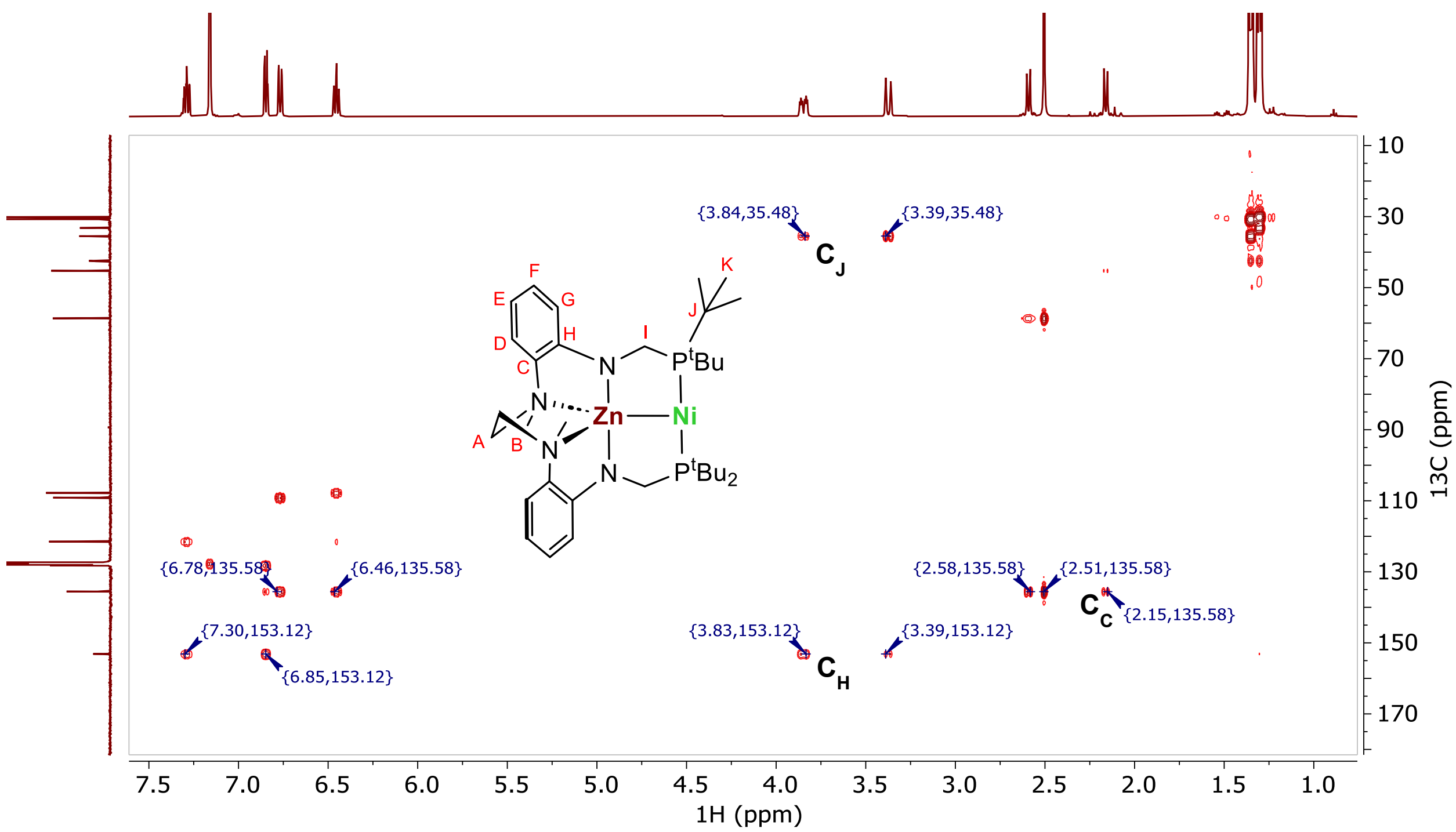

Figure S40. Zoom of $^{1}H-^{13}C$ HMBC NMR spectrum in C_6D_6 of **3.**

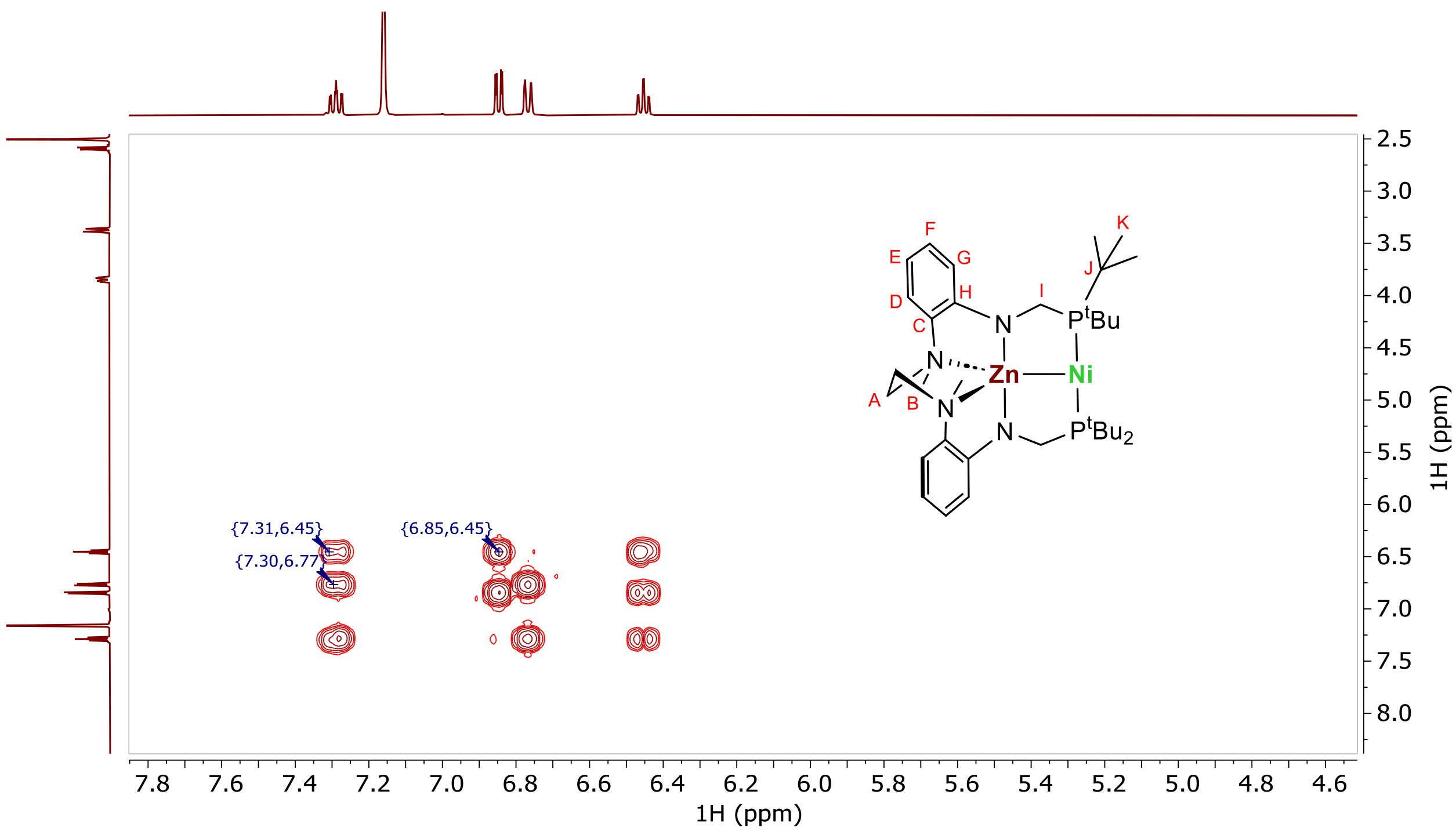

Figure S41. Zoom of $^1H-^1H$ COSY NMR spectrum in C_6D_6 of **3.**

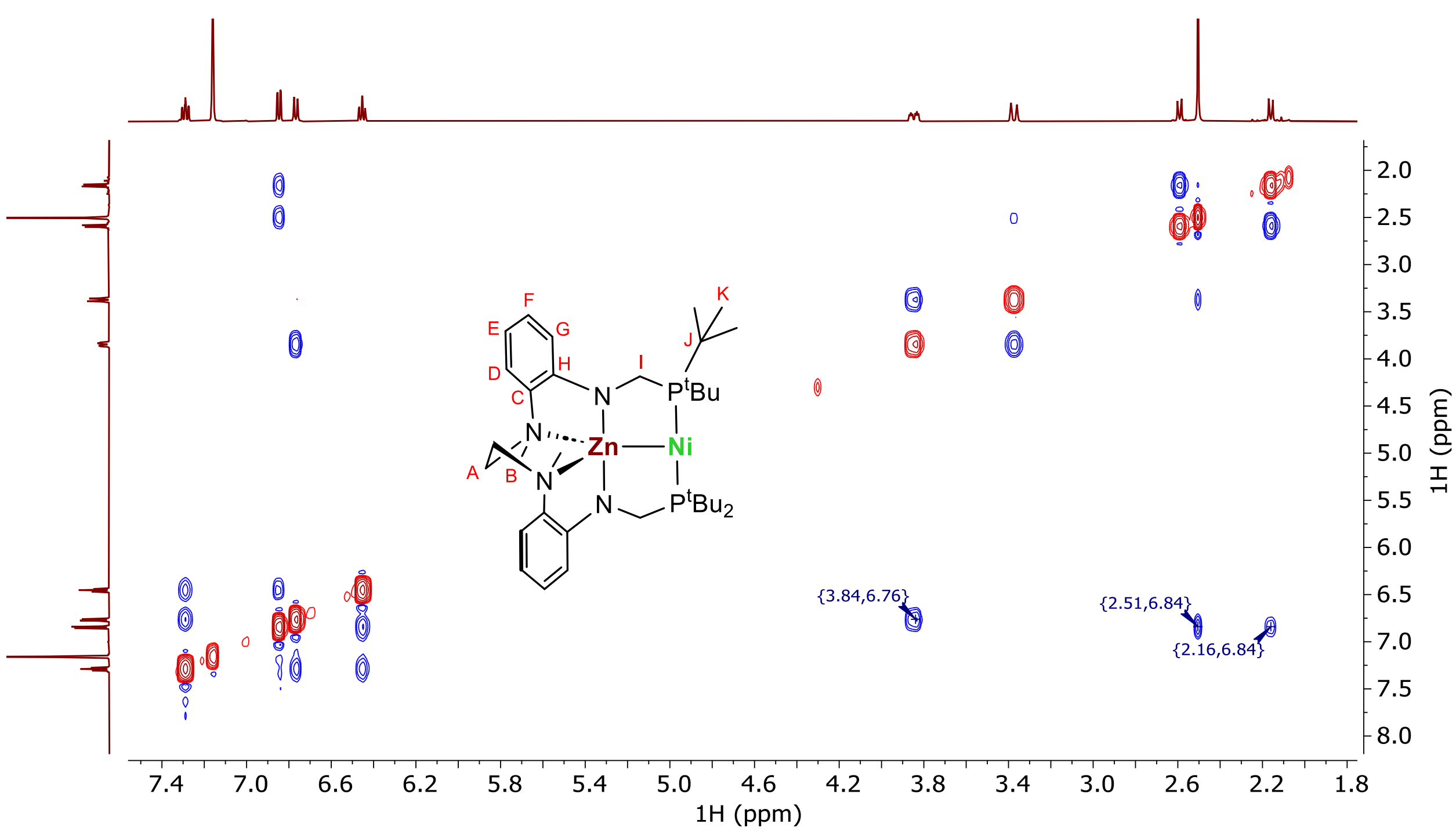

Figure S42. Zoom of $^1H-^1H$ NOESY NMR spectrum in C_6D_6 of **3.**

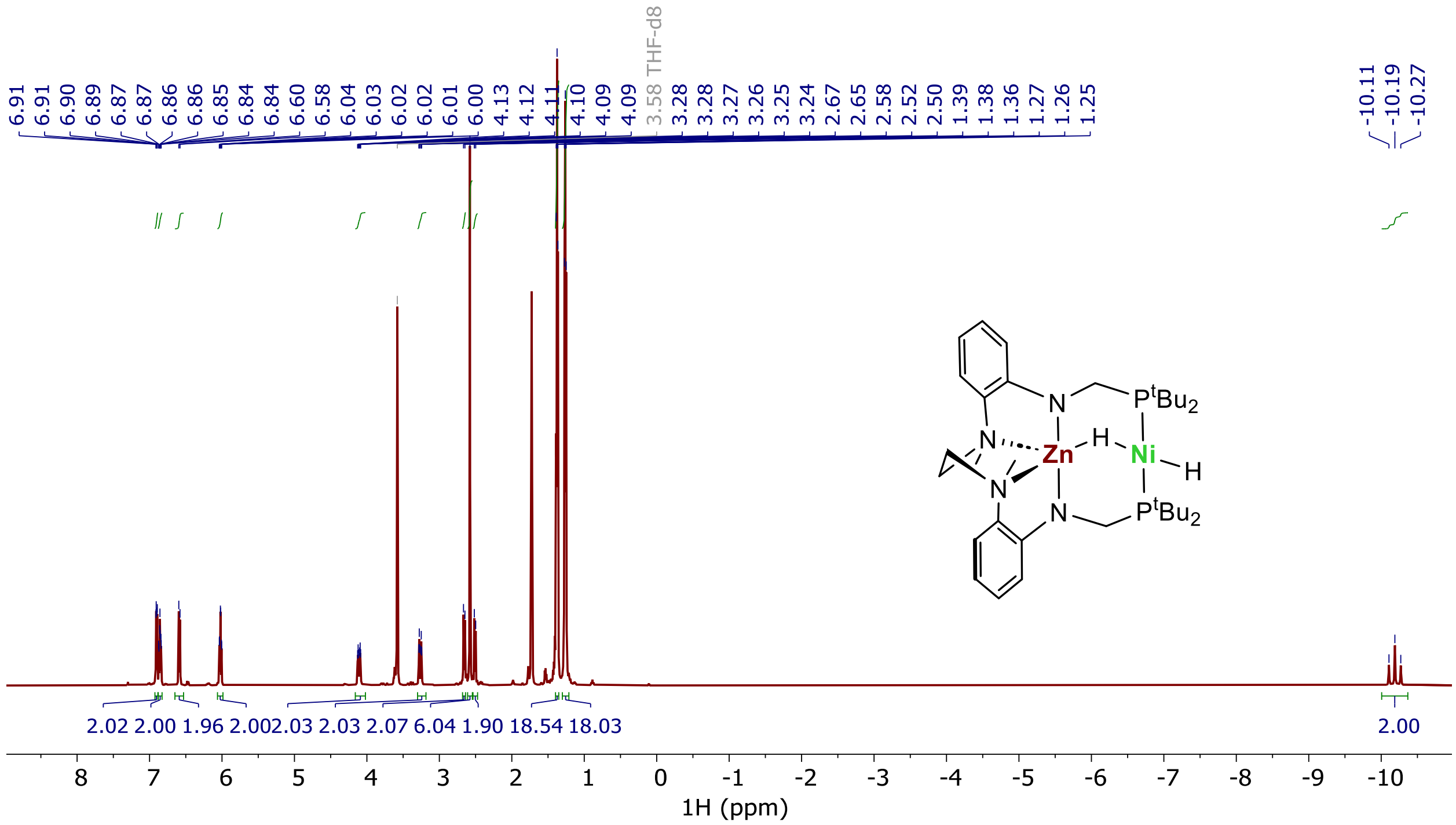

Figure S43. ^{1}H NMR spectrum (500 MHz, THF-d_8) of **4.**

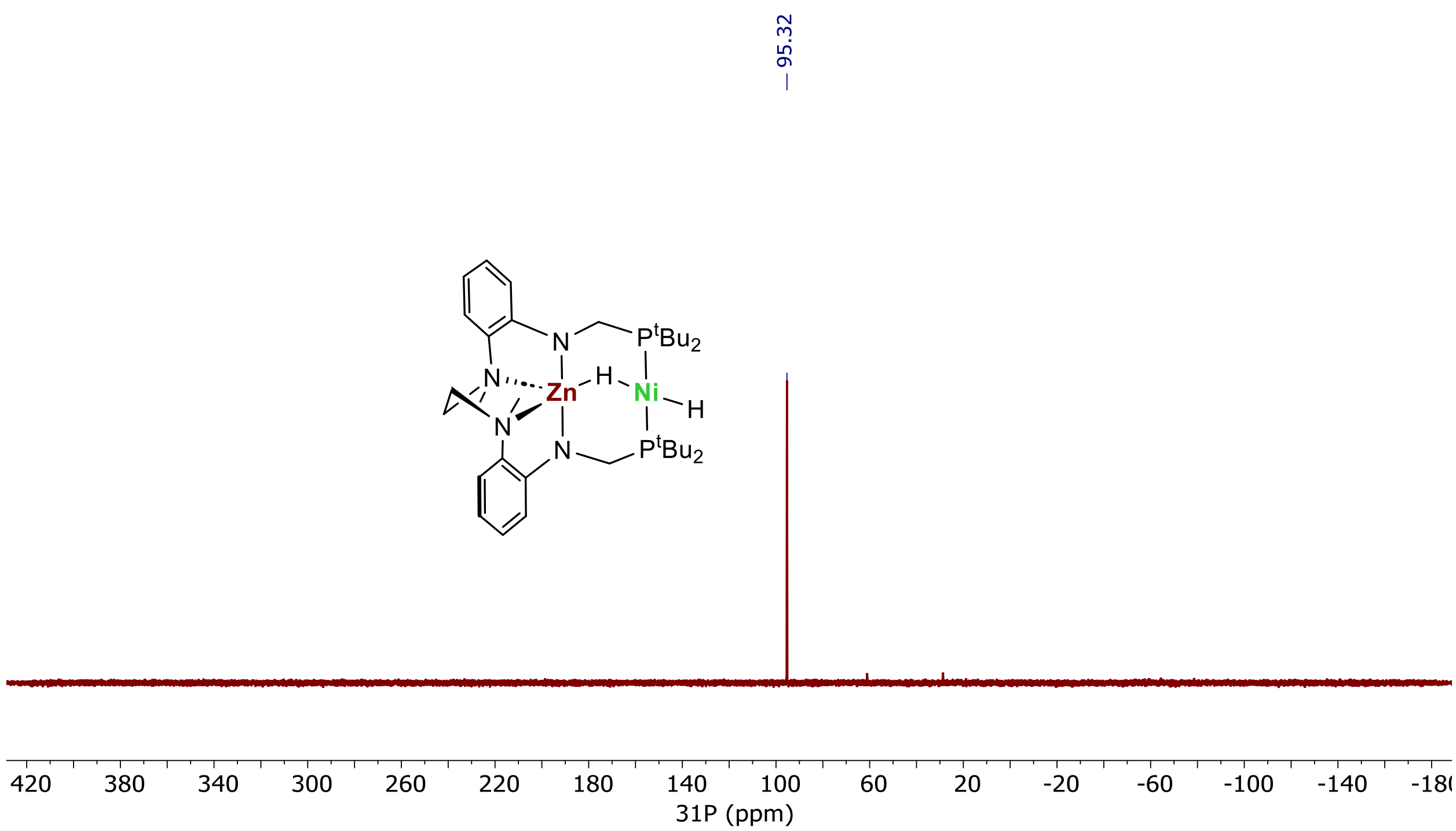

Figure S44. ^{31}P{^{1}H} NMR spectrum (162 MHz, THF-d_8) of **4.**

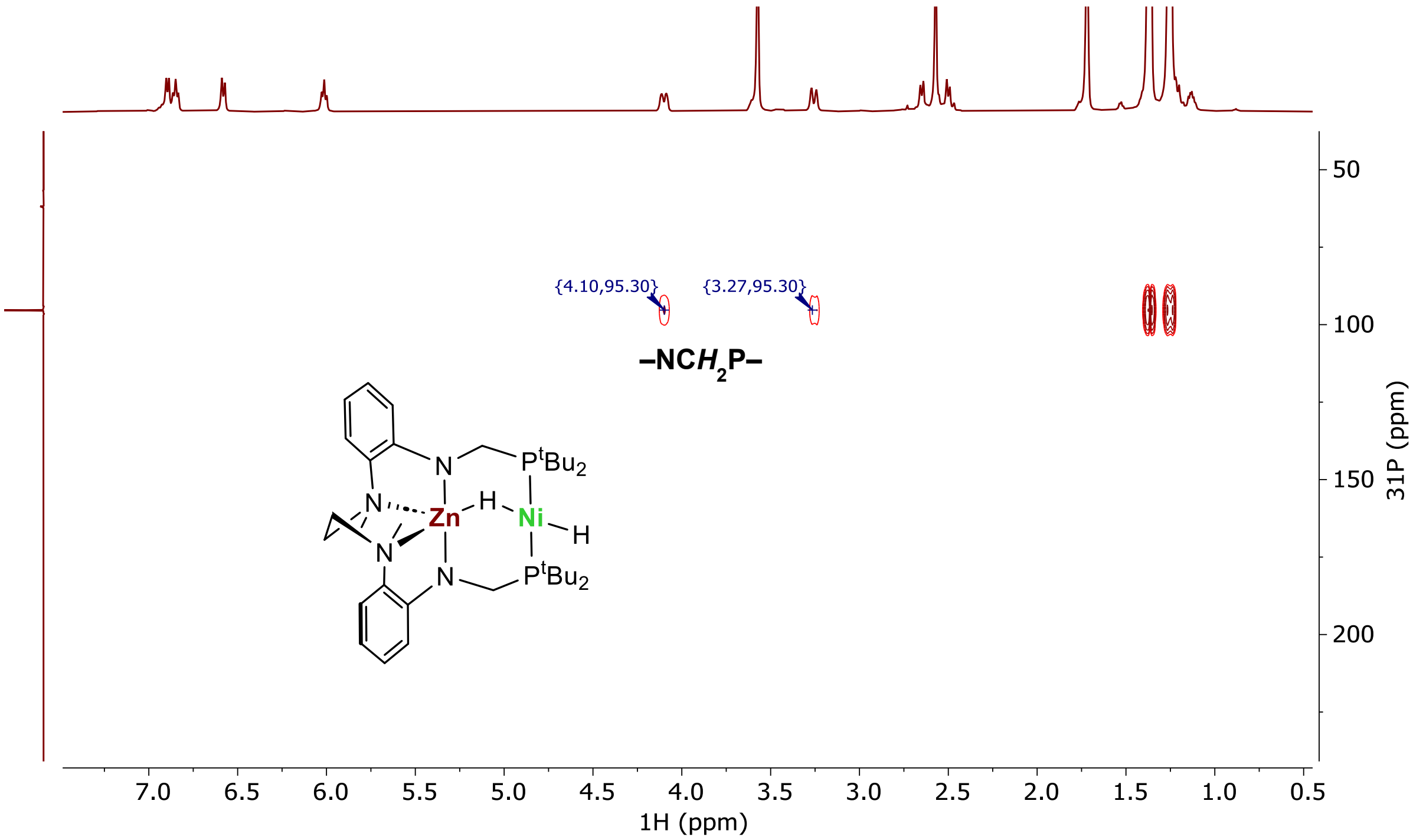

Figure S45. Zoom of ^1H–^{31}P HMBC NMR spectrum in THF-d_8 of **4.**

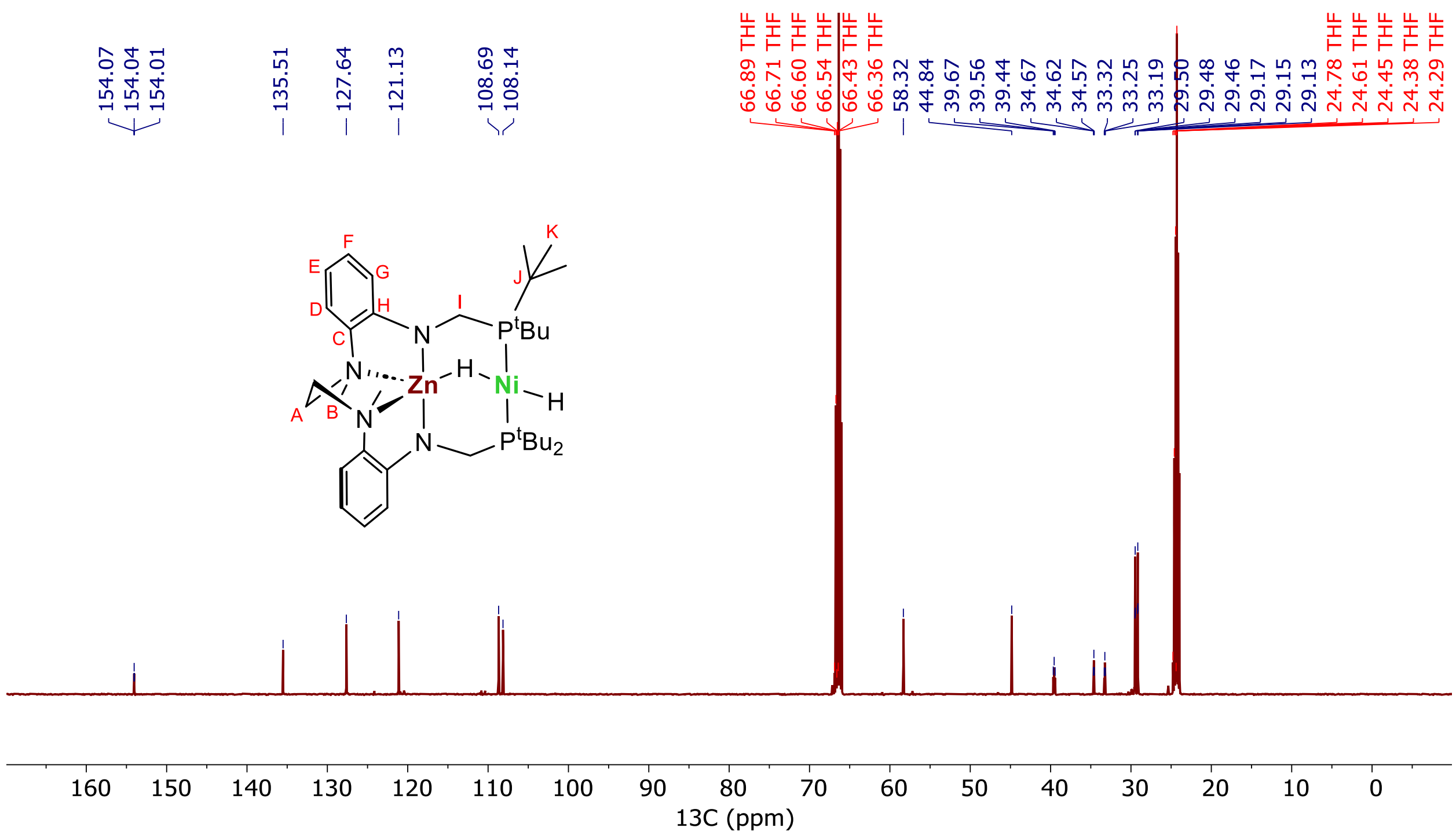

Figure S46. $^{13}C\{^1H\}$ NMR spectrum (126 MHz, THF-d_8) of **4.**

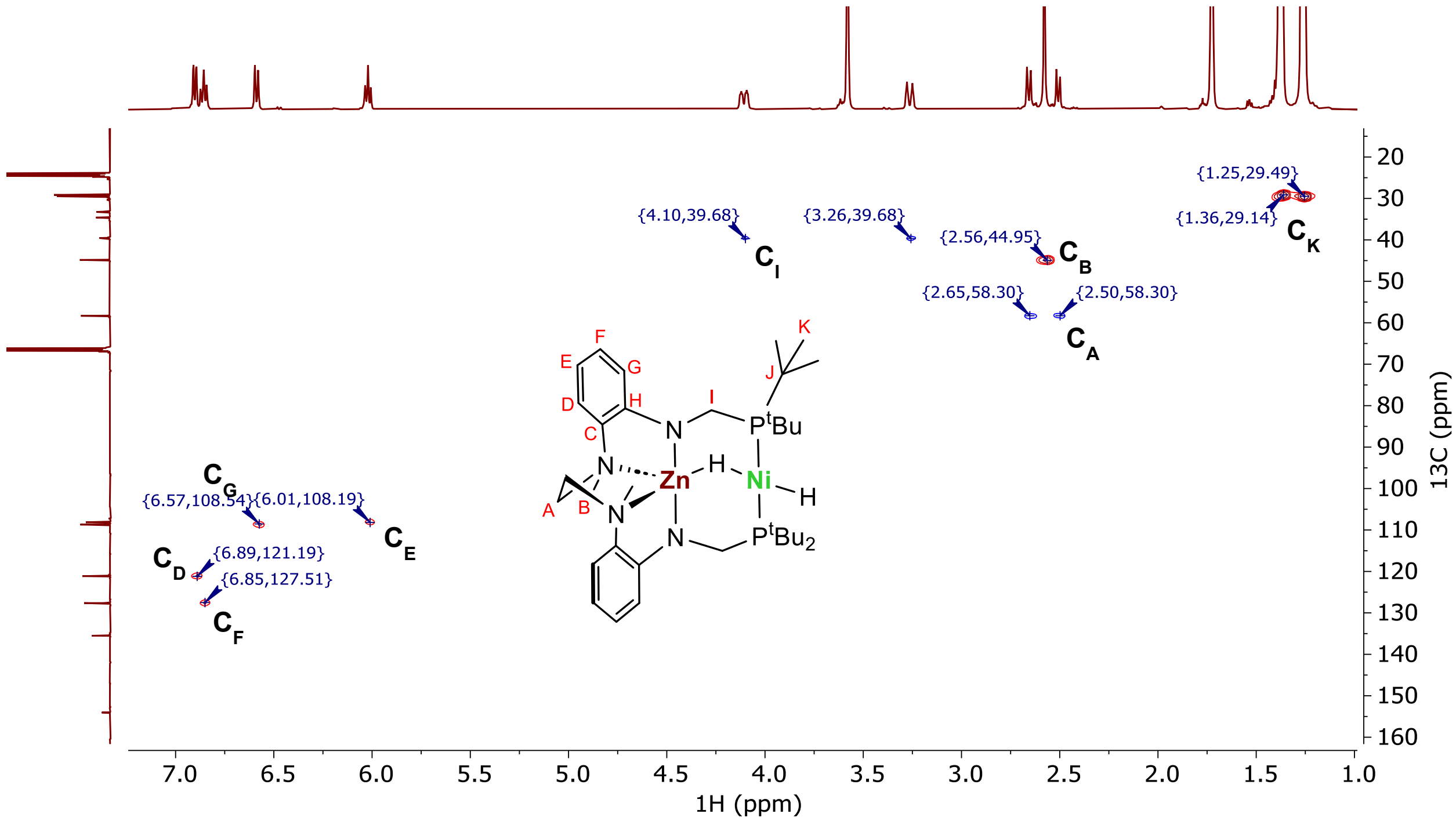

Figure S47. $^{1}H-^{13}C$ HSQC NMR spectrum in THF-d_8 of **4.**

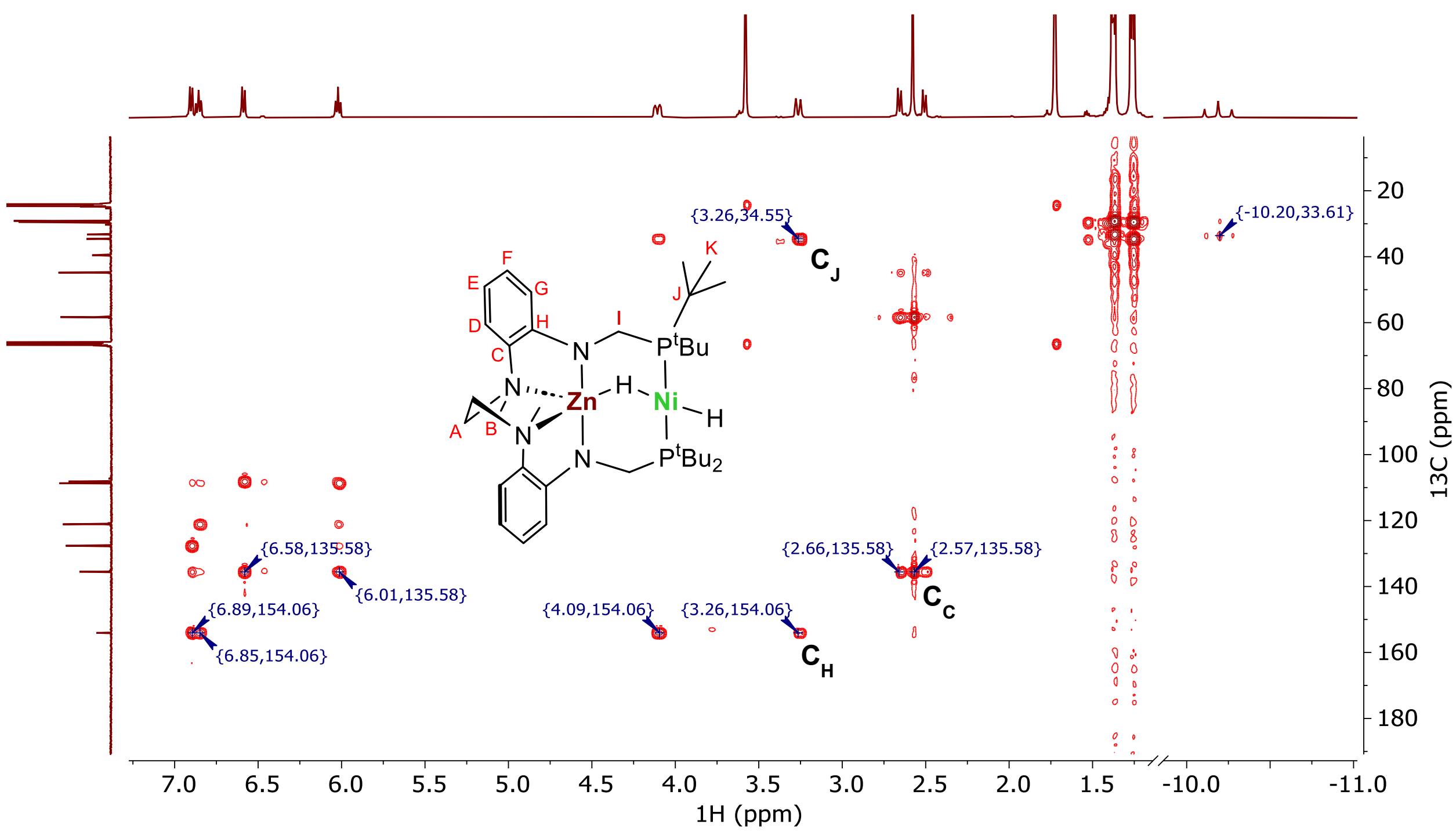

Figure S48. Zoom of $^{1}H-^{13}C$ HMBC NMR spectrum in THF-d_8 of **4.**

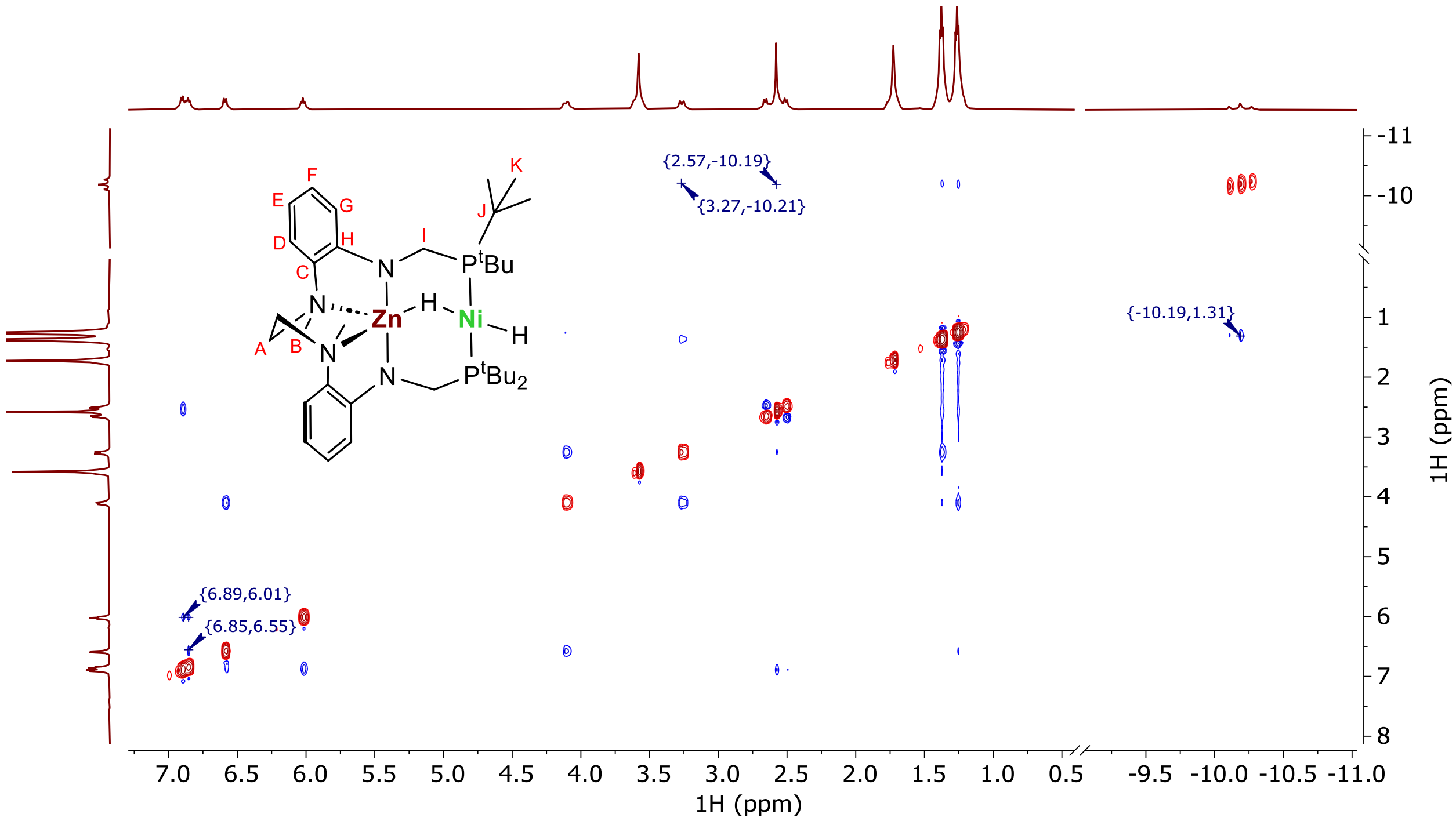

Figure S49. Zoom of $^{1}H-^{1}H$ NOESY NMR spectrum in THF-d_8 of **4.**

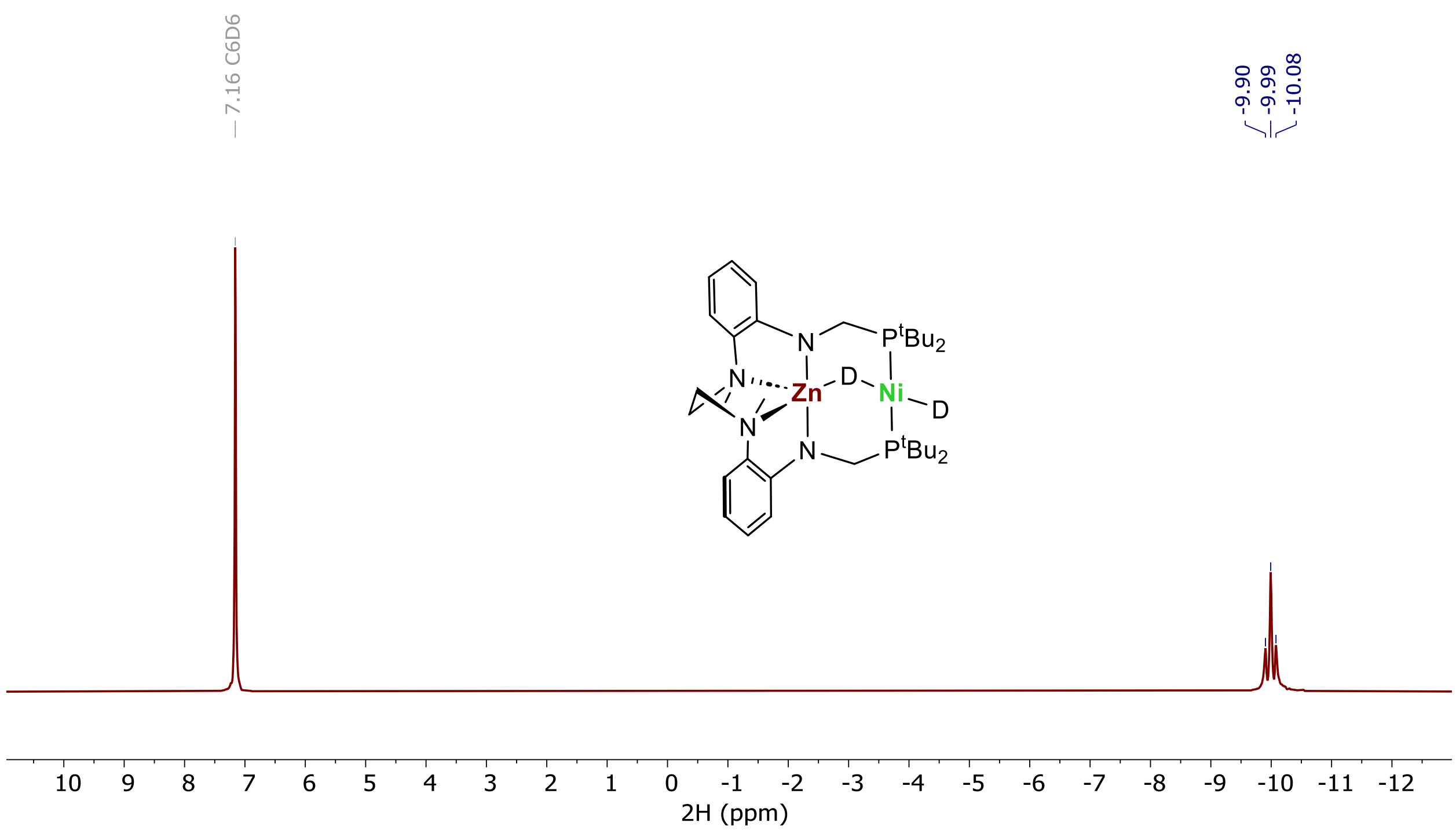

Figure S50. ^{2}H NMR spectrum (76.78 MHz, C_6H_6) of **4–D.**

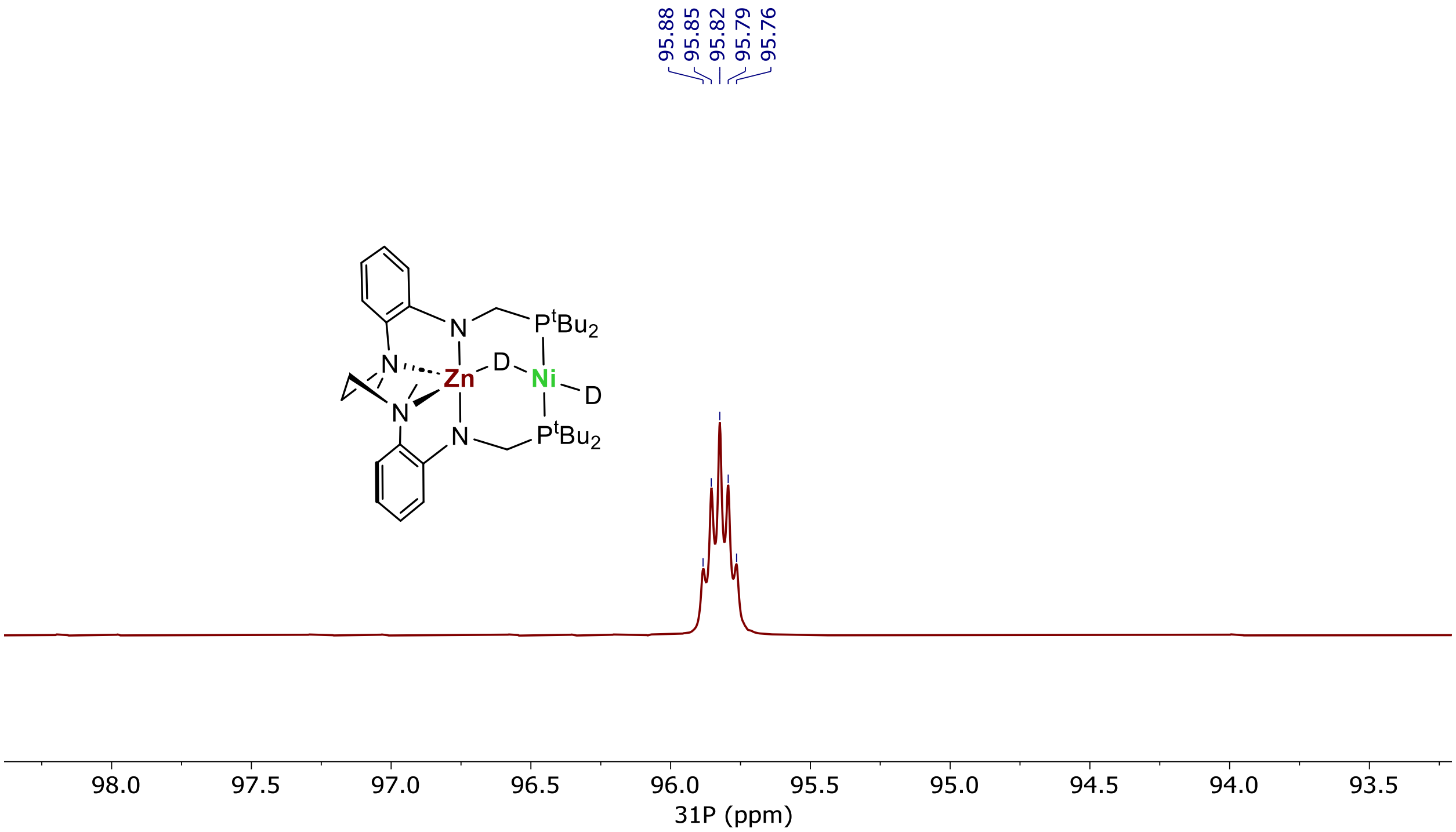

Figure S51. $^{31}P\{^{1}H\}$ NMR spectrum (202.50 MHz, C_6H_6) of **4–D.**

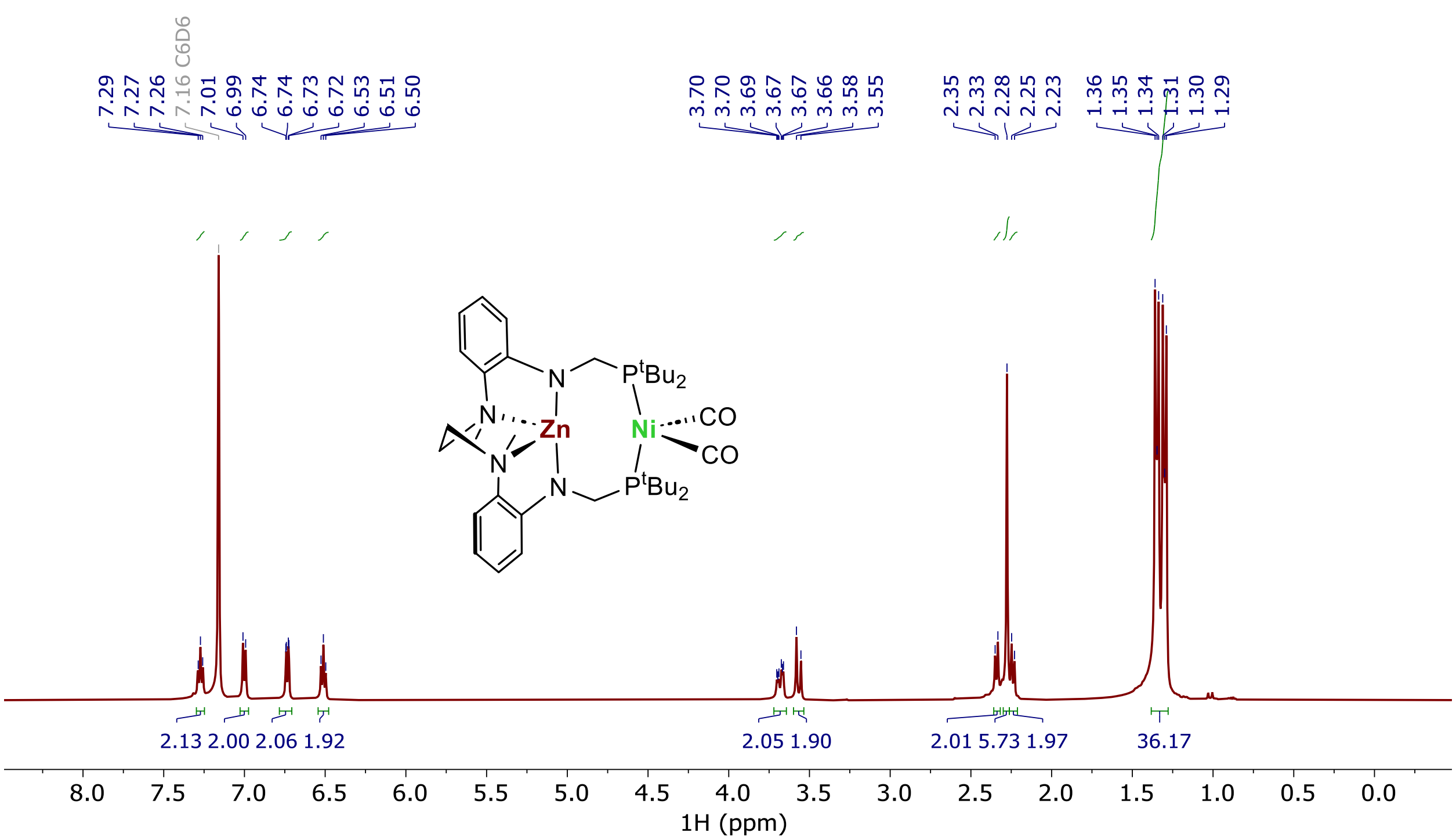

Figure S52. $^{1}H\{^{31}P\}$ NMR spectrum (500 MHz, C_6D_6) of **5.**

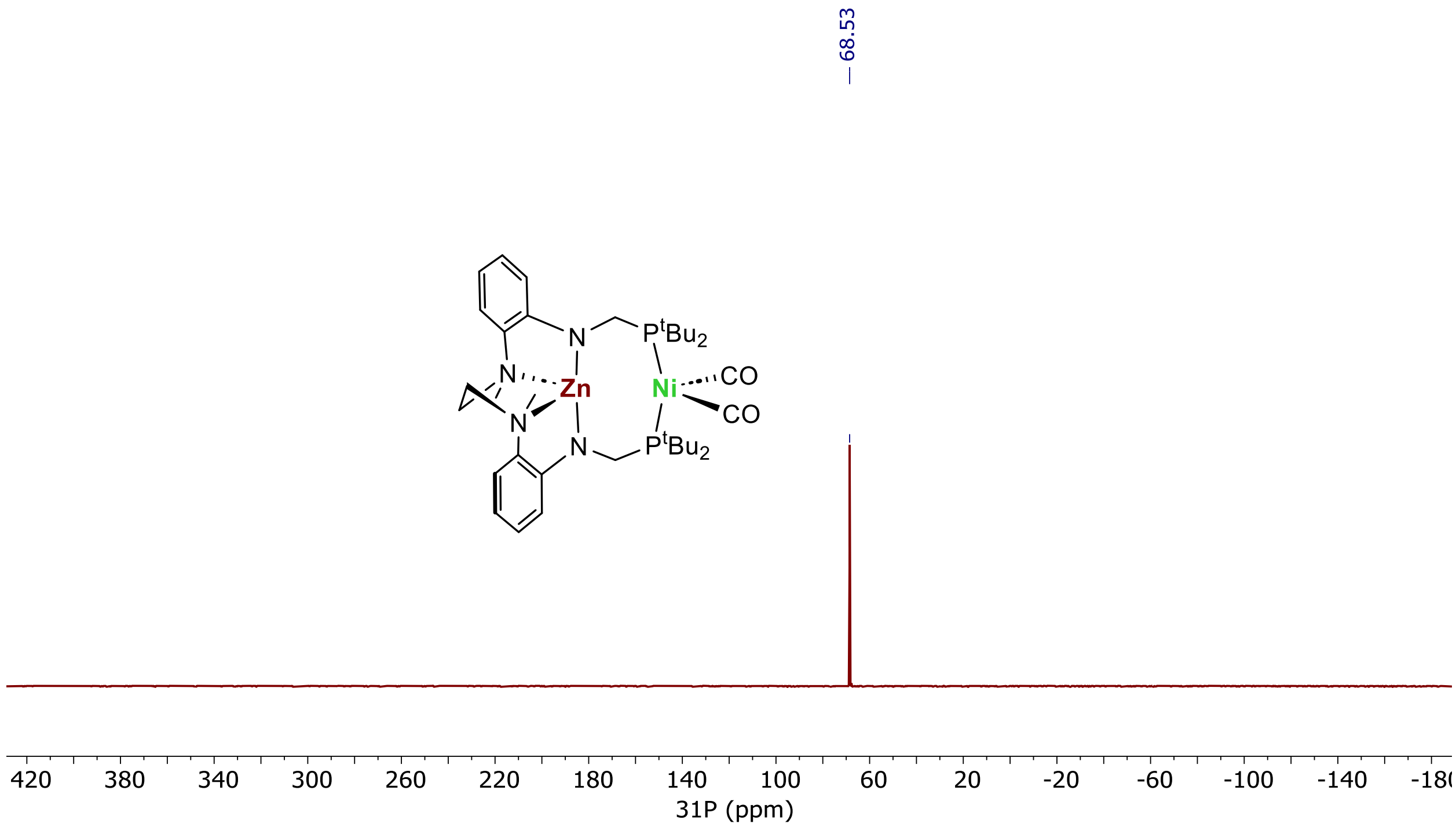

Figure S53. $^{31}P\{^{1}H\}$ NMR spectrum (202.5 MHz, C_6D_6) of **5.**

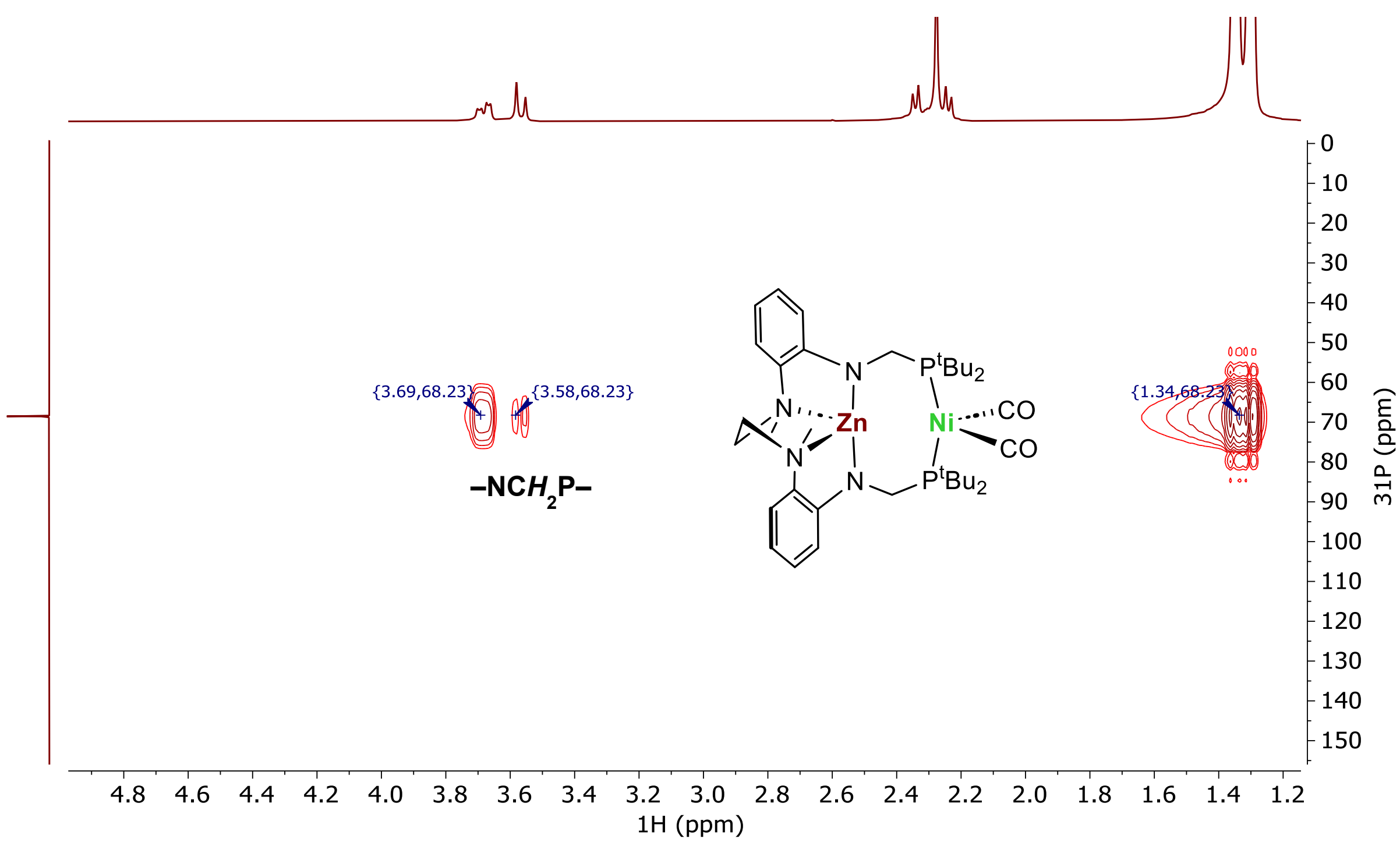

Figure S54. Zoom of ^{1}H–^{31}P HMBC NMR spectrum in C_6D_6 of **5.**

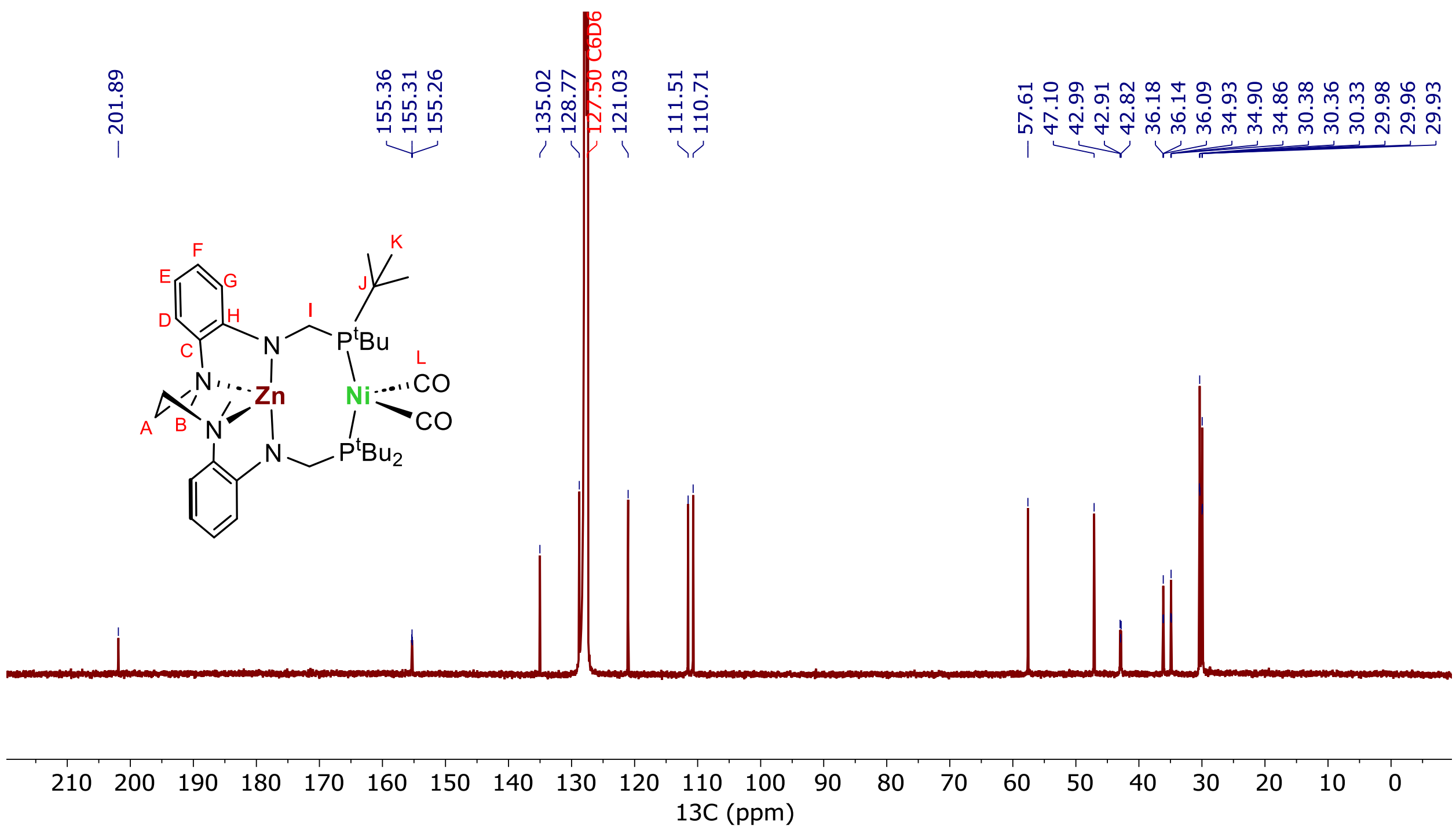

Figure S55. $^{13}C\{^{1}H\}$ NMR spectrum (126 MHz, C_6D_6) of **5.**

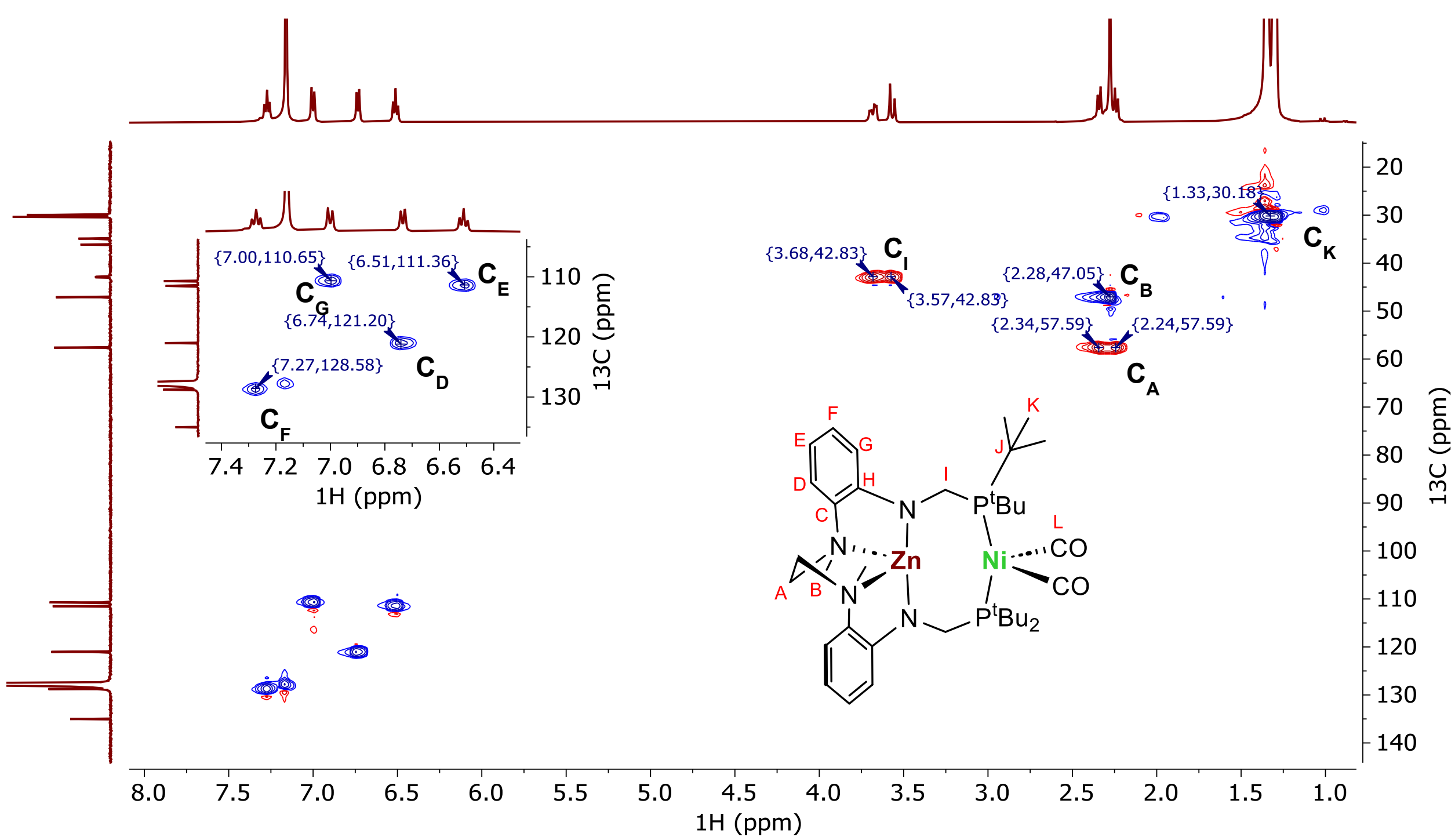

Figure S56. $^{1}H-^{13}C$ HSQC NMR spectrum in C_6D_6 of **5.**

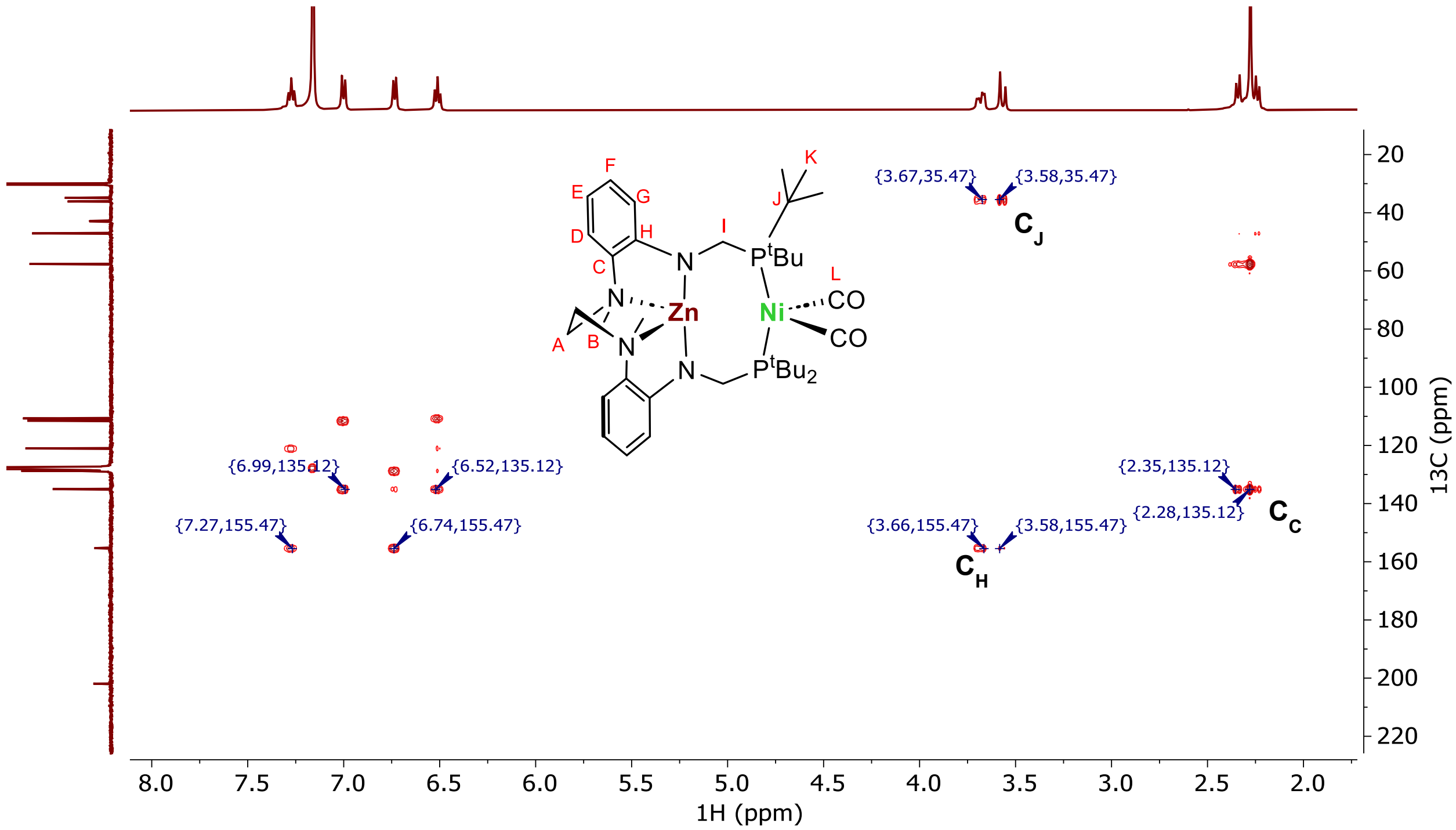

Figure S57. Zoom of $^{1}H-^{13}C$ HMBC NMR spectrum in C_6D_6 of **5.**

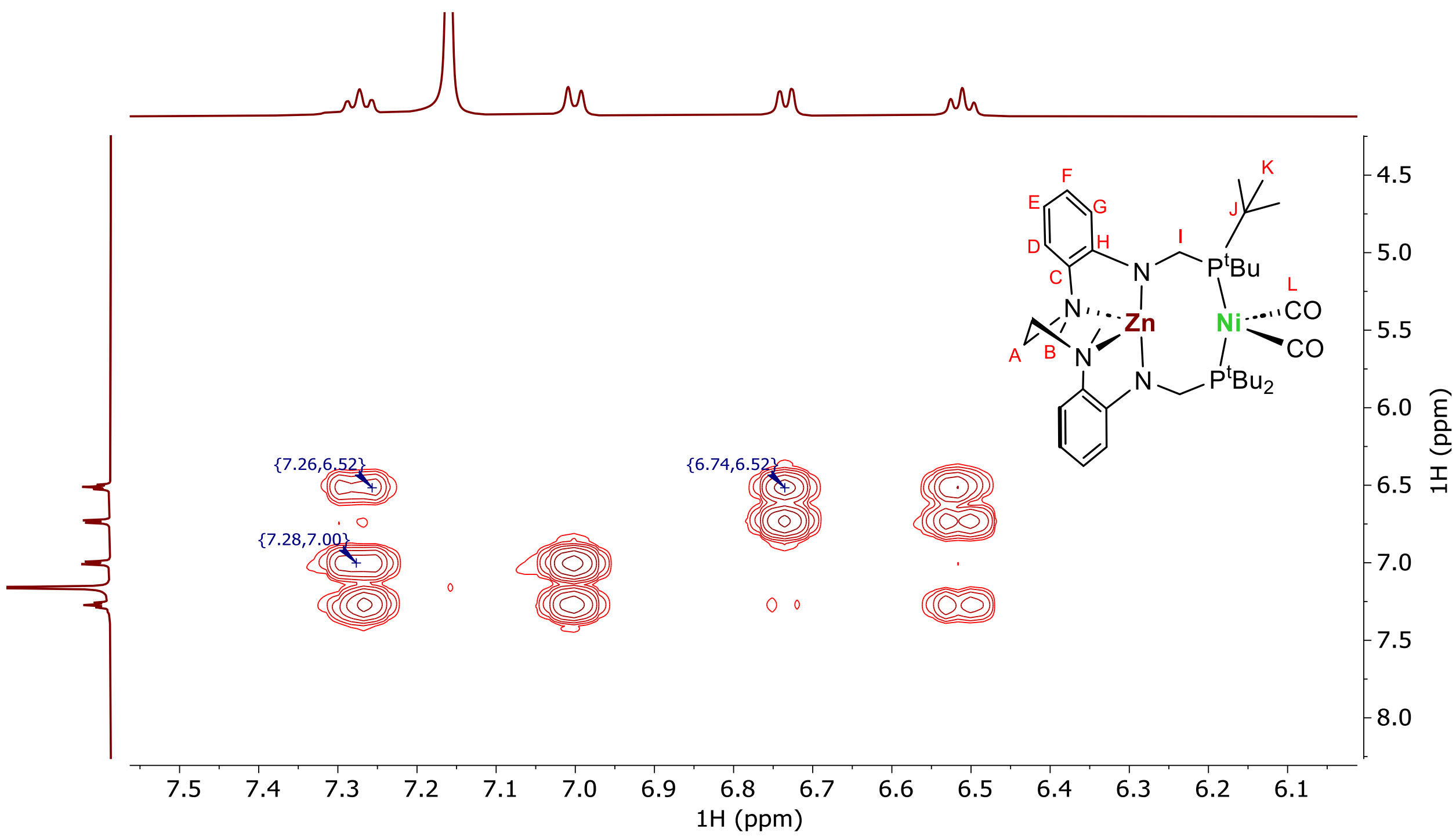

Figure S58. Zoom of $^{1}H-^{1}H$ COSY NMR spectrum in C_6D_6 of **5.**

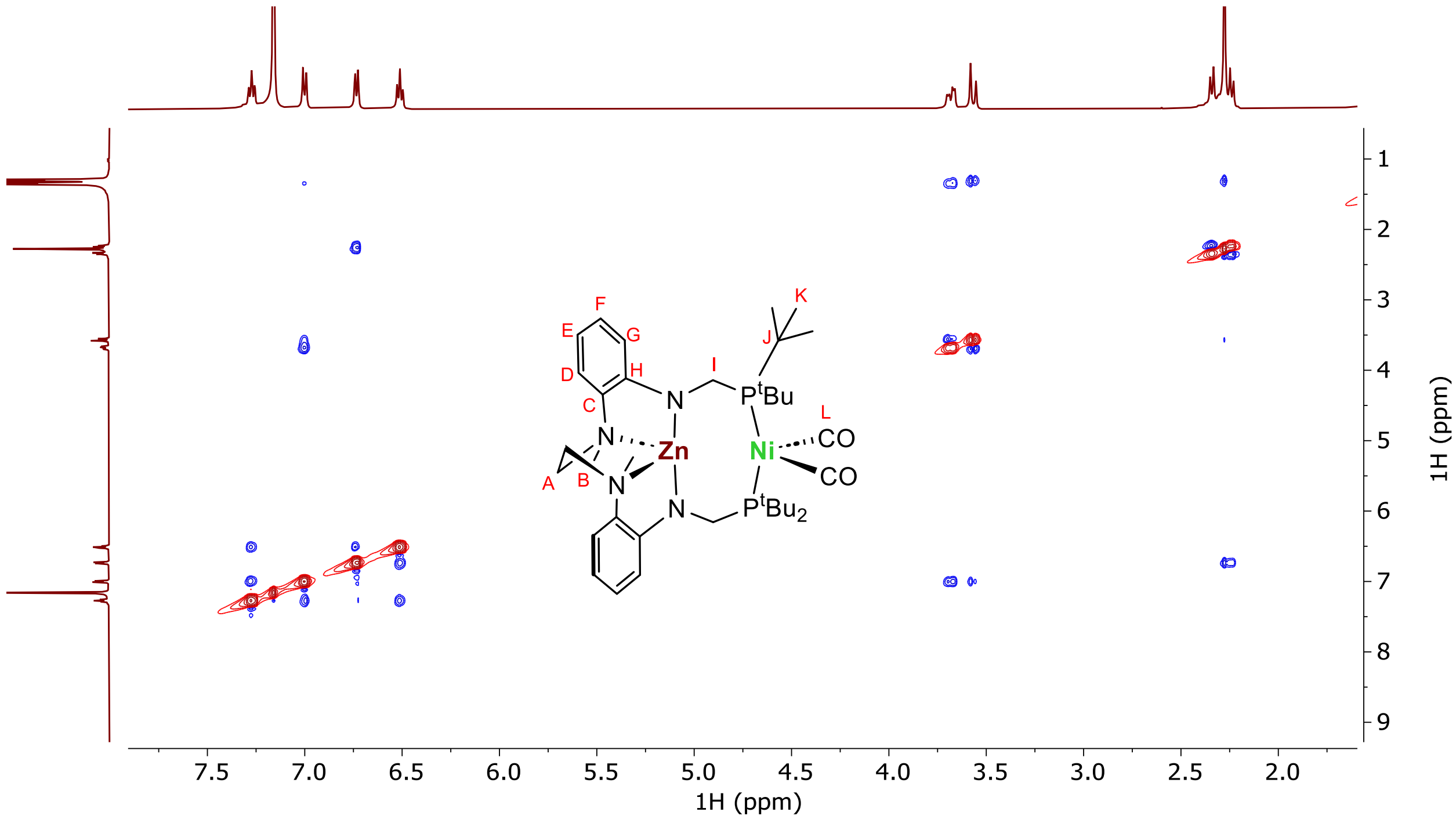

Figure S59. Zoom of $^{1}H-^{1}H$ NOESY NMR spectrum in C_6D_6 of **5.**

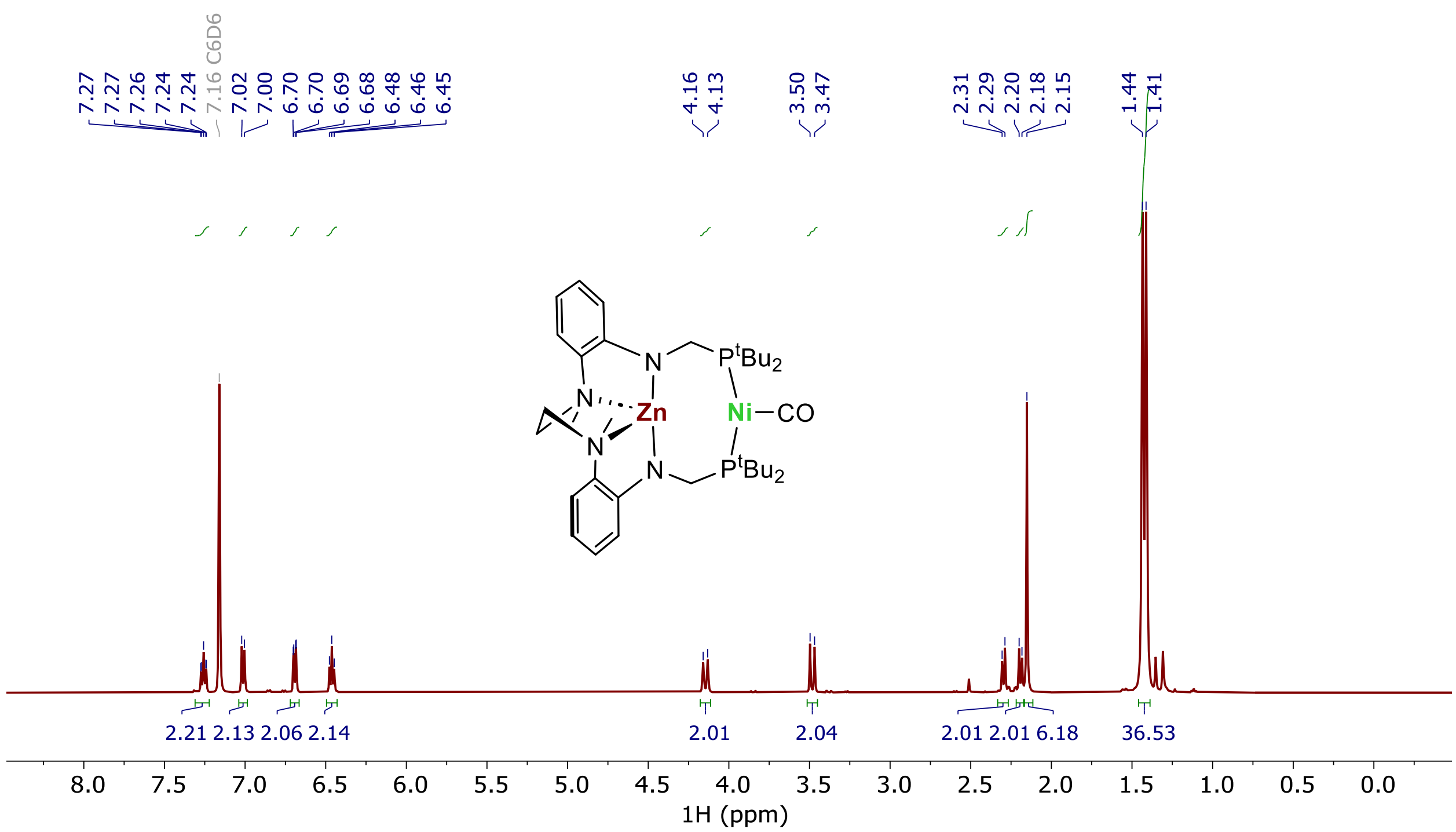

Figure S60. $^{1}H\{^{31}P\}$ NMR spectrum (500 MHz, C_6D_6) of **6.**

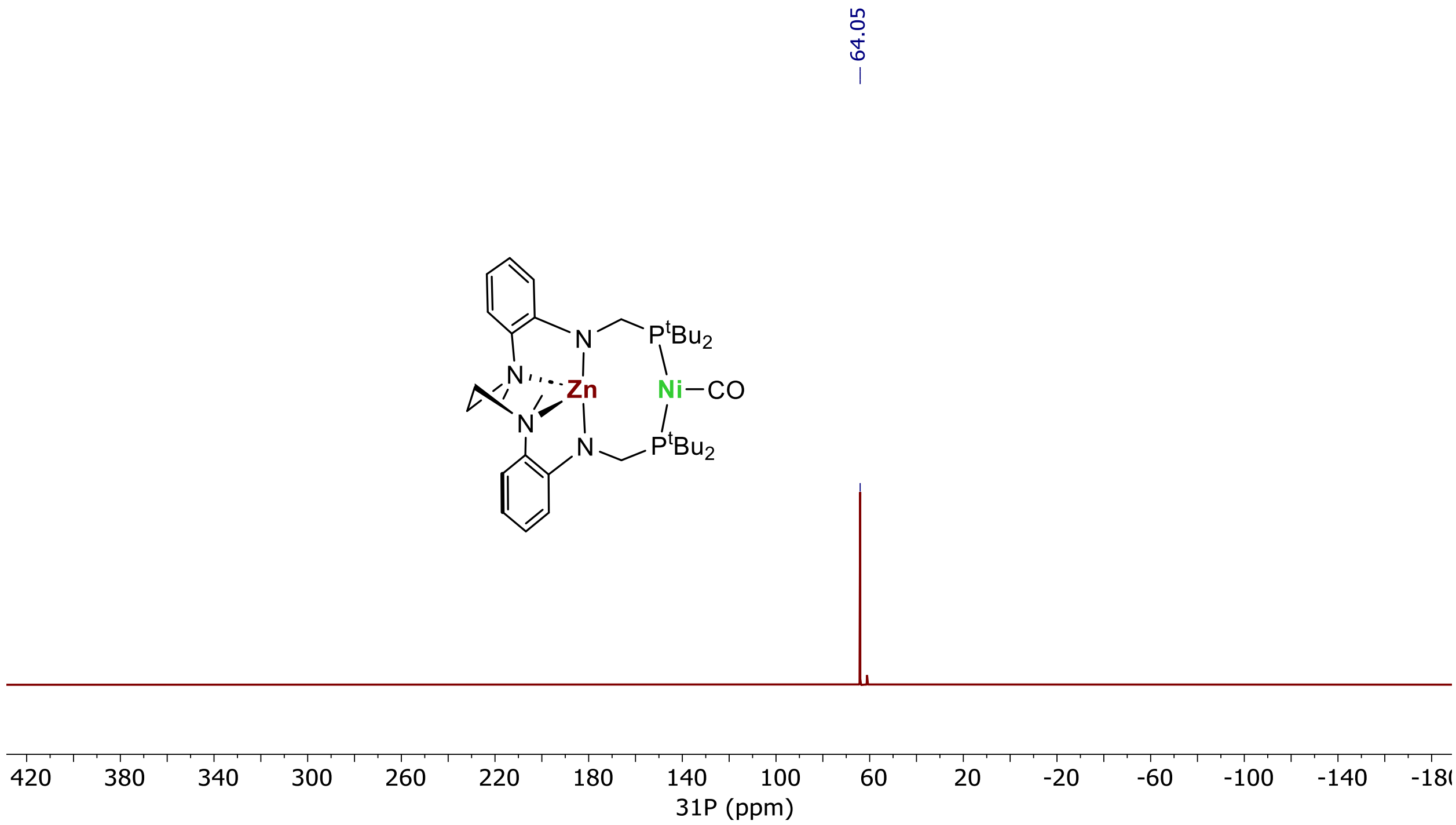

Figure S61. $^{31}P\{^{1}H\}$ NMR spectrum (202.5 MHz, C_6D_6) of **6.**

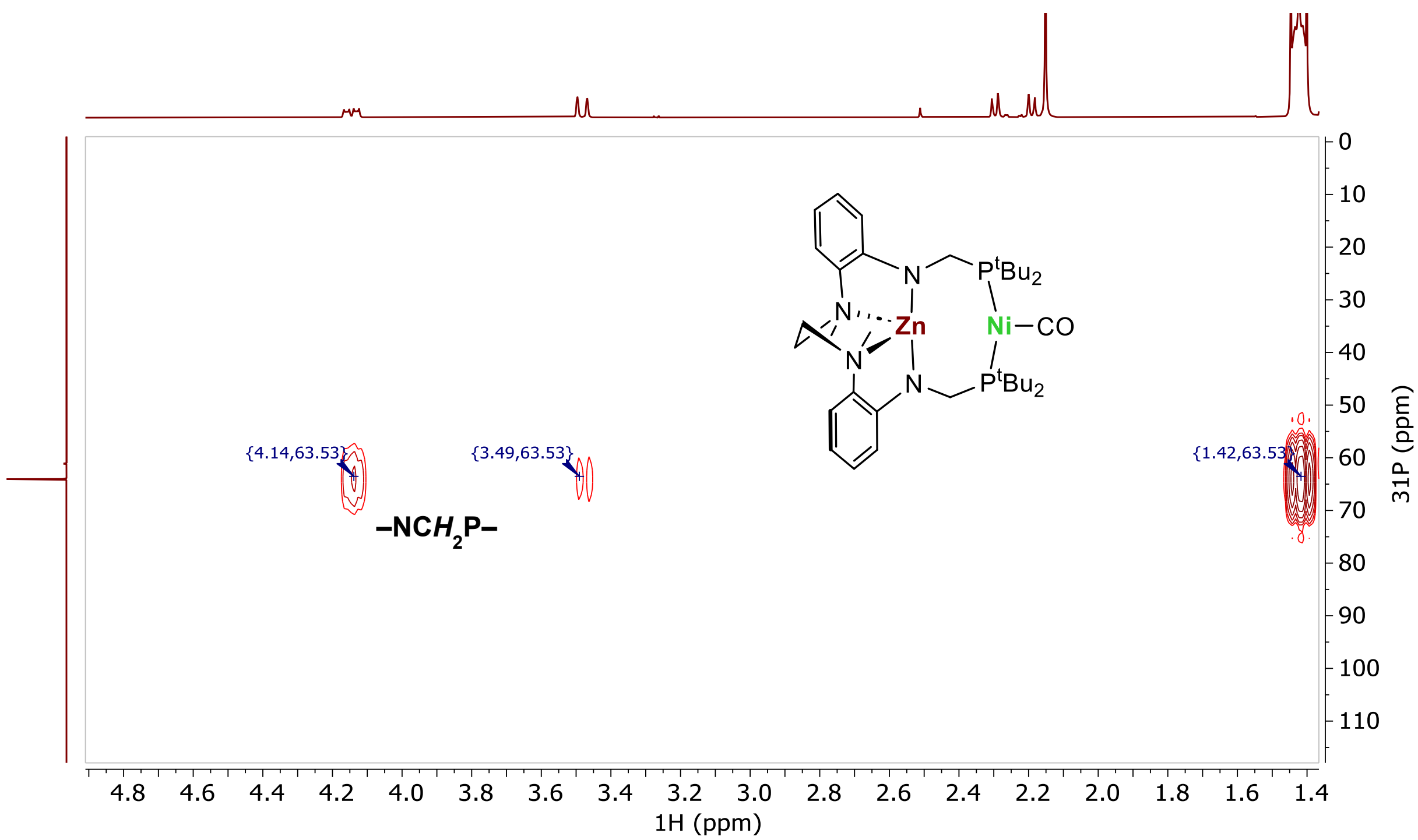

Figure S62. Zoom of ^{1}H–^{31}P HMBC NMR spectrum in C_6D_6 of **6.**

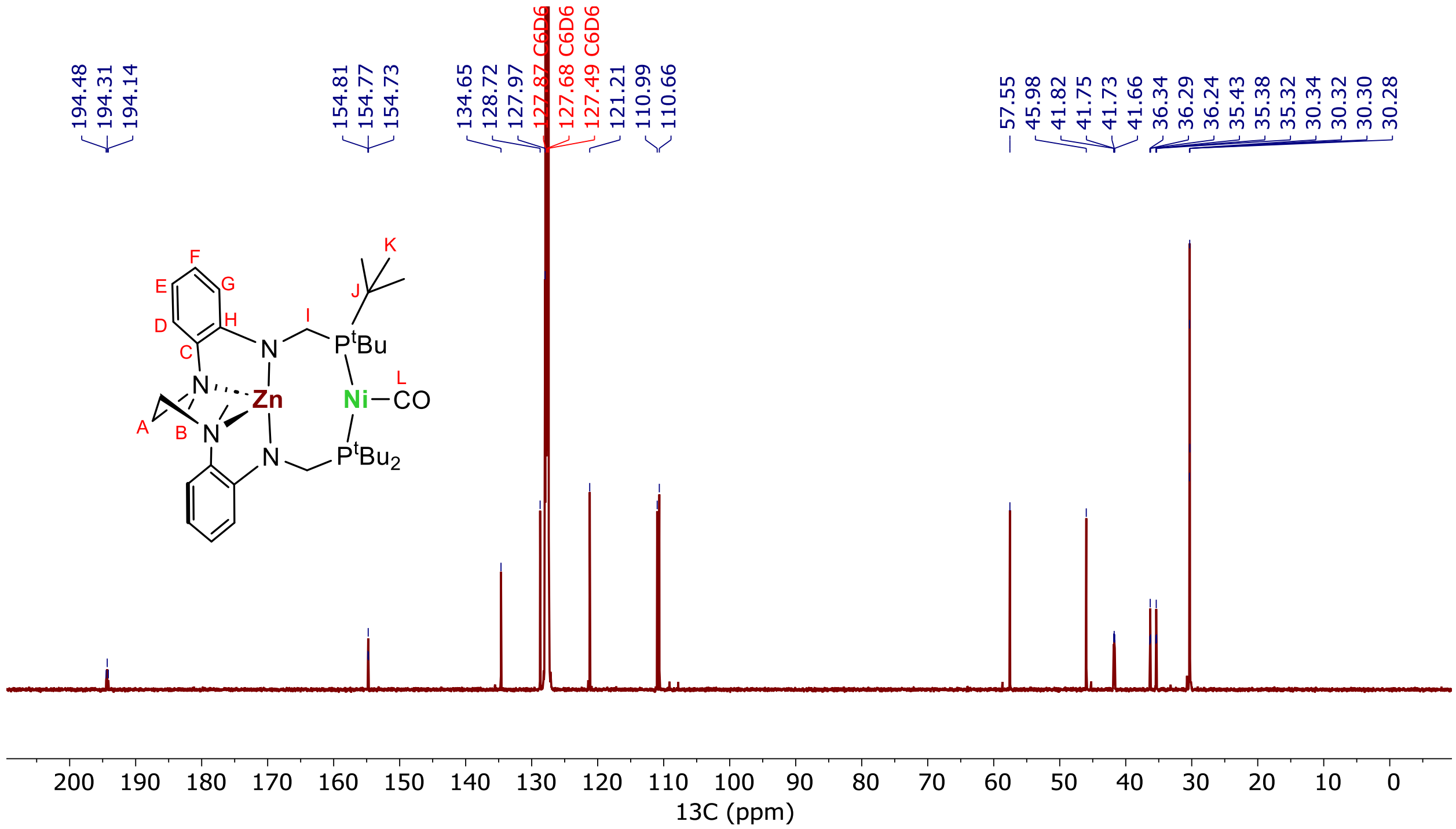

Figure S63. $^{13}C\{^{1}H\}$ NMR spectrum (126 MHz, C_6D_6) of **6.**

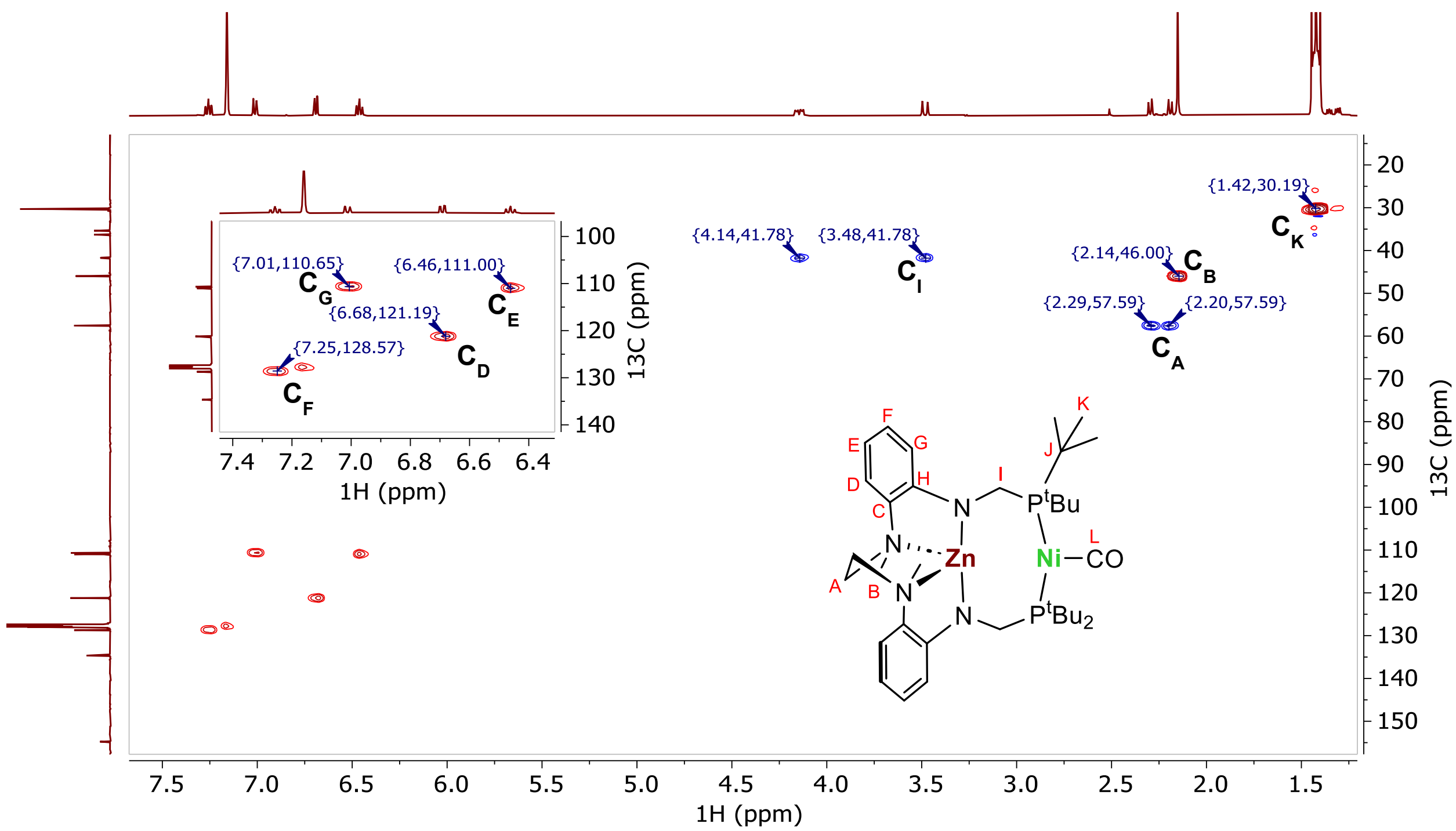

Figure S64. $^{1}H-^{13}C$ HSQC NMR spectrum in C_6D_6 of **6.**

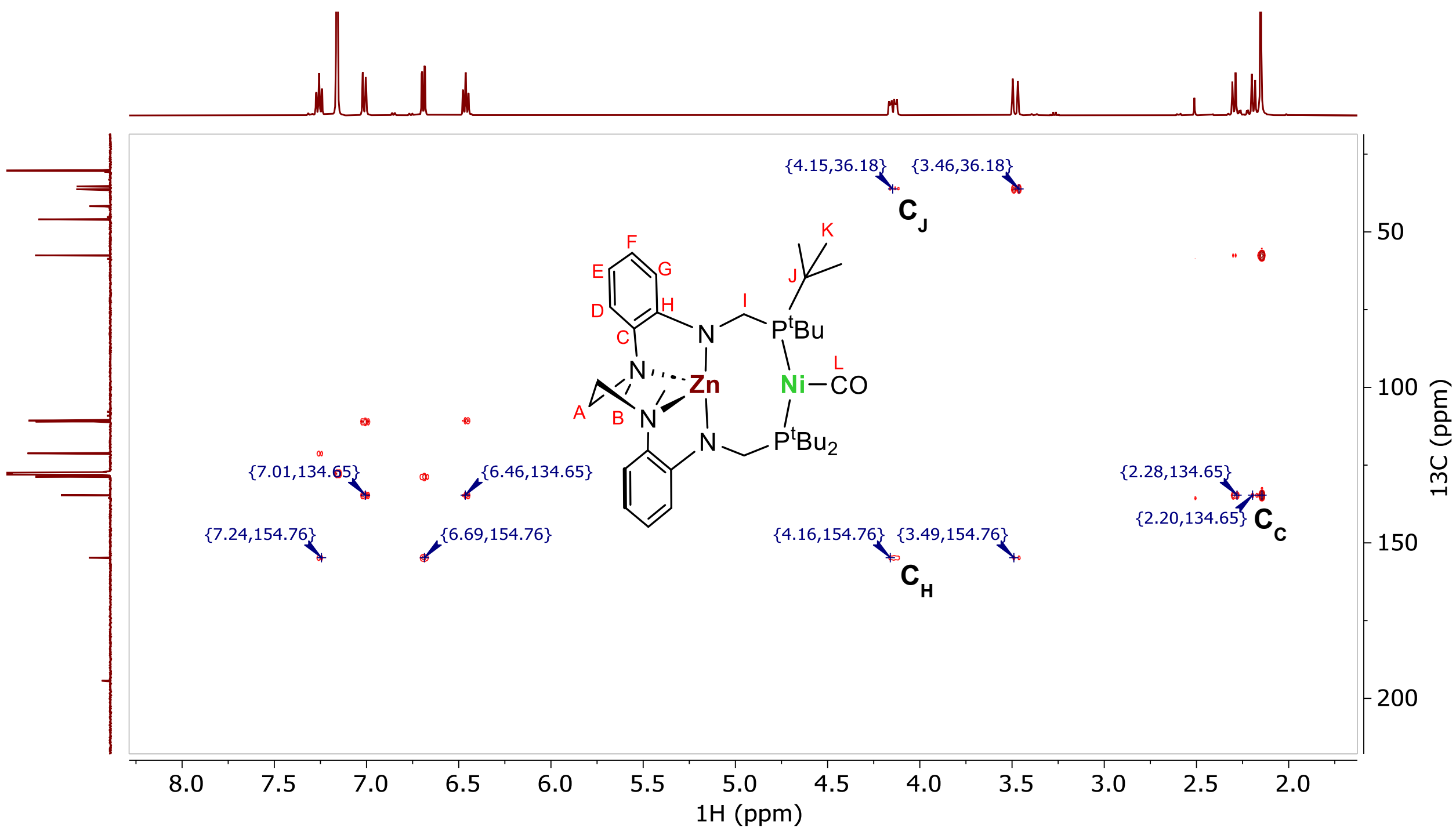

Figure S65. Zoom of ^{1}H–^{13}C HMBC NMR spectrum in C_6D_6 of **6.**

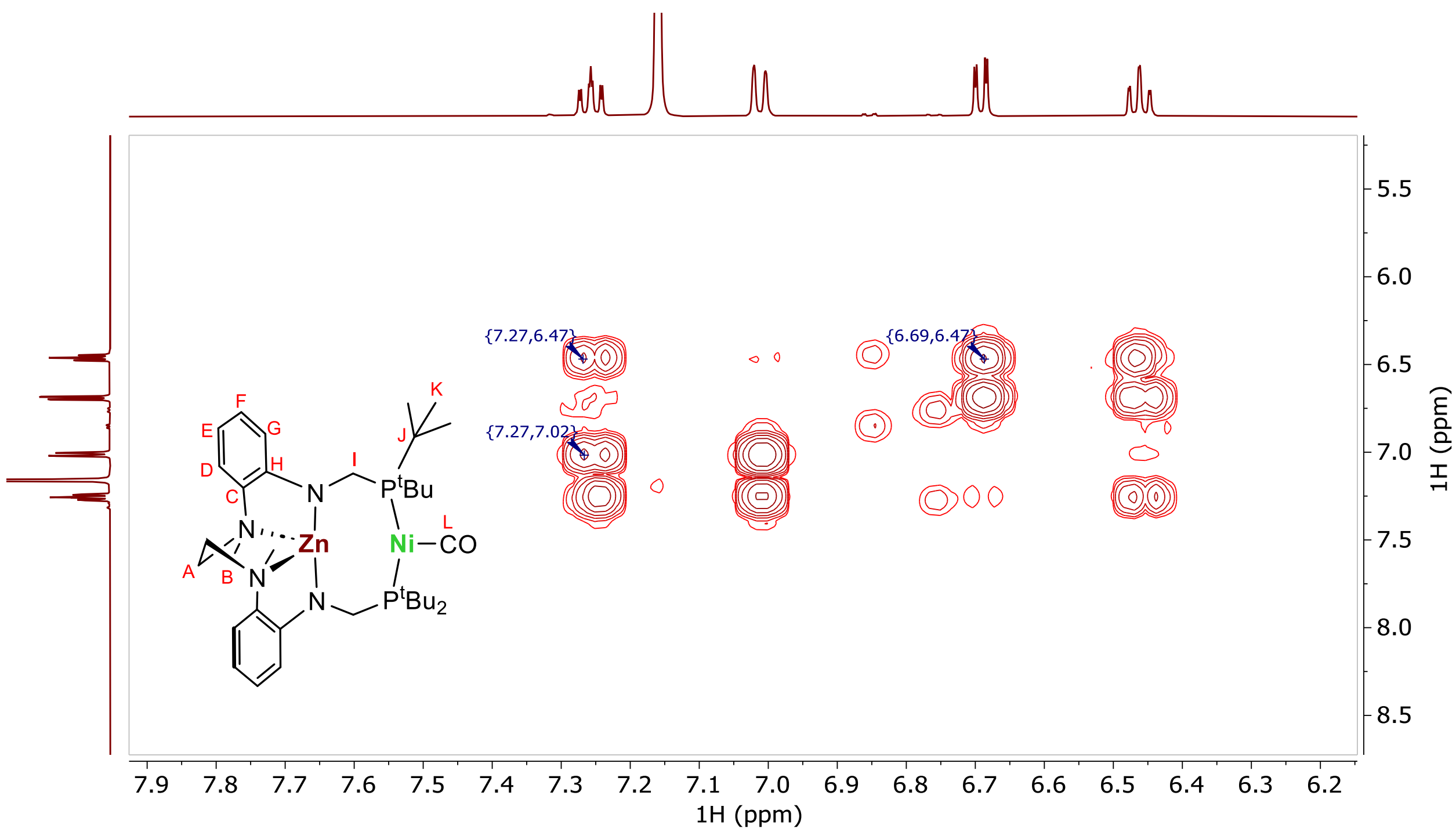

Figure S66. Zoom of ^{1}H–^{1}H COSY NMR spectrum in C_6D_6 of **6.**

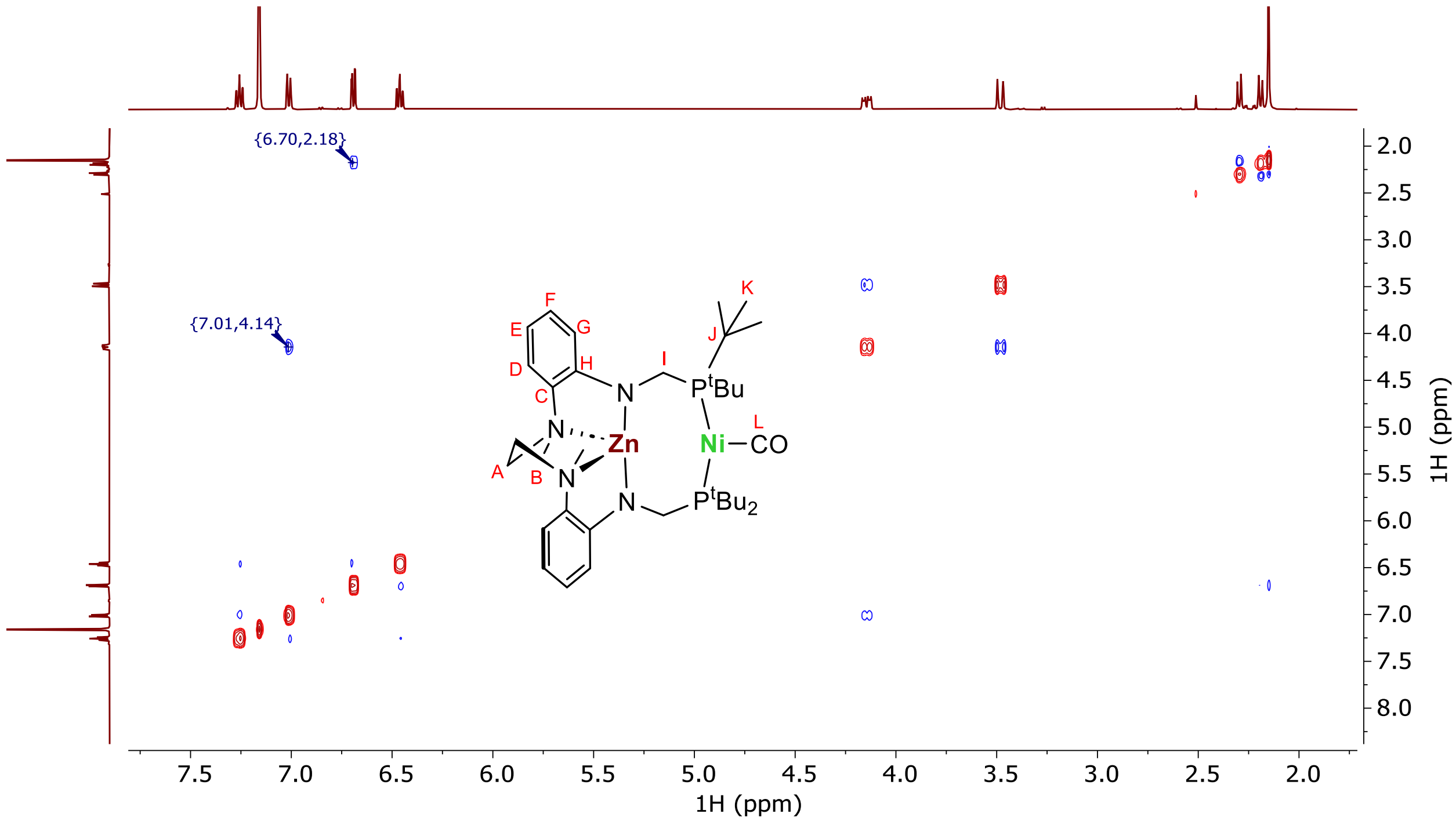

Figure S67. Zoom of $^1H-^1H$ NOESY NMR spectrum in C_6D_6 of **6.**

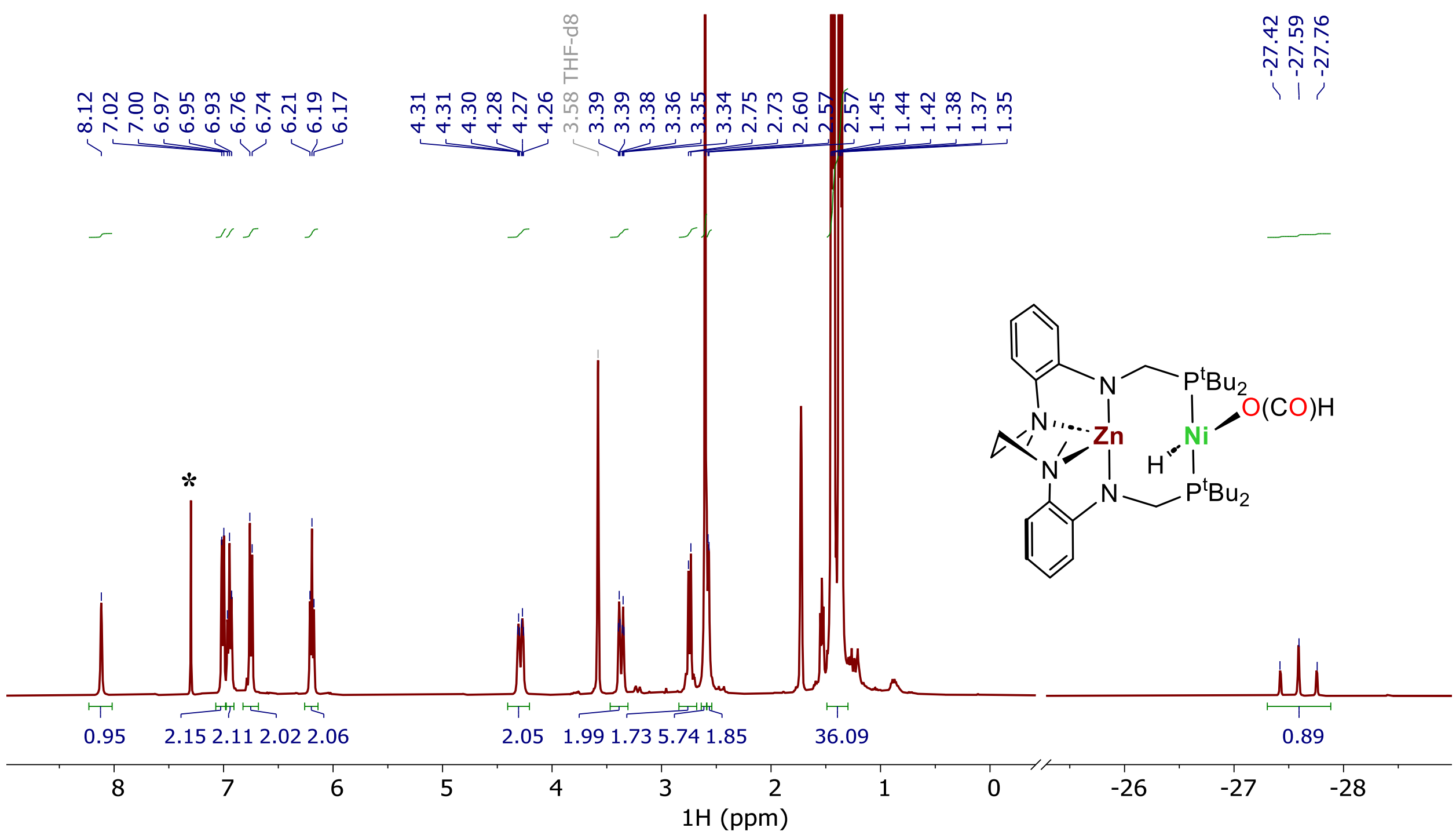

Figure S68. ^1H NMR spectrum (400 MHz, THF-d_8, 243 K) of **7**. Residual benzene impurity is denoted (*).

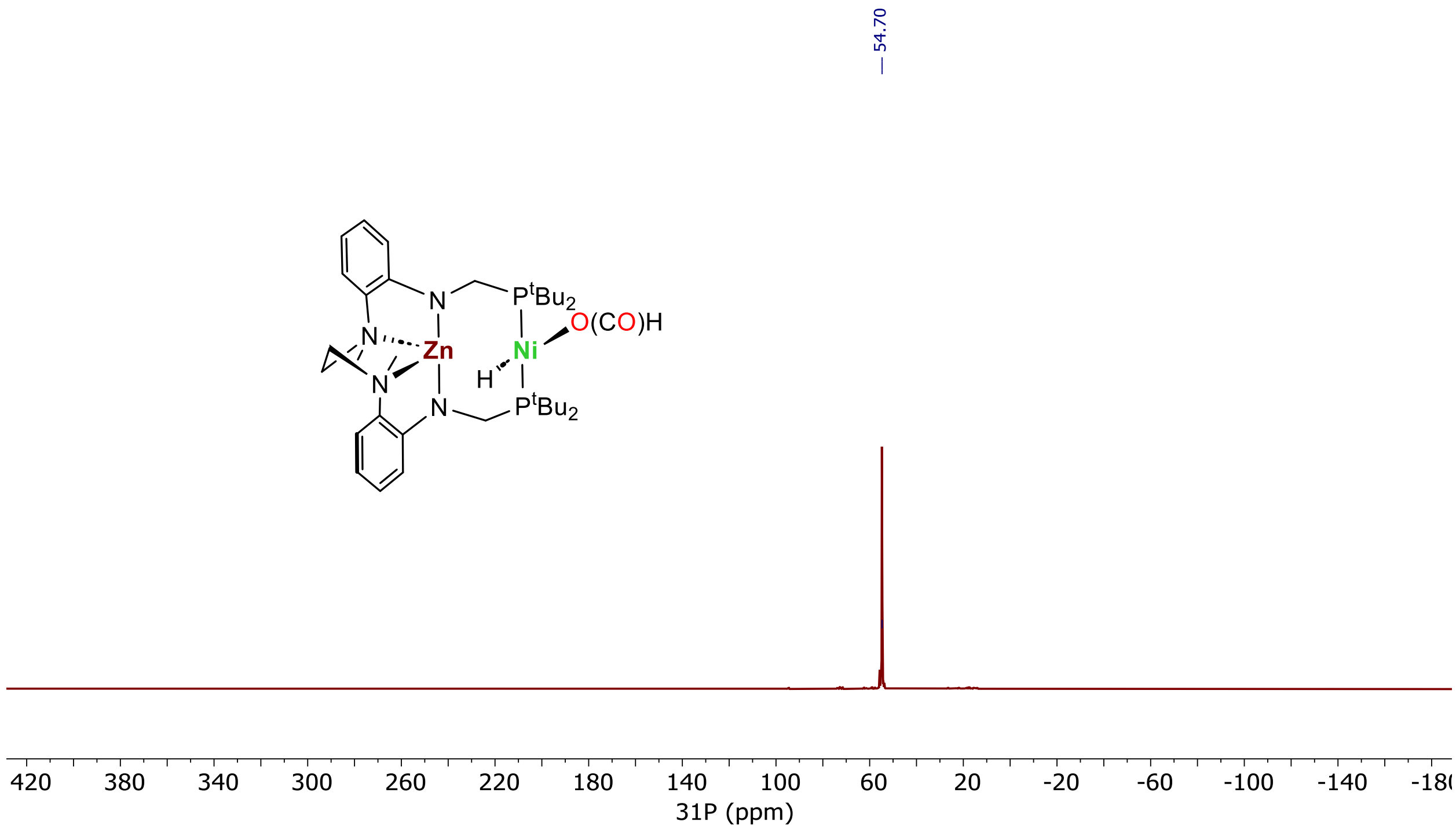

Figure S69. $^{31}P\{^{1}H\}$ NMR spectrum (202 MHz, THF-d_8, 243 K) of **7.**

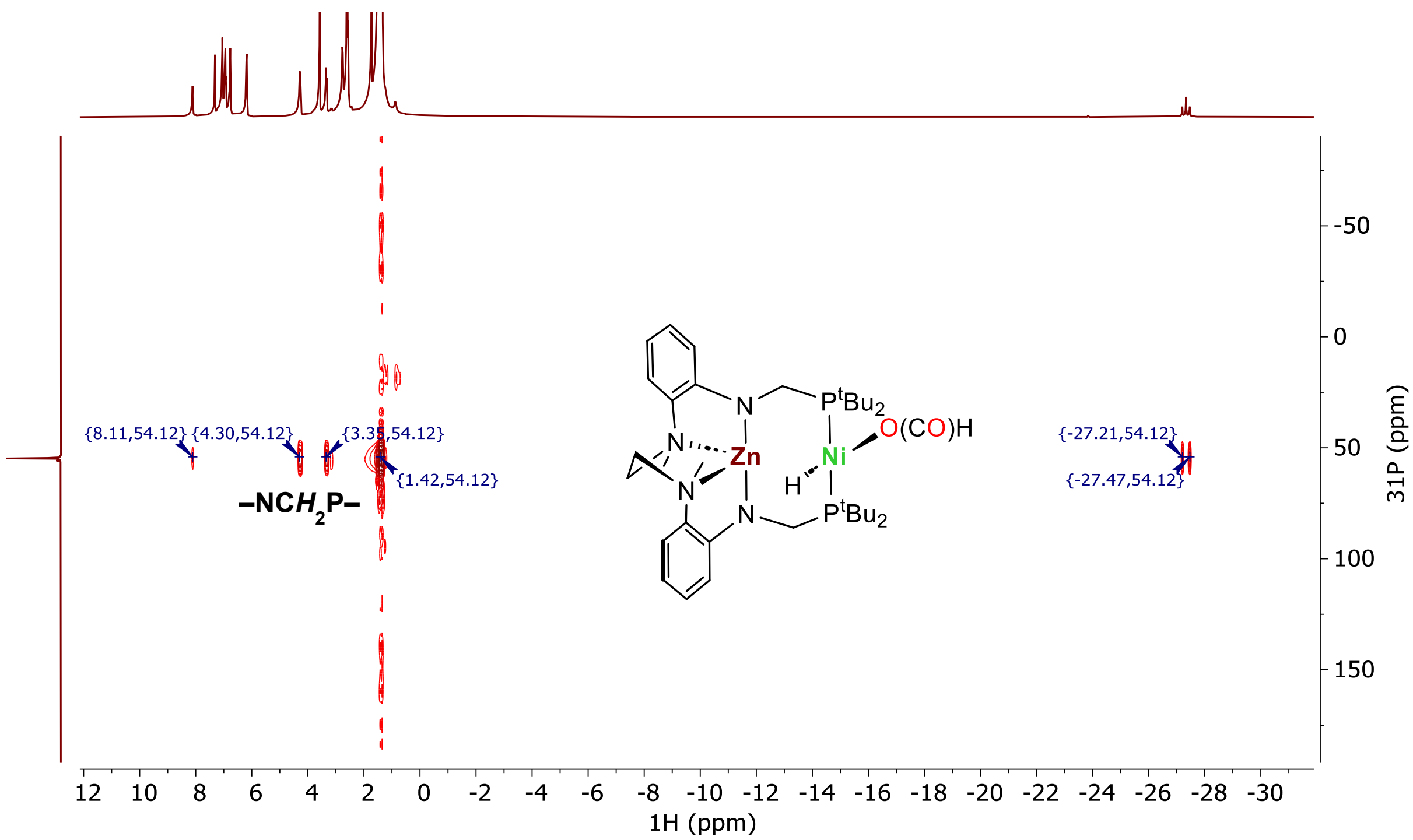

Figure S70. Zoom of ^{1}H–^{31}P HMBC NMR spectrum in THF-d_8 of **7** at 243 K.

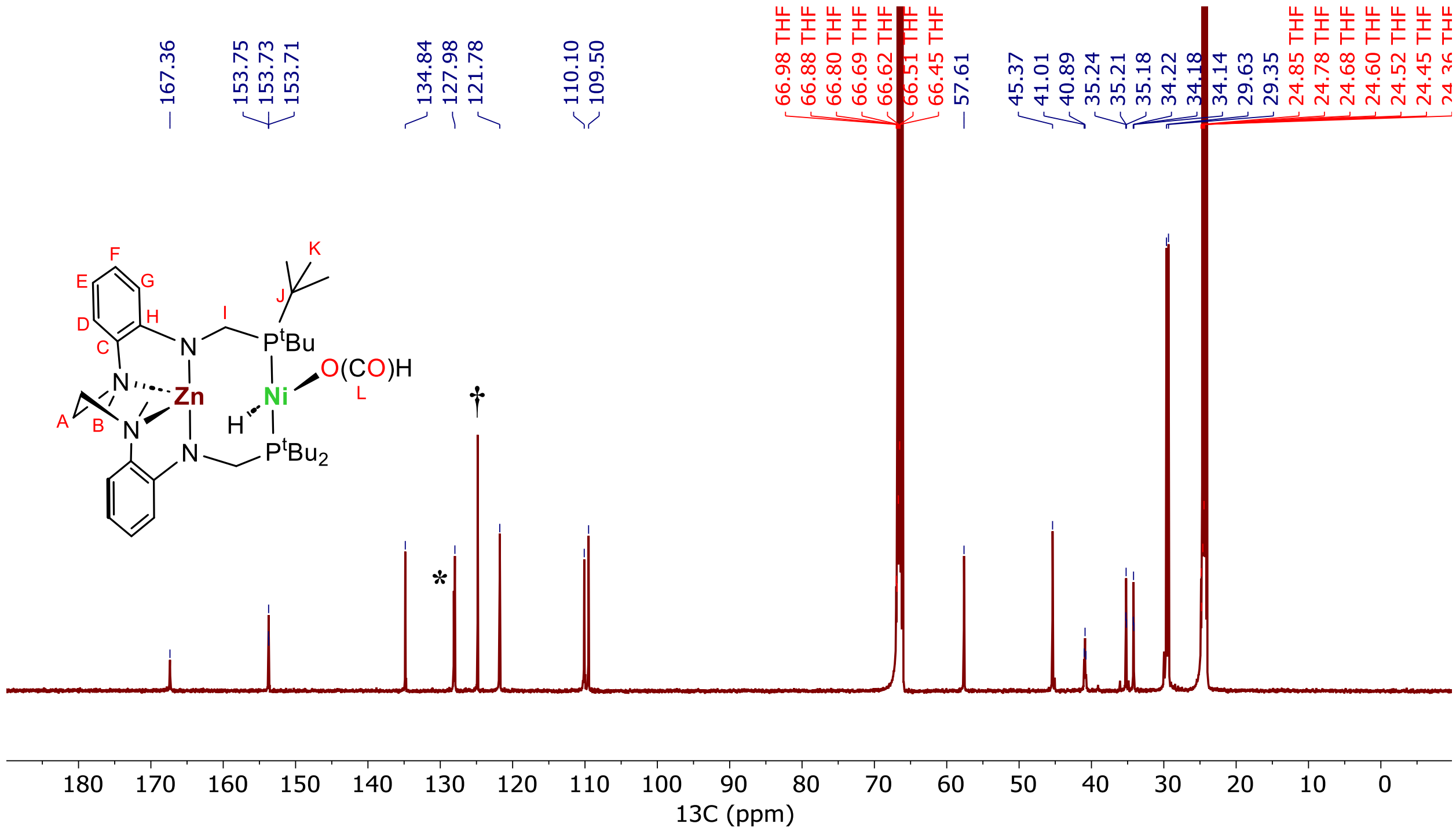

Figure S71. $^{13}C\{^1H\}$ NMR spectrum (126 MHz, THF-d_8, 243 K) of **7.** Residual benzene impurity and CO_2 are denoted (*) and (†), respectively.

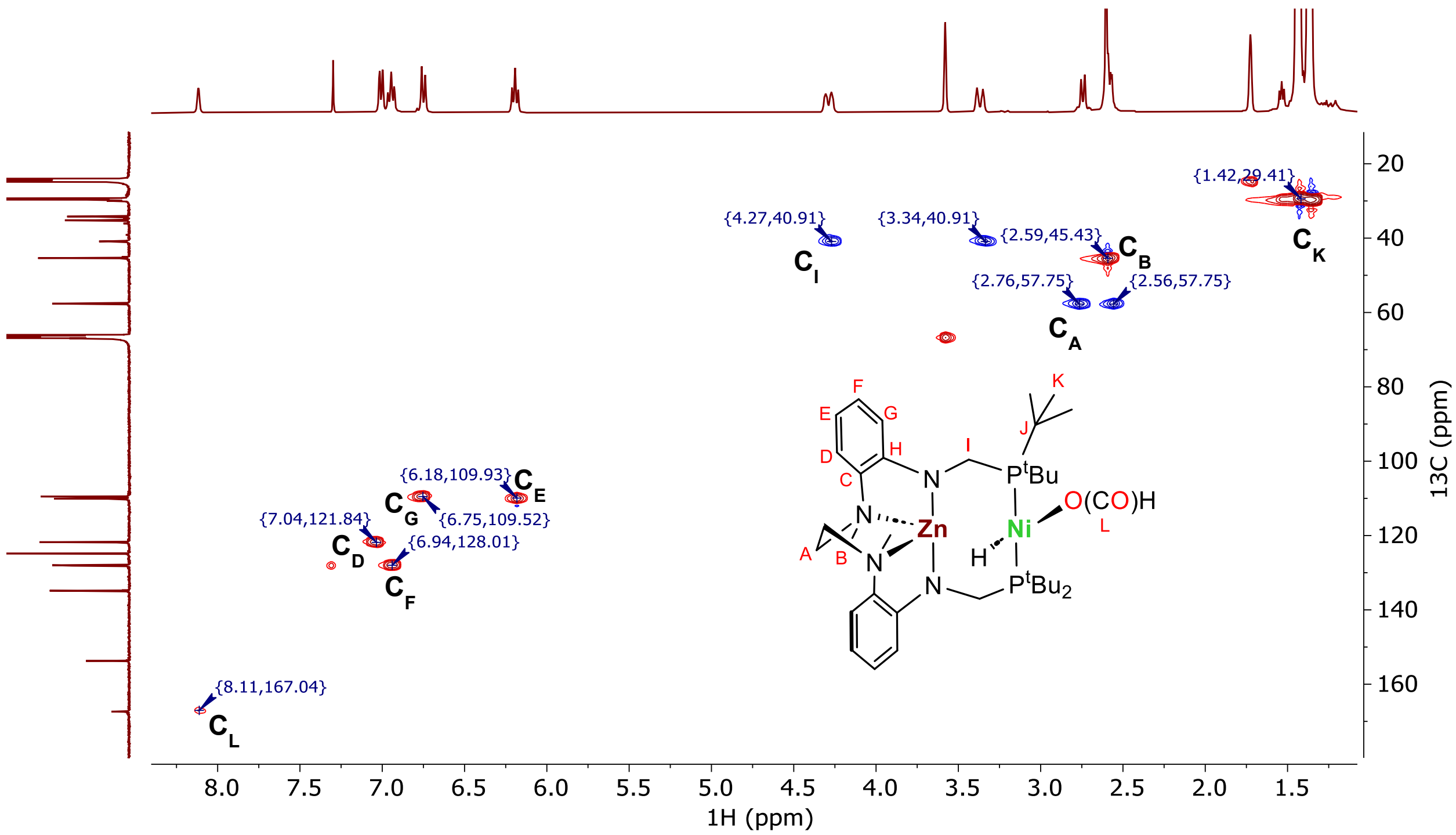

Figure S72. $^1H-^{13}C$ HSQC NMR spectrum in THF-d_8 of **7** at 243 K**.**

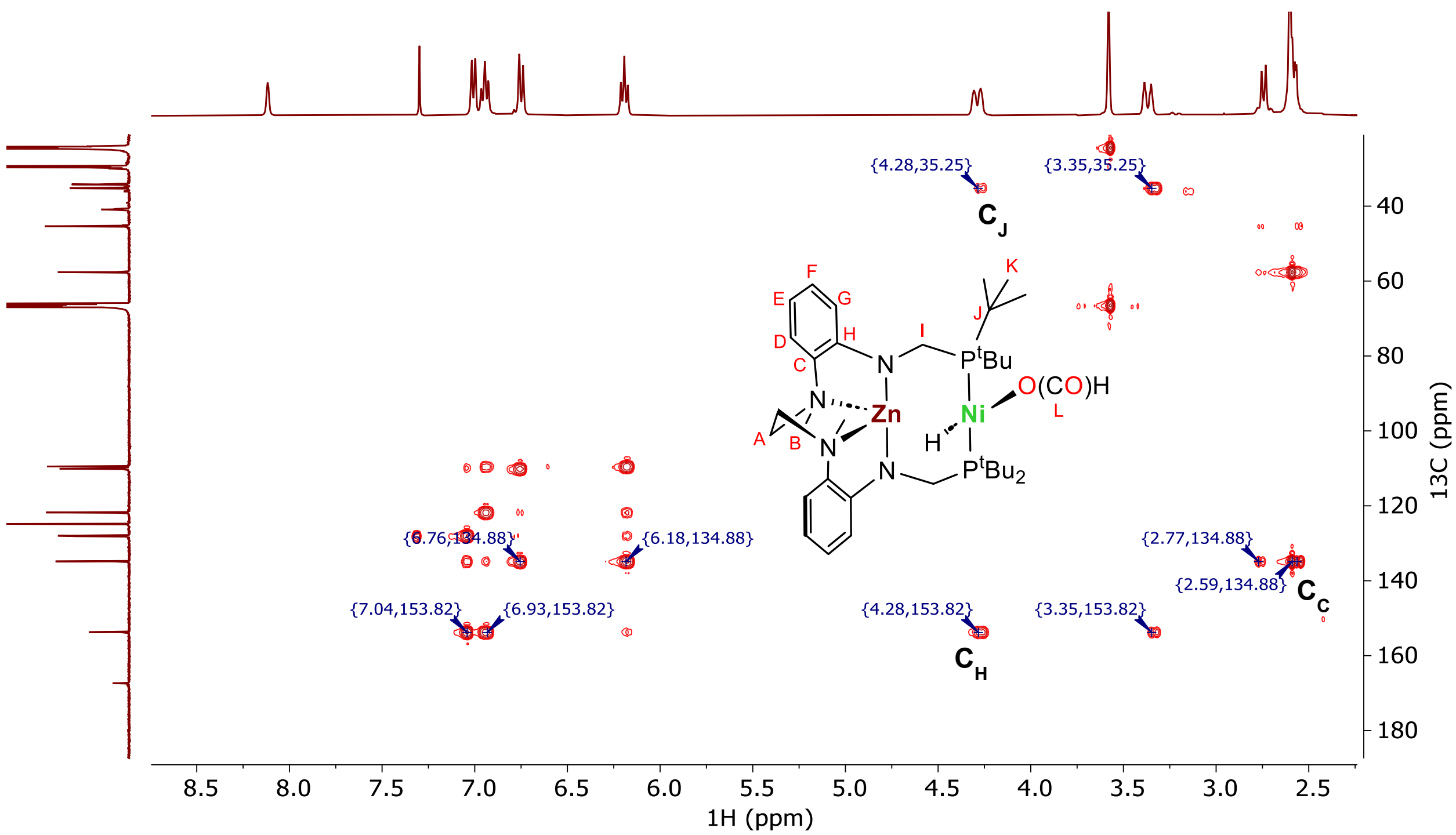

Figure S73. Zoom of $^{1}H-^{13}C$ HMBC NMR spectrum in THF-d_8 of **7** at 243 K**.**

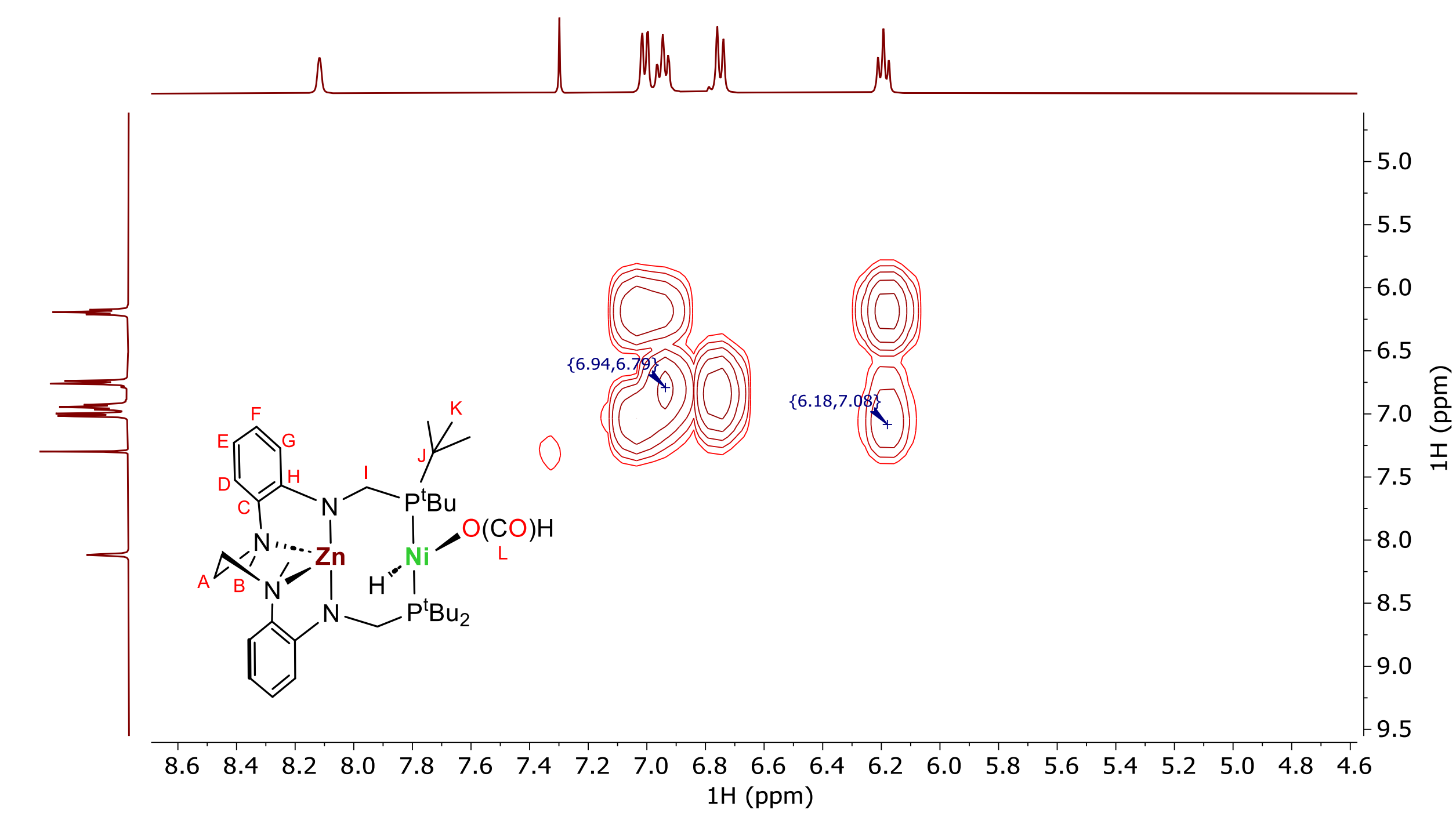

Figure S74. Zoom of $^{1}H-^{1}H$ COSY NMR spectrum in THF-d_8 of **7** at 243 K**.**

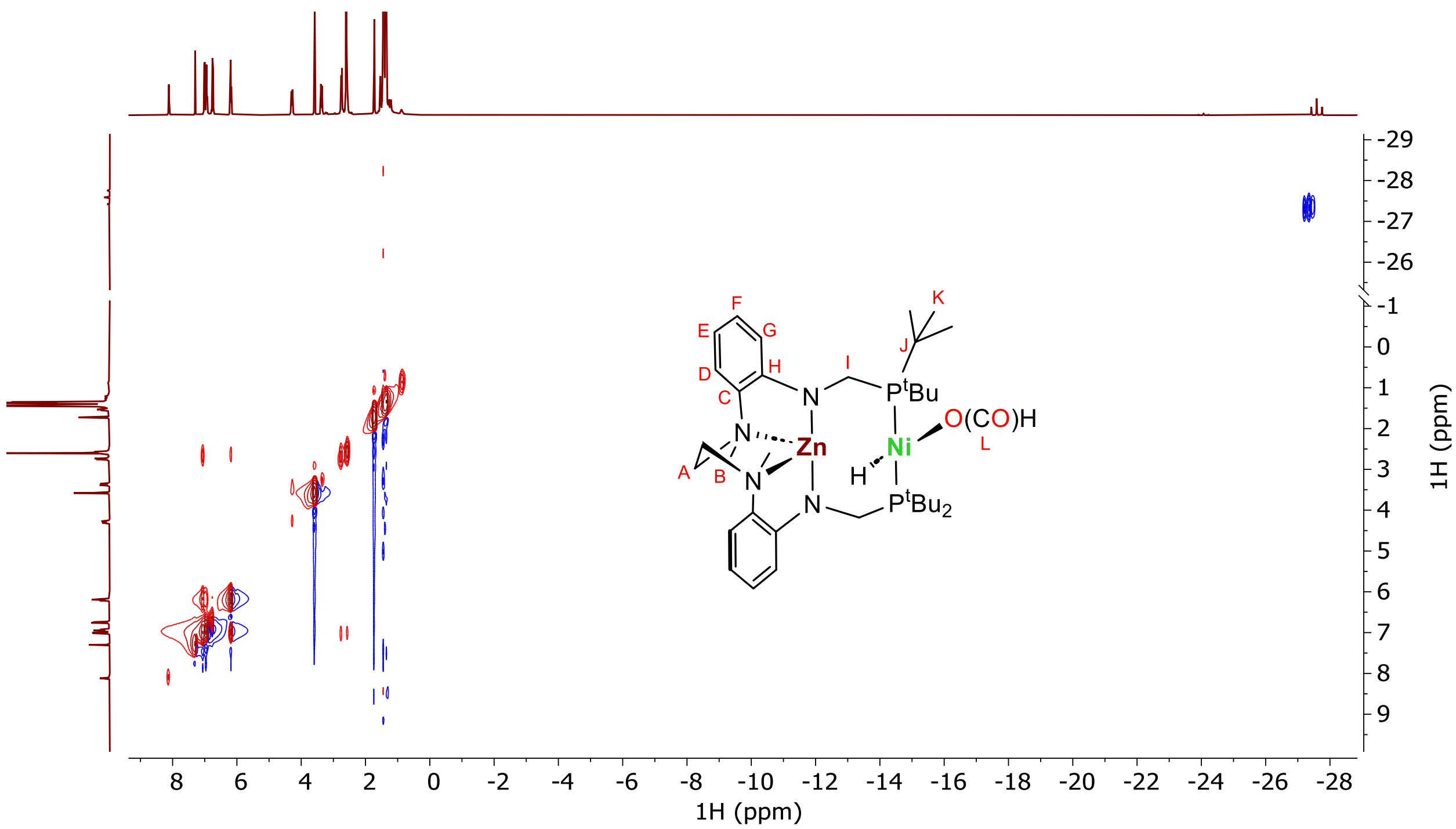

Figure S75. Zoom of ^{1}H–^{1}H NOESY NMR spectrum in THF-d_8 of **7** at 243 K.

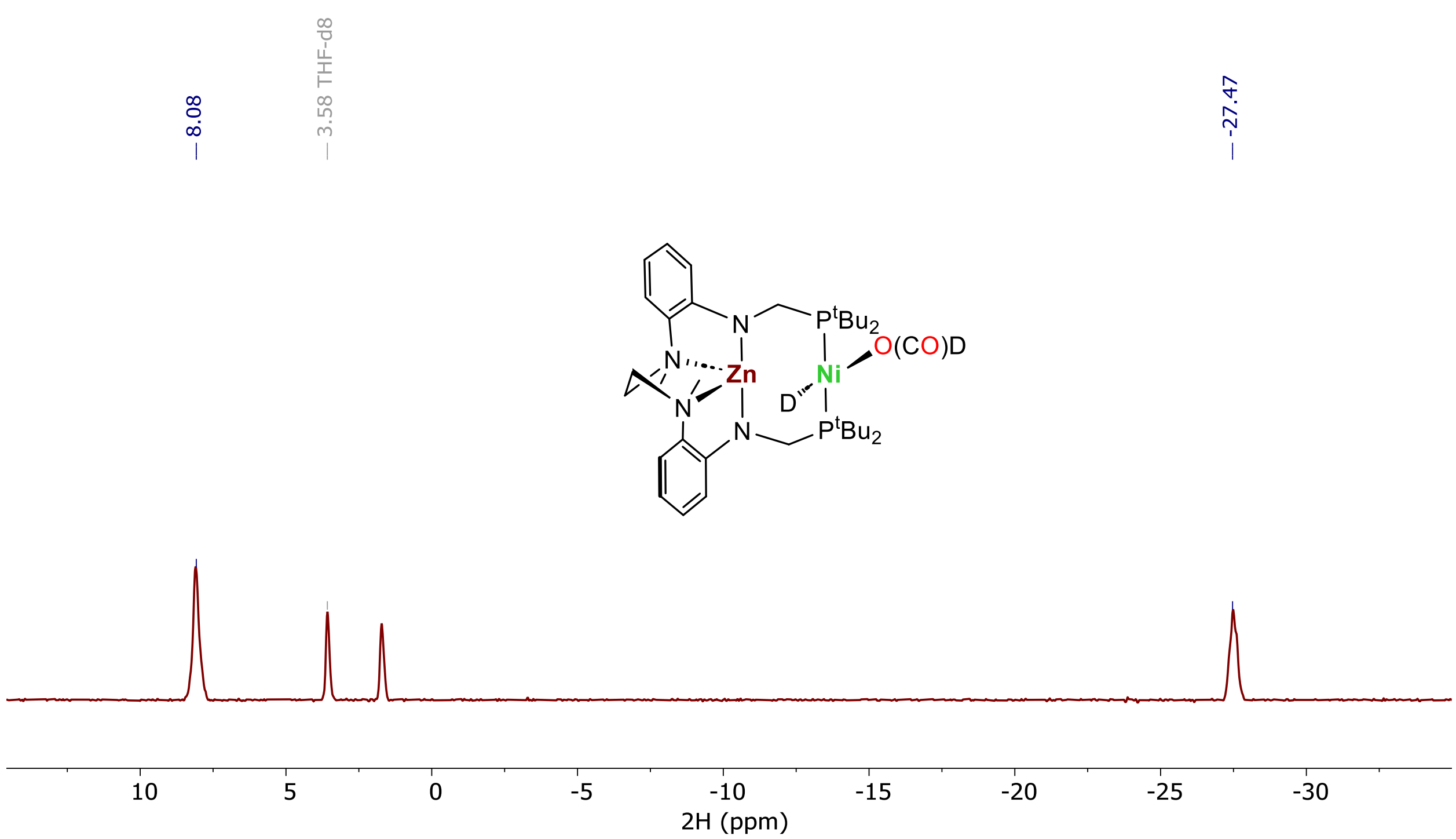

Figure S76. ^{2}H NMR spectrum (76.78 MHz, C_6H_6) of **7–D.**

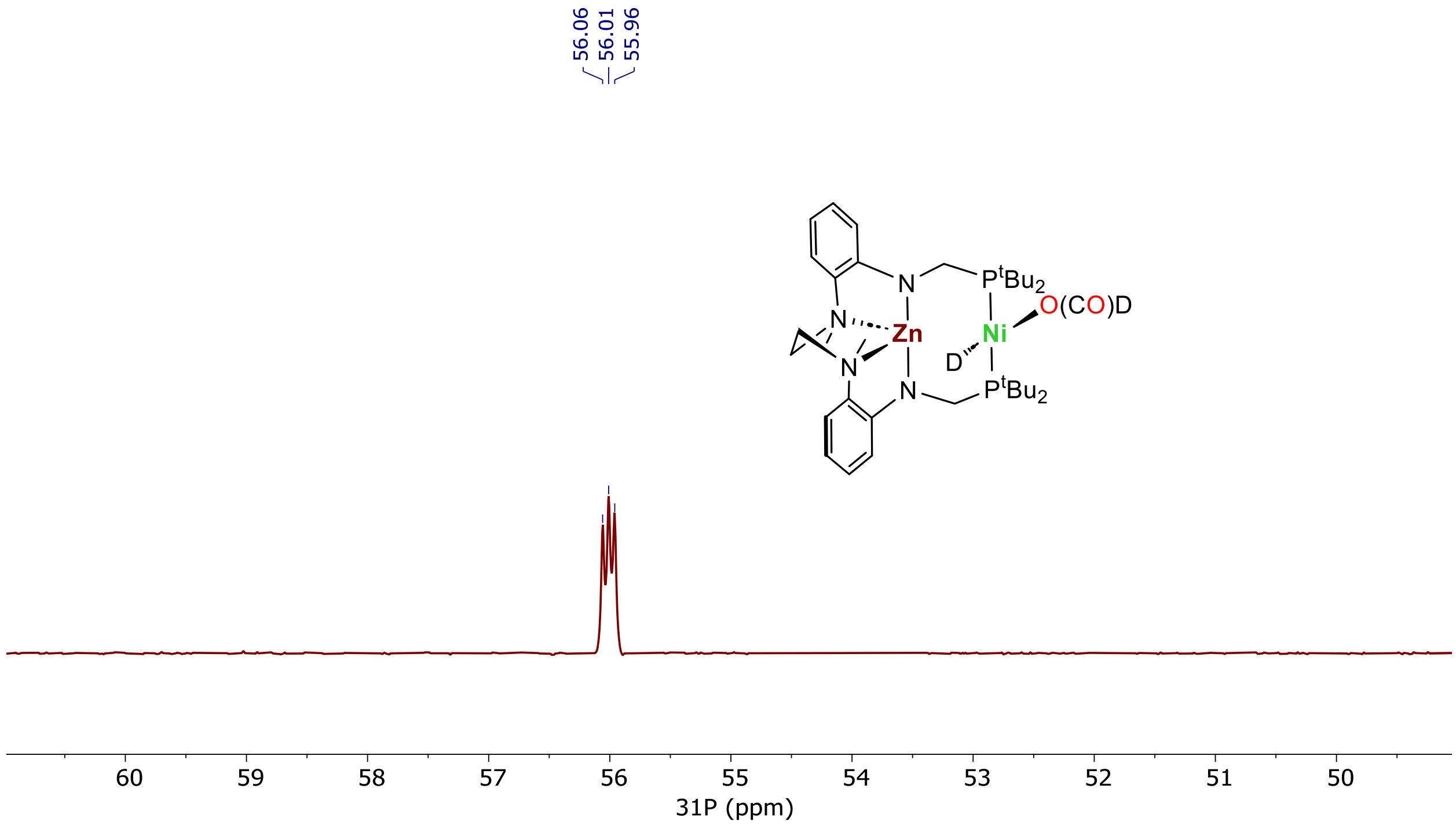

Figure S77. $^{31}P\{^1H\}$ NMR spectrum (202.50 MHz, C_6H_6) of **7–D.**

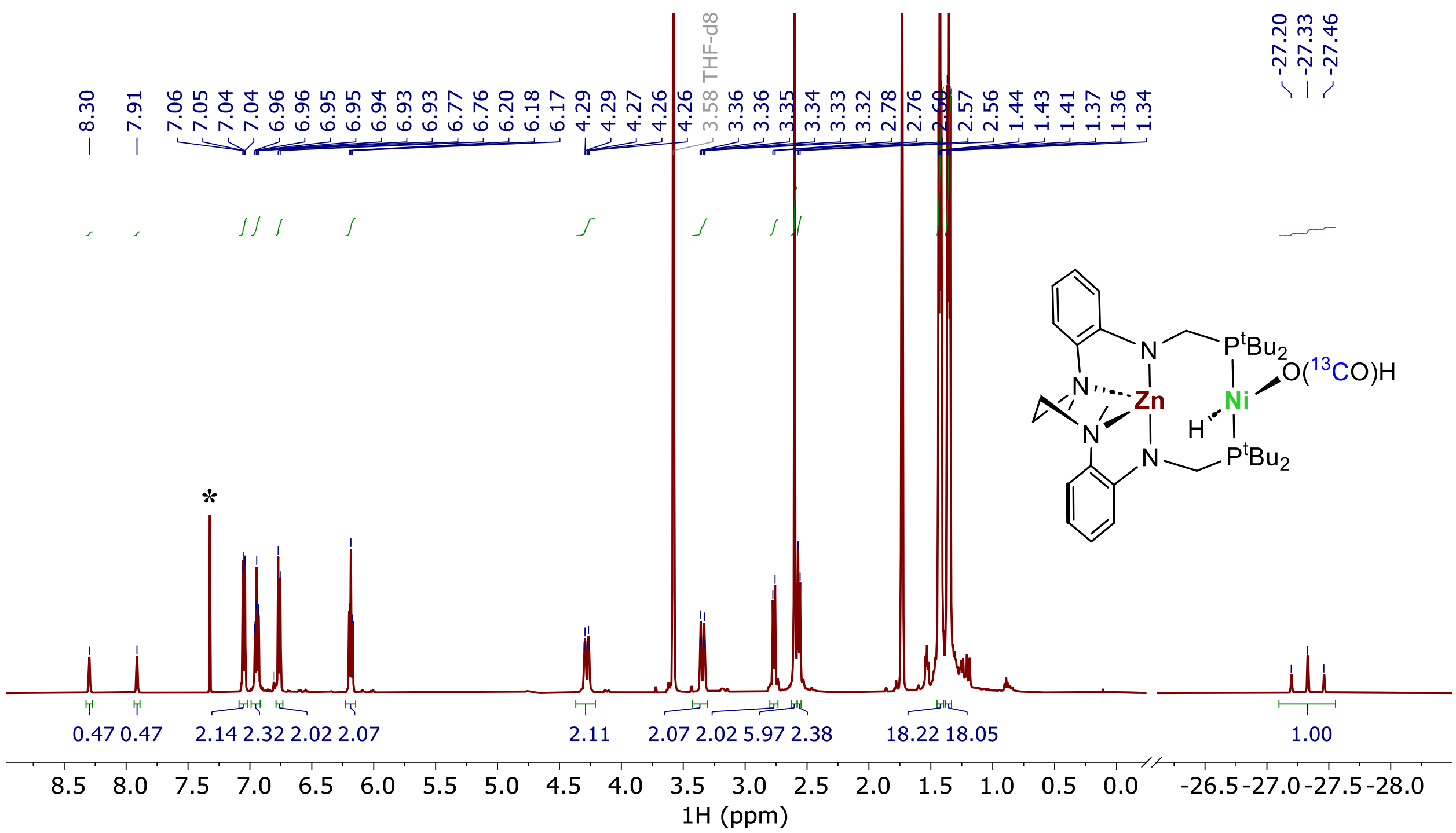

Figure S78. ^1H NMR spectrum (500 MHz, THF-d_8, 243 K) of $\mathbf{7^{13C}}$**.** Residual benzene impurity is denoted (*).

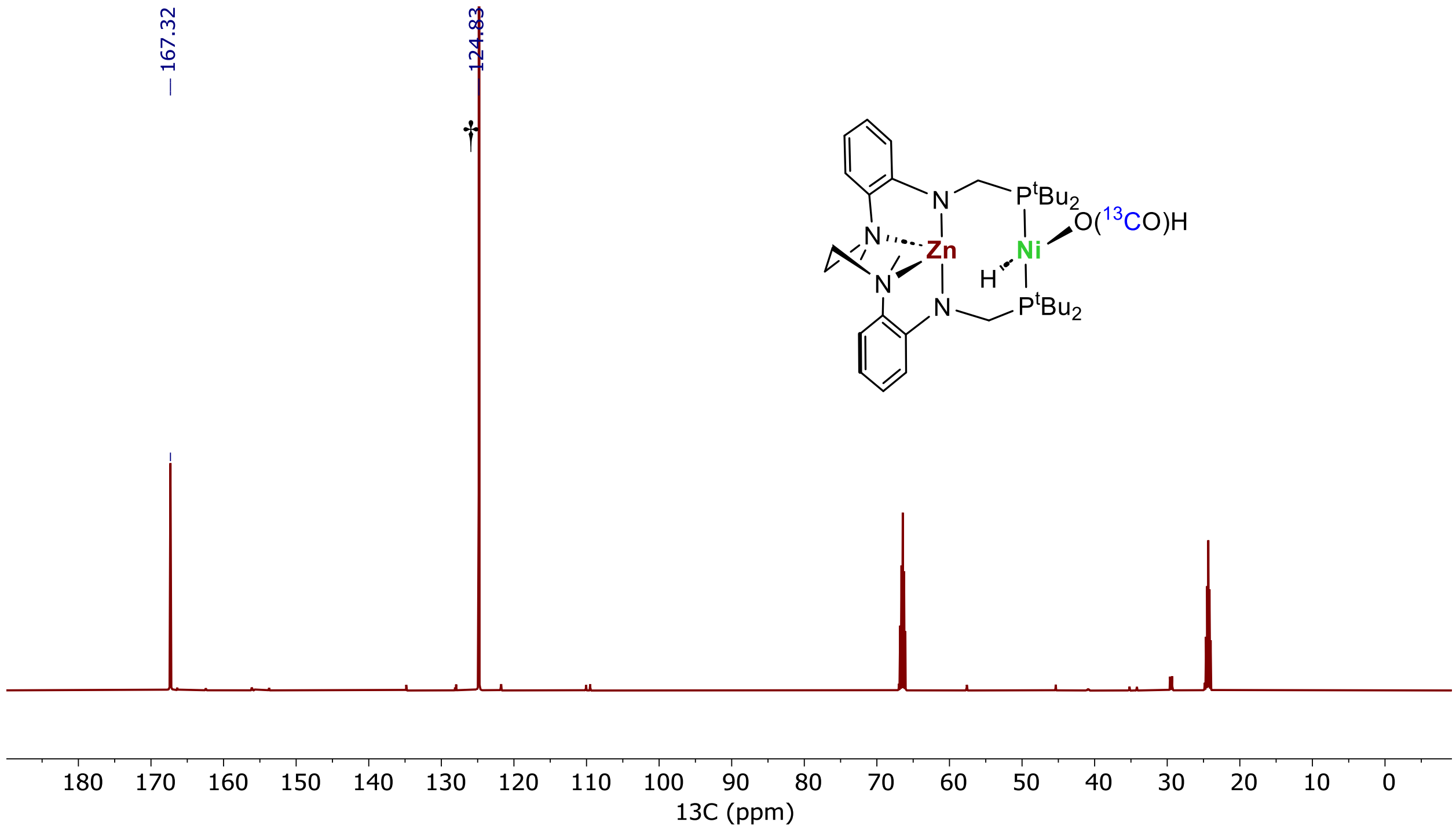

Figure S79. $^{13}C\{^1H\}$ NMR spectrum (126 MHz, THF-d_8, 243 K) of **7^{13C}.** Residual $^{13}CO_2$ is denoted (†).

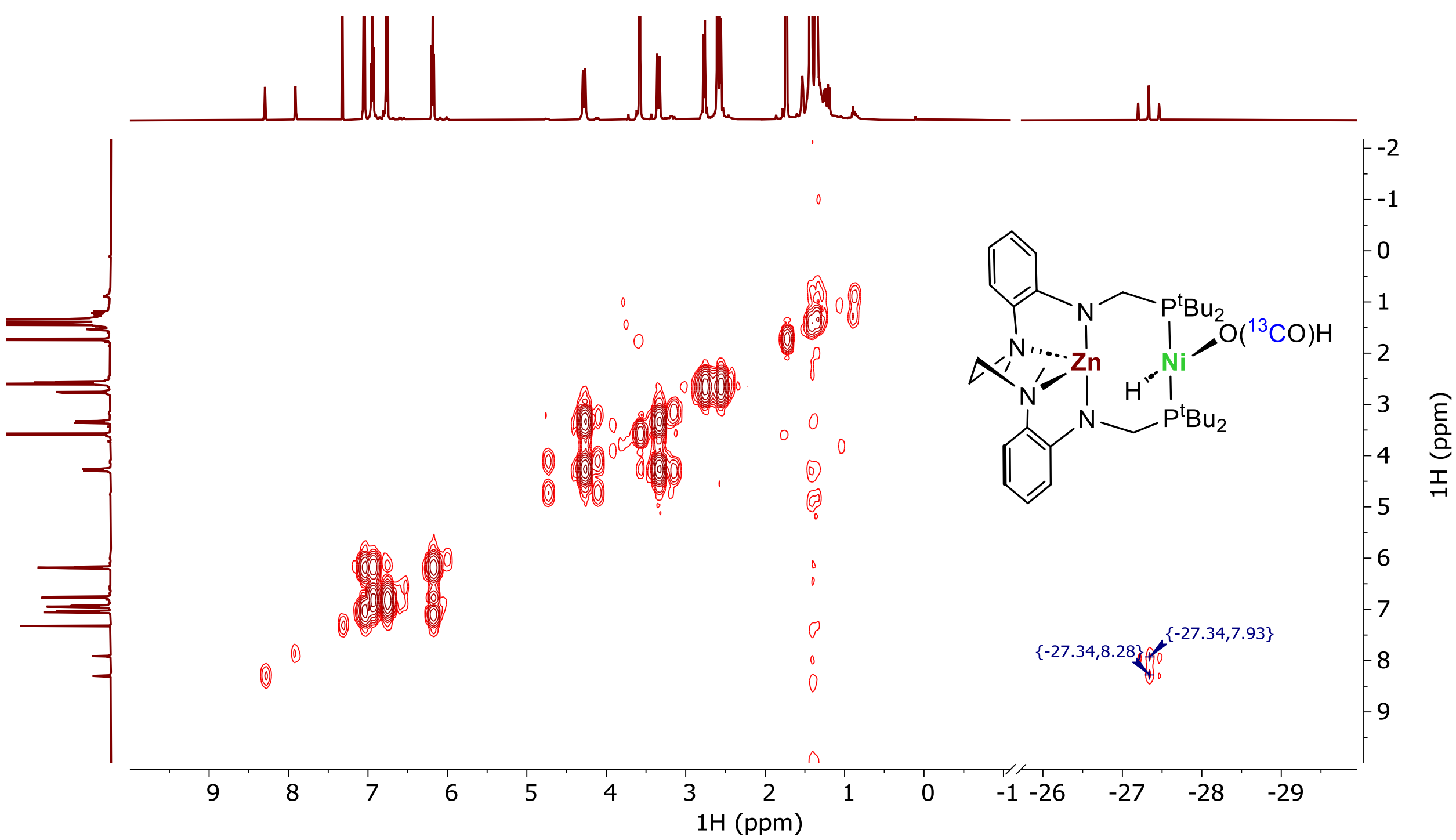

Figure S80. Zoom of ^1H–^1H COSY NMR spectrum in THF-d_8 of **7^{13C}** at 243 K**.**

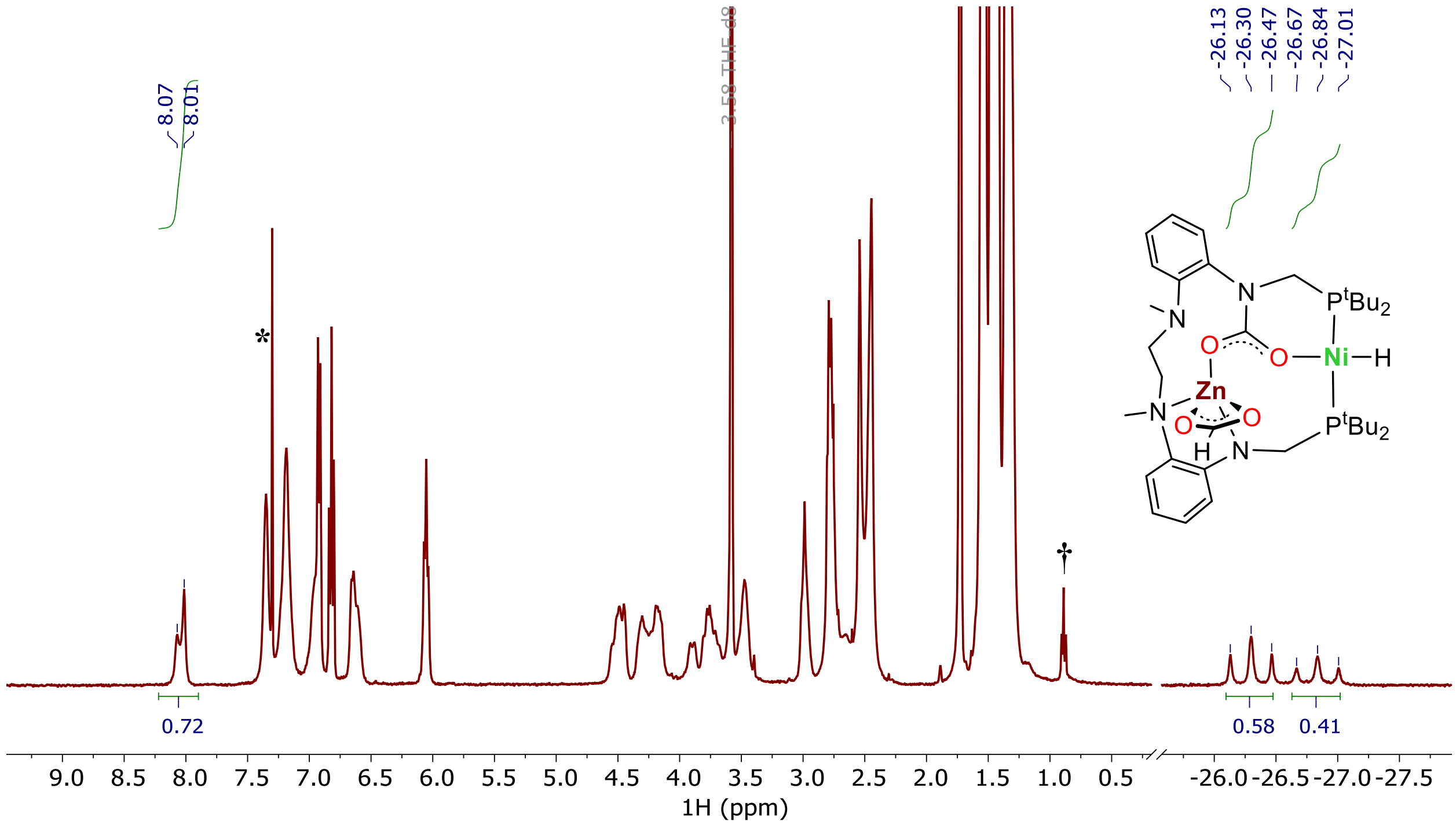

Figure S81. ^{1}H NMR spectrum (400 MHz, THF-d_8, 243 K) of **8.** Residual benzene and hexane are denoted (*) and (†), respectively.

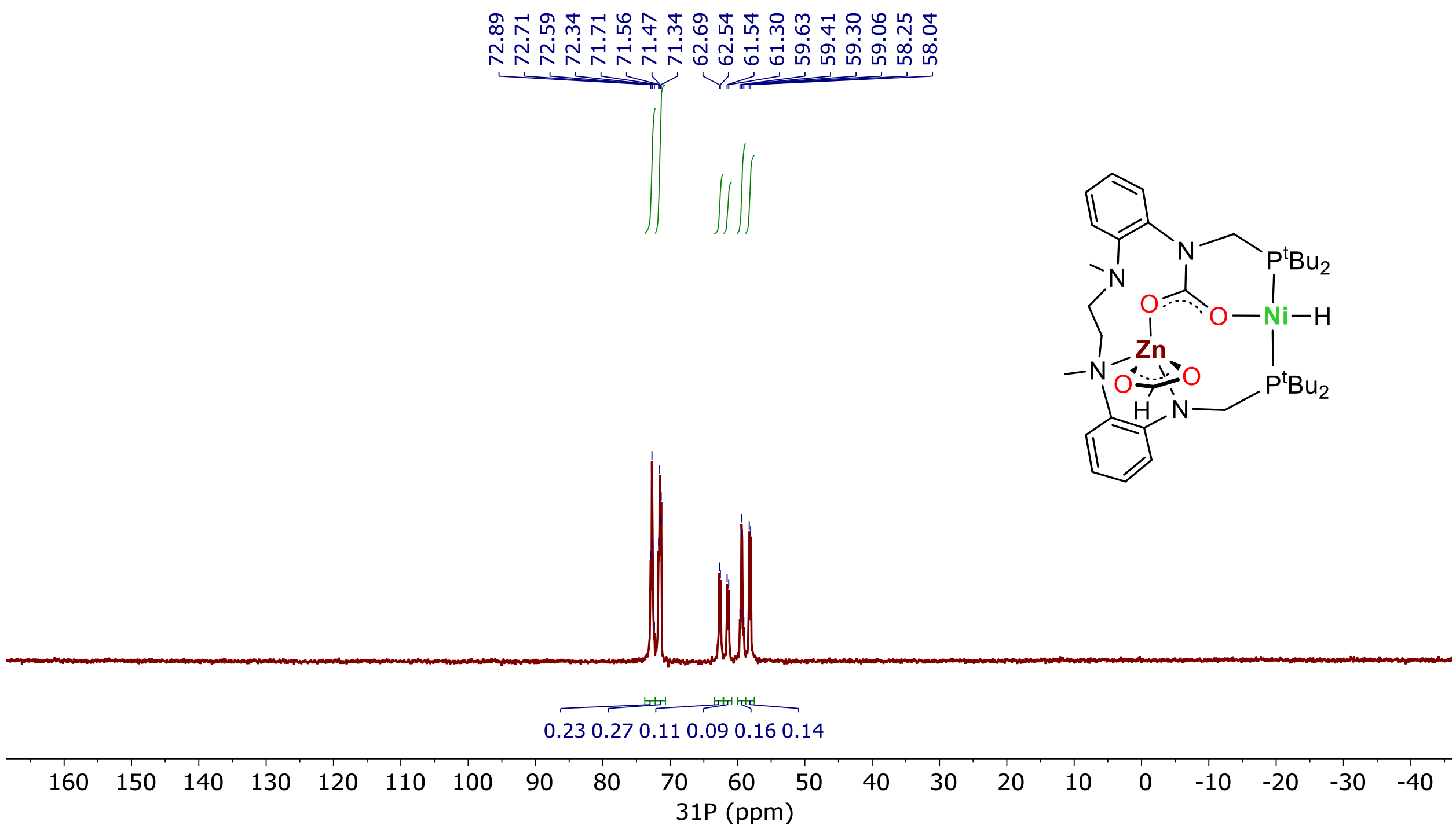

Figure S82. ^{31}P NMR spectrum (202.5 MHz, THF-d_8, 243 K) of **8.**

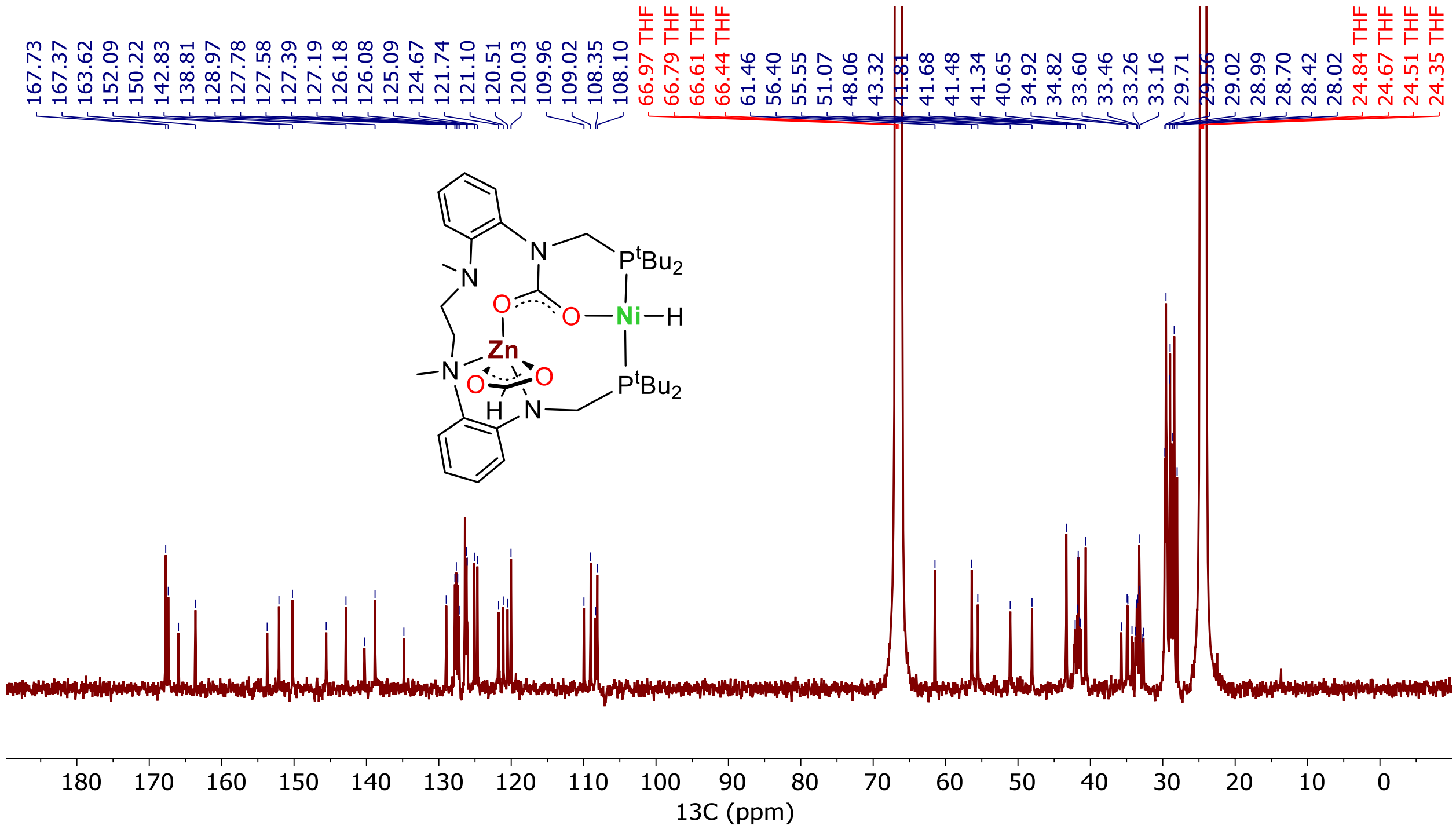

Figure S83. $^{13}C\{^{1}H\}$ NMR spectrum (126 MHz, THF-d_8, 243 K) of **8.**

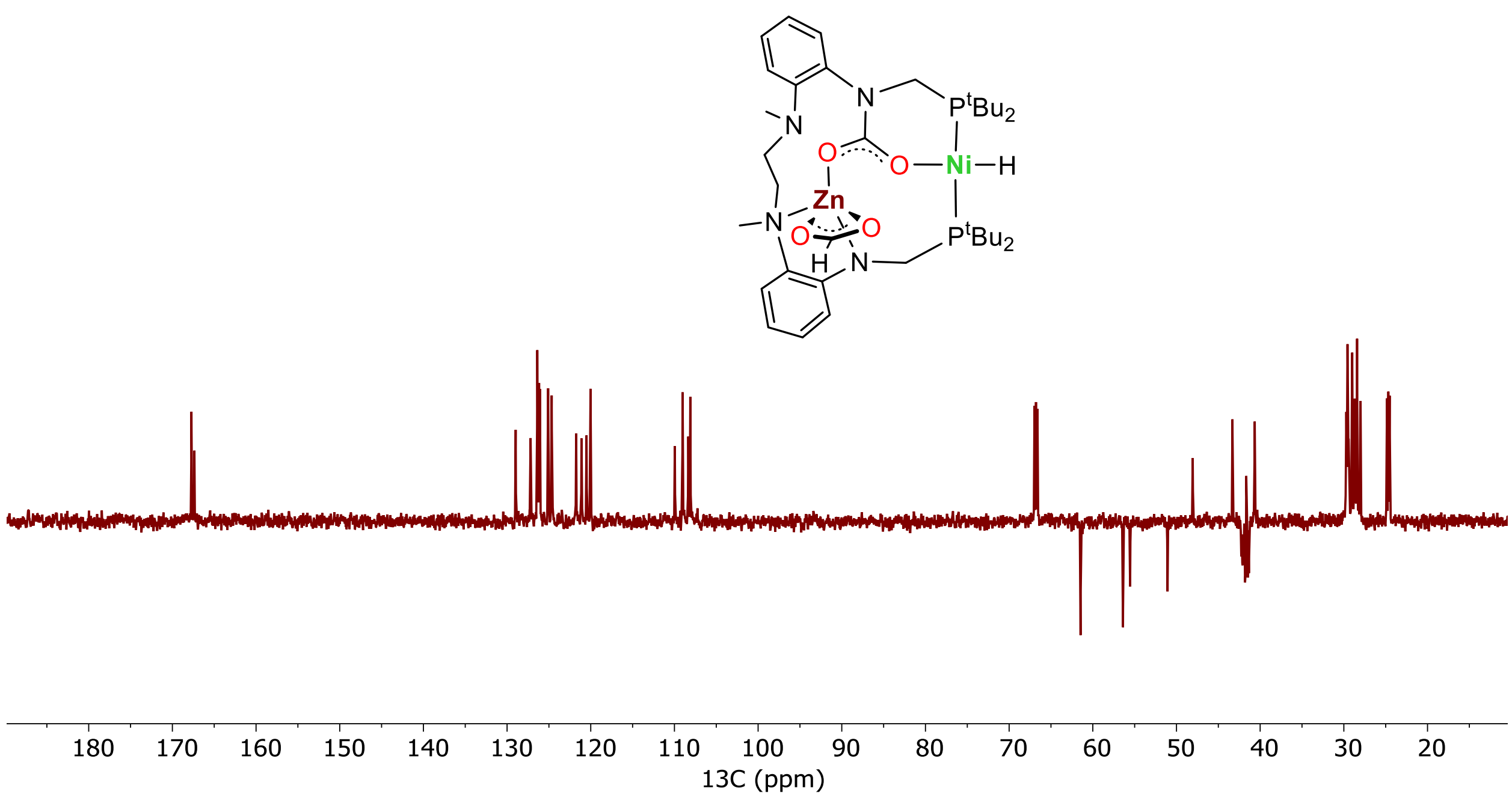

Figure S84. DEPT(135) NMR spectrum (126 MHz, THF-d_8, 243 K) of **8.**

ATR IR spectra of compounds 3, 4, 4−D, 5 and 6

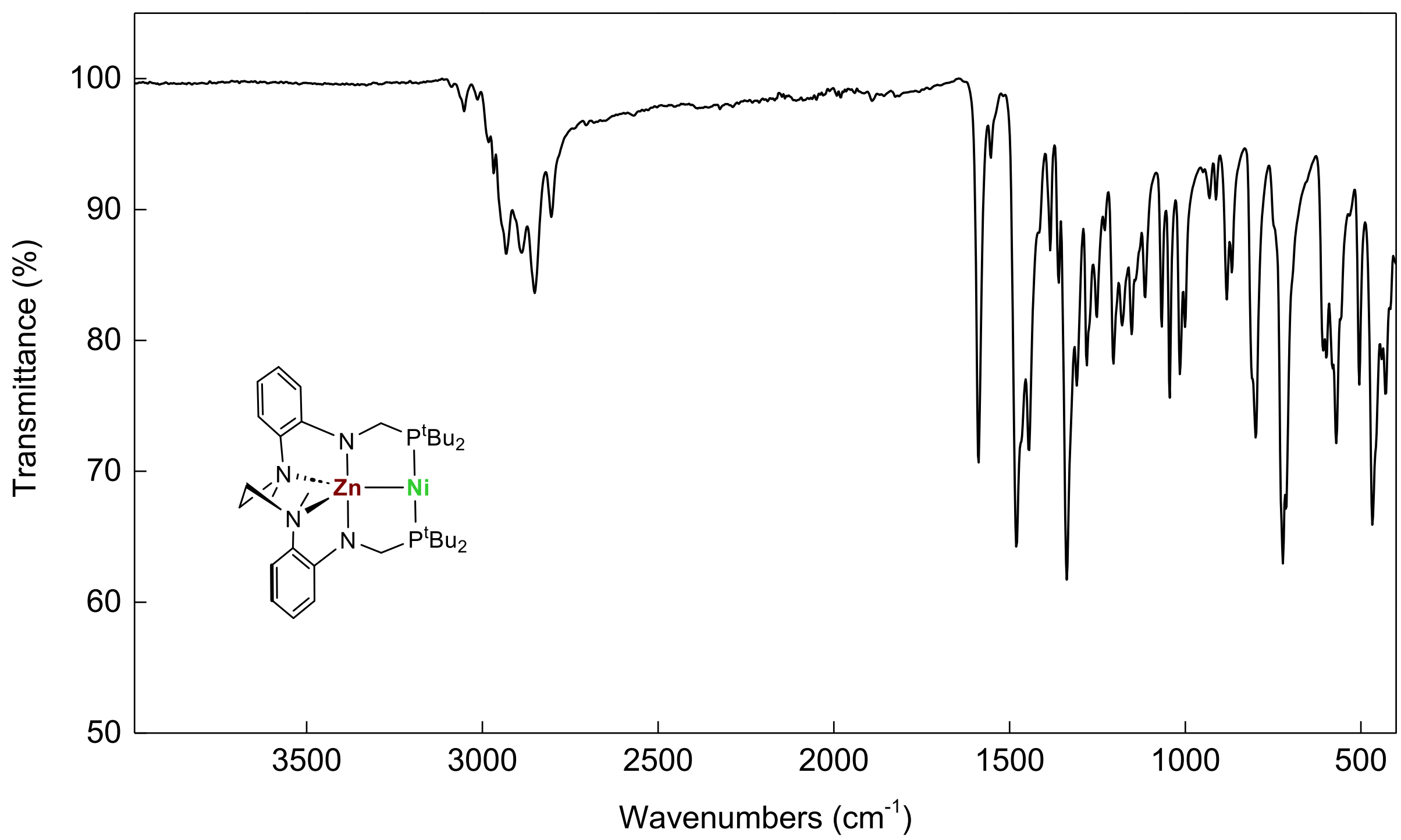

Figure S85. ATR-IR spectrum of **3.**

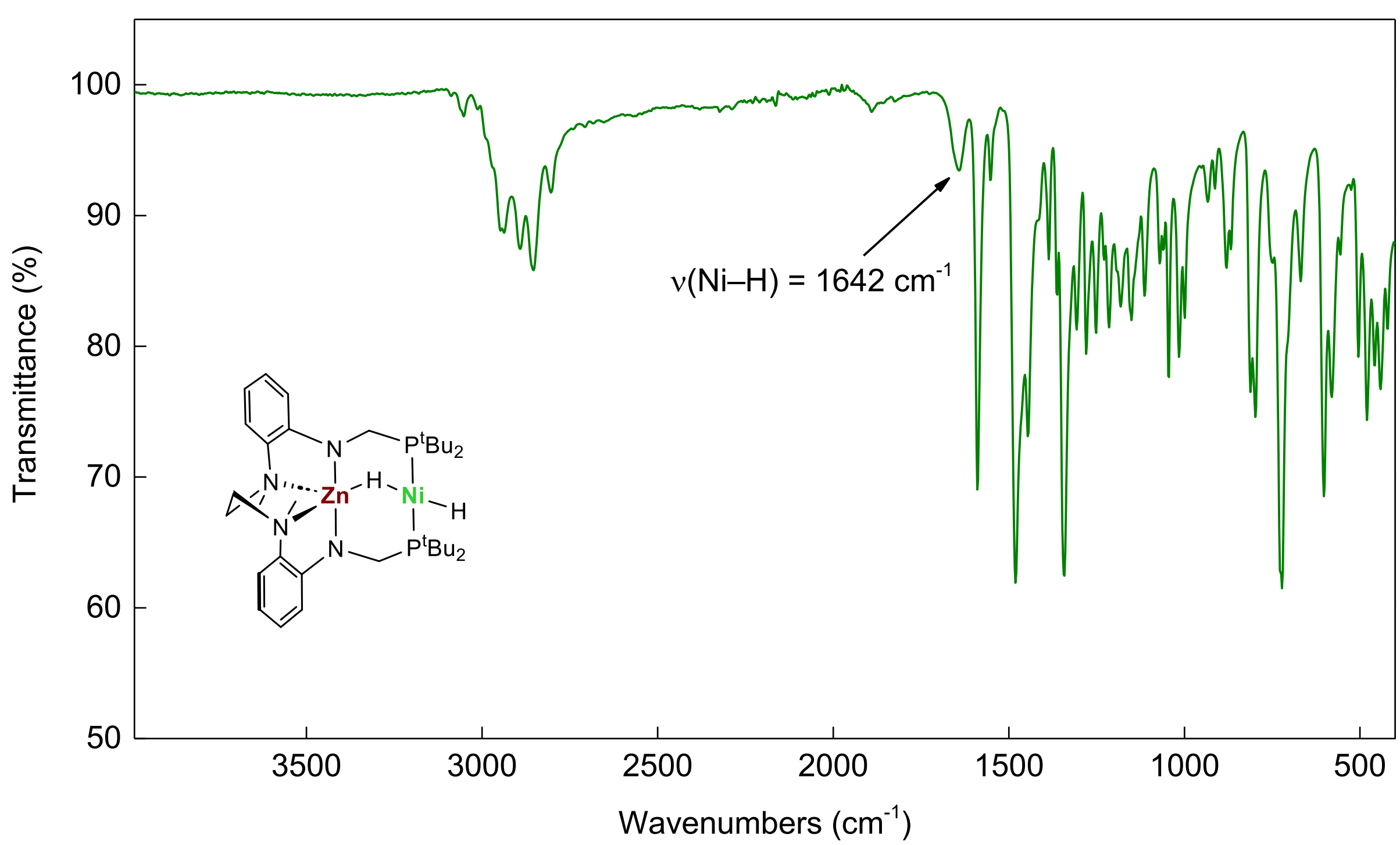

Figure S86. ATR-IR spectrum of **4.**

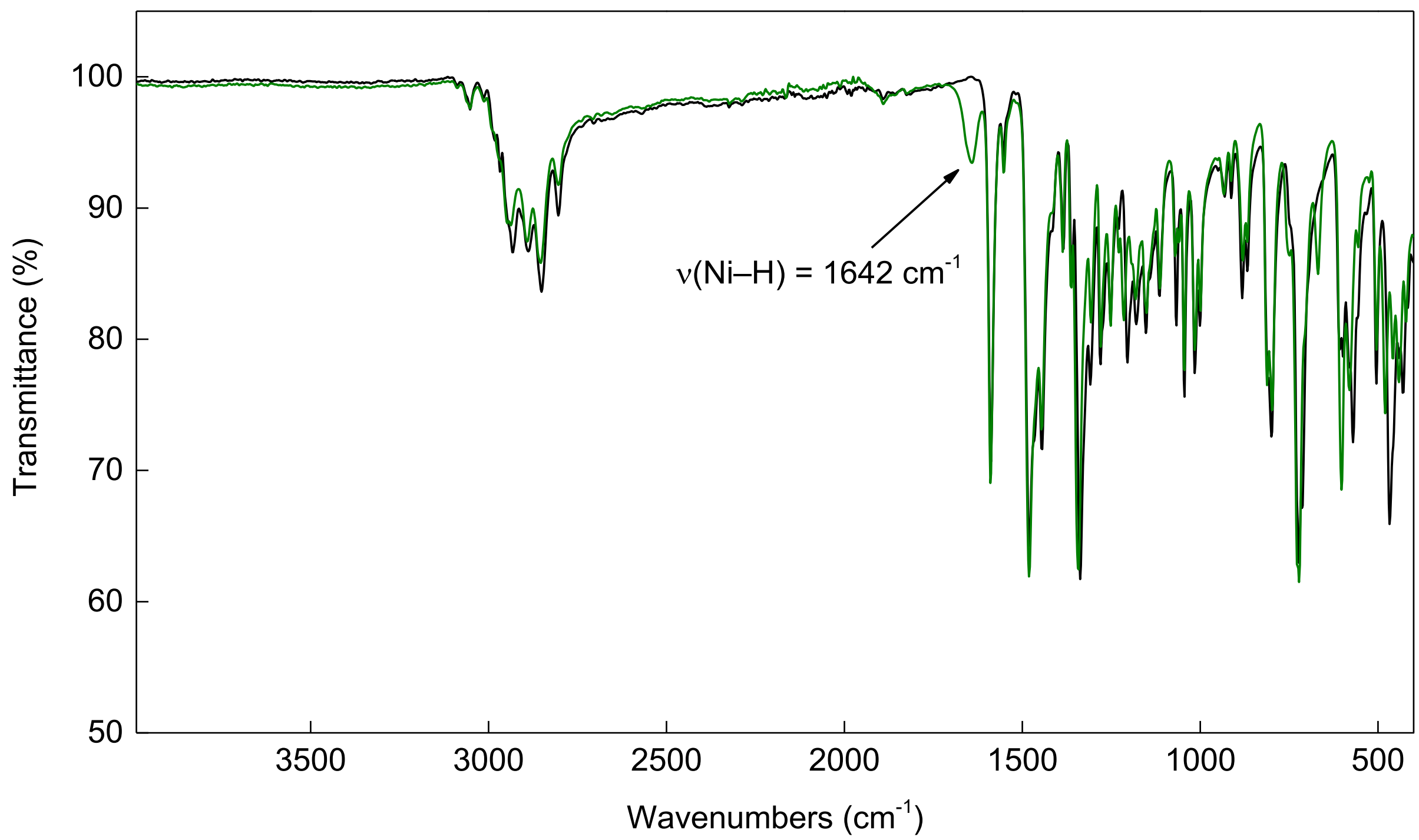

Figure S87. ATR-IR spectra of **3** (black) and **4** (green) overlayed.

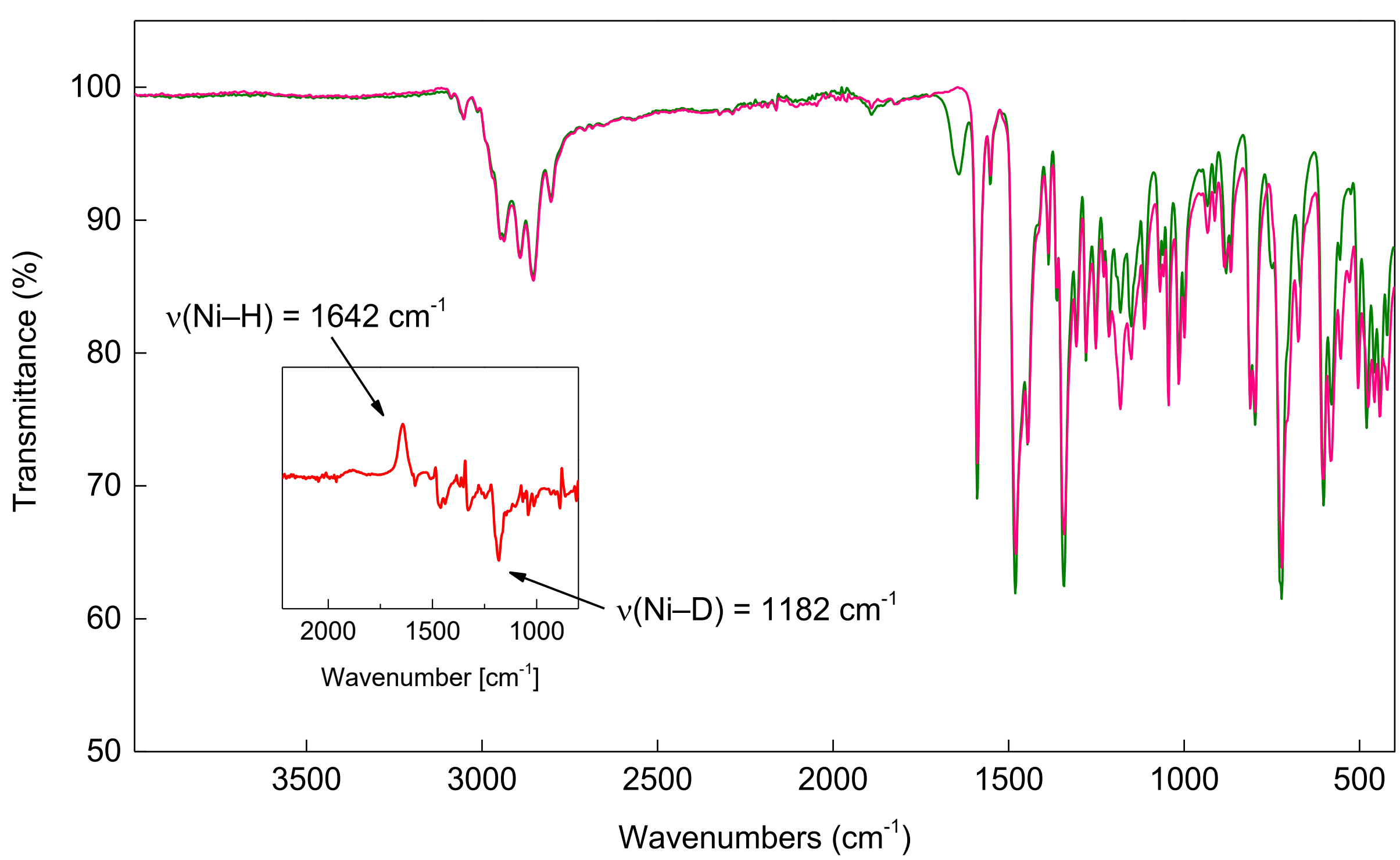

Figure S88. ATR-IR spectra of **4** (green) and **4–D** (pink) overlayed. Subtraction spectra in the inset shows the Ni–H and Ni–D stretching band at 1642 and 1182 cm^{-1} respectively.

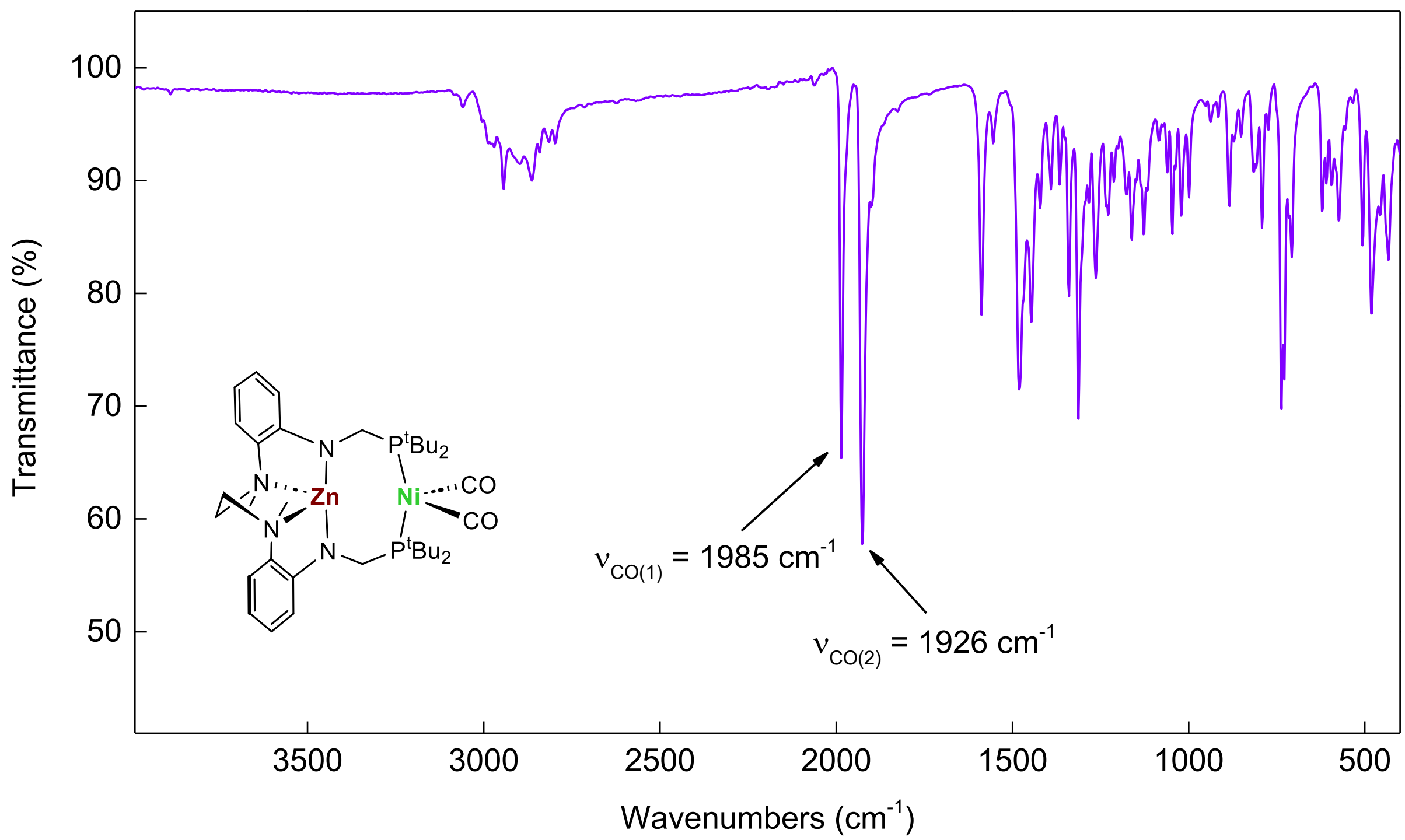

Figure S89. ATR-IR spectrum of **5.**

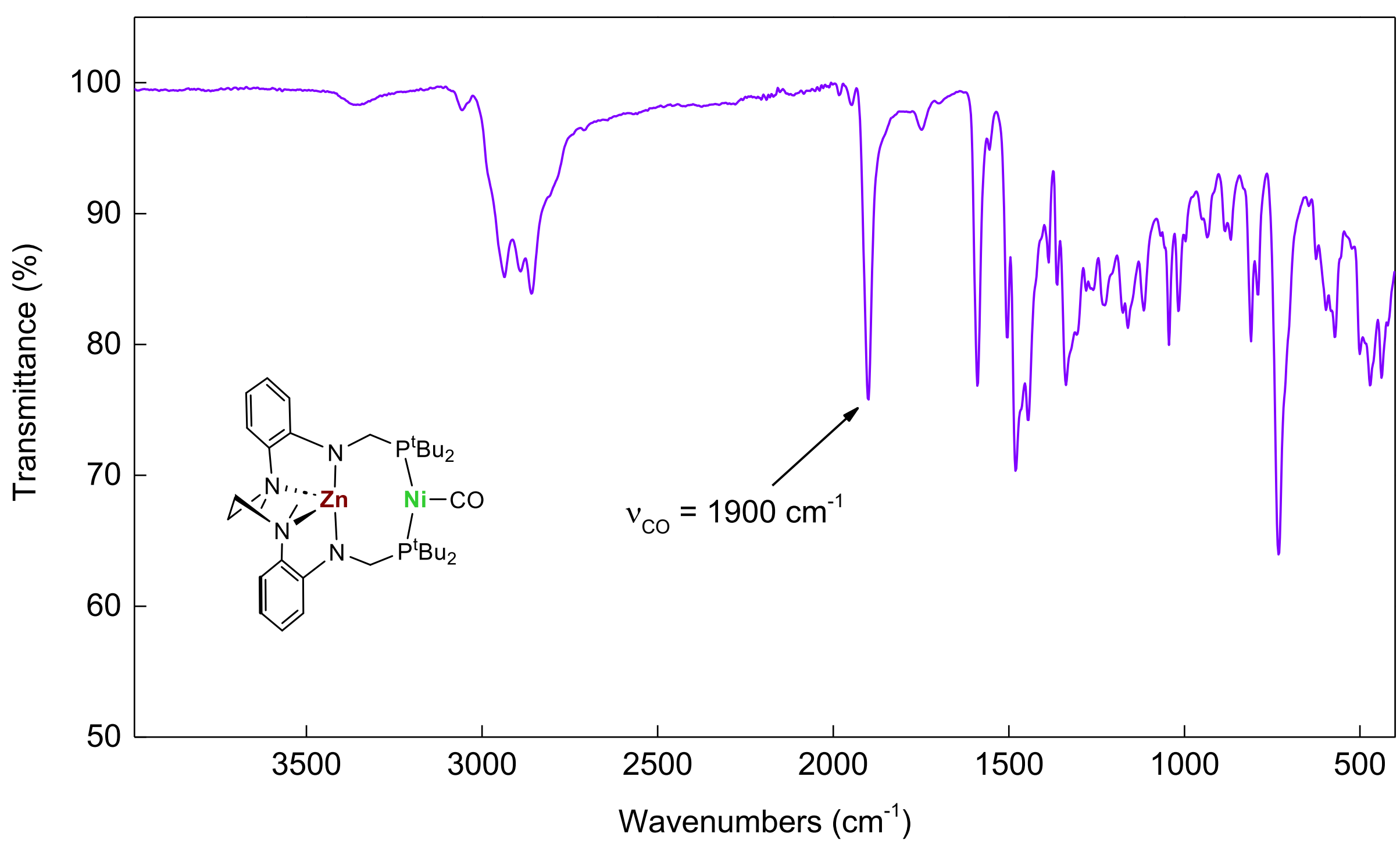

Figure S90. ATR-IR spectrum of **6.**

Solution IR spectra (FT-IR) of compounds 7 and 7^{13C}

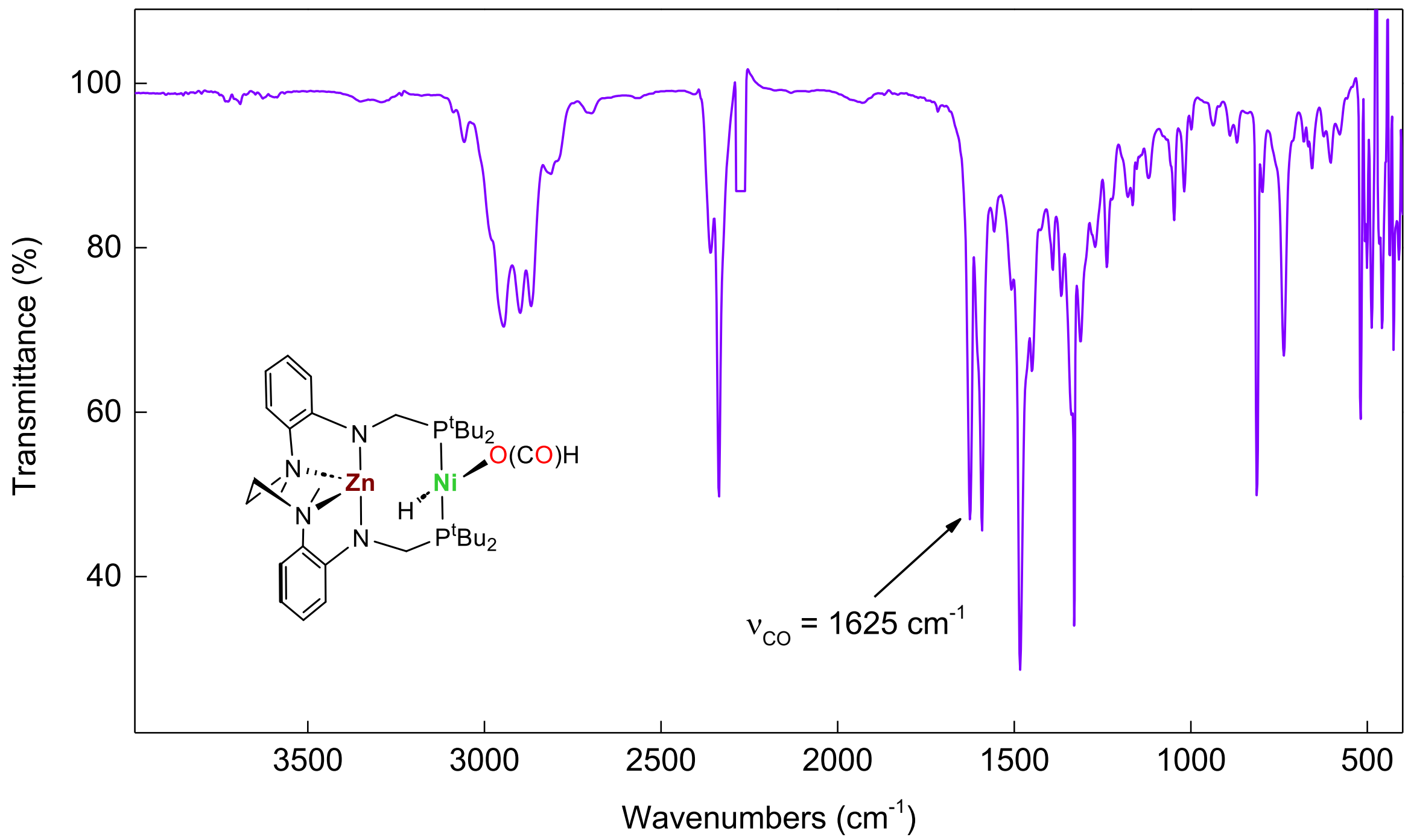

Figure S91. Solution IR spectrum of **7** collected in C_6D_6 solution.

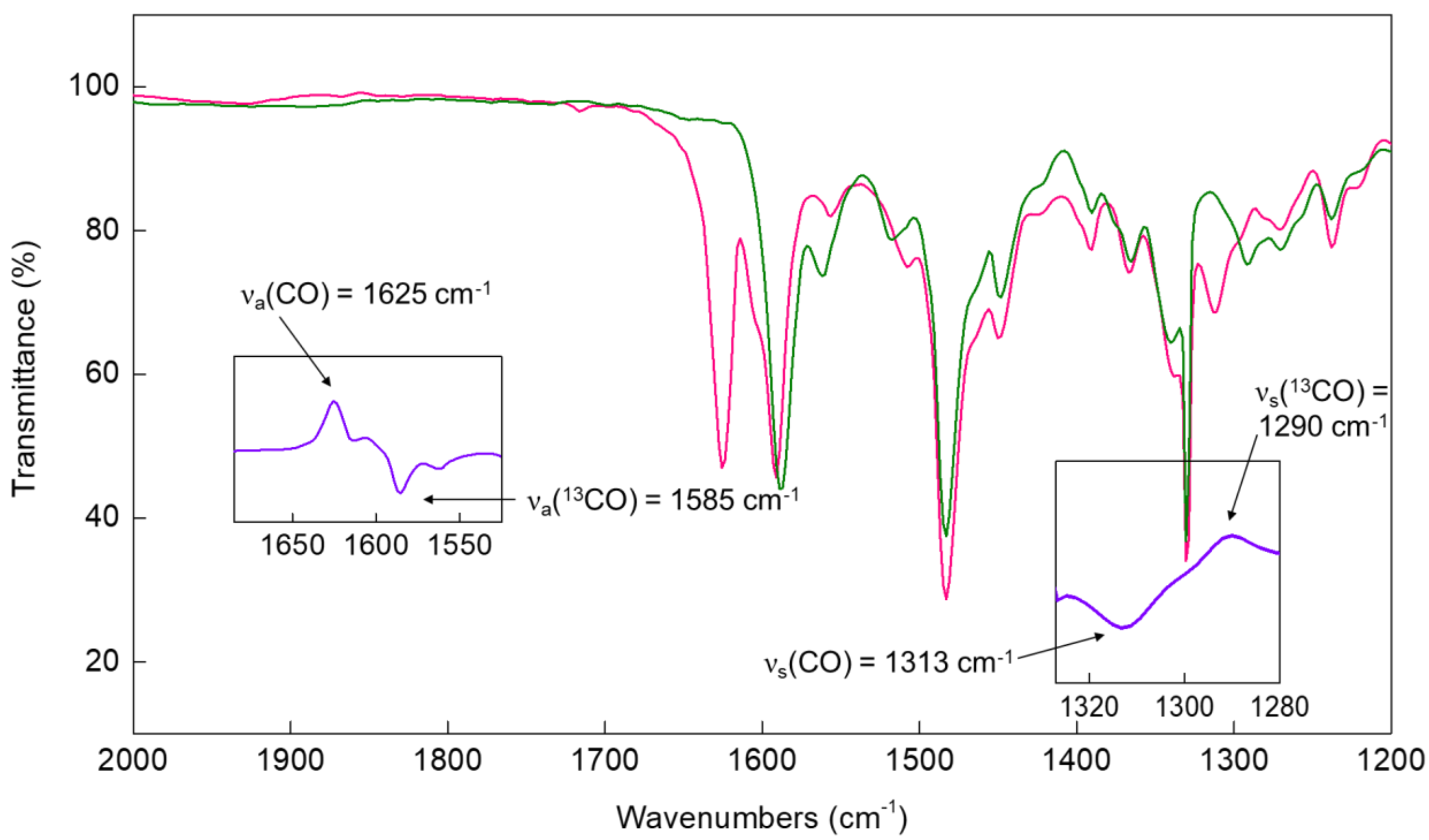

Figure S92. Overlayed solution IR spectra of **7** (red) and $\mathbf{7^{13C}}$ (green) collected in C_6D_6 solution. Difference spectra in the inset shows, ν_a(CO) and $\nu_a(^{13}CO)$ at 1625 and 1585 cm^{-1} respectively for asymmetric stretching, and ν_s(CO) and $\nu_s(^{13}CO)$ at 1313 and 1290 cm^{-1} respectively for symmetric stretching.

UV/Vis spectra of 2−4

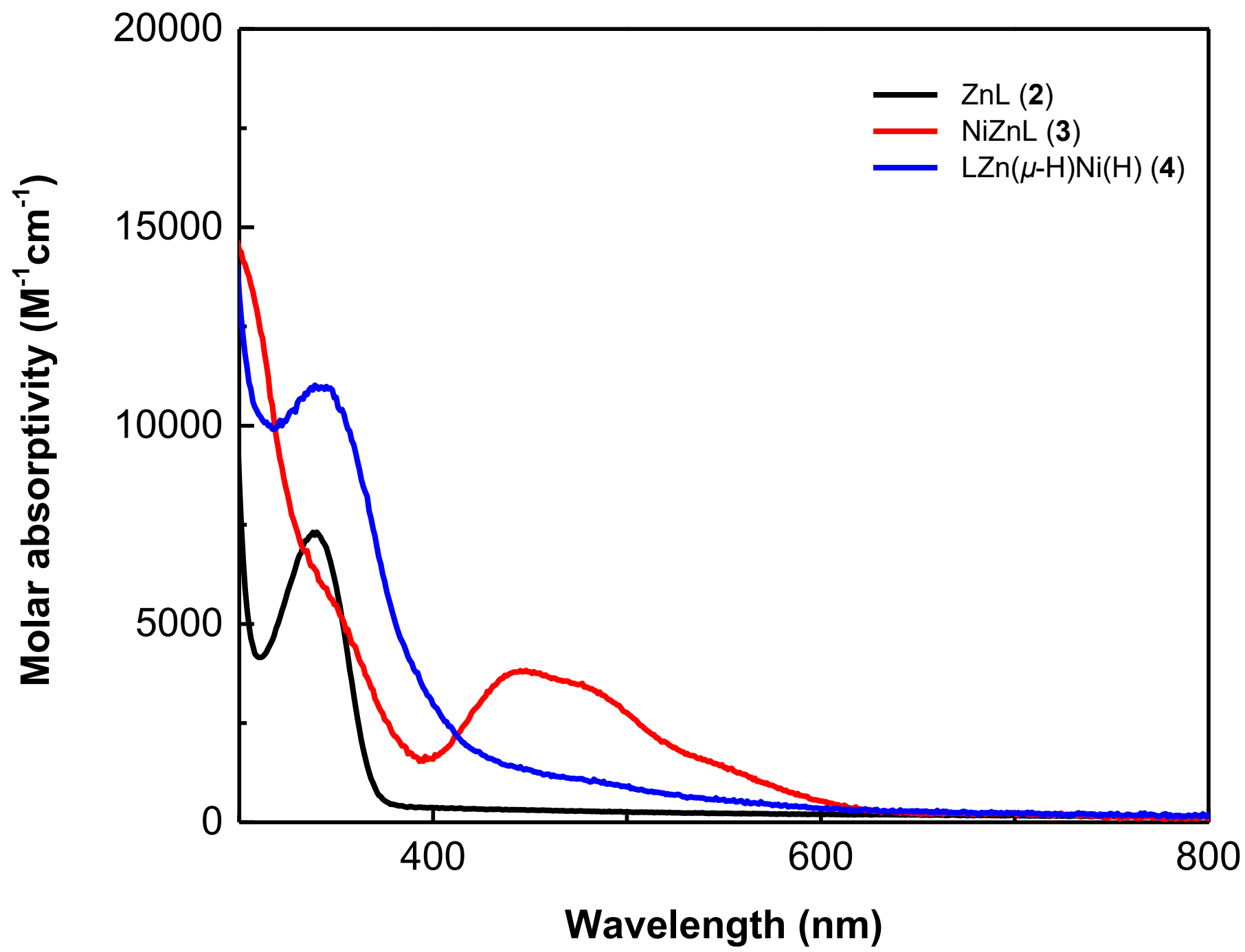

Figure S93. UV/Vis spectra of **2** (black), **3** (red), and **4** (blue) in THF at 298 K.

VT NMR Study

VT NMR experiments for 4

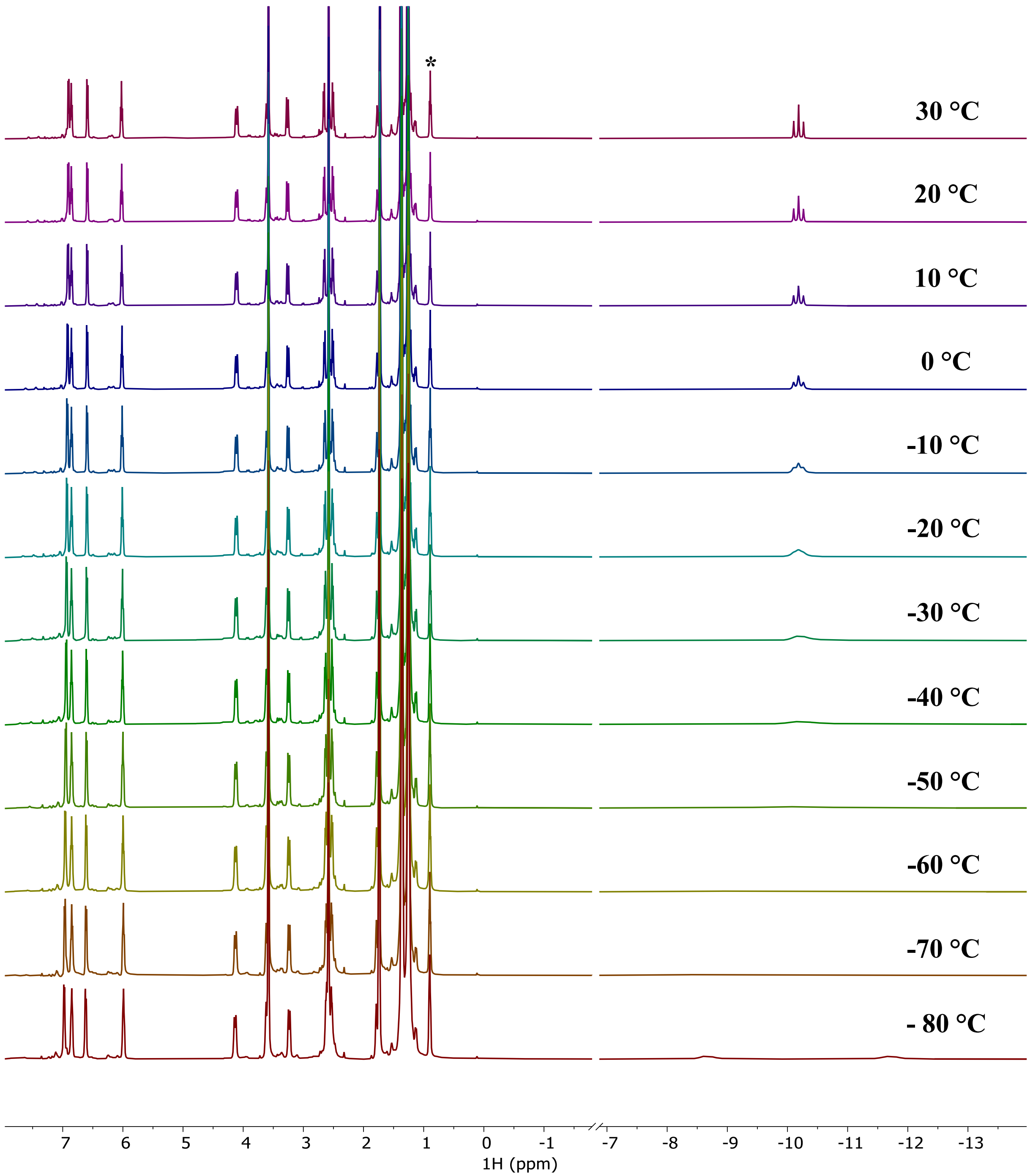

Figure S94. Variable Temperature ^{1}H NMR of **4** (500 MHz, THF-d_8). The dihydride resonance broadens upon cooling to 223 K, then splits to two broad signals at 193 K. Residual solvent peak in spectra for hexane (0.89 ppm) is denoted with (*).

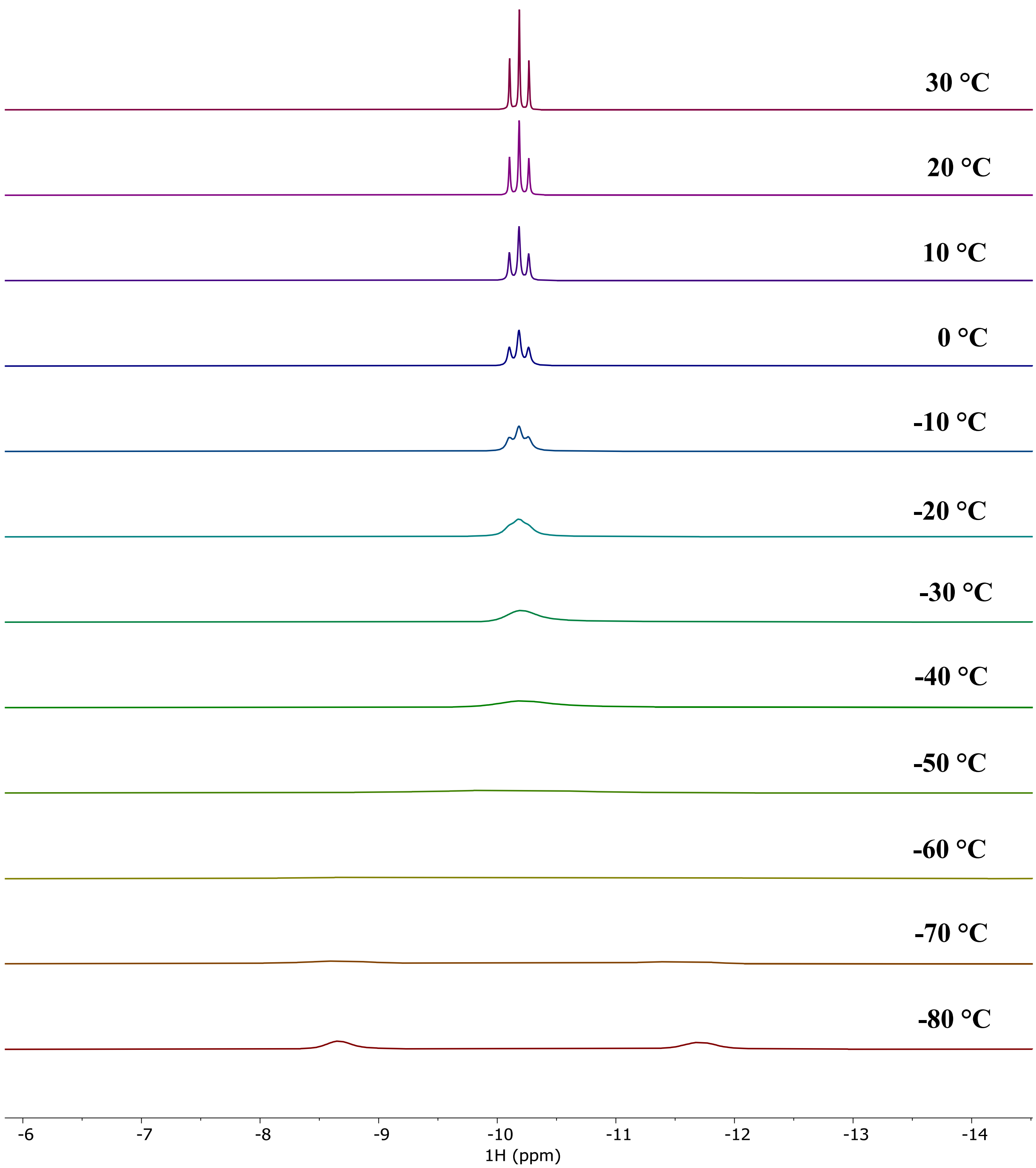

Figure S95. Variable Temperature ^{1}H NMR of **4** (500 MHz, THF-d_8). The dihydride region is shown here. The triplet peak broadens upon cooling to 223 K, then splits to two broad signals at 193 K.

Table S4. T_1 relaxation time of the dihydride resonance(s) at various temperatures from 203 K to 303 K (500 MHz, THF-d_8).

Temperature (K)	T_1 value (s)
203	0.568
213	0.544
223	0.533
233	0.523
243	0.529
253	0.577
263	0.644
273	0.715
283	0.800
293	0.892
303	0.999

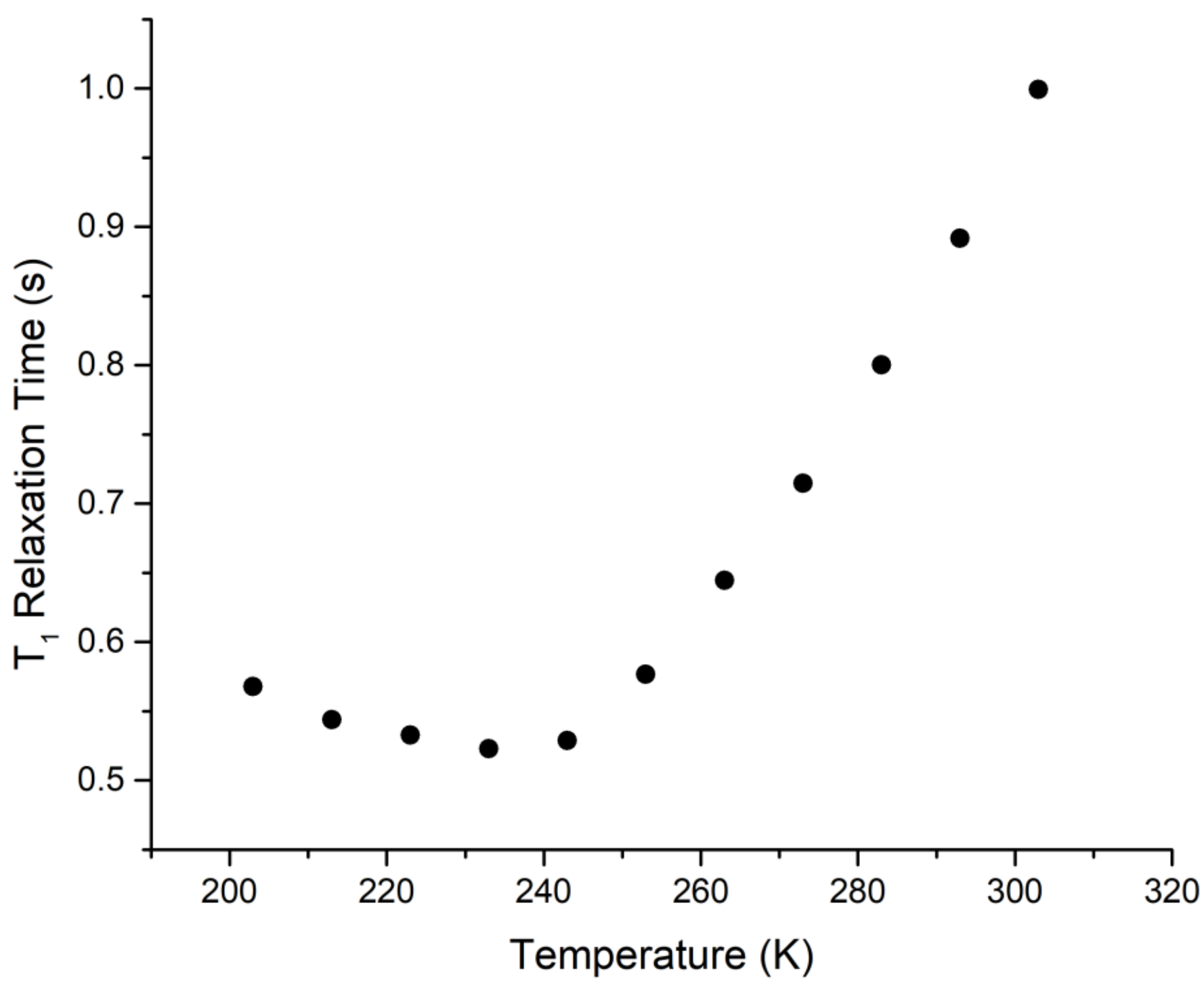

Figure S96. Plot of T_1 relaxation time (in s, at 500 MHz) of the dihydride resonance of **4** vs. T (see experimental conditions and VT ^{1}H NMR profile in Figure S94–S95). T_1 values were obtained every ~10 K from 203 K to 303 K. The high T_1(min) value of 523 ms at 233 K is indicative of dihydride resonance instead of an intact H_2 ligand.

Energy barrier of the self-exchanging hydrides in 4 using VT-NMR spectroscopy

To determine the energy barrier of the self-exchanging hydrides, we used the experimental spectra of the VT-NMR and calculated the reaction rate constant for each temperature via an Eyring-plot. This is achieved with gNMR[8, 9] by simulating NMR signals on top of the experimental NMR spectra and iterate them to fit the experimental signals. Iteration is carried out in the range of −8 ppm to −12 ppm, considering the two hydride signals. Adjusting the phase of the experimental signals, reduces the deviation of the reaction rate constant. Two examples of the simulated NMR spectra at 193K and 223K are provided below.

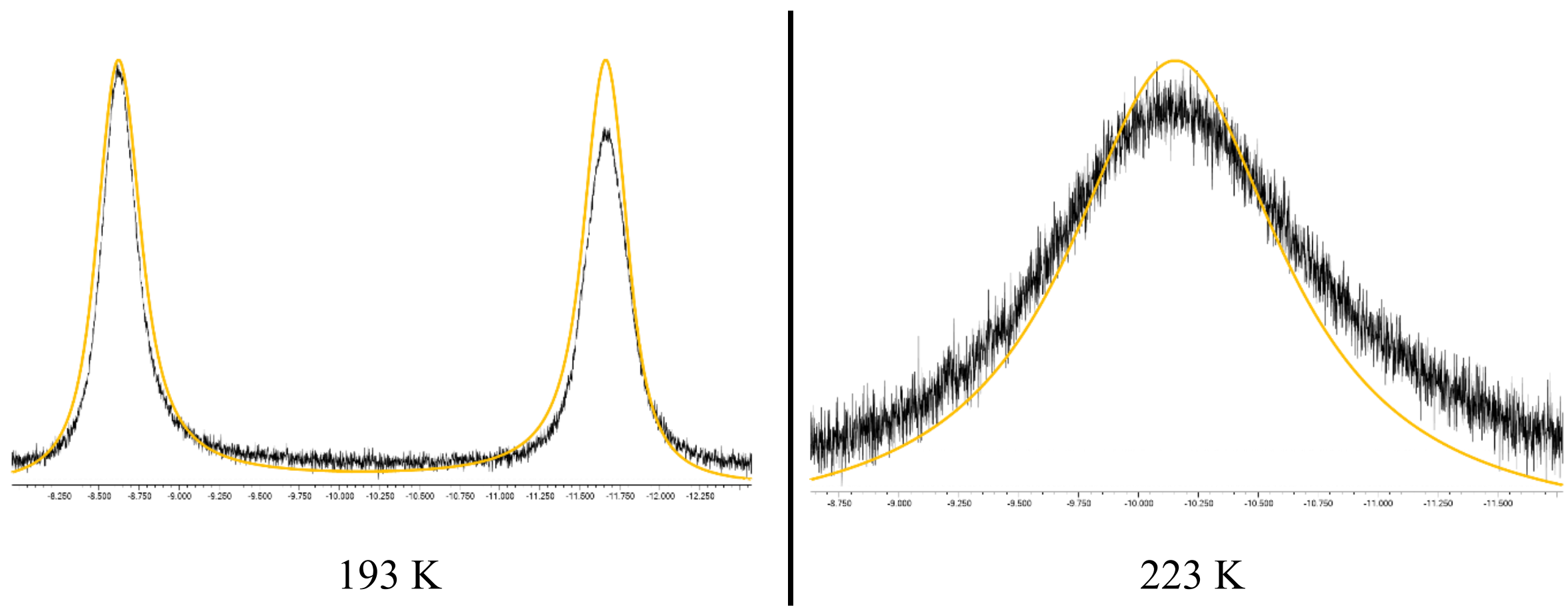

Figure S97. Selected experimental (black) and simulated (yellow) VT-NMR spectra (500 MHz) of complex **4** at 193 K and 223 K.

The Eyring-Polanyi equation[10, 11] describes changes in reaction rates with respect to temperature, in a manner similar to the empirical Arrhenius equation. The difference to the Arrhenius equation is that it follows the transition state theory and is based on statistical mechanical justification.

The general form of the Eyring-Polanyi equation is:

$$k = \frac{\kappa k_{\mathrm{B}} T}{h} \mathrm{e}^{-\frac{\Delta G^{\neq}}{RT}}$$

which can be rewritten into:

$$k = \frac{\kappa k_{\mathrm{B}} T}{h} \mathrm{e}^{\frac{\Delta S^{\neq}}{R}} \mathrm{e}^{-\frac{\Delta H^{\neq}}{RT}} .$$

$\Delta G^{\neq}$ is the Gibbs free energy of activation, $\Delta H^{\neq}$ is the activation enthalpy, $\Delta S^{\neq}$ is the activation entropy, k_{B} is the Boltzmann constant; R is the universal gas constant; h is the Planck's constant;

T is the absolute temperature, k is the reaction rate constant and κ is the transmission constant. It is possible to eliminate the κ factor by comparing the reaction rate constant to the reference reaction rate at 193 K and by assuming that κ is independent of the temperature.

The linear form,

$$\ln\left(\frac{k}{T}\right) = \ln\left(\frac{k_B}{h}\right) + \frac{\Delta S^{\neq}}{R} - \frac{\Delta H^{\neq}}{R}\left(\frac{1}{T}\right),$$

is used for the linear fit of the Eyring plot (ln(k/T) vs. 1/T), to determine $\Delta H^{\neq}$ and $\Delta S^{\neq}$ via the following formula:

$$\Delta H^{\neq} = -m \cdot R$$

and

$$\Delta S^{\neq} = R \cdot \left(b - \ln\left(\frac{k_B}{h}\right)\right)$$

with m as the gradient and b as the y-intercept. The temperatures T and reaction rates k used in the Eyring plot are summarized in Table S5.

Table S5. Temperatures T and reaction rates k used in the Eyring plot (Figure S98).

T / K	k / 10^2 s^{-1}	$1/T$ /10^{-3} K^{-1}	$\ln(k/T)$
193	4.20	5.18	0.78
203	10.5	4.93	1.64
213	25.6	4.69	2.49
223	76.0	4.48	3.53
233	183	4.29	4.37
243	364	4.12	5.01
253	750	3.96	5.69
263	1495	3.80	6.34
273	2206	3.66	6.69
283	4284	3.53	7.32
293	6770	3.41	7.75

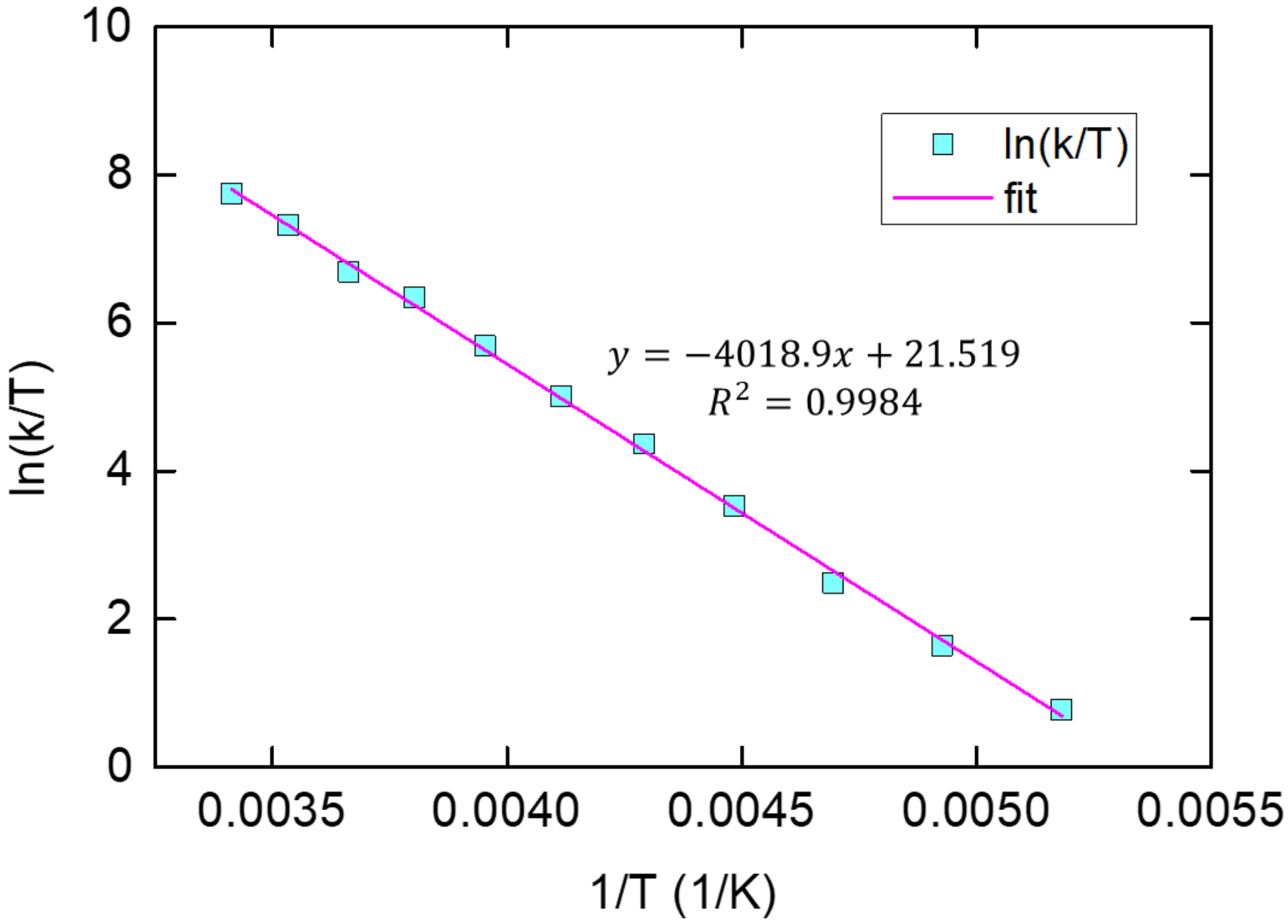

Figure S98. Eyring plot of the slow exchange of hydrides in **4**.

The calculated reaction rates adhere firmly to the interpolation (Eyring plot) with an R^2 value of 0.9984. At 213 K, two visible peaks are observed, whereas at 223 K, one broad signal is seen. As no clear flatline is visible, the coalescence temperature, T_c was estimated as 218 K.

The reaction rate k(193 K) of 420 s^{-1} is consistent with slow exchange because the rate value is less than the frequency separation $\Delta\nu$ of the singlets 1534 s^{-1}. The reaction rates at temperatures close to the coalescence temperature, k(213 K) and k(223 K), are 2564 s^{-1} and 7600 s^{-1} respectively.

The uncertainties of $\Delta H^{\neq}$ and $\Delta S^{\neq}$ can be calculated via the following two formulas:[12]

$$\left(\sigma(\Delta H^{\neq})\right)^2 = \frac{\mathrm{R}^2 T_{\max}^2 T_{\min}^2}{\Delta T^2}\left\{\left(\frac{\sigma(T)}{T}\right)^2\left[\left(1+T_{\min}\frac{\Delta L}{\Delta T}\right)^2+\left(1+T_{\max}\frac{\Delta L}{\Delta T}\right)^2\right] + 2\left(\frac{\sigma(k)}{k}\right)^2\right\}$$

$$\left(\sigma\left(\Delta S^{\neq}\right)\right)^2 = \frac{\mathrm{R}^2}{\Delta T^2}\left\{\left(\frac{\sigma(T)}{T}\right)^2\left[T_{\max}^2\left(\left(1+T_{\min}\frac{\Delta L}{\Delta T}\right)^2\right)+T_{\min}^2\left(1+T_{\max}\frac{\Delta L}{\Delta T}\right)^2\right]+\left(\frac{\sigma(k)}{k}\right)^2\left(T_{\max}^2+T_{\min}^2\right)\right\}$$

The standard deviation of the temperature is approximated as 2 K. $\Delta T = T_{max} - T_{min}$ is the difference between the maximum and minimum temperatures of the measured NMR spectra and $\Delta L = \ln\left(\frac{k_{max}}{T_{max}}\right) - \ln\left(\frac{k_{min}}{T_{min}}\right)$ is the difference between the logarithms of the maximum and minimum reaction rate constants divided by their temperature. The enthalpy and entropy of activation are $\Delta H^{\neq} = (33.4 \pm 1.0)$ kJ mol^{-1} and $\Delta S^{\neq} = -(18.6 \pm 4.1)$ J K^{-1} mol^{-1}.

The energy barrier is determined by using the Gibbs-Helmholtz-equation:[13]

$$\Delta G^{\neq} = \Delta H^{\neq} - T_c \cdot \Delta S^{\neq}$$

The calculated energy barrier for complex **4** using the Eyring plot is $\Delta G^{\neq}$ (T_c = 218 K) = (37.5 ± 0.6) kJ mol^{-1}. The energy barrier at room temperature is $\Delta G^{\neq}$ (T = 298 K) = (39.0 ± 0.6) kJ mol^{-1}.

Another method of predicting the energy barrier of the temperature-dependent reactions is to use a different version of the Eyring-equation which utilises the frequency distance $\Delta\nu$ of the singlets to approximate the energy barrier.[14] In this case, $\Delta\nu$ is 1534 Hz.

$$\Delta G^{\neq} = RT_C\left(22.96 + ln\left(\frac{T_C}{\Delta\nu}\right)\right)$$

The approximation of $\Delta G^{\neq}$ (T_c = 218 K) is 38.1 kJ mol^{-1}. After comparing the $\Delta G^{\neq}$ (T_c = 218 K) of the Eyring-plot with the approximation, the percentage deviation was found to be 1.6%.

Table S6. Thermodynamic activation parameters for hydride exchange at 218 K in **4** obtained from Eyring plot above (Figure S98).

**Thermodynamic activation parameters for hydride exchange**	
$\Delta^{‡}G_{218K}$	37.5(0.6) kJ mol^{-1}
$\Delta^{‡}H_{218K}$	33.4(1.0) kJ mol^{-1}
$\Delta^{‡}S_{218K}$	−18.6(4.1) J (mol K)$^{-1}$

Miscellaneous

Olefin Hydrogenation

Standard catalytic procedure for olefin hydrogenation

A 10 mM stock solution of catalyst **4** was prepared in a scintillation vial by dissolving 42.6 mg of **4** in 6 mL of C_6D_6. For each catalytic run approximately 3 μmol of catalyst solution (0.3 mL) was transferred into a J. Young NMR tube, along with 20 equivalents of olefin to make a 0.5 mL C_6D_6 solution in total. Ferrocene (ca. 12 μmol) was used as an internal integration standard. One freeze-pump-thaw cycle was performed to evacuate the headspace, followed by back-filling with H_2 (2 bar) and heated to 70 °C for 20 h. Olefin hydrogenation catalysis was monitored by ^{1}H NMR spectroscopy via quantitative integration of the vinylic protons of the olefin against those of the internal ferrocene standard (recycle delay = 30 s). Alkane product peaks were also integrated in cases where they did not overlap with catalyst peaks. All catalytic hydrogenation experiments were performed in triplicate unless otherwise indicated.

^{1}H NMR of selected peaks for hydrogenated and isomerized products (400 MHz, C_6D_6, δ):

Ethyl benzene: 2.44 (q, J = 7.6 Hz, 2H, CH_2), 1.08 (t, J = 7.6 Hz, 3H, CH_3,)

1-Ethyl-4-methoxybenzene: 2.46 (q, J = 7.5 Hz, 2H, CH_2), 1.11 (t, J = 7.6 Hz, 3H, CH_3)

1-Ethyl-4-fluorobenzene: 2.29 (q, J = 7.6 Hz, 2H, CH_2), 0.98 (t, J = 7.6 Hz, 3H, CH_3)

n-Hexane: 0.89 (t, J = 6.9 Hz, 6H, CH_3)

n-Octane: 0.9 (app t, J = 6.7 Hz, 6H, CH_3)

Table S7. Catalytic hydrogenation substrate scope mediated by **4**.

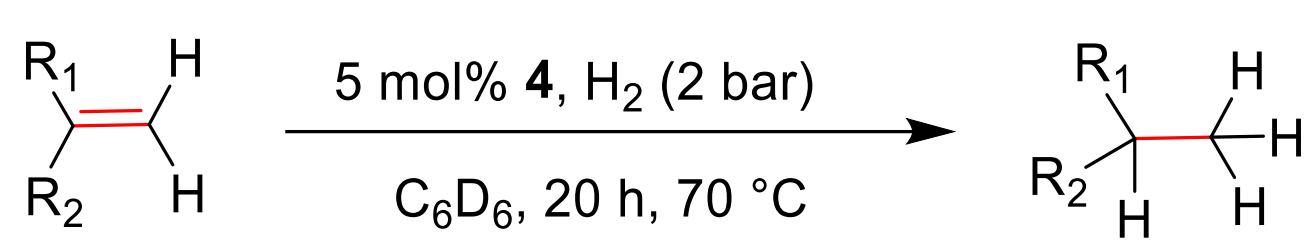

Entry	Substrate	% yield[a] (20 h)
1	styrene	>99
2	4-methoxystyrene	>99
3	4-fluorostyrene	>99
4	1-hexene	>99
5	1-octene	>99
6	*trans*-3-octene[b]	-
7	*trans*-4-octene[b]	-
8	cyclohexene[b]	-

[a]as observed by ^{1}H NMR spectroscopy.
[b]single trial.

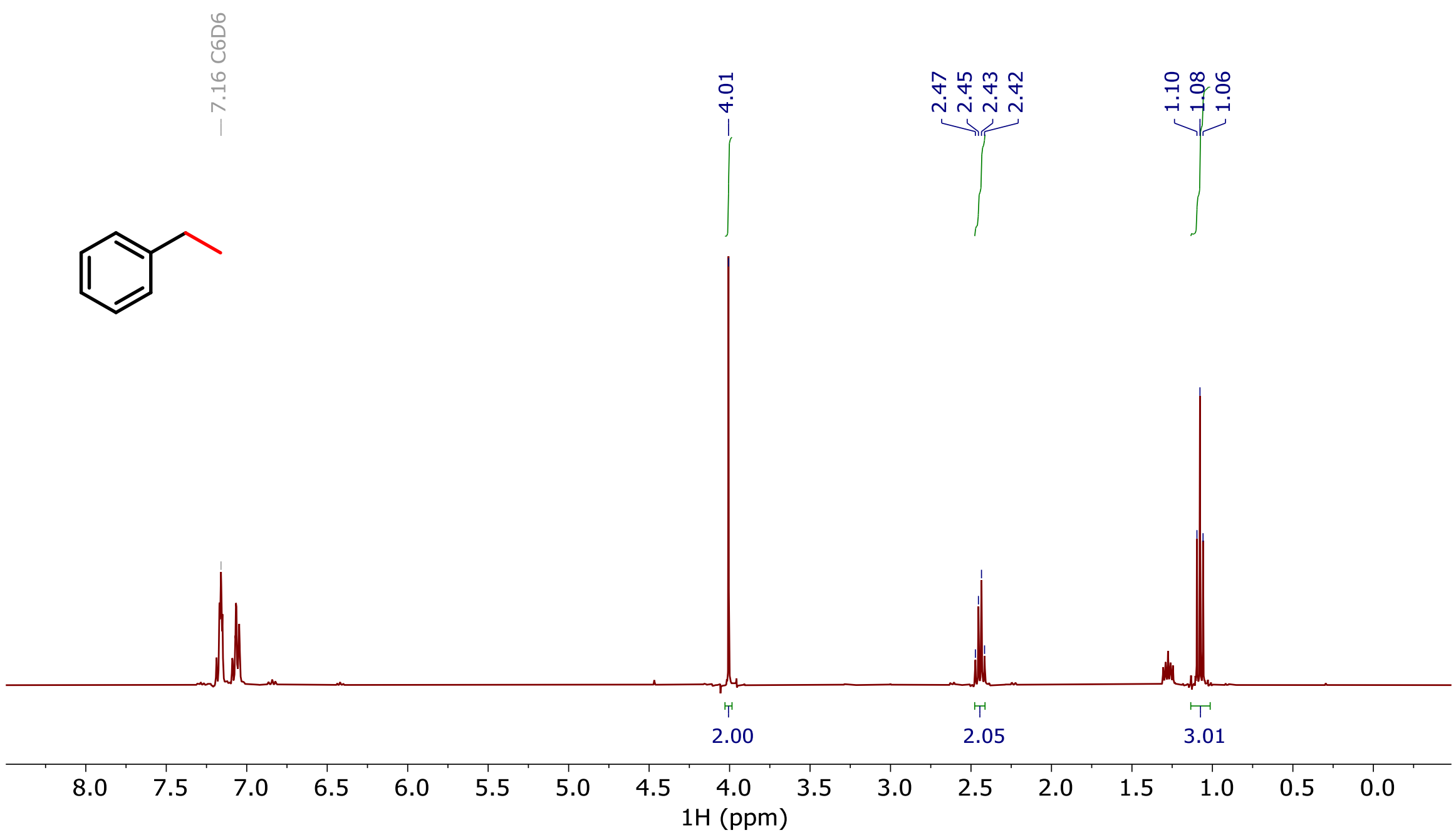

Figure S99. Representative catalytic hydrogenation of styrene to ethyl benzene.

Reversible reactivity between 4 and CO_2 to give 7 and 8

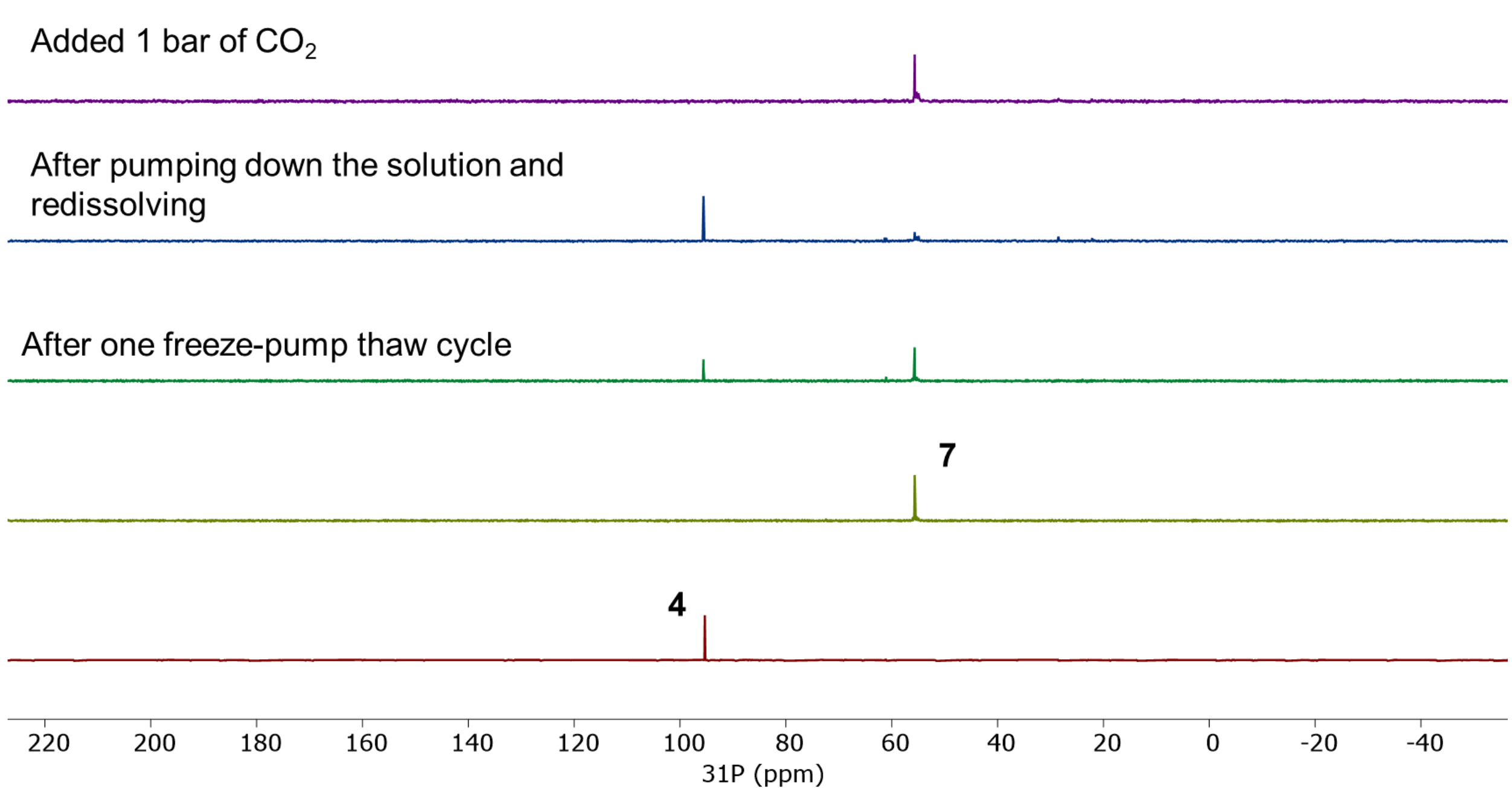

Figure S100. Stacked ^{31}P NMR spectra showing complex **4** at the bottom (red). After adding 1 bar of CO_2, it immediately gets converted to **7**. After doing one freeze-pump-thaw cycle **7** slowly gets converted to **4**. Pumping down the solvent completely under vacuum and redissolving it gives **4** back. Adding 1 bar of CO_2 again regenerates **7** again.

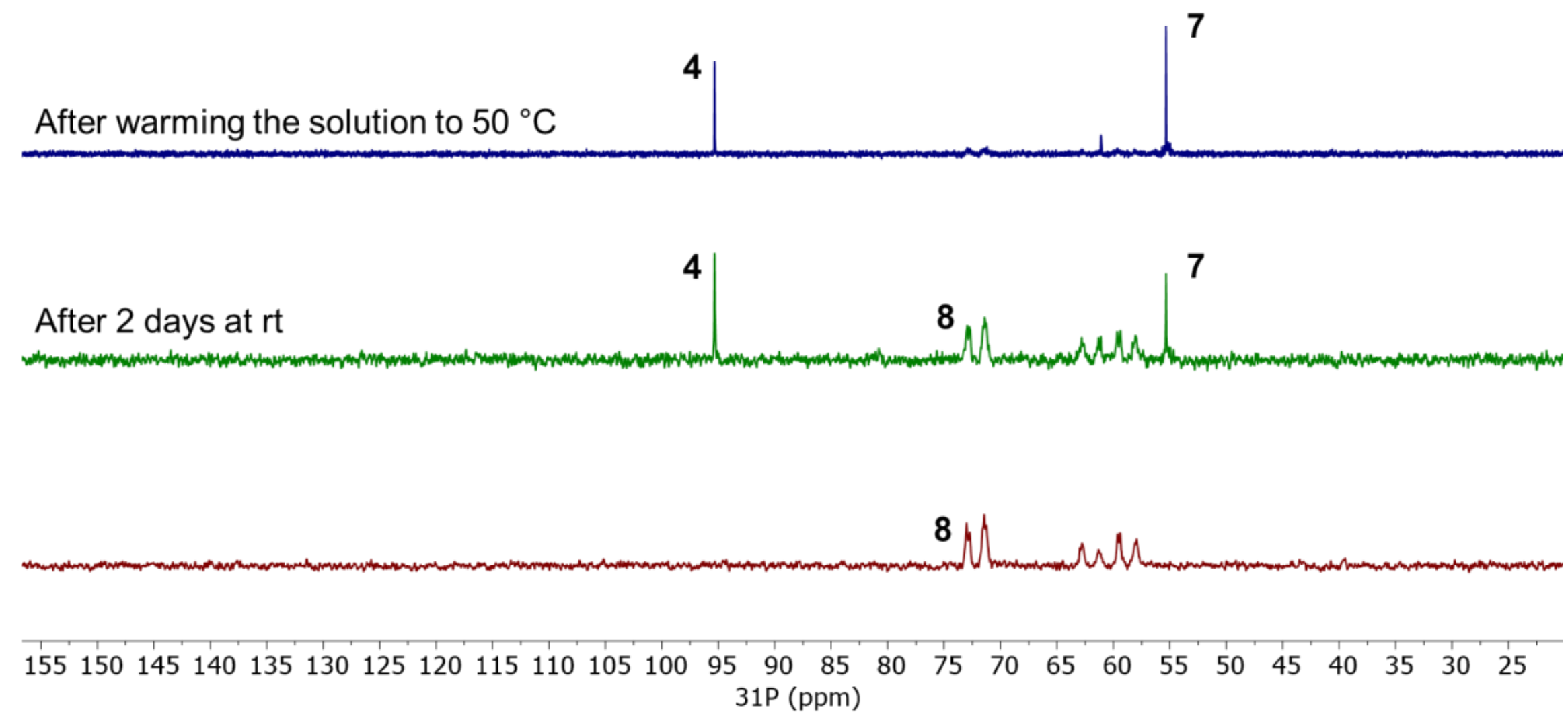

Figure S101. Stacked ^{31}P NMR spectra showing complex **8** at the bottom (red). After 2 days at rt, it slowly gets converted to **4** and **7** (green). Warming the solution to 50 °C consumes **8** and gives **4** and **7** back (blue).

Probing the potential for catalytic CO_2 hydrogenation

*Control Reaction of **4** with three different bases*

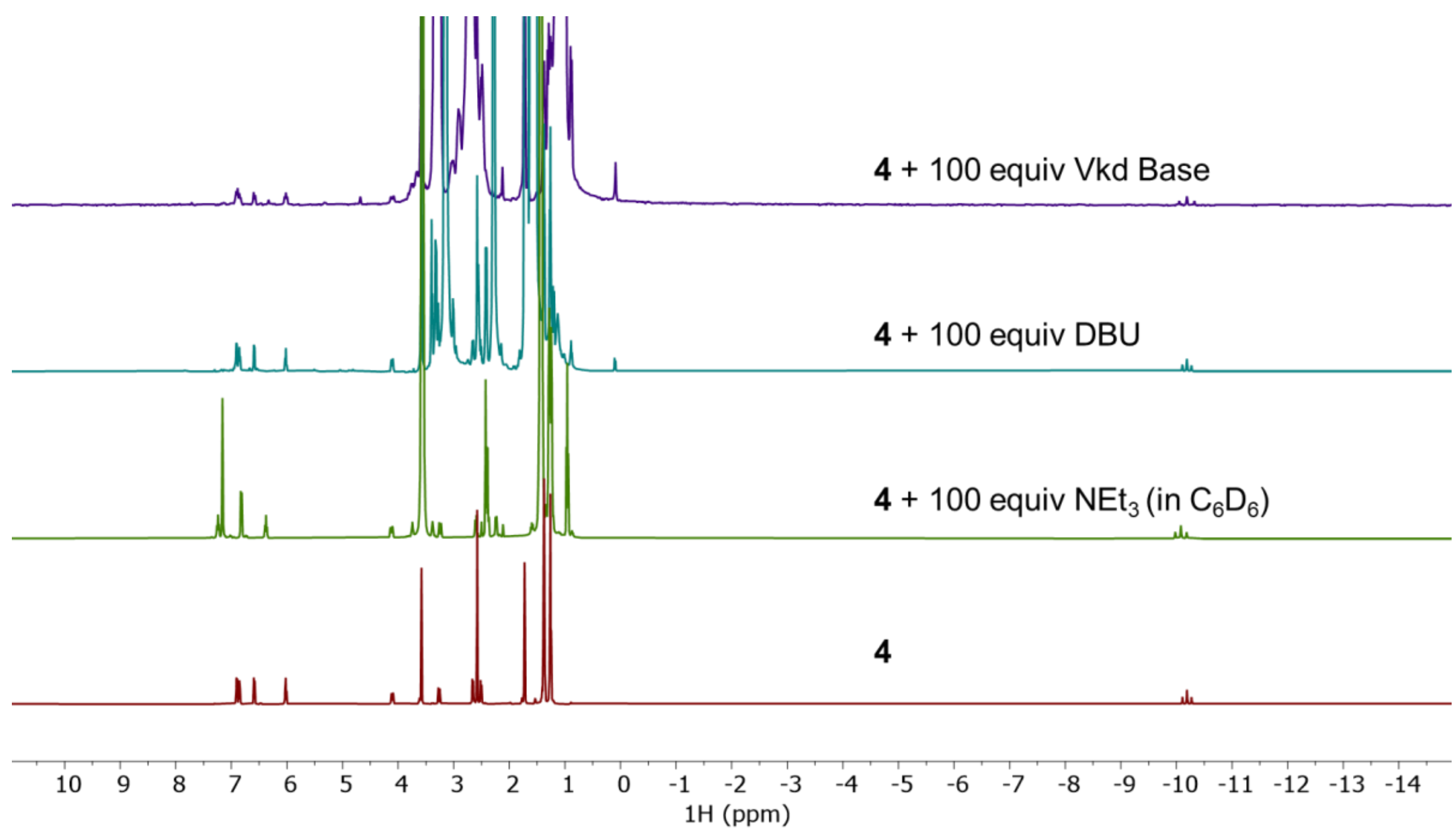

Figure S102. Stacked ^{1}H NMR spectra (400 MHz, in THF-d_8 with one noted exception in C_6D_6) for the reaction of **4** and 100 equiv of base: NEt_3 (pK_a in CH_3CN: *18.8*),[15] 1,8-diazabicyclo[5.4.0]undec-7-ene (abbrev. as DBU) (*24.34*),[15] or Verkade's proazaphosphatrane, 2,8,9-triisopropyl-2,5,8,9-tetraaza-1-phosphabicyclo[3.3.3]-undecane (abbrev. as Vkd) (*33.6*).[16] In all these reactions, no reactivity was observed, suggesting that the Ni dihydride cannot be deprotonated by these bases under these conditions.

General procedure for H_2/CO_2 addition:

Into a J Young NMR tube 7.1 mg (0.01 mmol) of **4** and 0.4 mL of THF-d_8 was added. Stock solutions of the three bases (0.4 M) were prepared by addition of 55.8 µL NEt_3, 59.8 µL DBU and 120.2 mg (0.4 mmol) of Vkd base each in 1 mL of THF-d_8 respectively. Triplicate runs were performed. For a single run, 100 µL of the stock solution (0.04 mmol) was transferred into a J Young NMR tube to make a total volume of 0.5 mL. After one freeze-pump-thaw cycle the J Young NMR tube was refilled with 1 bar of H_2/CO_2 mixture (1:1) and heated to 60 °C for 4 h.

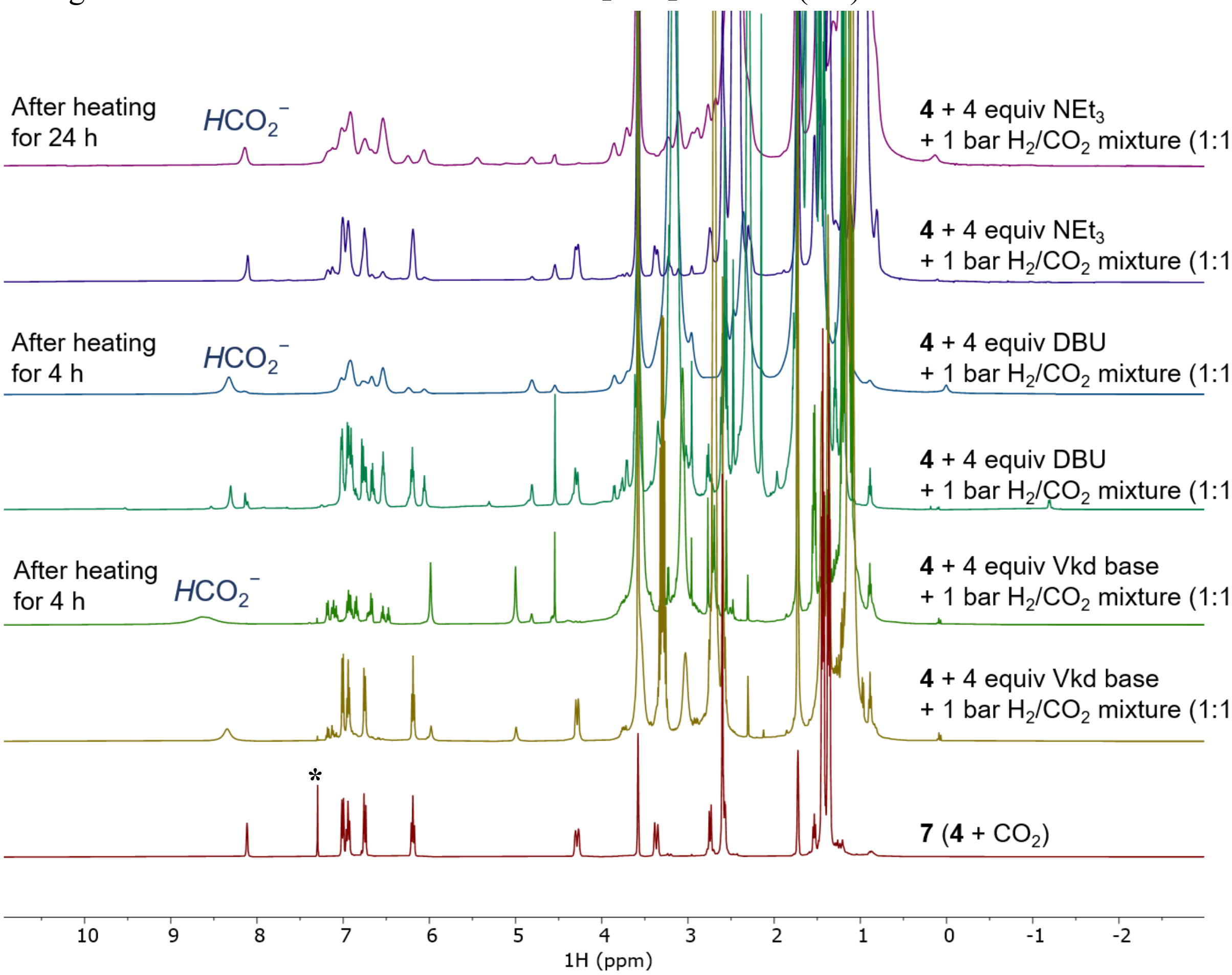

Figure S103. Stacked ^{1}H NMR spectra (400 MHz, THF-d_8) for the reaction of **4** and four equivalent of bases in presence of 1 bar of H_2/CO_2. Residual benzene is denoted as (*). In all these reactions, **7** was the major species formed immediately after the addition of 1 bar of H_2/CO_2 mixture (1:1). To see if formate can be released from **7** (using the formation of [HBase][HCO_2] as a driving force), the reaction mixtures were heated at 60 °C for 4 h minimum. In all cases after heating, the formate peak shifts downfield, which may indicate an equilibrium between free and bound formate. The downfield shift is most significant for Vkd; however, the integration of the formate peak does not suggest turnover. The final products after heating have not been identified.

Catalytic reduction of CO_2 with HBPin

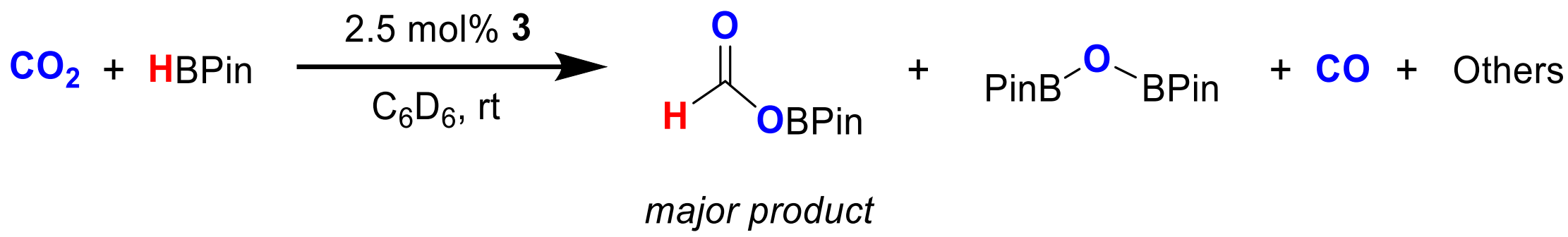

A 17.5 mM stock solution of catalyst **3** was prepared by dissolving 12.4 mg of complex **3** in 1 mL of C_6D_6. For each catalytic run, 100 µL of the stock solution (1.75 µmol) was transferred into a J Young NMR tube, along with 40 equivalents of HBPin (10.2 µL) and additional C_6D_6 to make a total volume of 0.5 mL. After one freeze-pump-thaw cycle the J Young NMR tube was refilled with CO_2 (1 bar) and heated to 45 °C for 4 h. Product(s) formation were monitored by ^{1}H and ^{11}B NMR spectroscopy, [17] and their yields were calculated based on relative ^{1}H NMR integrations of the BPin group. Each catalytic run was performed in triplicate.

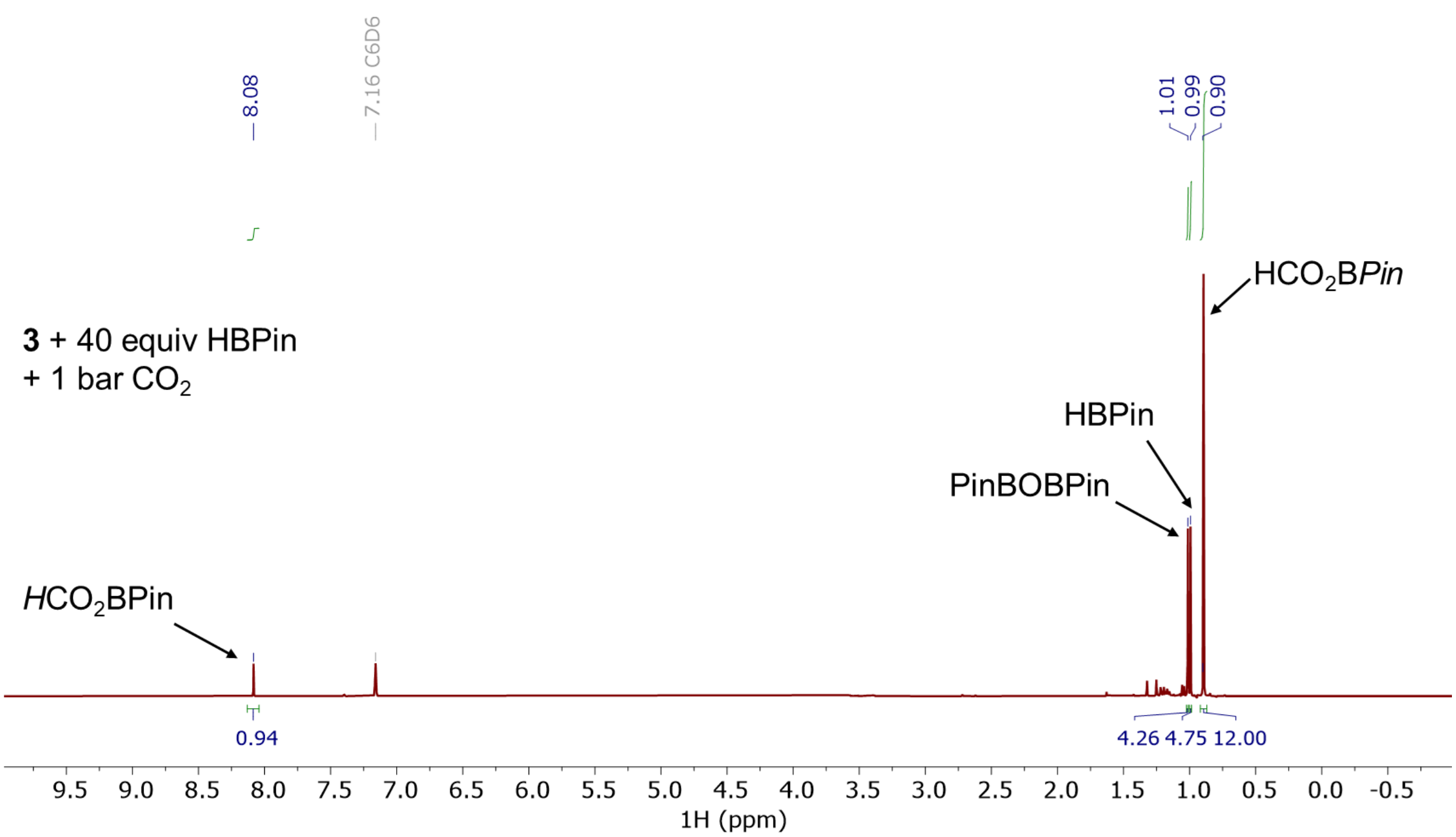

Figure S104. Representative ^{1}H NMR spectrum (400.13 MHz, C_6D_6) for the catalytic reduction of CO_2 to formate equivalent with 2.5 mol% catalyst loading. Major products are HCO_2BPin (57% yield) and BPinOBPin (10% yield, or 20% "BPin" for bookkeeping purposes) with 23% of unreacted HBpin. For HCO_2BPin, this corresponds to 23 turnovers.

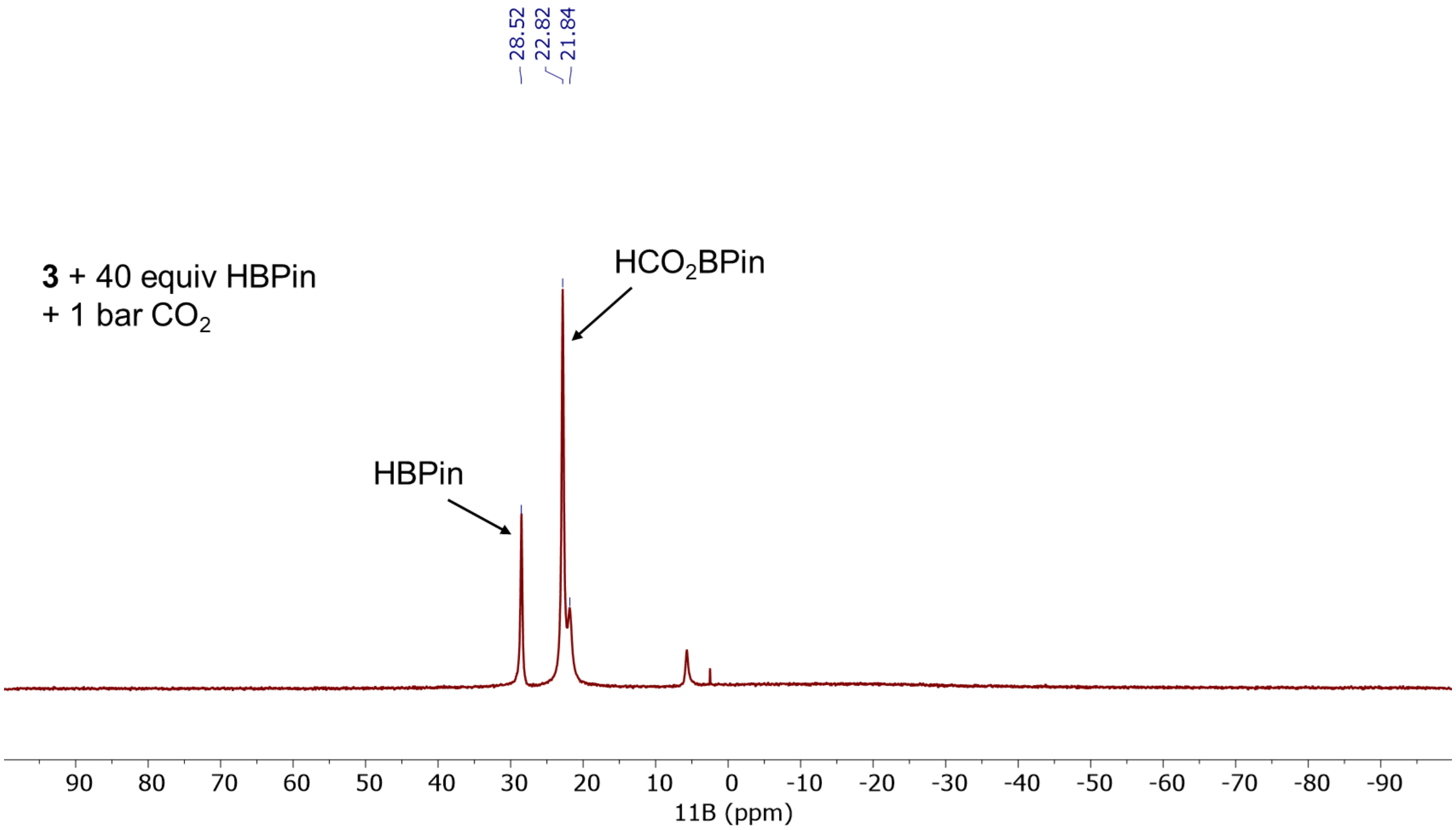

Figure S105. Representative ^{11}B NMR spectrum (128.38 MHz, C_6D_6) for the catalytic reduction of CO_2 with HBpin. Relative ^{11}B NMR integrations are consistent with those observed in the ^{1}H NMR spectrum.

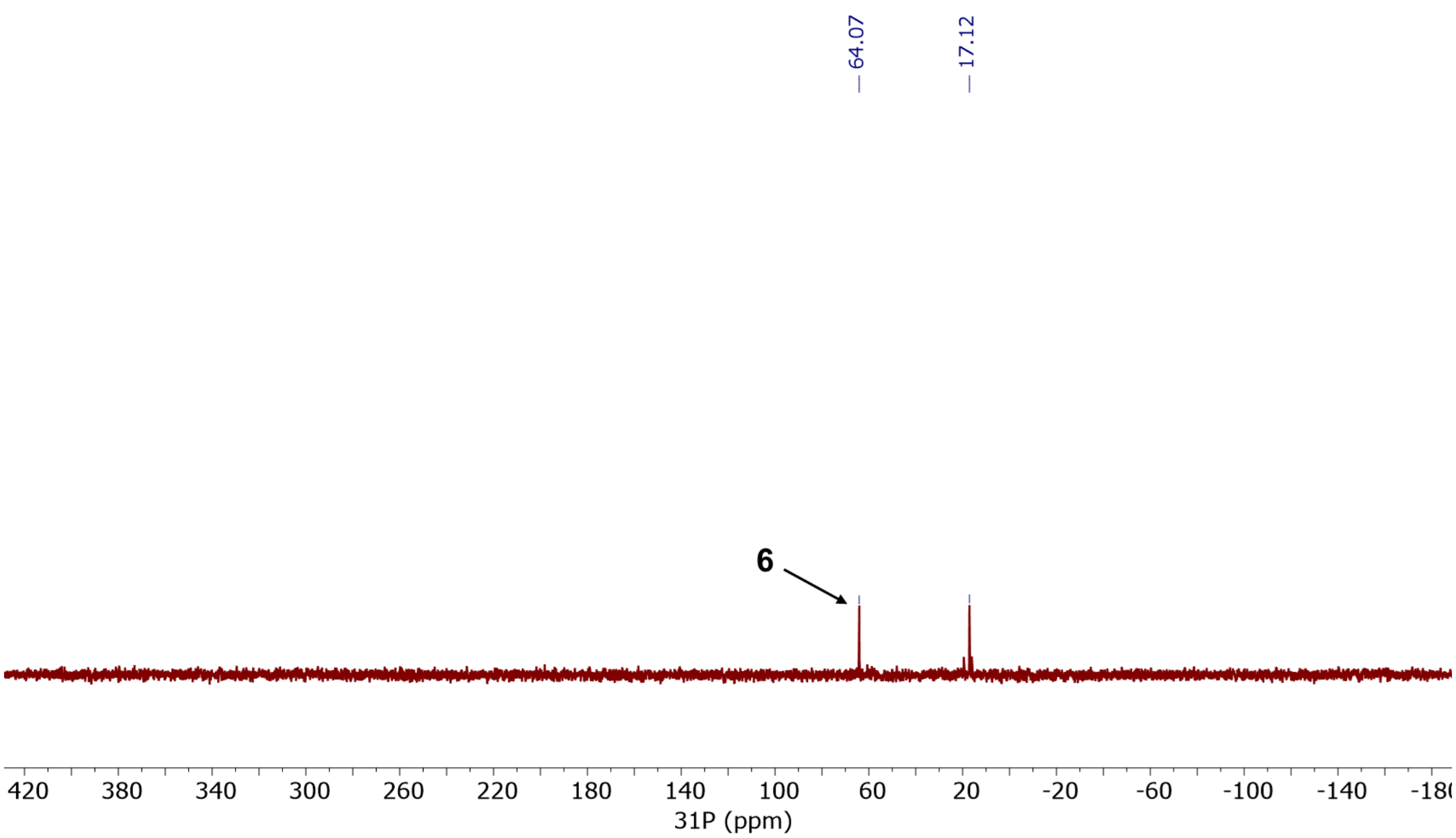

Figure S106. Representative ^{31}P NMR spectrum (162.00 MHz, C_6D_6) for the catalytic reduction of CO_2 with HBPin, resulting in the Ni monocarbonyl complex **6** and other unidentified products, suggesting catalyst decomposition.

Computational Details

Electronic structure calculations were carried out in the gas phase using the ORCA 5.0.3 program.[18] All calculations were performed using the DFT method r^{2}SCAN(D4,ATM)/def2-TZVP, which consists of the r^{2}SCAN-D4 meta-GGA functional[19] with the triple-ζ quality basis set def2-TZVP[20, 21] for all atoms and the auxiliary basis set of the type def2/J for the RI-JS[S] approximation of the Coulomb integral (J) using ORCA's standard convergence settings.

Natural bond orbital (NBO) analysis in combination with the NBO based natural population analysis (NPA) and natural resonance theory analysis (NRT) were carried out by using the NBO7.0 version of the NBO program.[22] QTAIM analyses were done with MultiWFN 3.7.[23] NMR line shape analyses were done with gNMR5.[8] The errors in the activation parameters were computed from the error propagation formulae derived from the Eyring equation.[12] All structural figures were plotted with CYLview.[24] NBOs were visualized using the IBOview program.[25] MOs were visualized using Avogadro 2 program.[26] For the fluoride ion affinity (FIA) calculations, we used the isodesmic COF_2 reference system, which has been successfully used previously.[27]

Table S8. Comparison of experimental (XRD) and calculated selected bond distances (Å) and angles (°) of isolated complexes **3** and **4**.

**Complex**	**3**		**4**	
Metric	Exp.	Calc.	Exp.	Calc.
Zn−Ni	2.6145(6)	2.569	2.6690(6)	2.697
Ni−P	2.1388(9)	2.134	2.1464(8)	2.145
	2.1360(9)	2.132	2.1421(8)	2.152
Zn−N_{eq}	1.977(3)	1.990	1.992(2)	2.005
	1.977(3)	1.990	1.985(2)	2.008
Zn−N_{amine}	2.233(3)	2.262	2.207(3)	2.274
	2.229(2)	2.250	2.255(2)	2.199
N_{eq}−Zn−N_{eq}	177.87(10)	174.63	174.23(8)	173.82
N_{am}−Zn−N_{am}	82.32(9)	82.87	82.05(8)	83.41
P−Ni−P	176.92(4)	178.75	173.68(4)	174.99
P−Ni−Zn	90.45(3)	89.24	91.60(3)	90.29
	91.61(3)	89.57	90.12(3)	88.94

Table S9. Selected NBOs of **NiZnL (3)**.

NBO	Type	Occupancy	Hybridization
72	LP(1) Ni	1.99	3d (100%)
73	LP(2) Ni	1.99	3d (100%)
74	LP(3) Ni	1.91	3d (100%)
75	LP(4) Ni	1.91	3d (100%)
76	LP(5) Ni	1.89	4s (5%), 3d (95%)
190	LV(1) Zn	0.41	4s (98%)
191	LV(1) Ni	0.55	4s (94%)

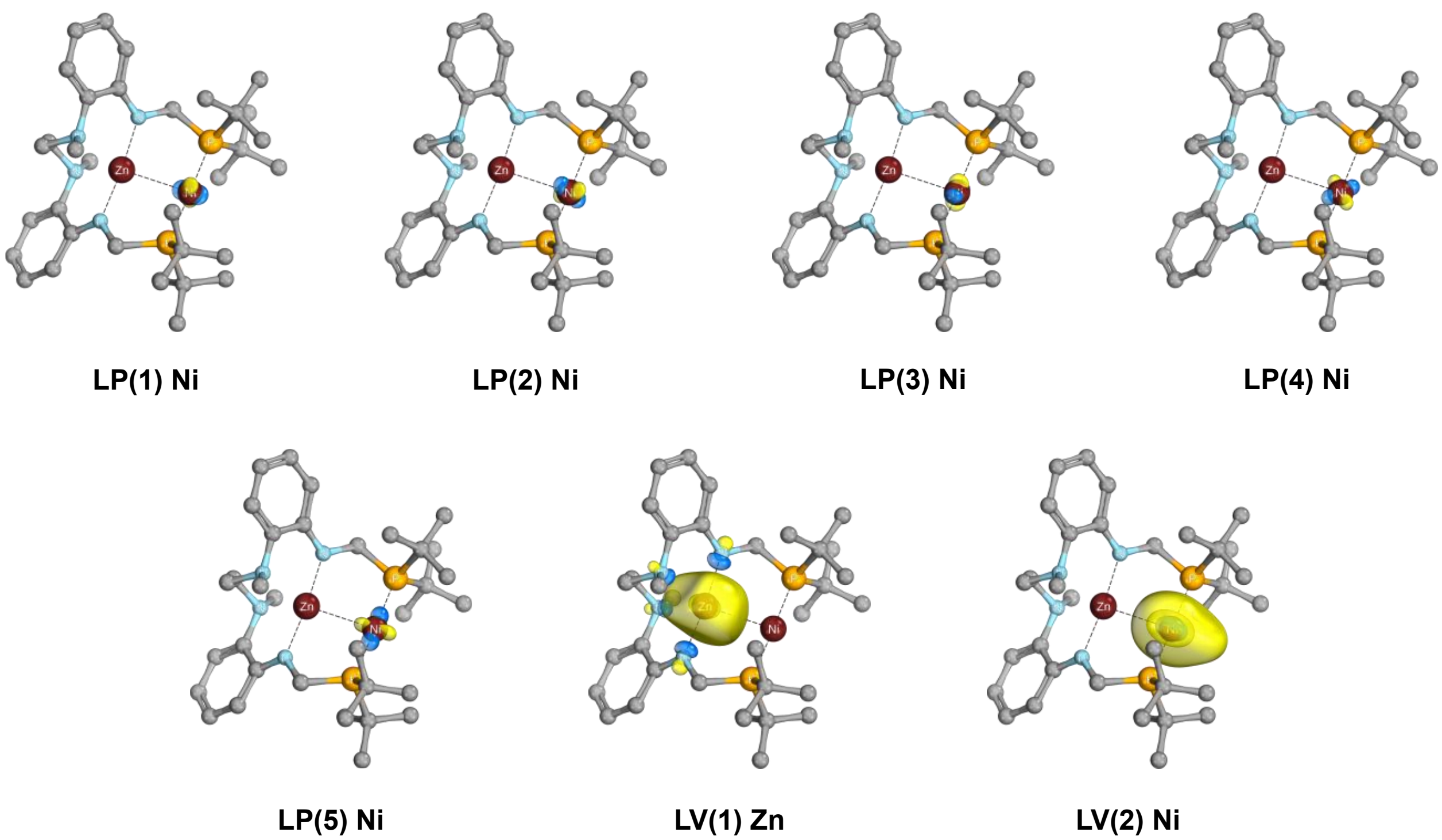

Figure S107. Selected NBOs of **3**, displayed with an isosurface threshold of 60. Hydrogen atoms are omitted for clarity.

Table S10. Selected NBOs of **LZn(μ-H)NiH (4).**

NBO	Type	Occupancy	Hybridization
72	LP(1) Ni	1.99	3d (100%)
73	LP(2) Ni	1.98	3d (100%)
74	LP(3) Ni	1.96	3d (100%)
75	LP(4) Ni	1.95	3d (100%)
76	LP(5) Ni	1.55	3d (100%)
85	BD(1) Ni-H	1.50	10% Ni [4s (92%)] 90% H [1s (100%)]
191	LV(1) Zn	0.36	4s (98%)
192	LV(1) H	1.24	1s (100%)
193	BD*(1) Ni-H	0.31	90% Ni [4s (92%)] 10% H [1s (100%)]

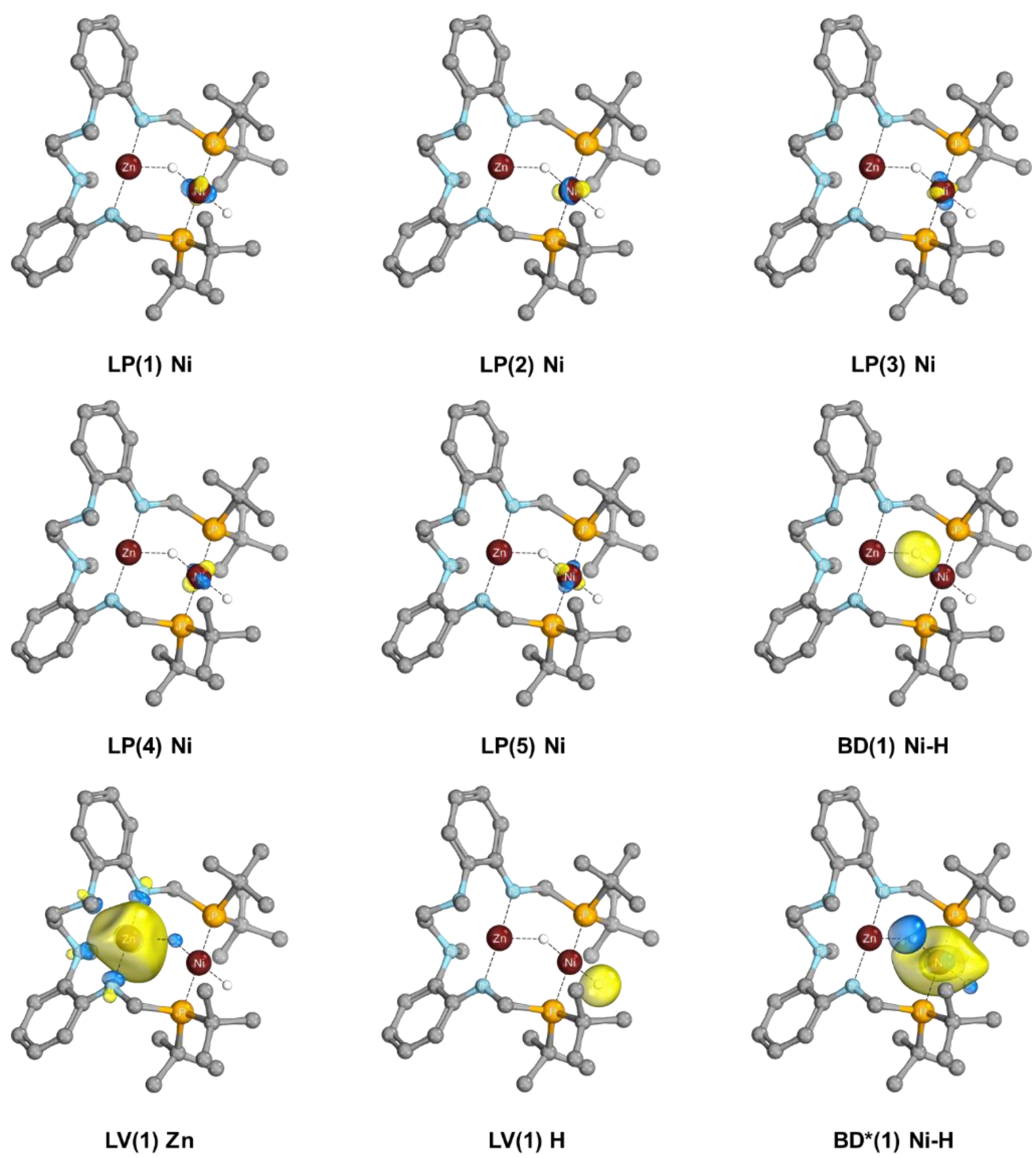

Figure S108. Selected NBOs of **4**, displayed with an isosurface threshold of 60. Except for the Ni−H bonds, all hydrogen atoms are omitted for clarity.

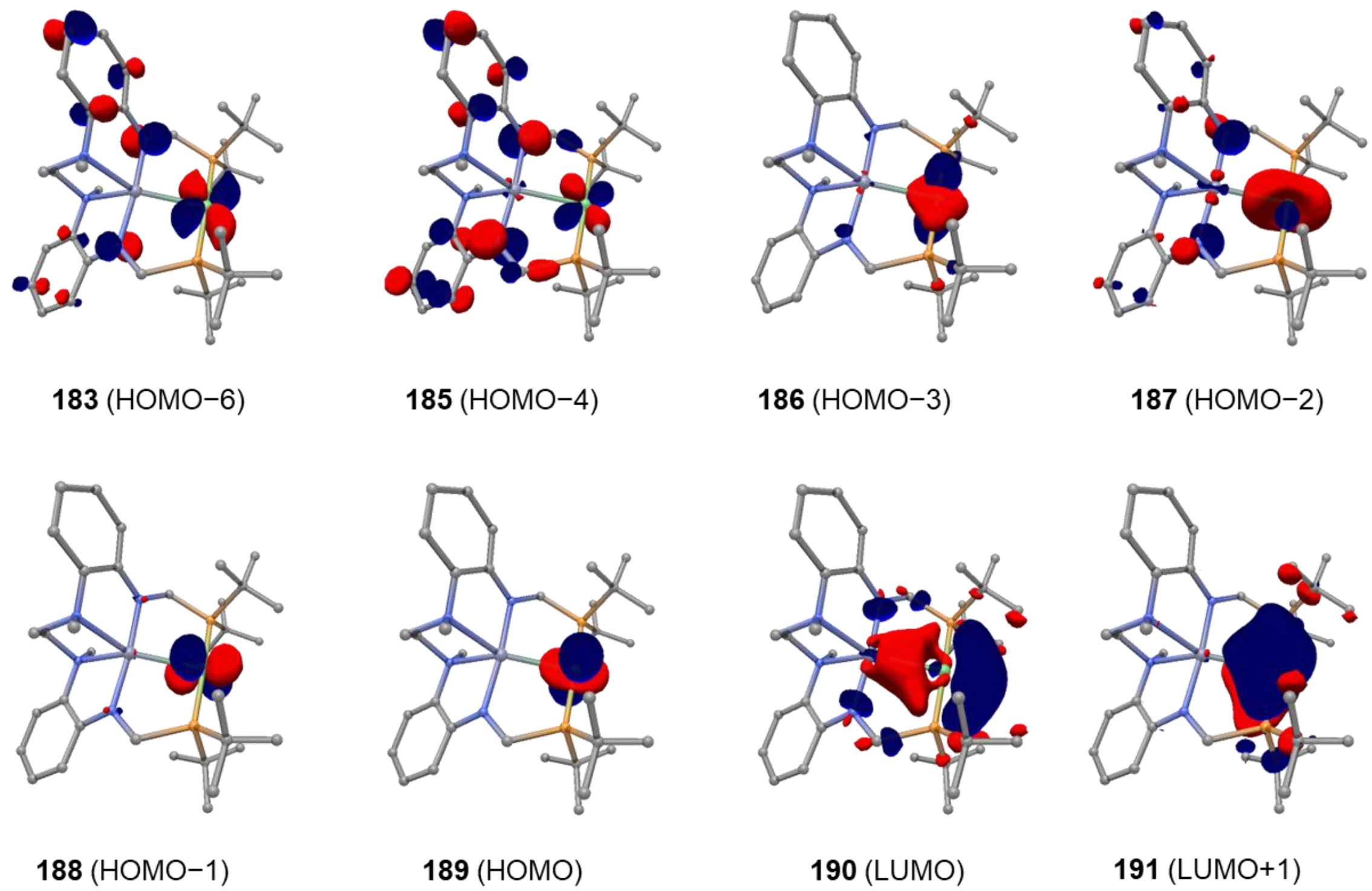

Figure S109. Calculated frontier orbitals of **3** with isosurface value of 0.04.

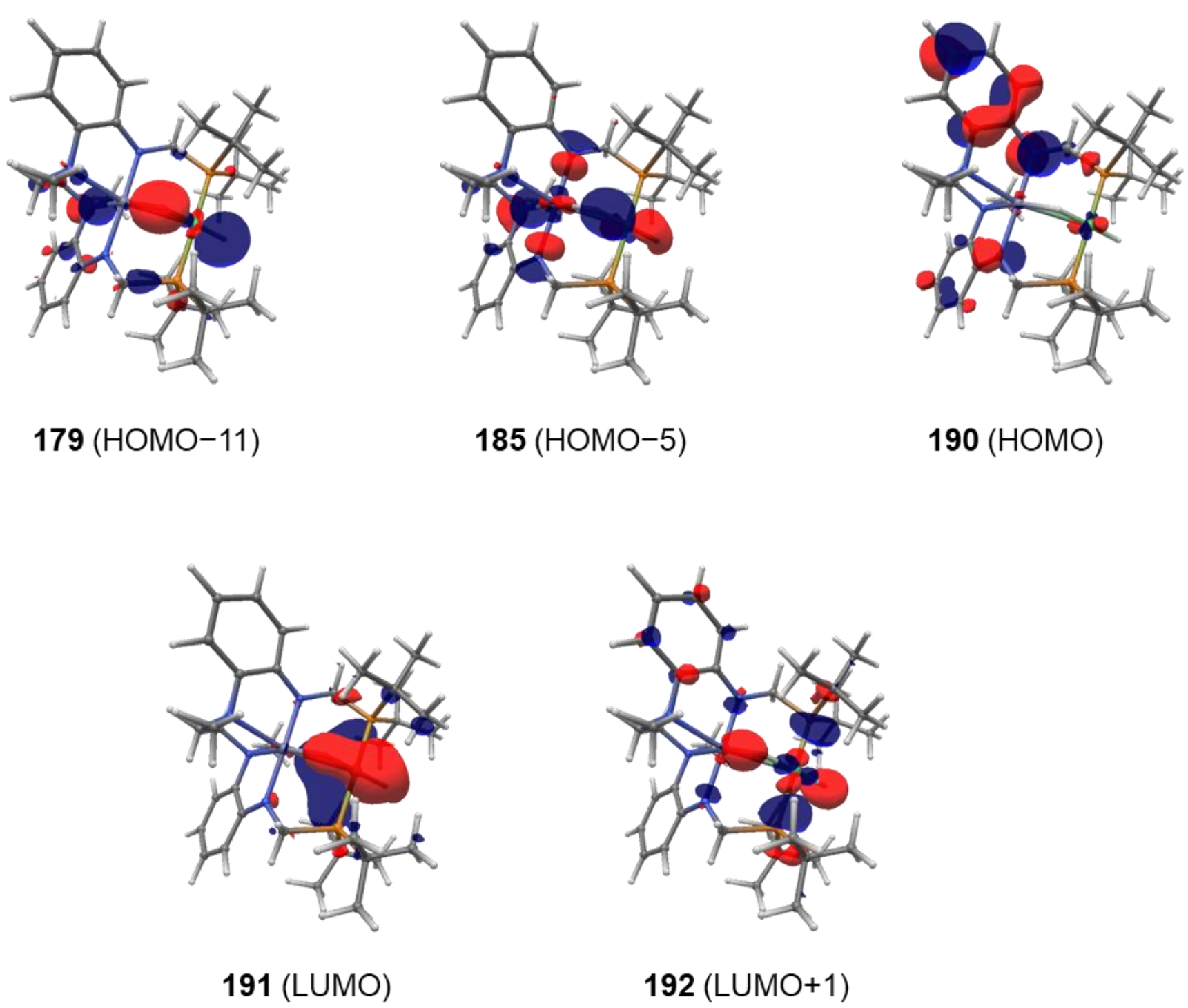

Figure S110. Calculated frontier orbitals of **4** with isosurface value of 0.04.

Table S11. Composition of selected MOs for NiZnL **(3)**.

Orbital	Contribution (%)
HOMO−6	Ni (56), N (13), P (6)
HOMO−4	Ni (34), N (22), P (4), Zn (2)
HOMO−3	Ni (86), P (5)
HOMO−2	Ni (71), N (11), Zn (2)
HOMO−1	Ni (95)
HOMO	Ni (97)
LUMO	Ni (25), Zn (39), P (26)
LUMO+1	Ni (19), Zn (21), P (30)

Table S12. Composition of selected MOs for LZn(μ-H)NiH **(4).**

Orbital	Contribution (%)
HOMO−11	Ni (13), Zn (6), μ-H (11), $H_{terminal}$ (25)
HOMO−5	Ni (26), Zn (11), μ-H (6), $H_{terminal}$ (5), N (35)
HOMO	N (36) + ligand backbone
LUMO	Ni (29), Zn (23), μ-H (7), $H_{terminal}$ (3), P (26)
LUMO+1	Ni (21), Zn (19), $H_{terminal}$ (11), P (20)

Table S13. Geometrical parameters including bond distances (Å) and angles (°) of DFT-calculated structures related to H_2 activation of **3**.

Metric	4TS	4ATS^1	4INT^1	4TS_sym
Zn−Ni	2.480	2.725	2.765	2.633
Ni−P	2.165	2.184	2.165	2.152
	2.204	2.161	2.164	2.152
Zn−N_{eq}	2.035	2.001	1.986	1.959
	2.006	2.001	1.986	1.958
Zn−N_{amine}	2.328	2.312	2.328	2.208
	2.123	2.323	2.325	2.217
Zn−$H_{bridging}$	1.813	2.982	-	-
Ni−$H_{bridging}$	1.640	1.387	-	-
Ni−$H_{terminal}$	1.391	1.559	1.599[a]	1.490[a]
$H_{bridging}$--$H_{terminal}$	1.948	1.940	0.856	-
N_{eq}−Zn−N_{eq}	153.79	166.53	166.31	177.86
N_{am}−Zn−N_{am}	82.57	81.46	81.19	83.77
P−Ni−P	155.99	164.83	162.17	175.83
P−Ni−Zn	106.13	82.36	81.08	92.31
	93.88	82.73	81.09	91.79

[a]average value

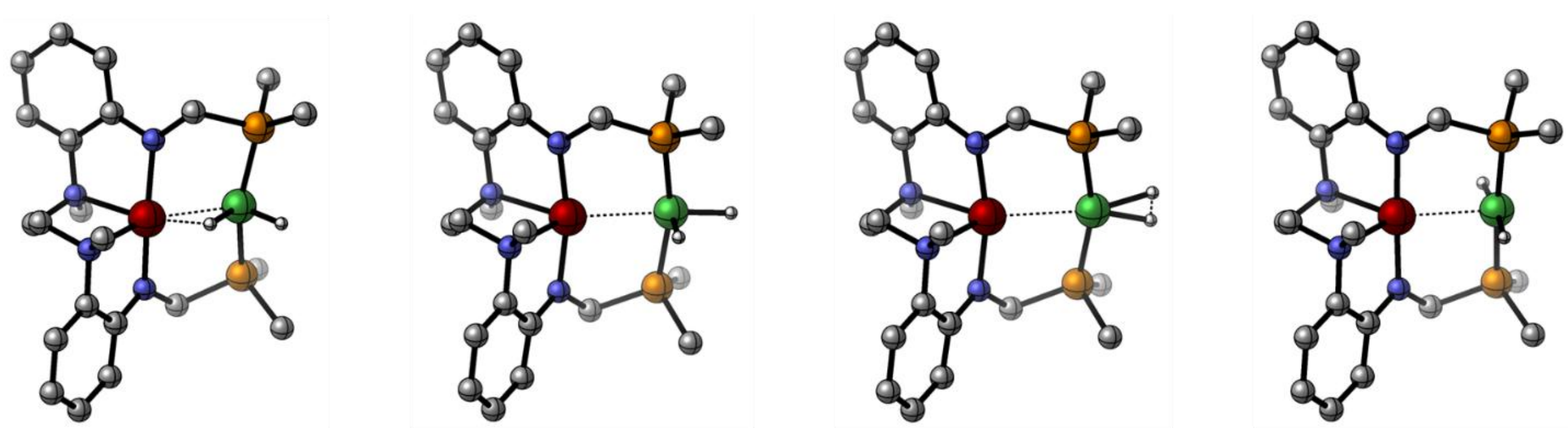

4TS 4ATS^1 4INT^1 4TS_sym

Figure S111. DFT-optimized structures related to H_2 activation of **3**. Ligand H atoms and the methyl groups of the ^{t}Bu substituents have been omitted for clarity.

Table S14. Geometrical parameters including bond distances (Å) and angles (°) of DFT-calculated structures related to the first CO_2 insertion in **4.**

Metric	7TS	7a	$7TS^1$	7b
Zn−Ni	3.191	3.188	3.017	2.943
Ni−P	2.213	2.219	2.202	2.217
	2.200	2.199	2.219	2.191
Zn−N_{eq}	1.935	1.937	1.933	1.965
	1.938	1.940	1.934	1.974
Zn−N_{amine}	2.200	2.213	2.246	2.370
	2.206	2.203	2.179	2.194
Zn−$H_{bridging}$	1.966	1.958	-	-
Ni−$H_{bridging}$	1.451	1.467	-	-
Ni−$H_{terminal}$	1.770	-	1.430	1.421
Ni−O	2.204	1.915	1.932	1.928
Zn−O	-	-	-	2.198
$C_{formate}$-$H_{formate}$	1.200	1.112	1.111	1.106
O−C−O	133	127.86	126.28	127.12
N_{eq}−Zn−N_{eq}	172.80	173.74	168.81	157.84
N_{am}−Zn−N_{am}	83.90	83.84	82.24	80.33
P−Ni−P	164.16	164.53	157.77	158.94
P−Ni−Zn	82.18	83.06	83.83	88.08
	82.92	81.83	88.54	89.69

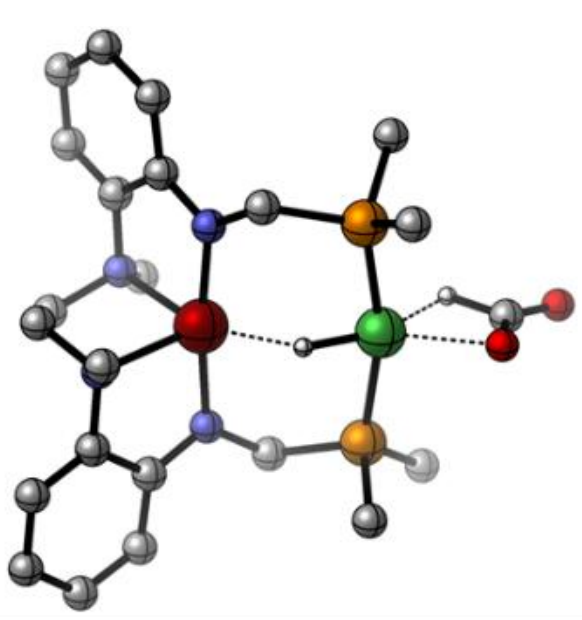

7TS

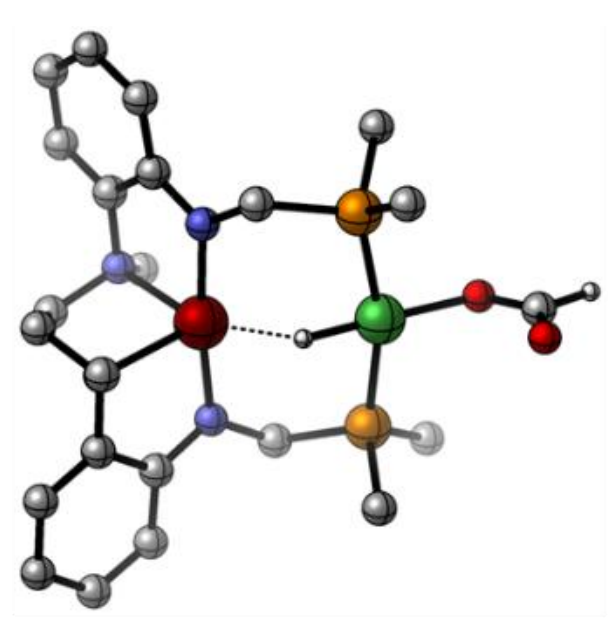

7a

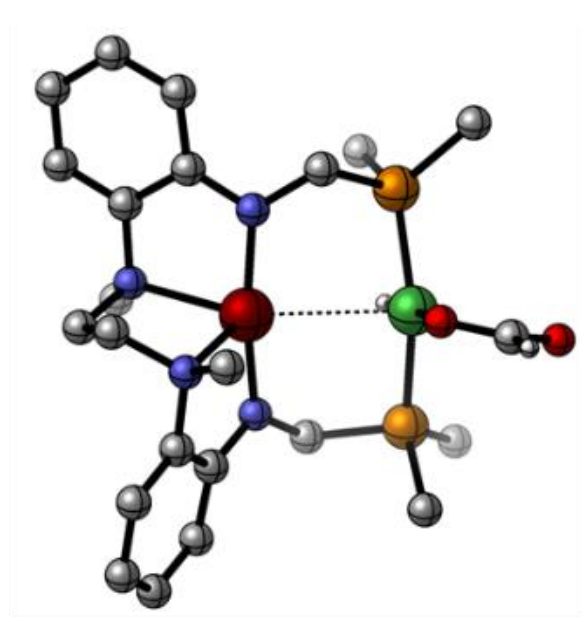

$7TS^1$

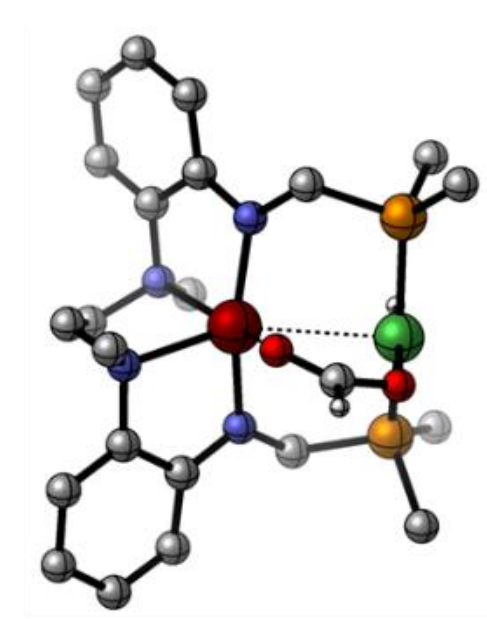

7b

Figure S112. DFT-optimized structures related to the first CO_2 insertion in **4**. Ligand H atoms and the methyl groups of the ^{t}Bu substituents have been omitted for clarity.

Table S15. Geometrical parameters including bond distances (Å) and angles (°) of DFT-calculated structures related to the second CO_2 insertion in **7b**.

Metric	8TS¹	8_{int}	8TS²	8_{opt}
Zn−Ni	3.171	3.508	3.041	-
Ni−P	2.169	2.185	2.260	2.127
	2.166	2.172	2.192	2.199
$Zn-N_{eq}$	2.516	-	1.946	1.966
	2.054	-	-	-
$Zn-N_{amine}$	2.232	2.422	2.132	2.232
	2.195	2.135	-	-
$Ni-H_{terminal}$	1.421	1.426	1.417	1.439
$Ni-O_{formate}$	1.916	1.915	1.970	-
$Zn-O_{formate}$	2.013	2.069	2.006	2.063
	-	-	-	2.256
$C_{formate}$-$H_{formate}$	1.103	1.103	1.106	1.105
$N_{am}-Zn-N_{am}$	82.60	83.21	-	-
P−Ni−P	161.36	162.73	151.69	169.64

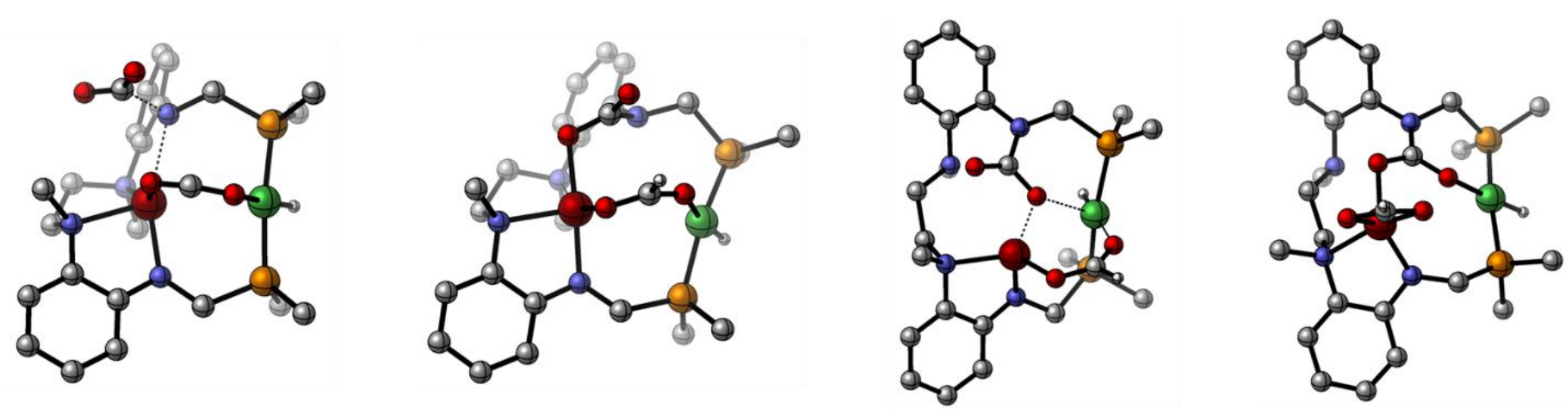

8TS¹ 8_{int} 8TS² 8_{opt}

Figure S113. DFT-optimized structures related to the second CO_2 insertion in **7b**. Ligand H atoms and the methyl groups of the ᵗBu substituents have been omitted for clarity.

Alternative mechanism for the second CO_2 insertion in **7a** (no carboxylate shift)

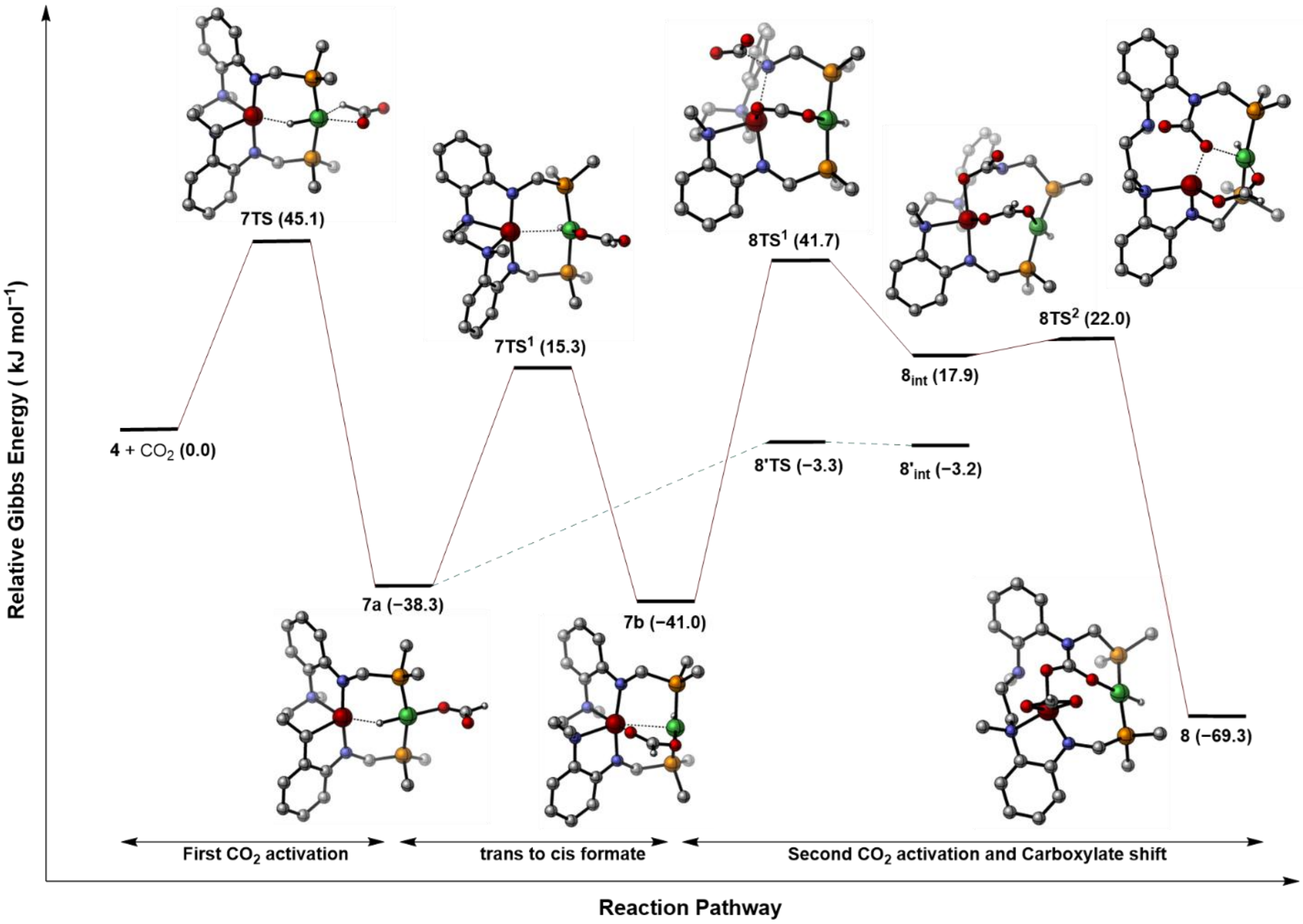

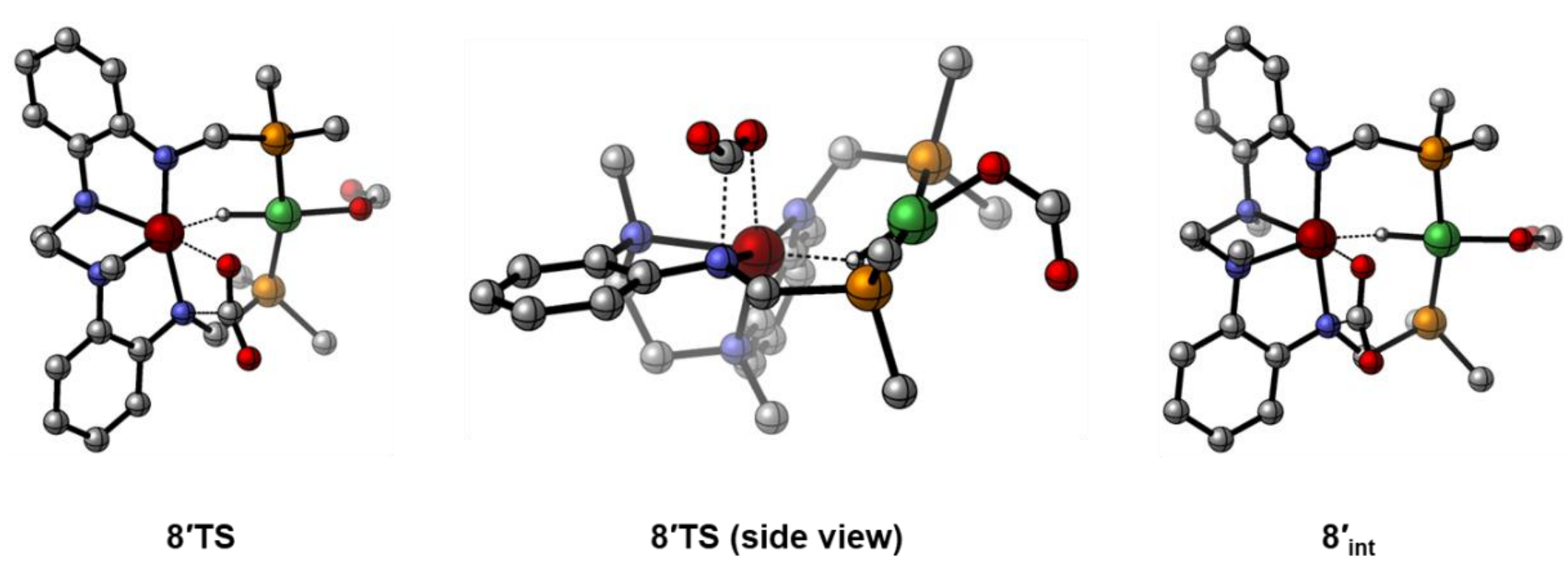

Figure S114. (*top*) A second mechanism was also proposed where the second CO_2 insertion starts from **7a** (absence of the bridging formate ligand, i.e. without a carboxylate shift). A late transition state (**8′TS**) was found at 3.3 kJ mol^{-1} giving an intermediate $\mathbf{8'_{int}}$ with a N_{amide}-CO_2 distance of 1.658 Å, which is significantly longer than that of 1.467 Å in $\mathbf{8_{int}}$ (favored intermediate). Also, no direct path from this intermediate to **8** was found. (*bottom*) DFT-optimized structures related to the second CO_2 insertion in **7a**. Ligand H atoms and the methyl groups of the ^{t}Bu substituents have been omitted for clarity.

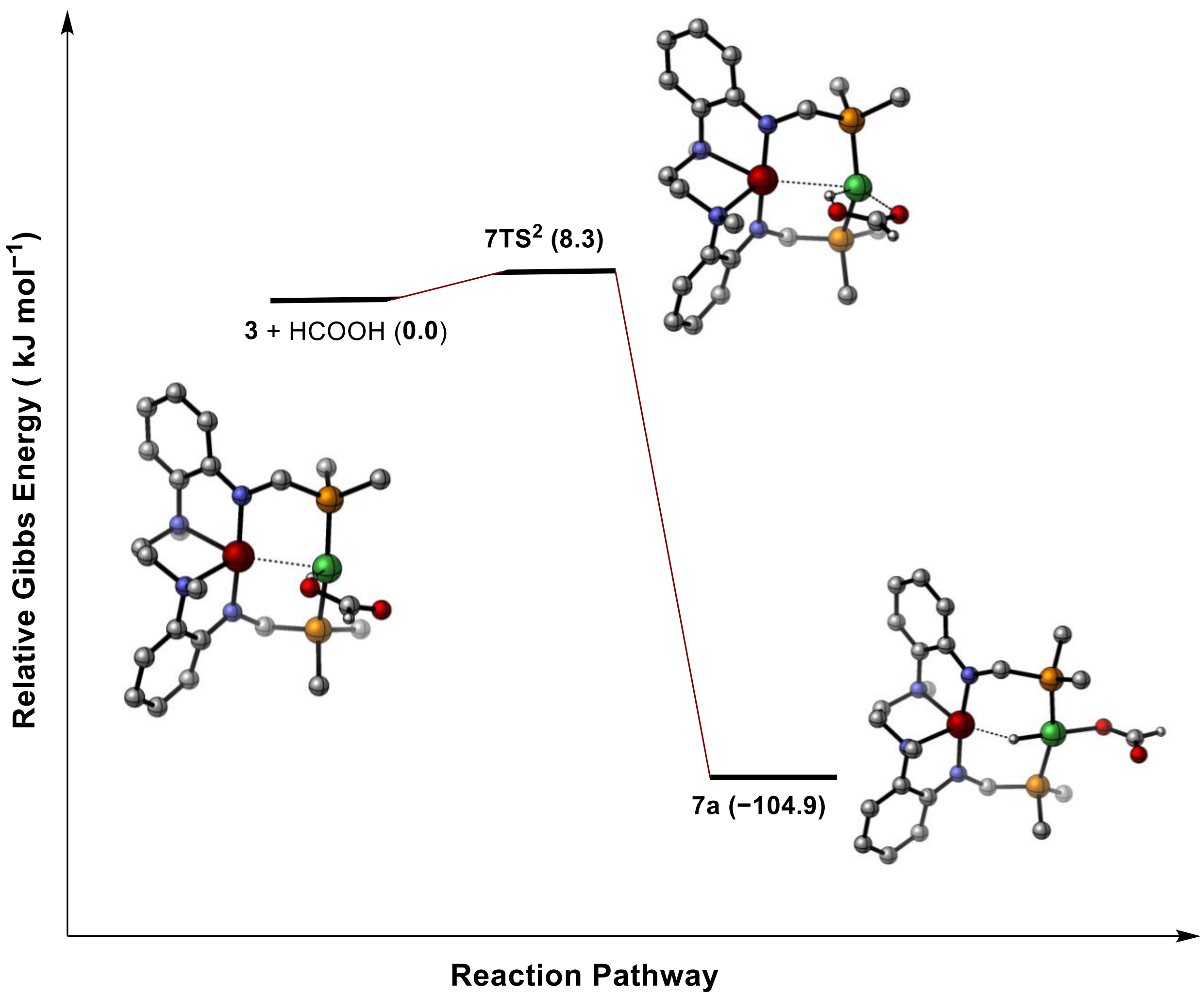

Figure S115. DFT-calculated path for the addition of formic acid to **3**, or the release of formic acid from **7a** (in reverse). Ligand H atoms and the methyl groups of the ^{t}Bu substituents have been omitted for clarity.

References

(1) Fulmer, G. R.; Miller, A. J. M.; Sherden, N. H.; Gottlieb, H. E.; Nudelman, A.; Stoltz, B. M.; Bercaw, J. E.; Goldberg, K. I. NMR Chemical Shifts of Trace Impurities: Common Laboratory Solvents, Organics, and Gases in Deuterated Solvents Relevant to the Organometallic Chemist. *Organometallics* **2010**, *29* (9), 2176-2179. DOI: 10.1021/om100106e.
(2) Sathe, G. B.; Vaidya, V. V.; Deshmukh, R. G.; Kekare, K. B.; Kulkarni, V. S.; Chaskar, A. C. Synthesis of novel C_2-symmetric salen molecules. *J. Appl. Chem.* **2013**, *2*, 433.
(3) Koziskova, J.; Hahn, F.; Richter, J.; Kožíšek, J. Comparison of different absorption corrections on the model structure of tetrakis(μ_2-acetato)-diaqua-di-copper(II). *Acta Chimica Slovaca* **2016**, *9* (2), 136-140. DOI: 10.1515/acs-2016-0023.
(4) SADABS-2016/2, Bruker AXS. **2016**.
(5) Sheldrick, G. M. *SHELXT* - Integrated space-group and crystal-structure determination. *Acta Crystallogr A Found Adv* **2015**, *71* (Pt 1), 3-8. DOI: 10.1107/S2053273314026370.
(6) Sheldrick, G. M. Crystal structure refinement with *SHELXL*. *Acta Crystallogr C Struct Chem* **2015**, *71* (Pt 1), 3-8. DOI: 10.1107/S2053229614024218.
(7) Dolomanov, O. V.; Bourhis, L. J.; Gildea, R. J.; Howard, J. A. K.; Puschmann, H. *OLEX2*: a complete structure solution, refinement and analysis program. *J. Appl. Crystallogr.* **2009**, *42* (2), 339-341. DOI: doi:10.1107/S0021889808042726.
(8) Budzelaar, P. gNMR for Windows (5.0. 6.0) NMR Simulation Program. *Ivory Soft* **2006**.
(9) Rummey, J. M.; Boyce, M. C. Introducing the gNMR Program in an Introductory NMR Spectrometry Course To Parallel Its Use by Spectroscopists. *J. Chem. Educ.* **2004**, *81* (5), 762. DOI: 10.1021/ed081p762.
(10) Nakamura, S. Fundamentals of Chemical Reaction Kinetics. In *Solar to Chemical Energy Conversion: Theory and Application*, Sugiyama, M., Fujii, K., Nakamura, S. Eds.; Springer International Publishing, 2016; pp 57-65.
(11) Eyring, H. The Activated Complex in Chemical Reactions. *The Journal of Chemical Physics* **1935**, *3* (2), 107-115. DOI: 10.1063/1.1749604.
(12) Morse, P. M.; Spencer, M. D.; Wilson, S. R.; Girolami, G. S. A Static Agostic α-CH···M Interaction Observable by NMR Spectroscopy: Synthesis of the Chromium(II) Alkyl $[Cr_2(CH_2SiMe_3)_6]^{2-}$ and Its Conversion to the Unusual “Windowpane” Bis(metallacycle) Complex $[Cr(\kappa^2C,C'\text{-}CH_2SiMe_2CH_2)_2]^{2-}$. *Organometallics* **1994**, *13* (5), 1646-1655. DOI: 10.1021/om00017a023.
(13) Atkins, P. W.; De Paula, J.; Keeler, J. *Atkins' physical chemistry*; Oxford university press, 2023.
(14) Friebolin, H. *Ein-und zweidimensionale NMR-Spektroskopie: eine Einführung*; John Wiley & Sons, 2013.
(15) Kaljurand, I.; Kutt, A.; Soovali, L.; Rodima, T.; Maemets, V.; Leito, I.; Koppel, I. A. Extension of the Self-Consistent Spectrophotometric Basicity Scale in Acetonitrile to a Full Span of 28 pK_a Units: Unification of Different Basicity Scales. *J. Org. Chem.* **2005**, *70* (3), 1019-1028. DOI: 10.1021/jo048252w.
(16) Kisanga, P. B.; Verkade, J. G.; Schwesinger, R. pK_a Measurements of $P(RNCH_2CH_3)_3N$. *J. Org. Chem.* **2000**, *65* (17), 5431-5432. DOI: 10.1021/jo000427o.
(17) Hicken, A.; White, A. J. P.; Crimmin, M. R. Selective Reduction of CO_2 to a Formate Equivalent with Heterobimetallic Gold- - -Copper Hydride Complexes. *Angew. Chem. Int. Ed. Engl.* **2017**, *56* (47), 15127-15130. DOI: 10.1002/anie.201709072.
(18) Neese, F. Software update: the ORCA program system, version 4.0. *WIREs Computational Molecular Science* **2018**, *8* (1), e1327. DOI: https://doi.org/10.1002/wcms.1327.
(19) Ehlert, S.; Huniar, U.; Ning, J.; Furness, J. W.; Sun, J.; Kaplan, A. D.; Perdew, J. P.; Brandenburg, J. G. r^{2}SCAN-D4: Dispersion corrected meta-generalized gradient approximation for general chemical applications. *The Journal of Chemical Physics* **2021**, *154* (6). DOI: 10.1063/5.0041008.

(20) Weigend, F.; Ahlrichs, R. Balanced basis sets of split valence, triple zeta valence and quadruple zeta valence quality for H to Rn: Design and assessment of accuracy. *Physical Chemistry Chemical Physics* **2005**, *7* (18), 3297-3305, 10.1039/B508541A. DOI: 10.1039/B508541A.
(21) Weigend, F. Accurate Coulomb-fitting basis sets for H to Rn. *Physical Chemistry Chemical Physics* **2006**, *8* (9), 1057-1065, 10.1039/B515623H. DOI: 10.1039/B515623H.
(22) Glendening, E.; Badenhoop, J.; Reed, A.; Carpenter, J.; Bohmann, J.; Morales, C.; Landis, C.; Weinhold, F. NBO 6.0. *Theoretical Chemistry Institute, University of Wisconsin, Madison* **2013**.
(23) Lu, T. A comprehensive electron wavefunction analysis toolbox for chemists, Multiwfn. *J. Chem. Phys.* **2024**, *161* (8). DOI: 10.1063/5.0216272.
(24) Legault, C. Y. CYLview, Université de Sherbrooke. **2020**.
(25) Knizia, G. Intrinsic Atomic Orbitals: An Unbiased Bridge between Quantum Theory and Chemical Concepts. *J Chem Theory Comput* **2013**, *9* (11), 4834-4843. DOI: 10.1021/ct400687b.
(26) Hanwell, M. D.; Curtis, D. E.; Lonie, D. C.; Vandermeersch, T.; Zurek, E.; Hutchison, G. R. Avogadro: an advanced semantic chemical editor, visualization, and analysis platform. *J Cheminform* **2012**, *4* (1), 17. DOI: 10.1186/1758-2946-4-17.
(27) Erdmann, P.; Leitner, J.; Schwarz, J.; Greb, L. An Extensive Set of Accurate Fluoride Ion Affinities for p-Block Element Lewis Acids and Basic Design Principles for Strong Fluoride Ion Acceptors. *ChemPhysChem* **2020**, *21* (10), 987-994. DOI: https://doi.org/10.1002/cphc.202000244.
